# Hyperin Alleviates Triptolide-Induced Ovarian Granulosa Cell Injury by Regulating AKT/TSC1/mTORC1 Signaling

**DOI:** 10.1155/2021/9399261

**Published:** 2021-10-18

**Authors:** Fang You, Junyan Cao, Li Cheng, Xiaogu Liu, Li Zeng

**Affiliations:** The Second Clinical College, Guizhou University of Chinese Medicine, Guiyang 550000, China

## Abstract

Premature ovarian insufficiency (POI) is characterized by the loss of ovarian function before 40 years of age and affects approximately 1% of women worldwide. *Caragana sinica* is a traditional Miao (a Chinese ethnic minority) medicine that improves ovarian function and follicular development. In the present study, we aimed to investigate the effect of active ingredients of *C. sinica* on POI and determine underlying mechanisms. Herein, the chemical composition of the *C. sinica* compound was analyzed using ultra-high-performance liquid chromatography, which identified hyperin (HR) as one of the main ingredients in *C. sinica*. Then, interaction targets of HR and POI were predicted and analyzed using network pharmacology and bioinformatics. The effect of HR on triptolide (TP)-induced granulosa cell injury was evaluated, and the underlying mechanism was explored based on bioinformatic results. A total of 100 interaction targets for POI and HR were obtained. The protein-protein interaction network of identified interaction targets emphasized the topological importance of AKT1. Kyoto Encyclopedia of Genes and Genomes (KEGG) analysis revealed that HR might regulate POI by modulating the mechanistic target of rapamycin (mTOR) signaling pathway. In addition, the KEGG graph of the mTOR signaling pathway revealed that AKT phosphorylation inhibits the TSC1/2, while TSC1/2 activation inhibits the expression of mTORC1. The fundamental experiment revealed that HR increased proliferation, progesterone receptor levels, and estradiol levels decreased by TP in KGN cells. Additionally, HR alleviated TP-induced apoptosis and G1/G1 phase arrest in KGN cells. Western blotting demonstrated that HR increased the phosphorylation of AKT and mTORC1 and decreased TSC1 expression in TP-induced KGN cells. Collectively, our findings revealed that HR alleviates TP-induced granulosa cell injury by regulating AKT/TSC1/mTORC1 signaling, providing insight into the treatment of POI.

## 1. Introduction

Premature ovarian insufficiency (POI), previously known as premature menopause or premature ovarian failure, is characterized by the loss of ovarian function before the age of 40 years and is mainly manifested as menstrual cessation, decreased androgen, and elevated gonadotropin. Meanwhile, it can lead to a series of health problems such as vascular disease, decreased bone mineral density, and decreased fertility, which seriously threatens human health [1]. And it is reported to affect about 1% of women worldwide [2, 3]. The presently available POI treatments include stem cell treatment, assisted reproductive technology, and hormonal replacement therapy. However, these treatments are limited to clinical application, and improved therapeutic strategies are urgently needed [4, 5].

In recent years, medicinal plants are widely studied [6–8], some of which have anticancer activity [9], natural anthraquinone derivatives have immunomodulatory, antibacterial, and anti-inflammatory properties [10], and many studies have proved that flavonoids can act as antioxidants to prevent the degradation of antioxidants and age-related cellular components [11]. Traditional Chinese medicine has been employed for treating POI and has achieved excellent efficacy [12]. *Caragana sinica* is a traditional Miao (Chinese ethnic minority) medicine containing *C. sinica*, *Hominis placenta*, glossy privet fruit, and *Eclipta*, and it has many pharmacological activities, such as analgesia, antioxidation, anti-inflammatory, avoiding microthrombosis, regulating immune function, inhibiting tumor cell growth, and so on [13]. Previously, we reported that *C. sinica* compound promotes the ovulation rate, as well as follicle growth and development in patients with a decline in ovarian reserve [14]. *C. sinica* improves ovarian function and follicular development by regulating the secretion of endogenous hormones [15, 16]. The pharmacological activity of *C. sinica* depends on its chemical composition. However, the active ingredients of *C. sinica* and its effect on POI have not been thoroughly investigated.

Clinical symptoms of POI primarily occur in response to dysregulated folliculogenesis and estrogen deficiency, including vulvovaginal atrophy, oligomenorrhea, amenorrhea, infertility, and vasomotor instability [10, 17]. Abnormal follicular atresia, a process characterized by follicular degeneration during growth and development, might enhance follicular depletion and result in ovarian insufficiency [10]. The decrease of ovarian reserve is related to the decrease of the number or quality of follicles and oocytes [18]. Granulosa cells refer to parietal cells wrapped on the surface of follicles, which can not only promote the formation and development of follicles, but also promote adenohormones to maintain the normal function of ovary [19]. It is reported that follicular development is accompanied by the proliferation of granulosa cells, which is regulated by the interaction between granulosa cells and oocytes [20–23]. Accumulating evidence has revealed that granulosa cell injury and apoptosis are major causes of follicular atresia [24–27]. The mechanism of affecting hormone synthesis and apoptosis of ovarian granulosa cells is complex, which is closely related to the regulation of ovarian function. It has been found that PI3K/PTEN/AKT and TSC/mTORC1 signaling pathways are key regulators of ovarian function, including quiescence, activation and survival of primordial follicles, proliferation and differentiation of granulosa cells, and meiotic maturation of oocytes [28, 29]. Tuberous sclerosis complex (TSC) 1/2 complex is considered to be the key regulator of mTOR activity. TSC1/2 plays an important role in the homeostasis and differentiation of immune cells through the negative regulation of mTOR signal pathway [30, 31].

In the present study, we analyzed the chemical composition of the *C. sinica* compound using ultra-high-performance liquid chromatography (UHPLC). Targets of hyperin (HR; one of the active ingredients of *C. sinica* compound) and POI were predicted and analyzed using network pharmacology and bioinformatics. Studies have proved that *Tripterygium wilfordii* has reproductive toxicity and can cause ovarian dysplasia and dysfunction. In this study, triptolide, one of the active ingredients of *Tripterygium wilfordii*, was used to induce ovarian granulosa cells injury, simulating the apoptosis or dysfunction of ovarian granulosa cells caused by ovarian dysfunction in vivo [32, 33]. The TP-induced granulosa cells were then treated with HR to evaluate its effect on POI. Additionally, the underlying mechanism was explored based on bioinformatic results.

## 2. Materials and Methods

### 2.1. Detection of HR Content in *C. sinica* Compound

**Figure 1:**
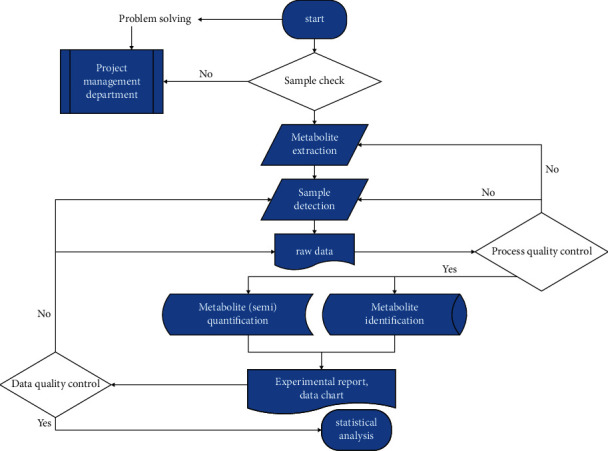
Extraction process of HR.

*C. sinica* compound granules, containing *C. sinica*, *Hominis placenta*, glossy privet fruit, and *Eclipta*, were supplied by Xinlvyao (Sichuan, China). UHPLC was performed to measure the active components present in *C. sinica*. The flowchart is shown in Figure 1. A 300 *μ*l aliquot of *C. sinica* compound was extracted with 1000 *μ*l methanol (80%) by vortex mixing for 30 s, with ice-water bath ultrasonic treatment performed for 5 min. After maintaining at −20°C for 1 h and centrifugation at 12000 rpm (4°C for 15 min), a 5 *μ*l aliquot of the supernatant was analyzed using a Nexera UHPLC LC-30A apparatus (Shimadzu, Japan), fitted with a UPLC BEH C18 Column (1.7 *μ*m, 2.1 mm × 100 mm; Waters Corporation). The mobile phase was composed of water-acetonitrile (gradient elution), maintained at a flow rate of 400 *μ*l/min. Primary and secondary mass spectra data were obtained using an AB 5600 Triple TOF mass spectrometer controlled by Analyst TF 1.7 software (AB Sciex, USA).

### 2.2. Network Construction and Analysis

For HR, target proteins were predicted using the TCMSP database (https://tcmspw.com/tcmsp.php), Swiss Target Prediction database (http://www.swisstargetprediction.ch/), and Pharm Mapper database (http://www.lilab-ecust.cn/pharmmapper/). The corresponding gene symbols were obtained through alignment with UniProtID in the UniProt database (https://www.uniprot.org/). Subsequently, POI-related targets were identified using the GeneCards database (https://www.genecards.org/). The interaction targets for POI and HR were screened with R software using the Venn Diagram package. Cytoscape (3.6.1) was subsequently used to visualize a protein-protein interaction (PPI) network based on the obtained interaction targets. Degree, betweenness centrality, and closeness centrality were analyzed using the Network Analyzer plugin to evaluate the topological importance of the nodes in the network. Finally, the interaction targets were analyzed by bioinformatics annotation (Kyoto Encyclopedia of Genes and Genomes, KEGG, https://www.kegg.jp) using DAVID: Functional Annotation Tolls.

### 2.3. Cell Culture and Treatment

The human ovarian granular cell line KGN (Procell) was cultured in Dulbecco's modified Eagle's medium (DMEM)/F12 (Hyclone, USA), supplemented with 10% fetal bovine serum (FBS; Gibco, USA) and maintained at 37°C with 5% CO_2_. On reaching 80–90% confluency, KGN cells were treated with different concentrations of HR (0 (control), 0.1, 1, 10, 50, and 100 *μ*g/ml; Aladdin, China) for 12, 24, and 48 h and TP (0 (control), 1, 5, 10, 20, 50, and 100 nM; Aladdin, China) for 24 h to detect cytotoxicity. Next, cells were treated with 50 nM TP for 24 h and subsequently treated with different concentrations of HR (1, 10, and 50 *μ*g/ml) for 24 h. Cell viability, apoptosis, and cell cycle were evaluated.

### 2.4. Cell Counting Kit-8 (CCK-8)

The harvested cells were seeded into 96-well plates at a density of 3 × 10^3^ cells per well (100 *μ*l) at 37°C in 5% CO_2_ overnight. After the different treatments, cells were cultured with an additional 10 *μ*l CCK-8 solution (Solarbio, China) for 4 h at 37°C. Finally, the absorbance of each well was measured at 450 nm using a microplate reader (Allsheng, China).

### 2.5. Flow Cytometry Assay

Flow cytometry was used to detect apoptosis and the cell cycle. For the apoptosis assay, the Annexin V-fluorescein isothiocyanate (FITC)/propidium iodide (PI) Apoptosis Detection Kit (BD, China) was used. In detail, 1 × 10^6^ resuspended cells were harvested from each group and then centrifuged for 5 min at 400 ×*g* and 4°C (repeated twice). The cells were then resuspended in 200 *μ*l phosphate-buffered saline (PBS) and stained for 30 min with 10 *μ*l Annexin V-FITC and 10 *μ*l PI at 4°C in the dark. Following the addition of 300 *μ*l PBS, the cells were subjected to flow cytometry (ACEA Biosciences, USA). For the cell cycle assay, 1 × 10^7^ resuspended cells were harvested from each group, centrifuged at 400 ×*g* for 5 min at 4°C, and resuspended in 300 *μ*l PBS. The cells were then fixed for 24 h in an additional 700 *μ*l absolute ethyl alcohol at −20°C and centrifuged at 700 ×*g* for 5 min at 4°C. Subsequently, the cells were resuspended in 100 *μ*l of 1 mg/ml RNase A solution (BD, China) and maintained at 37°C for 300 min. Thereafter, 400 *μ*l and 50 *μ*g/ml of PI were added and cultured at 4°C for 10 min in the dark. Finally, cells were subjected to flow cytometry (ACEA Biosciences, USA). Finally, ImageJ was used to analyze the apoptosis and cell cycle of each group.

### 2.6. Enzyme-Linked Immunosorbent Assay (ELISA)

According to the antigen or antibody, it binds to the surface of a solid-phase carrier and maintains its immune activity. Connect the antigen or antibody with an enzyme to form an enzyme labeled antigen or antibody, which retains both its immune activity and the activity of the enzyme. During the determination, the tested sample (determining the antibody or antigen) and the enzyme labeled antigen or antibody react with the antigen or antibody on the surface of the solid carrier according to different steps. The antigen antibody complex formed on the solid-phase carrier is separated from other substances by washing. Finally, the amount of enzyme combined on the solid-phase carrier is in proportion to the amount of tested substances in the sample. After adding the substrate of enzyme reaction, the substrate is catalyzed by enzyme to become colored products. The amount of products is directly related to the amount of tested substances in the sample, so it can be analyzed qualitatively or quantitatively according to the depth of color reaction. Levels of progesterone receptor (HM10675) and estradiol (HM10669) were determined by ELISA kit (Bioswamp, China). The measurement steps are carried out in strict accordance with the instructions.

### 2.7. Western Blotting

Whole proteins were extracted from KGN cells using radioimmunoprecipitation assay lysis buffer (Solarbio), and the protein concentration was quantified using a bicinchoninic acid assay kit (Solarbio). In brief, 20 *μ*g of proteins in each group was separated by 12% sodium dodecyl sulfate-polyacrylamide gel electrophoresis and transferred onto polyvinylidene fluoride membranes (Millipore, USA). The membranes were then blocked with 5% skim milk and cultured for 1 h with primary antibodies against TSC1 (Bioswamp, China), mTORC1 (Bioswamp, China), and phosphorylated (p)-mTORC1 (Abcam. USA), AKT (Bioswamp, China), p-AKT (Cell Signaling Technology, USA), and GAPDH (housekeeping control, Bioswamp, China), followed by 1 h of incubation with goat anti-rabbit IgG secondary antibody (Bioswamp, China).

### 2.8. Statistical Analysis

SPSS 23.0 software was used for one-way ANOVA. Data are presented as means ± standard deviation (SD). Differences among groups were analyzed using one-way analysis of variance followed by Tukey's test. Statistical significance was set at *p* < 0.05.

## 3. Results

### 3.1. HR Was the Main Ingredient Detected in *C. sinica* Compound and Regulated POI by Modulating the AKT/TSC1/mTORC1 Signaling Pathway

**Figure 2:**
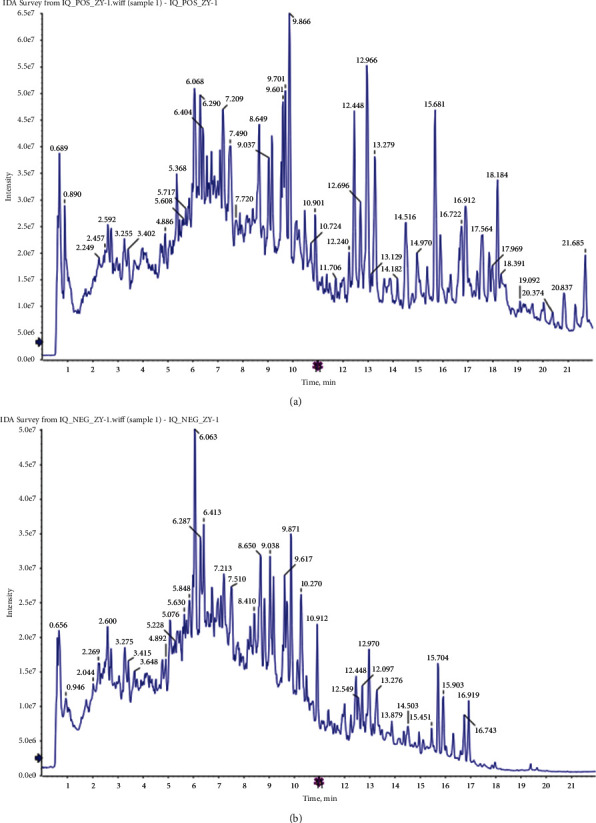
Total ion flow diagram of broom compound in positive (a) and negative (b) ion modes.

**Figure 3:**
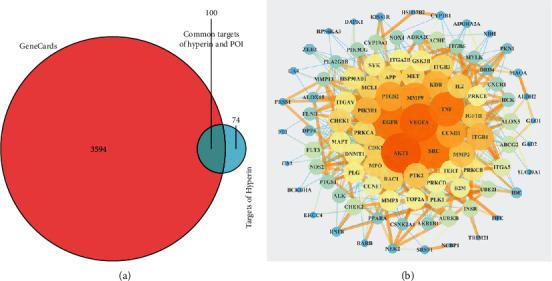
(a) Overlap of HR predicted targets and the POI-related targets. (b) The protein-protein interaction network of the predicted interaction targets of HR and POI. HR, hyperin; POI, premature ovarian insufficiency.

**Figure 4:**
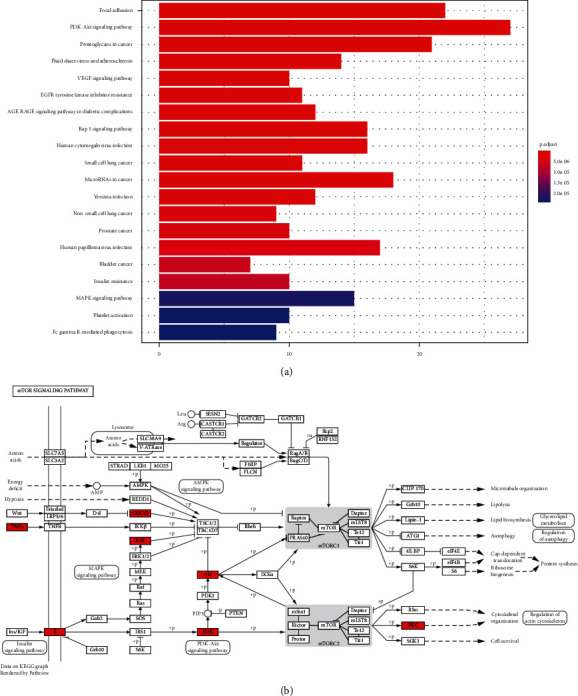
(a) Top 20 enriched KEGG pathways for the predicted interaction targets of HR and POI. (b) The KEGG graph of the mTOR signaling pathway (proteins in the red frame are interaction targets of POI and HR in the mTOR signaling pathway). KEGG, Kyoto Encyclopedia of Genes and Genomes; HR, hyperin; POI, premature ovarian insufficiency; mTOR, mechanistic target of rapamycin.

The principal components of compound Canary flower were analyzed by HPLC-MS.  Figure 2 shows the total ion flow diagram of broom compound in positive and negative ion modes. The main active ingredients identified in the *C. sinica* compound are shown in Table 1, including HR. Overall, 174 target proteins were predicted for HR using the TCMSP database, Swiss Target Prediction database, and Pharm Mapper database predicted (Supplementary 1). A total of 3694 targets related to POI were predicted using the GeneCards database (Supplementary 2). Accordingly, 100 interaction targets for POI and HR were obtained, as shown in the Venn diagram (Figure 3(a)), and the details are shown in Supplementary 3. The PPI of the interaction targets highlighted the topological importance of AKT1, as demonstrated by the maximum degree value, betweenness centrality, and closeness centrality (Figure 3(b) and Supplementary 4). To better understand the biological effects of HR, KEGG pathway enrichment was analyzed based on predicted targets. The enrichment plots of the top 20 KEGG pathways are shown in Figure 4(a), containing the PI3K-AKT signaling pathway; the details of predicted pathways are listed in Supplementary 5, containing the mechanistic target of rapamycin (mTOR) signaling pathway (Figure 4(b)). As shown in Figure 2(b), the proteins in the red frame were the interaction targets of POI and HR in the mTOR signaling pathway, containing proteins such as GSK3B, PI3K, and AKT. The KEGG graph for the mTOR signaling pathway showed that AKT phosphorylation inhibits the activation of TSC1/2, while TSC1/2 activation inhibits mTORC1 expression (Figure 4(b)).

### 3.2. HR Promoted KGN Cell Proliferation Reduced by TP

**Figure 5:**
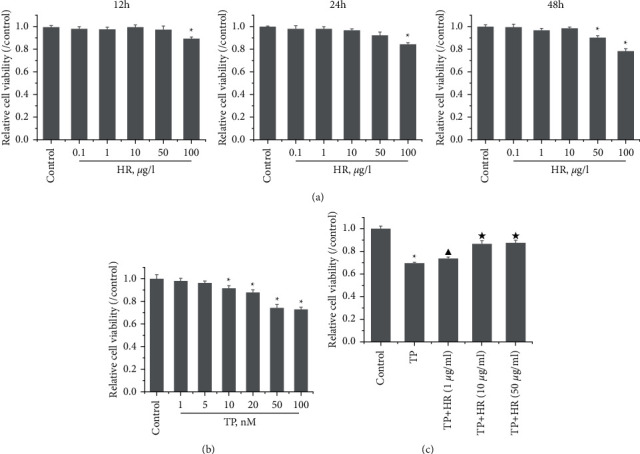
CCK-8 was performed to detect the viability of KGN cells treated with (a) HR or (b) TP alone, or (c) their combination. Data represent as mean ± standard deviation (SD), *n* = 3. ^*∗*^*p*<0.05 *vs*. control group, ^▲^*p* < 0.05*vs*. TP group, and ^★^*p*<0.05 *vs*. TP + HR (1 *μ*g/ml) group. HR, hyperin; TP, triptolide.

To investigate the effect of HR on TP-induced injury in KGN cells, we first evaluated the effect of HR on normal KGN cells. The CCK-8 assay showed that, at a low concentration (no more than 50 *μ*g/l), HR presented no cytotoxicity toward KGN cells after treatment for 12 and 24 h. However, an HR concentration exceeding 50 *μ*g/l revealed cytotoxicity after treatment for 48 h (Figure 5(a)). Thus, 1, 10, and 50 *μ*g/l of HR treatment for 24 h were selected for subsequent experiments. Next, to assess the cytotoxicity of TP on KGN cells, KGN cells were treated with different TP concentrations. The results demonstrated that a TP concentration of more than 10 nM was cytotoxic. Compared with control KGN cells, the cell viability of cells treated with 10 or 20 nM TP presented a statistical difference; the cell viability remained high (Figure 5(b)). Thus, 50 nM TP was selected for subsequent experiments. Then, the KGN cells were treated with 50 nM TP combined with 1, 10, and 50 *μ*g/l of HR. The CCK-8 assay revealed that TP decreased the viability of KGN cells; HR increased viability in a dose-dependent manner (Figure 5(c)).

### 3.3. HR Alleviated Apoptosis and G1/G1 Phase Arrest Induced by TP in KGN Cells

**Figure 6:**
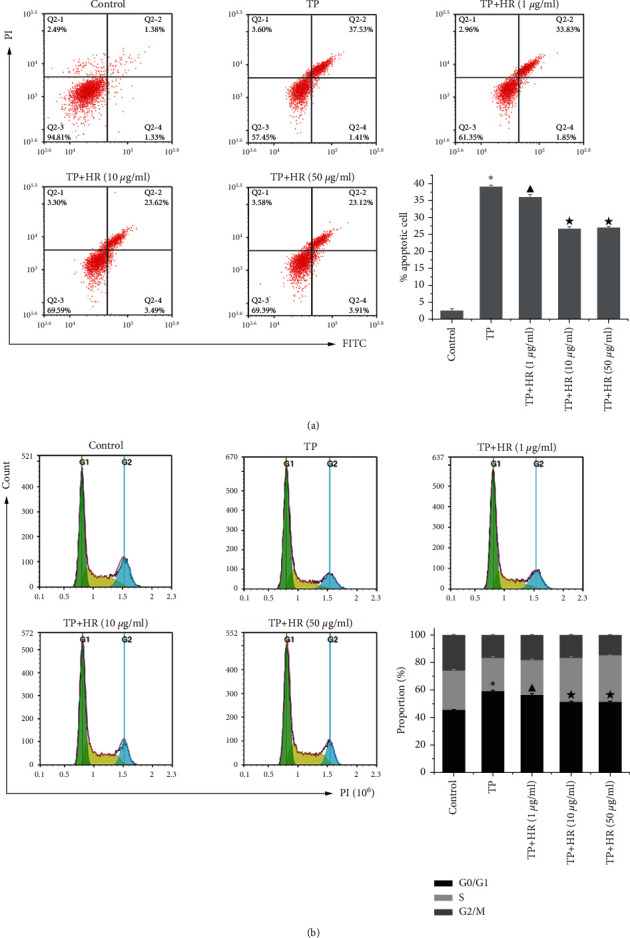
Flow cytometry was performed to detect the (a) apoptosis and (b) cycle of KGN cells treated with TP and HR combination. Data represent as mean ± standard deviation (SD), *n* = 3. ^*∗*^*p*<0.05 *vs*. control group, ^▲^*p* < 0.05 vs. TP group, and ^★^*p*<0.05 vs. TP + HR (1 *μ*g/ml) group. HR, hyperin; TP, triptolide.

Flow cytometry revealed that TP promoted apoptosis (Figure 6(a)) and induced G1/G1 phase arrest (Figure 6(b)) in KGN cells; HR alleviated these TP-induced effects in a dose-dependent manner.

### 3.4. HR Increased Levels of Progesterone Receptor and Estradiol Decreased by TP in KGN Cells

**Figure 7:**
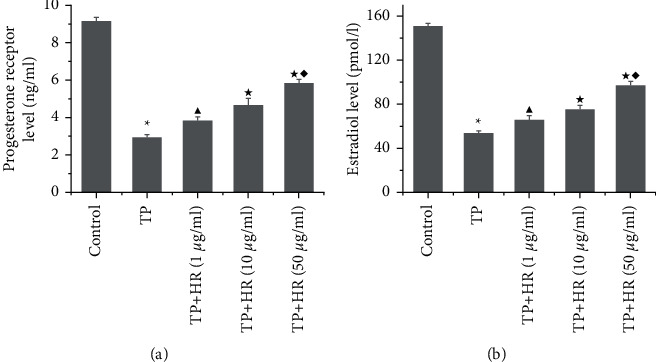
Enzyme-linked immunosorbent assay was performed to evaluate the (a) progesterone receptor and (b) estradiol levels in KGN cells treated with TP and HR combination. Data represent as mean ± standard deviation (SD), *n* = 3. ^*∗*^*p*<0.05 vs. control group, ^▲^*p* < 0.05*vs*. TP group, ^★^*p*<0.05 *vs*. TP + HR (1 *μ*g/ml) group, and ^◆^*p* < 0.05*vs*. TP + HR (10 *μ*g/ml) group. HR, hyperin; TP, triptolide.

Based on ELISA findings, TP treatment decreased the levels of progesterone receptors (Figure 7(a)) and estradiol (Figure 7(b)) in KGN cells, which were increased by HR in a dose-dependent manner.

### 3.5. HR Increased p-AKT and p-mTORC1 Activation and Decreased TSC1 Expression in TP-Induced KGN Cells

**Figure 8:**
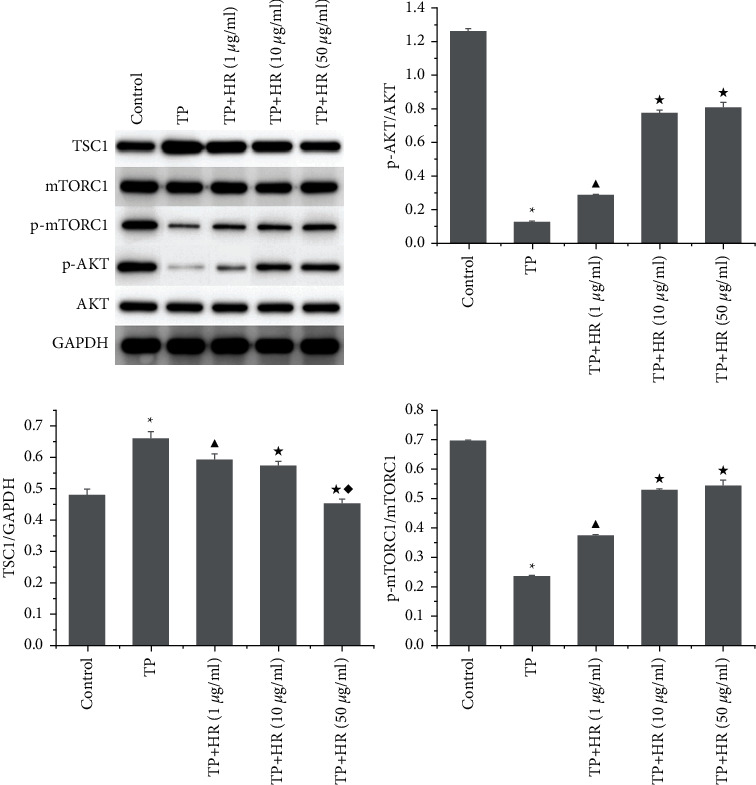
Western blot was performed to detect the expression of TSC1, mTORC1, p-mTORC1, AKT, and p-AKT in KGN cells treated with TP and HR combination. Data represent as mean ± standard deviation (SD), *n* = 3. ^*∗*^*p*<0.05 vs. control group, ^▲^*p* < 0.05*vs*. TP group, ^★^*p*<0.05 *vs*. TP + HR (1 *μ*g/ml) group, and ^◆^*p* < 0.05*vs*. TP + HR (10 *μ*g/ml) group. HR, hyperin; TP, triptolide.

Western blotting revealed that TP treatment inhibited the phosphorylation of AKT and mTORC1, which increased the expression of TSC1 in KGN cells. This phenomenon was reversed following HR treatment (Figure 8).

## 4. Discussion

POI refers to early menstruation to stop, and anovulation and decreased estrogen levels according to previous research have shown that its pathogenesis has strong genetic background and high heterogeneity; the genetic factors were involved in the X chromosome abnormalities, gene mutation, mitochondrial dysfunction, etc., and chromosome abnormality is one of the main causes of POI [34] and has become an important factor of the female infertility. Ovarian failure can cause the follicle to fail to develop, resulting in no ovulation. Granulosa cells (GCs) are associated with oocytes during the development and ovulation of follicles, helping to complete physiological processes such as sperm viability and prokaryotic formation. As the main somatic cells in follicles, ovarian granulosa cells play an important role in follicle growth and development, atresia, oocyte maturation, and ovulation [27, 35]. The regulation mechanism of hormone synthesis and apoptosis of ovarian granulosa cells is complex and affected by many factors. Hormone synthesis and apoptosis of ovarian granulosa cells are closely related to the regulation of ovarian function.

Principal component analysis revealed that HR was the main active ingredient present in *C. sinica*. HR is a flavonoid compound [36], also known as quercetin-3-O-beta-D-galactoside [37], and it can improve the endocrine function of the ovary [38]. A previous study has reported that HR promotes the proliferation and secretion of estrogen and progesterone in rat ovarian granulosa cells, thereby improving ovarian endocrine function [39]. However, the underlying molecular mechanisms need to be comprehensively elucidated. HR reportedly exhibits several pharmacological activities mediated via different mechanisms. Chao et al. have shown that HR affords a protective effect against cisplatin-induced acute kidney injury by inactivating nuclear factor kappa B and activating nuclear factor E2-related factor-2 signaling [36]. Cao et al. have reported that HR suppresses epilepsy-induced neuronal damage by inhibiting PI3K/AKT and MAPK pathway-mediated oxidative stress and autophagy [40]. Fu et al. have demonstrated the anticancer actions of HR in non-small cell lung cancer by inducing apoptosis and autophagy via inhibition of the AKT/mTOR/p70S6K signaling pathway [41]. Zeng et al. have shown that HR protects against amyloid *β* protein-induced neurotoxicity by regulating the PI3K/AKT-mediated mitochondrial apoptotic pathway [42]. TSC1 is a crucial upstream negative regulator of mTORC1. TSC1/2 plays an essential role in the homeostasis and differentiation of immune cells through the negative regulation of the mTOR signaling pathway [30, 31]. In addition, a previous study has reported that TSC1 is involved in folliculogenesis by adjusting apoptosis and proliferation of granulosa cells [43].

Based on previous studies on PI3K/PTEN/AKT and TSC/mTORC1 signaling pathways, in the present study, our findings indicated the topological importance of AKT1 in interaction targets of POI and HR through analysis of the protein-PPI network. KEGG pathway analysis showed that HR might regulate POI by modulating the mTOR signaling pathway. In addition, the KEGG graph for the mTOR signaling [27] pathway revealed that AKT phosphorylation inhibits the activation of TSC1/2, and, in turn, TSC1/2 activation inhibits mTORC1 expression. The *in vitro* experiment revealed the protective effect of HR against TP-induced injury in KGN cells. The levels of progesterone and estrogen increased, and the antiapoptotic effect of progesterone in ovarian cells was restored. Estrogen and progesterone are necessary in the reproductive process, and their production is closely related. Estrogen is mainly produced in granulosa cells during follicular development and produces negative feedback on luteinizing hormone [44]. Progesterone is synthesized and secreted by follicles and corpus luteum components of mammalian ovary, which can affect the function of granulosa cells during follicular development before ovulation [39]. TSC1 is an important upstream negative regulator of mTORC1. TSC1/2 plays an important role in the homeostasis and differentiation of immune cells by negatively regulating mTOR signaling pathway [30, 31]. TSC1 participates in folliculogenesis by regulating granulosa cell apoptosis and proliferation. Furthermore, HR increased the phosphorylation of AKT and mTORC1 and decreased TSC1 expression in TP-induced KGN cells, consistent with the KEGG pathway analysis.

Therefore, this study predicted and verified that HR is involved in regulating the AKT/TSC1/mTORC1 signaling pathway in TP-injured KGN cells. Several studies have revealed the regulatory actions of PI3K/AKT and TSC/mTOR signaling pathways in ovarian function, including differentiation and proliferation of granulosa cells, activation and survival of primordial follicles, and meiotic maturation of oocytes [41]. It is necessary to promote the recovery of granulosa cell activity during POI process and contribute to follicle development, which is extremely necessary for clinical treatment of ovarian insufficiency.

## 5. Conclusion

In conclusion, our findings provide evidence that HR alleviates TP-induced granulosa cell injury by regulating AKT/TSC1/mTORC1 signaling. The limitation of this work is that the involvement of the AKT/TSC1/mTORC1 pathway was not confirmed by modulating its activity through transfection or other methods. Collectively, this work further discussed the promotion and mechanism of *C. sinica* compound active ingredient HR on the proliferation and secretion of damaged granulosa cells, so as to provide a theoretical and experimental basis for the clinical treatment of POI with *C. sinica* compound and its active ingredients.

## Figures and Tables

**Table 1:** The main active constituents of Plantagenet compound detection using UHPLC-QTOF-MS.

Compound name	Molecular formula	CAS number	*m*/*z*
Specnuezhenide	C_31_H_42_O_17_	39011-92-2	685.2334
Oleamide	C_18_H_35_NO	301-02-0	282.2789
**Hyperin**	**C**_**21**_ **H**_**20**_ **O**_**12**_	**482-36-0**	**463.0874**
4-Caffeoylquinic acid	C_16_H_18_O_9_	905-99-7	353.0872
Eclalbasaponin I	C_42_H_68_O_14_	158511-59-2	841.458
5-Caffeoylquinic acid	C_16_H_18_O_9_	906-33-2	353.087
3,4-Dicaffeoylquinic acid	C_25_H_24_O_12_	14534-61-3	515.1183
Astragalin	C_21_H_20_O_11_	480-10-4	447.0924
Melezitose	C_18_H_32_O_16_	10030-67-8	527.1566
3,5-Dicaffeoylquinic acid	C_25_H_24_O_12_	2450-53-5	515.1182
Lanosterol	C_30_H_52_O	79-62-9	427.3924
Luteolin 7-glucoside	C_21_H_20_O_11_	5373-11-5	447.0924
Acteoside	C_29_H_36_O_15_	61276-17-3 (22323-52-0)	623.1975
Kaempferol	C_15_H_10_O_6_	520-18-3	287.0553
Luteolin	C_15_H_10_O_6_	491-70-3	285.0404
Beta-amyrin	C_30_H_50_O	559-70-6	426.72
Datiscetin	C_15_H_10_O_6_	480-15-9	287.0549
Stevioside	C_20_H_30_O_3_	471-80-7	318.45
Myristoleic acid	C_14_H_26_O_2_	544-64-9	226.35
Rebaudioside A	C_44_H_70_O_23_	58543-16-1	965.4233
Tiliroside	C_30_H_26_O_13_	20316-62-5	593.1294
Ursolic acid	C_30_H_48_O_3_	77-52-1	456.71

## Data Availability

The data used to support the findings of this study are included within the article.

## Supplementary Materials

Supplementary Materials Supplementary 1: the predicted target proteins of HR. Supplementary 2: the predicted targets of POI. Supplementary 3: the interaction targets of HR and POI. Supplementary 4: the degree value, betweenness centrality, and closeness centrality of the interaction targets of HR and POI analyzed using PPI. Supplementary 5: the details of predicted KEGG pathways of interaction targets of HR and POI.
