# Supplementary material for: Hyperin Alleviates Triptolide-Induced Ovarian Granulosa Cell Injury by Regulating AKT/TSC1/mTORC1 Signaling
Source: Evid Based Complement Alternat Med. 2021 Oct 18;2021:9399261. doi: 10.1155/2021/9399261 (PMC8545507; doi:10.1155/2021/9399261)
Supplement: Supplementary Materials — Supplementary 1: the predicted target proteins of HR. Supplementary 2: the predicted targets of POI. Supplementary 3: the interaction targets of HR and POI. Supplementary 4: the degree value, betweenness centrality, and closeness centrality of the interaction targets of HR and POI analyzed using PPI. Supplementary 5: the details of predicted KEGG pathways of interaction targets of HR and POI. [file 9399261.f1.zip › 9399261.f1/Supplementary 1-Targets of Hyperin.pdf]

NOX4
ADRA2C
AKR1B1
CA2
CA7
CA12
CA4
ACHE
NQO2
RPS6KA3
NMUR2
ADRA2A
PTGS2
CD38
PDE5A
TNF
IL2
ADORA1
XDH
ALOX5
SLC29A1
TERT
ADORA3
PLG
KCNA3
ABCG2
SRC
APP
CYP1B1
MCL1
ALDH2
ITGAV
F10
KISS1R
CA1
CA9
CA13
ITGA2B
ITGB1
HSP90AB1
ADORA2A
CHEK2
CHEK1
PRKCG
PRKCD
PRKCA
PRKCB
PRKCE

PRKCH
DNM2
MAPT
KDM4E
GPR35
AVPR2
TOP2A
MAOA
IGF1R
FLT3
CYP19A1
INSR
EGFR
PIM1
AURKB
DRD4
GLO1
MYLK
MPO
PIK3R1
DAPK1
PYGL
SYK
GSK3B
PTK2
HSD17B2
KDR
MMP13
MMP3
CA3
ALOX15
PLK1
CA6
CDK1
MMP9
PIK3CG
MMP2
PKN1
CA14
CSNK2A1
ALOX12
MET
NEK2
CXCR1
CAMK2B
ALK
AKT1
NEK6

PLA2G1B
CA5A
AXL
RNF8
HBP1
SEC13
CDK5R1
PPARA
UAP1
HHEX
ERCC4
BCKDHA
CCND1
HIBCH
SPON1
STARD5
PTPN4
UBL3
NCBP1
VAV3
CNTN2
SRSF1
TAB1
TRAF4
NSMCE2
POT1
RAB31
HCK
ZZZ3
RBP7
FLNB
APBB1
GBP1
DNMT1
USP19
B2M
ZBTB43
GFPT1
notch1
ppp1cb
UBE2I
AKR1C4
ITPKC
PDK2
dok2
SMG7
VEGFA
ZBTB21

CDC14B
GAD2
EPN1
RARB
IDE
CCNE1
CSDE1
SRM
SHFM1
RAC1
ZEB2
ECE2
SAMHD1
HFE
RHOQ
ITPK1
SENP7
ACAD8
GGH
RAD23B
TRIM21
DPP4
TOP2
PRSS1
NOS2
PTGS1
NCOA2
ITGB3
ITGB6
ITGA5
