# Supplementary material for: Hyperin Alleviates Triptolide-Induced Ovarian Granulosa Cell Injury by Regulating AKT/TSC1/mTORC1 Signaling
Source: Evid Based Complement Alternat Med. 2021 Oct 18;2021:9399261. doi: 10.1155/2021/9399261 (PMC8545507; doi:10.1155/2021/9399261)
Supplement: Supplementary Materials — Supplementary 1: the predicted target proteins of HR. Supplementary 2: the predicted targets of POI. Supplementary 3: the interaction targets of HR and POI. Supplementary 4: the degree value, betweenness centrality, and closeness centrality of the interaction targets of HR and POI analyzed using PPI. Supplementary 5: the details of predicted KEGG pathways of interaction targets of HR and POI. [file 9399261.f1.zip › 9399261.f1/Supplementary 2-POI prediction targets.pdf]

Gene Symbol	Description
BRCA2	BRCA2 DNA Repair Associated
BRCA1	BRCA1 DNA Repair Associated
NR5A1	Nuclear Receptor Subfamily 5 Group A Member 1
POF1B	POF1B Actin Binding Protein
TP53	Tumor Protein P53
BMP15	Bone Morphogenetic Protein 15
FOXL2	Forkhead Box L2
FSHR	Follicle Stimulating Hormone Receptor
NOBOX	NOBOX Oogenesis Homeobox
INS	Insulin
PTEN	Phosphatase And Tensin Homolog
FMR1	FMRP Translational Regulator 1
NBN	Nibrin
STAG3	Stromal Antigen 3
POMC	Proopiomelanocortin
FIGLA	Folliculogenesis Specific BHLH Transcription Factor
CYP11A1	Cytochrome P450 Family 11 Subfamily A Member 1
AKT1	AKT Serine/Threonine Kinase 1
ERCC6	ERCC Excision Repair 6, Chromatin Remodeling Factor
IL6	Interleukin 6
NR0B1	Nuclear Receptor Subfamily 0 Group B Member 1
ESR1	Estrogen Receptor 1
TP63	Tumor Protein P63
LMNA	Lamin A/C
GDF9	Growth Differentiation Factor 9
CYP19A1	Cytochrome P450 Family 19 Subfamily A Member 1
WT1	WT1 Transcription Factor
CTNNB1	Catenin Beta 1
CYP17A1	Cytochrome P450 Family 17 Subfamily A Member 1
HFM1	Helicase For Meiosis 1
MCM8	Minichromosome Maintenance 8 Homologous Recombination Repair Factor
ESR2	Estrogen Receptor 2
ALB	Albumin
AMH	Anti-Mullerian Hormone
DIAPH2	Diaphanous Related Formin 2
STAR	Steroidogenic Acute Regulatory Protein
GALT	Galactose-1-Phosphate Uridylyltransferase
EIF2B2	Eukaryotic Translation Initiation Factor 2B Subunit Beta
FGFR2	Fibroblast Growth Factor Receptor 2
ATM	ATM Serine/Threonine Kinase
TERT	Telomerase Reverse Transcriptase
AMHR2	Anti-Mullerian Hormone Receptor Type 2
IL10	Interleukin 10
FGFR1	Fibroblast Growth Factor Receptor 1
MCM9	Minichromosome Maintenance 9 Homologous Recombination Repair Factor
LHCGR	Luteinizing Hormone/Choriogonadotropin Receptor
POLG	DNA Polymerase Gamma, Catalytic Subunit
PSMC3IP	PSMC3 Interacting Protein
KRAS	KRAS Proto-Oncogene, GTPase
SYCE1	Synaptonemal Complex Central Element Protein 1
MIR146A	MicroRNA 146a
MSH5	MutS Homolog 5
TGFB1	Transforming Growth Factor Beta 1

PAEP	Progestagen Associated Endometrial Protein
LARS2	Leucyl-TRNA Synthetase 2, Mitochondrial
AIRE	Autoimmune Regulator
PIK3CA	Phosphatidylinositol-4,5-Bisphosphate 3-Kinase Catalytic Subunit Alp
EIF2B4	Eukaryotic Translation Initiation Factor 2B Subunit Delta
MRE11	MRE11 Homolog, Double Strand Break Repair Nuclease
MMP2	Matrix Metallopeptidase 2
ERBB2	Erb-B2 Receptor Tyrosine Kinase 2
HSD17B4	Hydroxysteroid 17-Beta Dehydrogenase 4
SOX9	SRY-Box Transcription Factor 9
FAS	Fas Cell Surface Death Receptor
HNF1B	HNF1 Homeobox B
TGFBR2	Transforming Growth Factor Beta Receptor 2
IGF2	Insulin Like Growth Factor 2
TLR4	Toll Like Receptor 4
BRAF	B-Raf Proto-Oncogene, Serine/Threonine Kinase
FANCM	FA Complementation Group M
KITLG	KIT Ligand
CYP21A2	Cytochrome P450 Family 21 Subfamily A Member 2
TWIST1	Twist Family BHLH Transcription Factor 1
IGF1	Insulin Like Growth Factor 1
APOE	Apolipoprotein E
RAD51C	RAD51 Paralog C
EGF	Epidermal Growth Factor
CCR6	C-C Motif Chemokine Receptor 6
CDKN2A	Cyclin Dependent Kinase Inhibitor 2A
MTOR	Mechanistic Target Of Rapamycin Kinase
GATA4	GATA Binding Protein 4
MYC	MYC Proto-Oncogene, BHLH Transcription Factor
RAD51	RAD51 Recombinase
INHBA	Inhibin Subunit Beta A
REN	Renin
FLT1	Fms Related Receptor Tyrosine Kinase 1
WNT4	Wnt Family Member 4
RAD50	RAD50 Double Strand Break Repair Protein
FSHB	Follicle Stimulating Hormone Subunit Beta
EGFR	Epidermal Growth Factor Receptor
TGFBR1	Transforming Growth Factor Beta Receptor 1
IFNG	Interferon Gamma
FGFR3	Fibroblast Growth Factor Receptor 3
MLH1	MutL Homolog 1
PREPL	Prolyl Endopeptidase Like
HRAS	HRas Proto-Oncogene, GTPase
PDGFRB	Platelet Derived Growth Factor Receptor Beta
NOTCH1	Notch Receptor 1
FASLG	Fas Ligand
MMP1	Matrix Metallopeptidase 1
MDM2	MDM2 Proto-Oncogene
WRN	WRN RecQ Like Helicase
HSD3B2	Hydroxy-Delta-5-Steroid Dehydrogenase, 3 Beta- And Steroid Delta-Iso
KISS1R	KISS1 Receptor
SOHLH1	Spermatogenesis And Oogenesis Specific Basic Helix-Loop-Helix 1
BLM	BLM RecQ Like Helicase
FBN1	Fibrillin 1

MRPS22	Mitochondrial Ribosomal Protein S22
CLPP	Caseinolytic Mitochondrial Matrix Peptidase Proteolytic Subunit
NF1	Neurofibromin 1
NUP107	Nucleoporin 107
SPP1	Secreted Phosphoprotein 1
TNF	Tumor Necrosis Factor
SGO2	Shugoshin 2
SMAD3	SMAD Family Member 3
GNRH1	Gonadotropin Releasing Hormone 1
FOS	Fos Proto-Oncogene, AP-1 Transcription Factor Subunit
ERCC1	ERCC Excision Repair 1, Endonuclease Non-Catalytic Subunit
FN1	Fibronectin 1
VEGFA	Vascular Endothelial Growth Factor A
SMAD4	SMAD Family Member 4
TGFB3	Transforming Growth Factor Beta 3
PGR	Progesterone Receptor
AFF2	AF4/FMR2 Family Member 2
BMP4	Bone Morphogenetic Protein 4
PPARG	Peroxisome Proliferator Activated Receptor Gamma
BMP2	Bone Morphogenetic Protein 2
MIR144	MicroRNA 144
CHEK2	Checkpoint Kinase 2
LOX	Lysyl Oxidase
MAPK1	Mitogen-Activated Protein Kinase 1
MIR21	MicroRNA 21
KIT	KIT Proto-Oncogene, Receptor Tyrosine Kinase
CAV1	Caveolin 1
MMP14	Matrix Metallopeptidase 14
H19	H19 Imprinted Maternally Expressed Transcript
IGFBP3	Insulin Like Growth Factor Binding Protein 3
TGFB2	Transforming Growth Factor Beta 2
CASP8	Caspase 8
GJA1	Gap Junction Protein Alpha 1
STK11	Serine/Threonine Kinase 11
CDH1	Cadherin 1
INHA	Inhibin Subunit Alpha
STAT1	Signal Transducer And Activator Of Transcription 1
BNC1	Basonuclin 1
RB1	RB Transcriptional Corepressor 1
GNAS	GNAS Complex Locus
AARS2	Alanyl-TRNA Synthetase 2, Mitochondrial
SRC	SRC Proto-Oncogene, Non-Receptor Tyrosine Kinase
PRL	Prolactin
ATR	ATR Serine/Threonine Kinase
AR	Androgen Receptor
ERCC2	ERCC Excision Repair 2, TFIIH Core Complex Helicase Subunit
CTSD	Cathepsin D
WDR19	WD Repeat Domain 19
INSR	Insulin Receptor
RAF1	Raf-1 Proto-Oncogene, Serine/Threonine Kinase
MIR17	MicroRNA 17
ELN	Elastin
SNAI2	Snail Family Transcriptional Repressor 2
JAK2	Janus Kinase 2

CXCL8	C-X-C Motif Chemokine Ligand 8
NFKB1	Nuclear Factor Kappa B Subunit 1
MIR126	MicroRNA 126
MIR155	MicroRNA 155
SHBG	Sex Hormone Binding Globulin
POR	Cytochrome P450 Oxidoreductase
PDGFRA	Platelet Derived Growth Factor Receptor Alpha
SRY	Sex Determining Region Y
ZMPSTE24	Zinc Metallopeptidase STE24
MIR483	MicroRNA 483
IRS1	Insulin Receptor Substrate 1
ITGB3	Integrin Subunit Beta 3
SQSTM1	Sequestosome 1
MAP2K1	Mitogen-Activated Protein Kinase Kinase 1
MET	MET Proto-Oncogene, Receptor Tyrosine Kinase
ENG	Endoglin
PIK3R1	Phosphoinositide-3-Kinase Regulatory Subunit 1
RAD51D	RAD51 Paralog D
BUB1B	BUB1 Mitotic Checkpoint Serine/Threonine Kinase B
FGF8	Fibroblast Growth Factor 8
APC	APC Regulator Of WNT Signaling Pathway
GLI3	GLI Family Zinc Finger 3
CDKN3	Cyclin Dependent Kinase Inhibitor 3
HLA-A	Major Histocompatibility Complex, Class I, A
MEG3	Maternally Expressed 3
IL1B	Interleukin 1 Beta
LEP	Leptin
CHEK1	Checkpoint Kinase 1
DES	Desmin
MC2R	Melanocortin 2 Receptor
CCN2	Cellular Communication Network Factor 2
AKT2	AKT Serine/Threonine Kinase 2
MIR223	MicroRNA 223
NPM1	Nucleophosmin 1
SLC2A1	Solute Carrier Family 2 Member 1
CRP	C-Reactive Protein
CDK4	Cyclin Dependent Kinase 4
CFTR	CF Transmembrane Conductance Regulator
PTCH1	Patched 1
MIR27A	MicroRNA 27a
RHOA	Ras Homolog Family Member A
MMP9	Matrix Metallopeptidase 9
MIR140	MicroRNA 140
FANCF	FA Complementation Group F
TWNK	Twinkle MtDNA Helicase
AXIN1	Axin 1
PMM2	Phosphomannomutase 2
NTRK1	Neurotrophic Receptor Tyrosine Kinase 1
MIR210	MicroRNA 210
GNRHR	Gonadotropin Releasing Hormone Receptor
FOXO3	Forkhead Box O3
CYP11B1	Cytochrome P450 Family 11 Subfamily B Member 1
RUNX2	RUNX Family Transcription Factor 2
HMGA2	High Mobility Group AT-Hook 2

TERC	Telomerase RNA Component
FST	Follistatin
PTH	Parathyroid Hormone
ATP7A	ATPase Copper Transporting Alpha
ETS1	ETS Proto-Oncogene 1, Transcription Factor
CTLA4	Cytotoxic T-Lymphocyte Associated Protein 4
RFC2	Replication Factor C Subunit 2
GHR	Growth Hormone Receptor
MIR34A	MicroRNA 34a
CGA	Glycoprotein Hormones, Alpha Polypeptide
MIR127	MicroRNA 127
FANCC	FA Complementation Group C
PALB2	Partner And Localizer Of BRCA2
POU5F1	POU Class 5 Homeobox 1
MIR22	MicroRNA 22
CD46	CD46 Molecule
MSX2	Msh Homeobox 2
FGF2	Fibroblast Growth Factor 2
FBLN5	Fibulin 5
H2AC18	H2A Clustered Histone 18
HIF1A	Hypoxia Inducible Factor 1 Subunit Alpha
CAT	Catalase
CSF3	Colony Stimulating Factor 3
MIR221	MicroRNA 221
AFP	Alpha Fetoprotein
ALPL	Alkaline Phosphatase, Biomineralization Associated
TPO	Thyroid Peroxidase
CDKN1C	Cyclin Dependent Kinase Inhibitor 1C
EIF2B5	Eukaryotic Translation Initiation Factor 2B Subunit Epsilon
MUC1	Mucin 1, Cell Surface Associated
HLA-DRB1	Major Histocompatibility Complex, Class II, DR Beta 1
RECQL4	RecQ Like Helicase 4
CEP290	Centrosomal Protein 290
MEN1	Menin 1
IL2	Interleukin 2
ACE	Angiotensin I Converting Enzyme
CCBE1	Collagen And Calcium Binding EGF Domains 1
SERPINE1	Serpin Family E Member 1
VWF	Von Willebrand Factor
SHH	Sonic Hedgehog Signaling Molecule
RYR1	Ryanodine Receptor 1
SMAD6	SMAD Family Member 6
IGF1R	Insulin Like Growth Factor 1 Receptor
SEPTIN9	Septin 9
COL2A1	Collagen Type II Alpha 1 Chain
CASP3	Caspase 3
ARID1B	AT-Rich Interaction Domain 1B
MIRLET7D	MicroRNA Let-7d
KDR	Kinase Insert Domain Receptor
TSC1	TSC Complex Subunit 1
CCND1	Cyclin D1
EDN1	Endothelin 1
EP300	E1A Binding Protein P300
ASNS	Asparagine Synthetase (Glutamine-Hydrolyzing)

KISS1	KiSS-1 Metastasis Suppressor
STAT3	Signal Transducer And Activator Of Transcription 3
LGR6	Leucine Rich Repeat Containing G Protein-Coupled Receptor 6
MFAP5	Microfibril Associated Protein 5
CDKN1A	Cyclin Dependent Kinase Inhibitor 1A
SIRT1	Sirtuin 1
TSC2	TSC Complex Subunit 2
RET	Ret Proto-Oncogene
MSH2	MutS Homolog 2
SMARCA4	SWI/SNF Related, Matrix Associated, Actin Dependent Regulator Of Chr
PMS2	PMS1 Homolog 2, Mismatch Repair System Component
NOTCH2	Notch Receptor 2
RIN2	Ras And Rab Interactor 2
TIMP1	TIMP Metallopeptidase Inhibitor 1
PARP1	Poly(ADP-Ribose) Polymerase 1
DKC1	Dyskerin Pseudouridine Synthase 1
SPINK1	Serine Peptidase Inhibitor Kazal Type 1
REC8	REC8 Meiotic Recombination Protein
LRP5	LDL Receptor Related Protein 5
BSCL2	BSCL2 Lipid Droplet Biogenesis Associated, Seipin
PAX2	Paired Box 2
IFT140	Intraflagellar Transport 140
IFT122	Intraflagellar Transport 122
ABCB1	ATP Binding Cassette Subfamily B Member 1
PTPN22	Protein Tyrosine Phosphatase Non-Receptor Type 22
ICAM1	Intercellular Adhesion Molecule 1
TG	Thyroglobulin
MIR139	MicroRNA 139
TTN	Titin
FOXP2	Forkhead Box P2
CASR	Calcium Sensing Receptor
ACTA2	Actin Alpha 2, Smooth Muscle
SOX3	SRY-Box Transcription Factor 3
VCP	Valosin Containing Protein
ABCA3	ATP Binding Cassette Subfamily A Member 3
TTC21B	Tetratricopeptide Repeat Domain 21B
BRIP1	BRCA1 Interacting Protein C-Terminal Helicase 1
PTPN11	Protein Tyrosine Phosphatase Non-Receptor Type 11
NPHP1	Nephrocystin 1
TTR	Transthyretin
NPHP4	Nephrocystin 4
IL4	Interleukin 4
GK	Glycerol Kinase
CD2AP	CD2 Associated Protein
ERAL1	Era Like 12S Mitochondrial RRNA Chaperone 1
IL1A	Interleukin 1 Alpha
IGFBP1	Insulin Like Growth Factor Binding Protein 1
TIMP2	TIMP Metallopeptidase Inhibitor 2
MIRLET7C	MicroRNA Let-7c
PTGS2	Prostaglandin-Endoperoxide Synthase 2
IHH	Indian Hedgehog Signaling Molecule
CSF2	Colony Stimulating Factor 2
KRT7	Keratin 7
SOD2	Superoxide Dismutase 2

EPPIN	Epididymal Peptidase Inhibitor
ABCD1	ATP Binding Cassette Subfamily D Member 1
NPHP3	Nephrocystin 3
OFD1	OFD1 Centriole And Centriolar Satellite Protein
NKX2-5	NK2 Homeobox 5
PGRMC1	Progesterone Receptor Membrane Component 1
PAX3	Paired Box 3
INHBB	Inhibin Subunit Beta B
SHOX	Short Stature Homeobox
FGF10	Fibroblast Growth Factor 10
GH1	Growth Hormone 1
MSH6	MutS Homolog 6
IL17A	Interleukin 17A
APOA1	Apolipoprotein A1
RETN	Resistin
EIF2B3	Eukaryotic Translation Initiation Factor 2B Subunit Gamma
EIF2B1	Eukaryotic Translation Initiation Factor 2B Subunit Alpha
DICER1	Dicer 1, Ribonuclease III
CCL2	C-C Motif Chemokine Ligand 2
FRAXA	Fragile Site, Folic Acid Type, Rare, Fra(X)(Q27.3) A (Macroorchidism
DSTYK	Dual Serine/Threonine And Tyrosine Protein Kinase
PRKCD	Protein Kinase C Delta
JUN	Jun Proto-Oncogene, AP-1 Transcription Factor Subunit
COL1A1	Collagen Type I Alpha 1 Chain
DACH2	Dachshund Family Transcription Factor 2
APOB	Apolipoprotein B
PLAU	Plasminogen Activator, Urokinase
BMP6	Bone Morphogenetic Protein 6
TNFSF11	TNF Superfamily Member 11
RCBTB1	RCC1 And BTB Domain Containing Protein 1
LIG4	DNA Ligase 4
MTHFR	Methylenetetrahydrofolate Reductase
ZP3	Zona Pellucida Glycoprotein 3
CFAP47	Cilia And Flagella Associated Protein 47
EPO	Erythropoietin
VDR	Vitamin D Receptor
HP	Haptoglobin
ANGPT2	Angiopoietin 2
ACTN4	Actinin Alpha 4
INVS	Inversin
HLA-B	Major Histocompatibility Complex, Class I, B
MSH4	MutS Homolog 4
POLR3A	RNA Polymerase III Subunit A
RELA	RELA Proto-Oncogene, NF-KB Subunit
ALPP	Alkaline Phosphatase, Placental
NANOS3	Nanos C2HC-Type Zinc Finger 3
CDH23	Cadherin Related 23
SOHLH2	Spermatogenesis And Oogenesis Specific Basic Helix-Loop-Helix 2
BDNF	Brain Derived Neurotrophic Factor
BARD1	BRCA1 Associated RING Domain 1
MUC16	Mucin 16, Cell Surface Associated
FLNA	Filamin A
IGF2R	Insulin Like Growth Factor 2 Receptor
MME	Membrane Metalloendopeptidase

TWIST2	Twist Family BHLH Transcription Factor 2
SERPINA3	Serpin Family A Member 3
DAZL	Deleted In Azoospermia Like
BGLAP	Bone Gamma-Carboxyglutamate Protein
CYP11B2	Cytochrome P450 Family 11 Subfamily B Member 2
SMC1A	Structural Maintenance Of Chromosomes 1A
KDM6A	Lysine Demethylase 6A
IGFBP2	Insulin Like Growth Factor Binding Protein 2
ADIPOQ	Adiponectin, C1Q And Collagen Domain Containing
MAP3K1	Mitogen-Activated Protein Kinase Kinase Kinase 1
COL1A2	Collagen Type I Alpha 2 Chain
ANXA5	Annexin A5
ZIC3	Zic Family Member 3
MIR145	MicroRNA 145
LHX8	LIM Homeobox 8
TRAF3IP1	TRAF3 Interacting Protein 1
PRKD1	Protein Kinase D1
F2	Coagulation Factor II, Thrombin
USP8	Ubiquitin Specific Peptidase 8
HELLS	Helicase, Lymphoid Specific
TNNT2	Troponin T2, Cardiac Type
VIM	Vimentin
TNFRSF1A	TNF Receptor Superfamily Member 1A
ADAMTS19	ADAM Metallopeptidase With Thrombospondin Type 1 Motif 19
FGF1	Fibroblast Growth Factor 1
GALK1	Galactokinase 1
ERBB3	Erb-B2 Receptor Tyrosine Kinase 3
ENO1	Enolase 1
WWOX	WW Domain Containing Oxidoreductase
SUFU	SUFU Negative Regulator Of Hedgehog Signaling
FGF9	Fibroblast Growth Factor 9
TOP1	DNA Topoisomerase I
ATRX	ATRX Chromatin Remodeler
SPO11	SPO11 Initiator Of Meiotic Double Stranded Breaks
JAG1	Jagged Canonical Notch Ligand 1
ABCB4	ATP Binding Cassette Subfamily B Member 4
TEK	TEK Receptor Tyrosine Kinase
EPRS1	Glutamyl-Prolyl-TRNA Synthetase 1
GAPDH	Glyceraldehyde-3-Phosphate Dehydrogenase
HGF	Hepatocyte Growth Factor
GPR3	G Protein-Coupled Receptor 3
ALX4	ALX Homeobox 4
EFNB1	Ephrin B1
NLRP5	NLR Family Pyrin Domain Containing 5
CD19	CD19 Molecule
FANCA	FA Complementation Group A
CD44	CD44 Molecule (Indian Blood Group)
SOX10	SRY-Box Transcription Factor 10
DDX4	DEAD-Box Helicase 4
CYCS	Cytochrome C, Somatic
LDB3	LIM Domain Binding 3
NUP133	Nucleoporin 133
EIF4ENIF1	Eukaryotic Translation Initiation Factor 4E Nuclear Import Factor 1
TMEM67	Transmembrane Protein 67

MT-CYB	Mitochondrially Encoded Cytochrome B
CD40LG	CD40 Ligand
DMRT1	Doublesex And Mab-3 Related Transcription Factor 1
TMEM216	Transmembrane Protein 216
IQCB1	IQ Motif Containing B1
NFKB2	Nuclear Factor Kappa B Subunit 2
FGF7	Fibroblast Growth Factor 7
MAPK14	Mitogen-Activated Protein Kinase 14
FOXE3	Forkhead Box E3
ACTC1	Actin Alpha Cardiac Muscle 1
ZP2	Zona Pellucida Glycoprotein 2
GSTP1	Glutathione S-Transferase Pi 1
XPNPEP2	X-Prolyl Aminopeptidase 2
KIF7	Kinesin Family Member 7
PRKAR1A	Protein Kinase CAMP-Dependent Type I Regulatory Subunit Alpha
TCF12	Transcription Factor 12
BCL2L1	BCL2 Like 1
MMP7	Matrix Metallopeptidase 7
CLU	Clusterin
CEP164	Centrosomal Protein 164
LIMK1	LIM Domain Kinase 1
RECQL	RecQ Like Helicase
USP9X	Ubiquitin Specific Peptidase 9 X-Linked
SDCCAG8	SHH Signaling And Ciliogenesis Regulator SDCCAG8
CBL	Cbl Proto-Oncogene
POLR3H	RNA Polymerase III Subunit H
IFT172	Intraflagellar Transport 172
ANGPT1	Angiopoietin 1
MAPK8	Mitogen-Activated Protein Kinase 8
CCL5	C-C Motif Chemokine Ligand 5
VEGFC	Vascular Endothelial Growth Factor C
MAPK3	Mitogen-Activated Protein Kinase 3
MIR125A	MicroRNA 125a
COL3A1	Collagen Type III Alpha 1 Chain
PRKN	Parkin RBR E3 Ubiquitin Protein Ligase
SMC1B	Structural Maintenance Of Chromosomes 1B
APEX1	Apurinic/Apyrimidinic Endodeoxyribonuclease 1
CXCL12	C-X-C Motif Chemokine Ligand 12
CPEB1	Cytoplasmic Polyadenylation Element Binding Protein 1
SYNE2	Spectrin Repeat Containing Nuclear Envelope Protein 2
SYCP3	Synaptonemal Complex Protein 3
THBS1	Thrombospondin 1
IL1RAPL1	Interleukin 1 Receptor Accessory Protein Like 1
RAB23	RAB23, Member RAS Oncogene Family
CASP10	Caspase 10
H2AX	H2A.X Variant Histone
HPRT1	Hypoxanthine Phosphoribosyltransferase 1
TNFSF10	TNF Superfamily Member 10
IL6R	Interleukin 6 Receptor
LCN2	Lipocalin 2
SPIDR	Scaffold Protein Involved In DNA Repair
FANCD2	FA Complementation Group D2
MMP8	Matrix Metallopeptidase 8
CSF1	Colony Stimulating Factor 1

IFNA1	Interferon Alpha 1
MIR200A	MicroRNA 200a
RAD21L1	RAD21 Cohesin Complex Component Like 1
IL3	Interleukin 3
ICOSLG	Inducible T Cell Costimulator Ligand
RPGRIP1L	RPGRIP1 Like
DNMT1	DNA Methyltransferase 1
NOTCH3	Notch Receptor 3
DAB2	DAB Adaptor Protein 2
RPS6KB1	Ribosomal Protein S6 Kinase B1
NPHS1	NPHS1 Adhesion Molecule, Nephrin
COL5A1	Collagen Type V Alpha 1 Chain
SARS2	Seryl-TRNA Synthetase 2, Mitochondrial
ELANE	Elastase, Neutrophil Expressed
DCN	Decorin
GJC2	Gap Junction Protein Gamma 2
FOXP3	Forkhead Box P3
PECAM1	Platelet And Endothelial Cell Adhesion Molecule 1
CRH	Corticotropin Releasing Hormone
STRA8	Stimulated By Retinoic Acid 8
CYP1A1	Cytochrome P450 Family 1 Subfamily A Member 1
CXCL10	C-X-C Motif Chemokine Ligand 10
COG7	Component Of Oligomeric Golgi Complex 7
DNMT3A	DNA Methyltransferase 3 Alpha
BIRC5	Baculoviral IAP Repeat Containing 5
TYMP	Thymidine Phosphorylase
SYCP1	Synaptonemal Complex Protein 1
FLNB	Filamin B
PLAUR	Plasminogen Activator, Urokinase Receptor
HSPA4	Heat Shock Protein Family A (Hsp70) Member 4
FAM122C	Family With Sequence Similarity 122C
EFEMP2	EGF Containing Fibulin Extracellular Matrix Protein 2
MT-CO1	Mitochondrially Encoded Cytochrome C Oxidase I
CYP3A4	Cytochrome P450 Family 3 Subfamily A Member 4
ATP7B	ATPase Copper Transporting Beta
CXCR4	C-X-C Motif Chemokine Receptor 4
XPA	XPA, DNA Damage Recognition And Repair Factor
THBD	Thrombomodulin
ACAN	Aggrecan
IFT80	Intraflagellar Transport 80
TBX5	T-Box Transcription Factor 5
DMPK	DM1 Protein Kinase
GRP	Gastrin Releasing Peptide
SLPI	Secretory Leukocyte Peptidase Inhibitor
DNMT3B	DNA Methyltransferase 3 Beta
EZH2	Enhancer Of Zeste 2 Polycomb Repressive Complex 2 Subunit
SFTPB	Surfactant Protein B
XIAP	X-Linked Inhibitor Of Apoptosis
PTGS1	Prostaglandin-Endoperoxide Synthase 1
NRXN1	Neurexin 1
HDAC9	Histone Deacetylase 9
PPP3CA	Protein Phosphatase 3 Catalytic Subunit Alpha
ERCC3	ERCC Excision Repair 3, TFIIH Core Complex Helicase Subunit
PCSK1	Proprotein Convertase Subtilisin/Kexin Type 1

RASGRP1	RAS Guanyl Releasing Protein 1
HSPA5	Heat Shock Protein Family A (Hsp70) Member 5
HSPD1	Heat Shock Protein Family D (Hsp60) Member 1
HADHA	Hydroxyacyl-CoA Dehydrogenase Trifunctional Multienzyme Complex Subu
NQO1	NAD(P)H Quinone Dehydrogenase 1
HNF4A	Hepatocyte Nuclear Factor 4 Alpha
INF2	Inverted Formin 2
CDK1	Cyclin Dependent Kinase 1
MUC5AC	Mucin 5AC, Oligomeric Mucus/Gel-Forming
MIR182	MicroRNA 182
AIP	Aryl Hydrocarbon Receptor Interacting Protein
ERBB4	Erb-B2 Receptor Tyrosine Kinase 4
BMPR1A	Bone Morphogenetic Protein Receptor Type 1A
MIR143	MicroRNA 143
MIR9-1	MicroRNA 9-1
SP1	Sp1 Transcription Factor
DHFR	Dihydrofolate Reductase
CD34	CD34 Molecule
ARID1A	AT-Rich Interaction Domain 1A
MIR195	MicroRNA 195
XRCC2	X-Ray Repair Cross Complementing 2
GPC3	Glypican 3
ZIC1	Zic Family Member 1
GZMB	Granzyme B
MIR200C	MicroRNA 200c
B4GALNT1	Beta-1,4-N-Acetyl-Galactosaminyltransferase 1
CDC25A	Cell Division Cycle 25A
CFLAR	CASP8 And FADD Like Apoptosis Regulator
FREM1	FRAS1 Related Extracellular Matrix 1
LDLR	Low Density Lipoprotein Receptor
HFE	Homeostatic Iron Regulator
BSG	Basigin (Ok Blood Group)
CDH5	Cadherin 5
RAN	RAN, Member RAS Oncogene Family
SMAD2	SMAD Family Member 2
SLC27A4	Solute Carrier Family 27 Member 4
MIR141	MicroRNA 141
TNNC1	Troponin C1, Slow Skeletal And Cardiac Type
SMARCB1	SWI/SNF Related, Matrix Associated, Actin Dependent Regulator Of Chr
TMEM231	Transmembrane Protein 231
BCL2	BCL2 Apoptosis Regulator
EDNRA	Endothelin Receptor Type A
SLC34A1	Solute Carrier Family 34 Member 1
ABCC2	ATP Binding Cassette Subfamily C Member 2
OXT	Oxytocin/Neurophysin I Prepropeptide
CD40	CD40 Molecule
C3	Complement C3
NRAS	NRAS Proto-Oncogene, GTPase
SOX2	SRY-Box Transcription Factor 2
CHD7	Chromodomain Helicase DNA Binding Protein 7
PLP1	Proteolipid Protein 1
CDH2	Cadherin 2
NFKBIA	NFKB Inhibitor Alpha
DCAF17	DDB1 And CUL4 Associated Factor 17

KCNQ1OT1	KCNQ1 Opposite Strand/Antisense Transcript 1
PCNA	Proliferating Cell Nuclear Antigen
THOC6	THO Complex 6
TP53BP1	Tumor Protein P53 Binding Protein 1
RAC1	Rac Family Small GTPase 1
PGM1	Phosphoglucomutase 1
COG4	Component Of Oligomeric Golgi Complex 4
TXN	Thioredoxin
AGPAT2	1-Acylglycerol-3-Phosphate O-Acyltransferase 2
KRT18	Keratin 18
F5	Coagulation Factor V
JAK1	Janus Kinase 1
PTPA	Protein Phosphatase 2 Phosphatase Activator
MIR181A1	MicroRNA 181a-1
AKT3	AKT Serine/Threonine Kinase 3
HSP90AA1	Heat Shock Protein 90 Alpha Family Class A Member 1
NRP1	Neuropilin 1
MIR10B	MicroRNA 10b
RARB	Retinoic Acid Receptor Beta
CDK6	Cyclin Dependent Kinase 6
ABL1	ABL Proto-Oncogene 1, Non-Receptor Tyrosine Kinase
BECN1	Beclin 1
PIK3CG	Phosphatidylinositol-4,5-Bisphosphate 3-Kinase Catalytic Subunit Gam
CLDN3	Claudin 3
TFRC	Transferrin Receptor
GSN	Gelsolin
MAP2K2	Mitogen-Activated Protein Kinase Kinase 2
MIR205	MicroRNA 205
MYLK	Myosin Light Chain Kinase
PLCG1	Phospholipase C Gamma 1
TNFRSF10B	TNF Receptor Superfamily Member 10b
FOXM1	Forkhead Box M1
FOXO1	Forkhead Box O1
SCN5A	Sodium Voltage-Gated Channel Alpha Subunit 5
RAD52	RAD52 Homolog, DNA Repair Protein
MIR30A	MicroRNA 30a
MALAT1	Metastasis Associated Lung Adenocarcinoma Transcript 1
CC2D2A	Coiled-Coil And C2 Domain Containing 2A
GDF15	Growth Differentiation Factor 15
BCL2L11	BCL2 Like 11
CDKN1B	Cyclin Dependent Kinase Inhibitor 1B
PLG	Plasminogen
EHMT1	Euchromatic Histone Lysine Methyltransferase 1
F3	Coagulation Factor III, Tissue Factor
MECP2	Methyl-CpG Binding Protein 2
TPM3	Tropomyosin 3
SERPINC1	Serpin Family C Member 1
MECOM	MDS1 And EVI1 Complex Locus
MCM3AP	Minichromosome Maintenance Complex Component 3 Associated Protein
SPG7	SPG7 Matrix AAA Peptidase Subunit, Paraplegin
NBR1	NBR1 Autophagy Cargo Receptor
PAX7	Paired Box 7
PROM1	Prominin 1
MIR142	MicroRNA 142

PAPPA	Pappalysin 1
RAD54L	RAD54 Like
DLL4	Delta Like Canonical Notch Ligand 4
HBEGF	Heparin Binding EGF Like Growth Factor
ENPP1	Ectonucleotide Pyrophosphatase/Phosphodiesterase 1
CSF1R	Colony Stimulating Factor 1 Receptor
CLDN4	Claudin 4
TPM1	Tropomyosin 1
KRT14	Keratin 14
IL7	Interleukin 7
COL5A2	Collagen Type V Alpha 2 Chain
SERPINB2	Serpin Family B Member 2
RHO	Rhodopsin
EIF4EBP1	Eukaryotic Translation Initiation Factor 4E Binding Protein 1
PTTG1	PTTG1 Regulator Of Sister Chromatid Separation, Securin
SLC17A5	Solute Carrier Family 17 Member 5
EFEMP1	EGF Containing Fibulin Extracellular Matrix Protein 1
GCLC	Glutamate-Cysteine Ligase Catalytic Subunit
ALG2	ALG2 Alpha-1,3/1,6-Mannosyltransferase
ITGB1	Integrin Subunit Beta 1
IL2RA	Interleukin 2 Receptor Subunit Alpha
POLE	DNA Polymerase Epsilon, Catalytic Subunit
TBX19	T-Box Transcription Factor 19
TFAP2A	Transcription Factor AP-2 Alpha
ABCG5	ATP Binding Cassette Subfamily G Member 5
TUSC3	Tumor Suppressor Candidate 3
SST	Somatostatin
MIR150	MicroRNA 150
BAX	BCL2 Associated X, Apoptosis Regulator
ABCA1	ATP Binding Cassette Subfamily A Member 1
GLI2	GLI Family Zinc Finger 2
MIR148A	MicroRNA 148a
AGTR1	Angiotensin II Receptor Type 1
PLK1	Polo Like Kinase 1
ZFPM2	Zinc Finger Protein, FOG Family Member 2
IFT52	Intraflagellar Transport 52
CTSB	Cathepsin B
DSP	Desmoplakin
GAS1	Growth Arrest Specific 1
CASP7	Caspase 7
HMGB1	High Mobility Group Box 1
KCNJ2	Potassium Inwardly Rectifying Channel Subfamily J Member 2
CGB5	Chorionic Gonadotropin Subunit Beta 5
ATAD1	ATPase Family AAA Domain Containing 1
SLC25A4	Solute Carrier Family 25 Member 4
GADD45A	Growth Arrest And DNA Damage Inducible Alpha
WRAP53	WD Repeat Containing Antisense To TP53
PTH1R	Parathyroid Hormone 1 Receptor
RRM2B	Ribonucleotide Reductase Regulatory TP53 Inducible Subunit M2B
TNFRSF10A	TNF Receptor Superfamily Member 10a
LPL	Lipoprotein Lipase
TNNI3	Troponin I3, Cardiac Type
NDUFB11	NADH:Ubiquinone Oxidoreductase Subunit B11
PROS1	Protein S

ANPEP	Alanyl Aminopeptidase, Membrane
GAD1	Glutamate Decarboxylase 1
FASN	Fatty Acid Synthase
TCTN3	Tectonic Family Member 3
CD4	CD4 Molecule
FBXW7	F-Box And WD Repeat Domain Containing 7
GRIN2B	Glutamate Ionotropic Receptor NMDA Type Subunit 2B
PDGFRL	Platelet Derived Growth Factor Receptor Like
COL11A2	Collagen Type XI Alpha 2 Chain
NRG1	Neuregulin 1
ACTA1	Actin Alpha 1, Skeletal Muscle
GUSB	Glucuronidase Beta
MIR376C	MicroRNA 376c
EIF4E	Eukaryotic Translation Initiation Factor 4E
CST3	Cystatin C
CDKN2B	Cyclin Dependent Kinase Inhibitor 2B
MIRLET7I	MicroRNA Let-7i
HOTAIR	HOX Transcript Antisense RNA
GRN	Granulin Precursor
EZR	Ezrin
ERN1	Endoplasmic Reticulum To Nucleus Signaling 1
PALLD	Palladin, Cytoskeletal Associated Protein
FANCB	FA Complementation Group B
NKX2-1	NK2 Homeobox 1
CD247	CD247 Molecule
MED12	Mediator Complex Subunit 12
IRS2	Insulin Receptor Substrate 2
WNT5A	Wnt Family Member 5A
TTI2	TELO2 Interacting Protein 2
CALCA	Calcitonin Related Polypeptide Alpha
TNFRSF11B	TNF Receptor Superfamily Member 11b
GGT1	Gamma-Glutamyltransferase 1
MBL2	Mannose Binding Lectin 2
NANOG	Nanog Homeobox
ERCC4	ERCC Excision Repair 4, Endonuclease Catalytic Subunit
SAMD9	Sterile Alpha Motif Domain Containing 9
PLAGL1	PLAG1 Like Zinc Finger 1
DRD2	Dopamine Receptor D2
VTN	Vitronectin
FH	Fumarate Hydratase
ACP5	Acid Phosphatase 5, Tartrate Resistant
FBLN1	Fibulin 1
LCAT	Lecithin-Cholesterol Acyltransferase
MIR495	MicroRNA 495
NEK8	NIMA Related Kinase 8
ATP6V0A2	ATPase H+ Transporting V0 Subunit A2
COMT	Catechol-O-Methyltransferase
BBS10	Bardet-Biedl Syndrome 10
COL11A1	Collagen Type XI Alpha 1 Chain
TKT	Transketolase
AGT	Angiotensinogen
MSX1	Msh Homeobox 1
GDNF	Glial Cell Derived Neurotrophic Factor
PGF	Placental Growth Factor

NEAT1	Nuclear Paraspeckle Assembly Transcript 1
TSHR	Thyroid Stimulating Hormone Receptor
CXADR	CXADR Ig-Like Cell Adhesion Molecule
KCNQ1	Potassium Voltage-Gated Channel Subfamily Q Member 1
RAD21	RAD21 Cohesin Complex Component
LYVE1	Lymphatic Vessel Endothelial Hyaluronan Receptor 1
TUG1	Taurine Up-Regulated 1
GHRL	Ghrelin And Obestatin Prepropeptide
NR3C1	Nuclear Receptor Subfamily 3 Group C Member 1
CPT2	Carnitine Palmitoyltransferase 2
ALMS1	ALMS1 Centrosome And Basal Body Associated Protein
TLR2	Toll Like Receptor 2
KDM4C	Lysine Demethylase 4C
MLX	MAX Dimerization Protein MLX
NACC1	Nucleus Accumbens Associated 1
HDAC8	Histone Deacetylase 8
CCNG1	Cyclin G1
CDKN2B-AS1	CDKN2B Antisense RNA 1
CTSL	Cathepsin L
CDC42	Cell Division Cycle 42
CYP27B1	Cytochrome P450 Family 27 Subfamily B Member 1
CD24	CD24 Molecule
FSCN1	Fascin Actin-Bundling Protein 1
XIST	X Inactive Specific Transcript
PCSK9	Proprotein Convertase Subtilisin/Kexin Type 9
SLC22A18	Solute Carrier Family 22 Member 18
PGBD3	PiggyBac Transposable Element Derived 3
SLX4	SLX4 Structure-Specific Endonuclease Subunit
F7	Coagulation Factor VII
EPHB4	EPH Receptor B4
MLH3	MutL Homolog 3
NDUFAF2	NADH:Ubiquinone Oxidoreductase Complex Assembly Factor 2
RPS6KA2	Ribosomal Protein S6 Kinase A2
MIR29B1	MicroRNA 29b-1
ZNF711	Zinc Finger Protein 711
SERPINA7	Serpin Family A Member 7
CD8A	CD8a Molecule
CLCNKB	Chloride Voltage-Gated Channel Kb
RSPO1	R-Spondin 1
FLI1	Fli-1 Proto-Oncogene, ETS Transcription Factor
SMPD1	Sphingomyelin Phosphodiesterase 1
RPS19	Ribosomal Protein S19
KRT5	Keratin 5
GTF2I	General Transcription Factor IIi
ACTB	Actin Beta
TGFA	Transforming Growth Factor Alpha
CCK	Cholecystokinin
LIPC	Lipase C, Hepatic Type
MAGEA1	MAGE Family Member A1
BAG3	BAG Cochaperone 3
BAZ1B	Bromodomain Adjacent To Zinc Finger Domain 1B
SERPINA1	Serpin Family A Member 1
CR2	Complement C3d Receptor 2
MIR99A	MicroRNA 99a

GATA3	GATA Binding Protein 3
PAX8	Paired Box 8
SUCLG1	Succinate-CoA Ligase GDP/ADP-Forming Subunit Alpha
SNCA	Synuclein Alpha
HMGA1	High Mobility Group AT-Hook 1
JUP	Junction Plakoglobin
STN1	STN1 Subunit Of CST Complex
GFAP	Glial Fibrillary Acidic Protein
TH2LCRR	T Helper Type 2 Locus Control Region Associated RNA
CRYAA	Crystallin Alpha A
CFH	Complement Factor H
XRCC5	X-Ray Repair Cross Complementing 5
NOS3	Nitric Oxide Synthase 3
TMEM138	Transmembrane Protein 138
EMD	Emerin
XRCC6	X-Ray Repair Cross Complementing 6
SPRY4-IT1	SPRY4 Intronic Transcript 1
PTHLH	Parathyroid Hormone Like Hormone
SOD1	Superoxide Dismutase 1
CFI	Complement Factor I
GTF2IRD1	GTF2I Repeat Domain Containing 1
FGF23	Fibroblast Growth Factor 23
KRT20	Keratin 20
FXYD2	FXYD Domain Containing Ion Transport Regulator 2
SELE	Selectin E
HERC2	HECT And RLD Domain Containing E3 Ubiquitin Protein Ligase 2
GHRH	Growth Hormone Releasing Hormone
SNRPN	Small Nuclear Ribonucleoprotein Polypeptide N
FGFR4	Fibroblast Growth Factor Receptor 4
SPG11	SPG11 Vesicle Trafficking Associated, Spatacsin
SYNE1	Spectrin Repeat Containing Nuclear Envelope Protein 1
TF	Transferrin
CDK2	Cyclin Dependent Kinase 2
STIM1	Stromal Interaction Molecule 1
PEX1	Peroxisomal Biogenesis Factor 1
RPS6KA3	Ribosomal Protein S6 Kinase A3
PURA	Purine Rich Element Binding Protein A
SRA1	Steroid Receptor RNA Activator 1
CGB3	Chorionic Gonadotropin Subunit Beta 3
HAND2	Heart And Neural Crest Derivatives Expressed 2
POLG2	DNA Polymerase Gamma 2, Accessory Subunit
HMGCR	3-Hydroxy-3-Methylglutaryl-CoA Reductase
PEX13	Peroxisomal Biogenesis Factor 13
PLEC	Plectin
NPPB	Natriuretic Peptide B
NDRG1	N-Myc Downstream Regulated 1
CD274	CD274 Molecule
MMP3	Matrix Metallopeptidase 3
EDNRB	Endothelin Receptor Type B
CRNDE	Colorectal Neoplasia Differentially Expressed
GPR101	G Protein-Coupled Receptor 101
PRKDC	Protein Kinase, DNA-Activated, Catalytic Subunit
NEB	Nebulin
WNT10A	Wnt Family Member 10A

TMEM43	Transmembrane Protein 43
MFN2	Mitofusin 2
TERF1	Telomeric Repeat Binding Factor 1
THPO	Thrombopoietin
TBL2	Transducin Beta Like 2
NOS2	Nitric Oxide Synthase 2
HDAC1	Histone Deacetylase 1
PEX2	Peroxisomal Biogenesis Factor 2
CLIP2	CAP-Gly Domain Containing Linker Protein 2
BMPR2	Bone Morphogenetic Protein Receptor Type 2
SLC31A1	Solute Carrier Family 31 Member 1
PTK2	Protein Tyrosine Kinase 2
HNF1A	HNF1 Homeobox A
HOXA11	Homeobox A11
HSPB1	Heat Shock Protein Family B (Small) Member 1
NSD1	Nuclear Receptor Binding SET Domain Protein 1
ABCC1	ATP Binding Cassette Subfamily C Member 1
IL6ST	Interleukin 6 Signal Transducer
PROP1	PROP Paired-Like Homeobox 1
FOXE1	Forkhead Box E1
E2F1	E2F Transcription Factor 1
TNXB	Tenascin XB
GLIS2	GLIS Family Zinc Finger 2
CCNE1	Cyclin E1
PRODH	Proline Dehydrogenase 1
SUCLA2	Succinate-CoA Ligase ADP-Forming Subunit Beta
MMACHC	Metabolism Of Cobalamin Associated C
SOCS3	Suppressor Of Cytokine Signaling 3
TERF2	Telomeric Repeat Binding Factor 2
SPARC	Secreted Protein Acidic And Cysteine Rich
UMOD	Uromodulin
ITPR3	Inositol 1,4,5-Trisphosphate Receptor Type 3
GSTM1	Glutathione S-Transferase Mu 1
CEACAM5	CEA Cell Adhesion Molecule 5
MYH11	Myosin Heavy Chain 11
PRTN3	Proteinase 3
EDN3	Endothelin 3
SETD2	SET Domain Containing 2, Histone Lysine Methyltransferase
PRSS1	Serine Protease 1
IL18	Interleukin 18
SOS1	SOS Ras/Rac Guanine Nucleotide Exchange Factor 1
HSD3BP4	Hydroxy-Delta-5-Steroid Dehydrogenase, 3 Beta, Pseudogene 4
GCK	Glucokinase
CROCC	Ciliary Rootlet Coiled-Coil, Rootletin
GREM1	Gremlin 1, DAN Family BMP Antagonist
MYH6	Myosin Heavy Chain 6
MEGF8	Multiple EGF Like Domains 8
TCF7L2	Transcription Factor 7 Like 2
TMCO1	Transmembrane And Coiled-Coil Domains 1
TYMS	Thymidylate Synthetase
S100B	S100 Calcium Binding Protein B
FOLR1	Folate Receptor Alpha
NPY	Neuropeptide Y
LIG1	DNA Ligase 1

NOD2	Nucleotide Binding Oligomerization Domain Containing 2
AURKA	Aurora Kinase A
GBA	Glucosylceramidase Beta
AQP2	Aquaporin 2
FEN1	Flap Structure-Specific Endonuclease 1
MS4A1	Membrane Spanning 4-Domains A1
L1CAM	L1 Cell Adhesion Molecule
CASP9	Caspase 9
RPA1	Replication Protein A1
BSND	Barttin CLCNK Type Accessory Subunit Beta
HSD11B2	Hydroxysteroid 11-Beta Dehydrogenase 2
DNAH8	Dynein Axonemal Heavy Chain 8
U2AF1	U2 Small Nuclear RNA Auxiliary Factor 1
NOG	Noggin
NR1H2	Nuclear Receptor Subfamily 1 Group H Member 2
CCNA2	Cyclin A2
LMNB2	Lamin B2
EDAR	Ectodysplasin A Receptor
PON1	Paraoxonase 1
WFDC2	WAP Four-Disulfide Core Domain 2
TIMP3	TIMP Metallopeptidase Inhibitor 3
MT-ND1	Mitochondrially Encoded NADH:Ubiquinone Oxidoreductase Core Subunit
OSR2	Odd-Skipped Related Transciption Factor 2
AMBP	Alpha-1-Microglobulin/Bikunin Precursor
ABCB11	ATP Binding Cassette Subfamily B Member 11
UGT1A1	UDP Glucuronosyltransferase Family 1 Member A1
LIFR	LIF Receptor Subunit Alpha
MCL1	MCL1 Apoptosis Regulator, BCL2 Family Member
EWSR1	EWS RNA Binding Protein 1
RBBP8	RB Binding Protein 8, Endonuclease
PRKCA	Protein Kinase C Alpha
PLOD1	Procollagen-Lysine,2-Oxoglutarate 5-Dioxygenase 1
LGALS3	Galectin 3
MMUT	Methylmalonyl-CoA Mutase
FGF3	Fibroblast Growth Factor 3
BCR	BCR Activator Of RhoGEF And GTPase
EXO1	Exonuclease 1
ABCG2	ATP Binding Cassette Subfamily G Member 2 (Junior Blood Group)
MLXIPL	MLX Interacting Protein Like
LAMA2	Laminin Subunit Alpha 2
HSPA1A	Heat Shock Protein Family A (Hsp70) Member 1A
LEMD3	LEM Domain Containing 3
FIG4	FIG4 Phosphoinositide 5-Phosphatase
MAGT1	Magnesium Transporter 1
AAAS	Aladin WD Repeat Nucleoporin
FGF4	Fibroblast Growth Factor 4
TOP2A	DNA Topoisomerase II Alpha
ADA	Adenosine Deaminase
SNAI1	Snail Family Transcriptional Repressor 1
PRDM10	PR/SET Domain 10
HSD11B1	Hydroxysteroid 11-Beta Dehydrogenase 1
PLAT	Plasminogen Activator, Tissue Type
MIF	Macrophage Migration Inhibitory Factor
DIABLO	Diablo IAP-Binding Mitochondrial Protein

CCNB1	Cyclin B1
LTBP4	Latent Transforming Growth Factor Beta Binding Protein 4
MIR23A	MicroRNA 23a
LBR	Lamin B Receptor
MUC4	Mucin 4, Cell Surface Associated
NME1	NME/NM23 Nucleoside Diphosphate Kinase 1
CP	Ceruloplasmin
APPL1	Adaptor Protein, Phosphotyrosine Interacting With PH Domain And Leuc
CREBBP	CREB Binding Protein
YAP1	Yes1 Associated Transcriptional Regulator
TUBGCP6	Tubulin Gamma Complex Associated Protein 6
ITGB4	Integrin Subunit Beta 4
HADHB	Hydroxyacyl-CoA Dehydrogenase Trifunctional Multienzyme Complex Subu
GATA6	GATA Binding Protein 6
HLA-DQB1	Major Histocompatibility Complex, Class II, DQ Beta 1
STS	Steroid Sulfatase
NPPA	Natriuretic Peptide A
ABCC6	ATP Binding Cassette Subfamily C Member 6
DUPXQ27.3Q28	Chromosome Xq27.3-Q28 Duplication Syndrome
CPS1	Carbamoyl-Phosphate Synthase 1
GCM2	Glial Cells Missing Transcription Factor 2
C1S	Complement C1s
TH	Tyrosine Hydroxylase
SELP	Selectin P
ZEB1	Zinc Finger E-Box Binding Homeobox 1
CDON	Cell Adhesion Associated, Oncogene Regulated
DDC	Dopa Decarboxylase
RLIM	Ring Finger Protein, LIM Domain Interacting
BMPR1B	Bone Morphogenetic Protein Receptor Type 1B
VCL	Vinculin
ROCK1	Rho Associated Coiled-Coil Containing Protein Kinase 1
CLDN14	Claudin 14
SMAD7	SMAD Family Member 7
IL1RN	Interleukin 1 Receptor Antagonist
PLIN1	Perilipin 1
TPH1	Tryptophan Hydroxylase 1
BUB1	BUB1 Mitotic Checkpoint Serine/Threonine Kinase
TGM1	Transglutaminase 1
NAGS	N-Acetylglutamate Synthase
ADAMTS13	ADAM Metallopeptidase With Thrombospondin Type 1 Motif 13
TXNRD2	Thioredoxin Reductase 2
PTPRC	Protein Tyrosine Phosphatase Receptor Type C
CLDN16	Claudin 16
RASSF1	Ras Association Domain Family Member 1
GAA	Glucosidase Alpha, Acid
SKP2	S-Phase Kinase Associated Protein 2
IFI27	Interferon Alpha Inducible Protein 27
ZFHX4	Zinc Finger Homeobox 4
ABCC8	ATP Binding Cassette Subfamily C Member 8
VIP	Vasoactive Intestinal Peptide
HSPG2	Heparan Sulfate Proteoglycan 2
MITF	Melanocyte Inducing Transcription Factor
CRYAB	Crystallin Alpha B
NES	Nestin

CD28	CD28 Molecule
SERPINH1	Serpin Family H Member 1
TFF1	Trefoil Factor 1
ABCA12	ATP Binding Cassette Subfamily A Member 12
MPO	Myeloperoxidase
CXCL1	C-X-C Motif Chemokine Ligand 1
RBM28	RNA Binding Motif Protein 28
ERCC5	ERCC Excision Repair 5, Endonuclease
RAP1A	RAP1A, Member Of RAS Oncogene Family
SULF1	Sulfatase 1
CDC25C	Cell Division Cycle 25C
ESRRB	Estrogen Related Receptor Beta
APTX	Aprataxin
SMARCA2	SWI/SNF Related, Matrix Associated, Actin Dependent Regulator Of Chr
FZD4	Frizzled Class Receptor 4
TLR9	Toll Like Receptor 9
SMC3	Structural Maintenance Of Chromosomes 3
YARS2	Tyrosyl-TRNA Synthetase 2
ATP8B1	ATPase Phospholipid Transporting 8B1
VCAM1	Vascular Cell Adhesion Molecule 1
ALOX5	Arachidonate 5-Lipoxygenase
CTNNA1	Catenin Alpha 1
DLK1	Delta Like Non-Canonical Notch Ligand 1
NCOA3	Nuclear Receptor Coactivator 3
USP7	Ubiquitin Specific Peptidase 7
TGM2	Transglutaminase 2
PPARA	Peroxisome Proliferator Activated Receptor Alpha
ZEB2	Zinc Finger E-Box Binding Homeobox 2
KLK7	Kallikrein Related Peptidase 7
UQCRFS1	Ubiquinol-Cytochrome C Reductase, Rieske Iron-Sulfur Polypeptide 1
TP73	Tumor Protein P73
MIR135A1	MicroRNA 135a-1
MKS1	MKS Transition Zone Complex Subunit 1
PKHD1	PKHD1 Ciliary IPT Domain Containing Fibrocystin/Polyductin
B2M	Beta-2-Microglobulin
ZIC4	Zic Family Member 4
ANXA2	Annexin A2
KLRK1	Killer Cell Lectin Like Receptor K1
ECHS1	Enoyl-CoA Hydratase, Short Chain 1
LEPQTL1	Leptin, Serum Levels Of
RB1CC1	RB1 Inducible Coiled-Coil 1
LHB	Luteinizing Hormone Subunit Beta
IL24	Interleukin 24
CKB	Creatine Kinase B
PSAP	Prosaposin
CHAT	Choline O-Acetyltransferase
TRH	Thyrotropin Releasing Hormone
PDCD1	Programmed Cell Death 1
IKBKB	Inhibitor Of Nuclear Factor Kappa B Kinase Subunit Beta
MBP	Myelin Basic Protein
NTRK3	Neurotrophic Receptor Tyrosine Kinase 3
LEPR	Leptin Receptor
SHC1	SHC Adaptor Protein 1
CTCF	CCCTC-Binding Factor

KCNH2	Potassium Voltage-Gated Channel Subfamily H Member 2
BANF1	BAF Nuclear Assembly Factor 1
F2R	Coagulation Factor II Thrombin Receptor
CD36	CD36 Molecule
PCCB	Propionyl-CoA Carboxylase Subunit Beta
BCKDHA	Branched Chain Keto Acid Dehydrogenase E1 Subunit Alpha
NFE2L2	Nuclear Factor, Erythroid 2 Like 2
G6PD	Glucose-6-Phosphate Dehydrogenase
DDR2	Discoidin Domain Receptor Tyrosine Kinase 2
IGFBP5	Insulin Like Growth Factor Binding Protein 5
SGO1	Shugoshin 1
FANCL	FA Complementation Group L
MIR24-1	MicroRNA 24-1
USP53	Ubiquitin Specific Peptidase 53
FANCI	FA Complementation Group I
ESRRG	Estrogen Related Receptor Gamma
UBE3A	Ubiquitin Protein Ligase E3A
TNFRSF10C	TNF Receptor Superfamily Member 10c
RARA	Retinoic Acid Receptor Alpha
LTA	Lymphotoxin Alpha
PISRT1	PISRT1 LncRNA
NR3C2	Nuclear Receptor Subfamily 3 Group C Member 2
CCND3	Cyclin D3
KLK3	Kallikrein Related Peptidase 3
SOX14	SRY-Box Transcription Factor 14
BPESC1	Blepharophimosis, Epicanthus Inversus And Ptosis Candidate 1
SPATA8-AS1	SPATA8 Antisense RNA 1 (Head To Head)
PDX1	Pancreatic And Duodenal Homeobox 1
MTRR	5-Methyltetrahydrofolate-Homocysteine Methyltransferase Reductase
SELL	Selectin L
CREB1	CAMP Responsive Element Binding Protein 1
RAB27A	RAB27A, Member RAS Oncogene Family
NAMPT	Nicotinamide Phosphoribosyltransferase
NPHS2	NPHS2 Stomatin Family Member, Podocin
DLL1	Delta Like Canonical Notch Ligand 1
IFNA2	Interferon Alpha 2
NR1H4	Nuclear Receptor Subfamily 1 Group H Member 4
KLK5	Kallikrein Related Peptidase 5
PTPN3	Protein Tyrosine Phosphatase Non-Receptor Type 3
CALB2	Calbindin 2
RPS4X	Ribosomal Protein S4 X-Linked
TAGLN	Transgelin
KLK4	Kallikrein Related Peptidase 4
AREG	Amphiregulin
SELENBP1	Selenium Binding Protein 1
AKR1B1	Aldo-Keto Reductase Family 1 Member B
GAS5	Growth Arrest Specific 5
BCKDHB	Branched Chain Keto Acid Dehydrogenase E1 Subunit Beta
ALPG	Alkaline Phosphatase, Germ Cell
GNA11	G Protein Subunit Alpha 11
FANCE	FA Complementation Group E
GNAI2	G Protein Subunit Alpha I2
RECQL5	RecQ Like Helicase 5
PRF1	Perforin 1

PRKG1	Protein Kinase CGMP-Dependent 1
ELAVL1	ELAV Like RNA Binding Protein 1
FLT4	Fms Related Receptor Tyrosine Kinase 4
RAD51B	RAD51 Paralog B
CD55	CD55 Molecule (Cromer Blood Group)
PHB	Prohibitin
MT-ATP6	Mitochondrially Encoded ATP Synthase Membrane Subunit 6
POSTN	Periostin
KMT2D	Lysine Methyltransferase 2D
MGP	Matrix Gla Protein
CTAG1B	Cancer/Testis Antigen 1B
MYD88	MYD88 Innate Immune Signal Transduction Adaptor
EPHX1	Epoxide Hydrolase 1
HPSE2	Heparanase 2 (Inactive)
MIRLET7B	MicroRNA Let-7b
MMP12	Matrix Metallopeptidase 12
SDHA	Succinate Dehydrogenase Complex Flavoprotein Subunit A
TET2	Tet Methylcytosine Dioxygenase 2
PAX6	Paired Box 6
SCNN1A	Sodium Channel Epithelial 1 Subunit Alpha
PCCA	Propionyl-CoA Carboxylase Subunit Alpha
RBPJ	Recombination Signal Binding Protein For Immunoglobulin Kappa J Regi
MIR130A	MicroRNA 130a
HBG2	Hemoglobin Subunit Gamma 2
MUC5B	Mucin 5B, Oligomeric Mucus/Gel-Forming
AURKB	Aurora Kinase B
LAMP2	Lysosomal Associated Membrane Protein 2
HOXD13	Homeobox D13
IFNB1	Interferon Beta 1
F9	Coagulation Factor IX
NUP160	Nucleoporin 160
TDGF1	Teratocarcinoma-Derived Growth Factor 1
HIBCH	3-Hydroxyisobutyryl-CoA Hydrolase
E2F2	E2F Transcription Factor 2
RPE65	Retinoid Isomerohydrolase RPE65
CD69	CD69 Molecule
CNC2	Carney Complex Type 2, Multiple Neoplasia And Lentiginosis
F10	Coagulation Factor X
HARS2	Histidyl-TRNA Synthetase 2, Mitochondrial
F8	Coagulation Factor VIII
TLR5	Toll Like Receptor 5
KMT2A	Lysine Methyltransferase 2A
FANCG	FA Complementation Group G
SHOX2	Short Stature Homeobox 2
FKBP10	FKBP Prolyl Isomerase 10
PSEN1	Presenilin 1
IL7R	Interleukin 7 Receptor
DCK	Deoxycytidine Kinase
IL5	Interleukin 5
NGF	Nerve Growth Factor
GLA	Galactosidase Alpha
NFATC1	Nuclear Factor Of Activated T Cells 1
FGF13	Fibroblast Growth Factor 13
BGN	Biglycan

FGA	Fibrinogen Alpha Chain
DHCR7	7-Dehydrocholesterol Reductase
ARL13B	ADP Ribosylation Factor Like GTPase 13B
HBA2	Hemoglobin Subunit Alpha 2
ABCG8	ATP Binding Cassette Subfamily G Member 8
COL7A1	Collagen Type VII Alpha 1 Chain
NIPBL	NIPBL Cohesin Loading Factor
IFT88	Intraflagellar Transport 88
PRLR	Prolactin Receptor
BUD23	BUD23 RRNA Methyltransferase And Ribosome Maturation Factor
ITGAM	Integrin Subunit Alpha M
MIR184	MicroRNA 184
BLOC1S1	Biogenesis Of Lysosomal Organelles Complex 1 Subunit 1
TNFRSF1B	TNF Receptor Superfamily Member 1B
SIM1	SIM BHLH Transcription Factor 1
SLC6A4	Solute Carrier Family 6 Member 4
HMOX1	Heme Oxygenase 1
TCTN2	Tectonic Family Member 2
CD27	CD27 Molecule
CRHBP	Corticotropin Releasing Hormone Binding Protein
GJB2	Gap Junction Protein Beta 2
COL9A1	Collagen Type IX Alpha 1 Chain
MIR486-1	MicroRNA 486-1
RAG1	Recombination Activating 1
CLCN7	Chloride Voltage-Gated Channel 7
CYP27A1	Cytochrome P450 Family 27 Subfamily A Member 1
CYB5A	Cytochrome B5 Type A
HSPB2	Heat Shock Protein Family B (Small) Member 2
TSPY1	Testis Specific Protein Y-Linked 1
CYP1A2	Cytochrome P450 Family 1 Subfamily A Member 2
TNFRSF8	TNF Receptor Superfamily Member 8
LMX1B	LIM Homeobox Transcription Factor 1 Beta
DCLRE1C	DNA Cross-Link Repair 1C
LFNG	LFNG O-Fucosylpeptide 3-Beta-N-Acetylglucosaminyltransferase
LPA	Lipoprotein(A)
SH2D1A	SH2 Domain Containing 1A
CFL1	Cofilin 1
PXN	Paxillin
MROH7	Maestro Heat Like Repeat Family Member 7
SAA1	Serum Amyloid A1
TPM2	Tropomyosin 2
NPC1	NPC Intracellular Cholesterol Transporter 1
NTRK2	Neurotrophic Receptor Tyrosine Kinase 2
ABCB7	ATP Binding Cassette Subfamily B Member 7
CTNND1	Catenin Delta 1
NTS	Neurotensin
GATA1	GATA Binding Protein 1
GRB2	Growth Factor Receptor Bound Protein 2
MUS81	MUS81 Structure-Specific Endonuclease Subunit
ASPM	Assembly Factor For Spindle Microtubules
IL15	Interleukin 15
RHEB	Ras Homolog, MTORC1 Binding
SREBF2	Sterol Regulatory Element Binding Transcription Factor 2
ARF1	ADP Ribosylation Factor 1

ASS1	Argininosuccinate Synthase 1
UGT1A6	UDP Glucuronosyltransferase Family 1 Member A6
IDH2	Isocitrate Dehydrogenase (NADP(+)) 2
ARSH	Arylsulfatase Family Member H
MIR186	MicroRNA 186
GFRA1	GDNF Family Receptor Alpha 1
SALL1	Spalt Like Transcription Factor 1
LRP2	LDL Receptor Related Protein 2
BBS1	Bardet-Biedl Syndrome 1
GAST	Gastrin
HTR2C	5-Hydroxytryptamine Receptor 2C
CACNA1C	Calcium Voltage-Gated Channel Subunit Alpha1 C
RSF1	Remodeling And Spacing Factor 1
NDN	Necdin, MAGE Family Member
CHRND	Cholinergic Receptor Nicotinic Delta Subunit
SYP	Synaptophysin
CAMSAP2	Calmodulin Regulated Spectrin Associated Protein Family Member 2
SMN1	Survival Of Motor Neuron 1, Telomeric
SEMA3A	Semaphorin 3A
RECK	Reversion Inducing Cysteine Rich Protein With Kazal Motifs
TNFRSF11A	TNF Receptor Superfamily Member 11a
DNAH1	Dynein Axonemal Heavy Chain 1
AICDA	Activation Induced Cytidine Deaminase
RBP4	Retinol Binding Protein 4
ATF4	Activating Transcription Factor 4
ARAF	A-Raf Proto-Oncogene, Serine/Threonine Kinase
GJA5	Gap Junction Protein Alpha 5
PML	PML Nuclear Body Scaffold
TARDBP	TAR DNA Binding Protein
CCL3	C-C Motif Chemokine Ligand 3
HERC1	HECT And RLD Domain Containing E3 Ubiquitin Protein Ligase Family Me
KLLN	Killin, P53 Regulated DNA Replication Inhibitor
BBS5	Bardet-Biedl Syndrome 5
GTF2H3	General Transcription Factor IIH Subunit 3
ACTG2	Actin Gamma 2, Smooth Muscle
XDH	Xanthine Dehydrogenase
DHPS	Deoxyhypusine Synthase
EFHC2	EF-Hand Domain Containing 2
GSK3B	Glycogen Synthase Kinase 3 Beta
TNFAIP3	TNF Alpha Induced Protein 3
IL13	Interleukin 13
ADRB2	Adrenoceptor Beta 2
MIR18A	MicroRNA 18a
SLC2A4	Solute Carrier Family 2 Member 4
GAD2	Glutamate Decarboxylase 2
IL1R1	Interleukin 1 Receptor Type 1
GARS1	Glycyl-TRNA Synthetase 1
TGIF1	TGFB Induced Factor Homeobox 1
PKM	Pyruvate Kinase M1/2
PXDN	Peroxidasin
MIR494	MicroRNA 494
GK2	Glycerol Kinase 2
RMI2	RecQ Mediated Genome Instability 2
RPS17	Ribosomal Protein S17

UGT1A8	UDP Glucuronosyltransferase Family 1 Member A8
BRD4	Bromodomain Containing 4
RNASET2	Ribonuclease T2
HES7	Hes Family BHLH Transcription Factor 7
PPIA	Peptidylprolyl Isomerase A
LNPEP	Leucyl And Cystinyl Aminopeptidase
CNTNAP1	Contactin Associated Protein 1
AGER	Advanced Glycosylation End-Product Specific Receptor
TCF4	Transcription Factor 4
KRT13	Keratin 13
RAD17	RAD17 Checkpoint Clamp Loader Component
CEP57	Centrosomal Protein 57
DGUOK	Deoxyguanosine Kinase
IBSP	Integrin Binding Sialoprotein
WFS1	Wolframin ER Transmembrane Glycoprotein
ZIC2	Zic Family Member 2
NHEJ1	Non-Homologous End Joining Factor 1
FOXK2	Forkhead Box K2
COQ8B	Coenzyme Q8B
DHH	Desert Hedgehog Signaling Molecule
KPNA2	Karyopherin Subunit Alpha 2
EIF2S1	Eukaryotic Translation Initiation Factor 2 Subunit Alpha
GDAP1	Ganglioside Induced Differentiation Associated Protein 1
LARS2-AS1	LARS2 Antisense RNA 1
NODAL	Nodal Growth Differentiation Factor
PNPO	Pyridoxamine 5'-Phosphate Oxidase
CFHR2	Complement Factor H Related 2
RMI1	RecQ Mediated Genome Instability 1
MIR499A	MicroRNA 499a
B9D1	B9 Domain Containing 1
SSBP1	Single Stranded DNA Binding Protein 1
CDH11	Cadherin 11
KCND2	Potassium Voltage-Gated Channel Subfamily D Member 2
B9D2	B9 Domain Containing 2
WNT7A	Wnt Family Member 7A
CABIN1	Calcineurin Binding Protein 1
C4A	Complement C4A (Rodgers Blood Group)
SATB2	SATB Homeobox 2
MDC1	Mediator Of DNA Damage Checkpoint 1
IRS4	Insulin Receptor Substrate 4
PHLDA2	Pleckstrin Homology Like Domain Family A Member 2
RBX1	Ring-Box 1
PBX1	PBX Homeobox 1
RPS4Y1	Ribosomal Protein S4 Y-Linked 1
BBS2	Bardet-Biedl Syndrome 2
GPX3	Glutathione Peroxidase 3
OGG1	8-Oxoguanine DNA Glycosylase
MIR15A	MicroRNA 15a
MMP13	Matrix Metallopeptidase 13
MIR663AHG	MIR663A Host Gene
CALM1	Calmodulin 1
COX5A	Cytochrome C Oxidase Subunit 5A
KEAP1	Kelch Like ECH Associated Protein 1
AHI1	Abelson Helper Integration Site 1

FAT4	FAT Atypical Cadherin 4
TRAF6	TNF Receptor Associated Factor 6
SSTR2	Somatostatin Receptor 2
DGKE	Diacylglycerol Kinase Epsilon
COL6A3	Collagen Type VI Alpha 3 Chain
ACVR2A	Activin A Receptor Type 2A
SLC52A2	Solute Carrier Family 52 Member 2
GLB1	Galactosidase Beta 1
RPS29	Ribosomal Protein S29
ADM	Adrenomedullin
NEU1	Neuraminidase 1
TNFRSF6B	TNF Receptor Superfamily Member 6b
NFIX	Nuclear Factor I X
PPP1R15A	Protein Phosphatase 1 Regulatory Subunit 15A
FADD	Fas Associated Via Death Domain
AHR	Aryl Hydrocarbon Receptor
CHRNA1	Cholinergic Receptor Nicotinic Alpha 1 Subunit
ACADS	Acyl-CoA Dehydrogenase Short Chain
PSMB8	Proteasome 20S Subunit Beta 8
SLC10A2	Solute Carrier Family 10 Member 2
SIGLEC5	Sialic Acid Binding Ig Like Lectin 5
IL12RB1	Interleukin 12 Receptor Subunit Beta 1
LRBA	LPS Responsive Beige-Like Anchor Protein
FDXR	Ferredoxin Reductase
OSGEP	O-Sialoglycoprotein Endopeptidase
CDC6	Cell Division Cycle 6
MPV17	Mitochondrial Inner Membrane Protein MPV17
GTF2H5	General Transcription Factor IIH Subunit 5
TLR3	Toll Like Receptor 3
GPT	Glutamic--Pyruvic Transaminase
COL4A4	Collagen Type IV Alpha 4 Chain
EIF2S3	Eukaryotic Translation Initiation Factor 2 Subunit Gamma
ALDH3A2	Aldehyde Dehydrogenase 3 Family Member A2
CACNA1H	Calcium Voltage-Gated Channel Subunit Alpha1 H
KDM5C	Lysine Demethylase 5C
DDB2	Damage Specific DNA Binding Protein 2
SDHB	Succinate Dehydrogenase Complex Iron Sulfur Subunit B
AIFM1	Apoptosis Inducing Factor Mitochondria Associated 1
AMACR	Alpha-Methylacyl-CoA Racemase
MIR20A	MicroRNA 20a
ALPI	Alkaline Phosphatase, Intestinal
FRAS1	Fraser Extracellular Matrix Complex Subunit 1
SPINK5	Serine Peptidase Inhibitor Kazal Type 5
LUC7L2	LUC7 Like 2, Pre-MRNA Splicing Factor
PDGFD	Platelet Derived Growth Factor D
MLANA	Melan-A
CETP	Cholesteryl Ester Transfer Protein
JAK3	Janus Kinase 3
INPPL1	Inositol Polyphosphate Phosphatase Like 1
BBS4	Bardet-Biedl Syndrome 4
NUP155	Nucleoporin 155
DSPP	Dentin Sialophosphoprotein
RIPK1	Receptor Interacting Serine/Threonine Kinase 1
AP3B1	Adaptor Related Protein Complex 3 Subunit Beta 1

TRIM5	Tripartite Motif Containing 5
MCPH1	Microcephalin 1
IGFBP4	Insulin Like Growth Factor Binding Protein 4
STAG1	Stromal Antigen 1
PPP1R15B	Protein Phosphatase 1 Regulatory Subunit 15B
APRT	Adenine Phosphoribosyltransferase
CHRNB1	Cholinergic Receptor Nicotinic Beta 1 Subunit
BBS9	Bardet-Biedl Syndrome 9
SALL4	Spalt Like Transcription Factor 4
IGHE	Immunoglobulin Heavy Constant Epsilon
ENO2	Enolase 2
CAPN10	Calpain 10
PRKAG2	Protein Kinase AMP-Activated Non-Catalytic Subunit Gamma 2
PDLIM1	PDZ And LIM Domain 1
ACVR1	Activin A Receptor Type 1
SLSN3	Senior-Loken Syndrome 3
HEPACAM	Hepatic And Glial Cell Adhesion Molecule
APP	Amyloid Beta Precursor Protein
GCSH	Glycine Cleavage System Protein H
EIF2AK4	Eukaryotic Translation Initiation Factor 2 Alpha Kinase 4
LCT	Lactase
MTHFD1	Methylenetetrahydrofolate Dehydrogenase, Cyclohydrolase And Formylte
ARHGAP10	Rho GTPase Activating Protein 10
CRKL	CRK Like Proto-Oncogene, Adaptor Protein
PIK3CD	Phosphatidylinositol-4,5-Bisphosphate 3-Kinase Catalytic Subunit Del
ARX	Aristaless Related Homeobox
APOC3	Apolipoprotein C3
NR4A1	Nuclear Receptor Subfamily 4 Group A Member 1
STAT5B	Signal Transducer And Activator Of Transcription 5B
LIG3	DNA Ligase 3
LAMA4	Laminin Subunit Alpha 4
TARS1	Threonyl-TRNA Synthetase 1
CYP1B1	Cytochrome P450 Family 1 Subfamily B Member 1
ADAR	Adenosine Deaminase RNA Specific
ACADVL	Acyl-CoA Dehydrogenase Very Long Chain
CSH1	Chorionic Somatomammotropin Hormone 1
OTC	Ornithine Carbamoyltransferase
GLUL	Glutamate-Ammonia Ligase
PSEN2	Presenilin 2
EPOR	Erythropoietin Receptor
ATRIP	ATR Interacting Protein
BMI1	BMI1 Proto-Oncogene, Polycomb Ring Finger
NAA10	N-Alpha-Acetyltransferase 10, NatA Catalytic Subunit
SCT	Secretin
MT-CO3	Mitochondrially Encoded Cytochrome C Oxidase III
CPOX	Coproporphyrinogen Oxidase
LAMB2	Laminin Subunit Beta 2
MYB	MYB Proto-Oncogene, Transcription Factor
LIF	LIF Interleukin 6 Family Cytokine
TIMM8A	Translocase Of Inner Mitochondrial Membrane 8A
APOH	Apolipoprotein H
IL2RB	Interleukin 2 Receptor Subunit Beta
POLR3B	RNA Polymerase III Subunit B
CLPS	Colipase

GBGT1	Globoside Alpha-1,3-N-Acetylgalactosaminyltransferase 1 (FORS Blood [illegible]
PTX3	Pentraxin 3
FDX1	Ferredoxin 1
IMMT	Inner Membrane Mitochondrial Protein
GC	GC Vitamin D Binding Protein
ERCC6L2	ERCC Excision Repair 6 Like 2
ACOT11	Acyl-CoA Thioesterase 11
TCTN1	Tectonic Family Member 1
CEL	Carboxyl Ester Lipase
LTF	Lactotransferrin
ITGAV	Integrin Subunit Alpha V
GCG	Glucagon
DGCR2	DiGeorge Syndrome Critical Region Gene 2
GATA2	GATA Binding Protein 2
TSPAN12	Tetraspanin 12
XBP1	X-Box Binding Protein 1
COL6A1	Collagen Type VI Alpha 1 Chain
SLC5A3	Solute Carrier Family 5 Member 3
H6PD	Hexose-6-Phosphate Dehydrogenase/Glucose 1-Dehydrogenase
PROKR2	Prokineticin Receptor 2
EIF2S2	Eukaryotic Translation Initiation Factor 2 Subunit Beta
SLC26A4	Solute Carrier Family 26 Member 4
TOR1A	Torsin Family 1 Member A
MIR181A2	MicroRNA 181a-2
LGALS7	Galectin 7
LEF1	Lymphoid Enhancer Binding Factor 1
NDUFS2	NADH:Ubiquinone Oxidoreductase Core Subunit S2
GNB4	G Protein Subunit Beta 4
STAC2	SH3 And Cysteine Rich Domain 2
SCARB1	Scavenger Receptor Class B Member 1
TUBB	Tubulin Beta Class I
COG2	Component Of Oligomeric Golgi Complex 2
SYNPO2	Synaptopodin 2
TNFSF13B	TNF Superfamily Member 13b
FGF17	Fibroblast Growth Factor 17
NDUFA12	NADH:Ubiquinone Oxidoreductase Subunit A12
SERPINF1	Serpin Family F Member 1
NEK9	NIMA Related Kinase 9
KIF3A	Kinesin Family Member 3A
DPP4	Dipeptidyl Peptidase 4
LAGE3	L Antigen Family Member 3
TH2-LCR	Th2 Cytokine Locus Control Region
SSTR1	Somatostatin Receptor 1
COL4A5	Collagen Type IV Alpha 5 Chain
KRT19	Keratin 19
NDUFA9	NADH:Ubiquinone Oxidoreductase Subunit A9
CASP1	Caspase 1
MKI67	Marker Of Proliferation Ki-67
FAM124B	Family With Sequence Similarity 124 Member B
NUP85	Nucleoporin 85
SSTR3	Somatostatin Receptor 3
LCK	LCK Proto-Oncogene, Src Family Tyrosine Kinase
CST6	Cystatin E/M
KRT17	Keratin 17

ATCAY	ATCAY Kinesin Light Chain Interacting Caytaxin
MAD2L2	Mitotic Arrest Deficient 2 Like 2
CD79A	CD79a Molecule
HSD3B1	Hydroxy-Delta-5-Steroid Dehydrogenase, 3 Beta- And Steroid Delta-Iso
CCL11	C-C Motif Chemokine Ligand 11
STRADB	STE20 Related Adaptor Beta
MIR203A	MicroRNA 203a
LIPE	Lipase E, Hormone Sensitive Type
SERPINA6	Serpin Family A Member 6
CNTLN	Centlein
GLI1	GLI Family Zinc Finger 1
SRD5A1	Steroid 5 Alpha-Reductase 1
DBH	Dopamine Beta-Hydroxylase
MAPT	Microtubule Associated Protein Tau
GALE	UDP-Galactose-4-Epimerase
AKAP6	A-Kinase Anchoring Protein 6
SSTR5	Somatostatin Receptor 5
ABCA4	ATP Binding Cassette Subfamily A Member 4
ASPA	Aspartoacylase
KL	Klotho
CSH2	Chorionic Somatomammotropin Hormone 2
UNC13D	Unc-13 Homolog D
ACHE	Acetylcholinesterase (Cartwright Blood Group)
HTR2A	5-Hydroxytryptamine Receptor 2A
EIF4G1	Eukaryotic Translation Initiation Factor 4 Gamma 1
CCNH	Cyclin H
RPS20	Ribosomal Protein S20
CAVIN1	Caveolae Associated Protein 1
BAG5	BAG Cochaperone 5
GAL	Galanin And GMAP Prepropeptide
CLCN1	Chloride Voltage-Gated Channel 1
ABCC3	ATP Binding Cassette Subfamily C Member 3
COL6A2	Collagen Type VI Alpha 2 Chain
LGALS7B	Galectin 7B
UGDH	UDP-Glucose 6-Dehydrogenase
MRPL44	Mitochondrial Ribosomal Protein L44
GALM	Galactose Mutarotase
UGP2	UDP-Glucose Pyrophosphorylase 2
UAP1	UDP-N-Acetylglucosamine Pyrophosphorylase 1
GALK2	Galactokinase 2
CLC	Charcot-Leyden Crystal Galectin
TCF15	Transcription Factor 15
MST1R	Macrophage Stimulating 1 Receptor
ITGAL	Integrin Subunit Alpha L
IFT20	Intraflagellar Transport 20
ALOXE3	Arachidonate Lipoxygenase 3
MATK	Megakaryocyte-Associated Tyrosine Kinase
XPC	XPC Complex Subunit, DNA Damage Recognition And Repair Factor
MTR	5-Methyltetrahydrofolate-Homocysteine Methyltransferase
ALOX12B	Arachidonate 12-Lipoxygenase, 12R Type
PPP1CB	Protein Phosphatase 1 Catalytic Subunit Beta
CDC7	Cell Division Cycle 7
SMCR8	SMCR8-C9orf72 Complex Subunit
EIF5AL1	Eukaryotic Translation Initiation Factor 5A Like 1

SCGB1A1	Secretoglobin Family 1A Member 1
HSD17B1	Hydroxysteroid 17-Beta Dehydrogenase 1
VRK2	VRK Serine/Threonine Kinase 2
FGR	FGR Proto-Oncogene, Src Family Tyrosine Kinase
PSMB5	Proteasome 20S Subunit Beta 5
CHGA	Chromogranin A
IL12B	Interleukin 12B
INSL3	Insulin Like 3
PDGFB	Platelet Derived Growth Factor Subunit B
CTSA	Cathepsin A
C2CD3	C2 Domain Containing 3 Centriole Elongation Regulator
P2RY12	Purinergic Receptor P2Y12
SKI	SKI Proto-Oncogene
LDLRAP1	Low Density Lipoprotein Receptor Adaptor Protein 1
GLDC	Glycine Decarboxylase
HSPA8	Heat Shock Protein Family A (Hsp70) Member 8
SGCE	Sarcoglycan Epsilon
POLD1	DNA Polymerase Delta 1, Catalytic Subunit
TRPV6	Transient Receptor Potential Cation Channel Subfamily V Member 6
GSR	Glutathione-Disulfide Reductase
COL17A1	Collagen Type XVII Alpha 1 Chain
CBS	Cystathionine Beta-Synthase
EGR2	Early Growth Response 2
BMP1	Bone Morphogenetic Protein 1
SSH1	Slingshot Protein Phosphatase 1
DLG4	Discs Large MAGUK Scaffold Protein 4
GDF5	Growth Differentiation Factor 5
CCR5	C-C Motif Chemokine Receptor 5
FKBP1A	FKBP Prolyl Isomerase 1A
MYOCD	Myocardin
CDH3	Cadherin 3
HNRNPA2B1	Heterogeneous Nuclear Ribonucleoprotein A2/B1
FXN	Frataxin
APLN	Apelin
PLOD2	Procollagen-Lysine,2-Oxoglutarate 5-Dioxygenase 2
GSC	Goosecoid Homeobox
AGTR2	Angiotensin II Receptor Type 2
CLDN11	Claudin 11
ANKRD49	Ankyrin Repeat Domain 49
UBE2G1	Ubiquitin Conjugating Enzyme E2 G1
HSPA12A	Heat Shock Protein Family A (Hsp70) Member 12A
TYR	Tyrosinase
UPF1	UPF1 RNA Helicase And ATPase
MC4R	Melanocortin 4 Receptor
OSGIN2	Oxidative Stress Induced Growth Inhibitor Family Member 2
MAS1L	MAS1 Proto-Oncogene Like, G Protein-Coupled Receptor
COQ2	Coenzyme Q2, Polyprenyltransferase
CDH13	Cadherin 13
TNFSF12	TNF Superfamily Member 12
NUP153	Nucleoporin 153
SMARCD2	SWI/SNF Related, Matrix Associated, Actin Dependent Regulator Of Chr
NCAM1	Neural Cell Adhesion Molecule 1
GSTT1	Glutathione S-Transferase Theta 1
POLB	DNA Polymerase Beta

CENPI	Centromere Protein I
STAT4	Signal Transducer And Activator Of Transcription 4
BIRC2	Baculoviral IAP Repeat Containing 2
LOC110806262	Solute Carrier Family 6 Member 4 Gene Promoter
TTC30B	Tetratricopeptide Repeat Domain 30B
AKAP13	A-Kinase Anchoring Protein 13
SACS	Sacsin Molecular Chaperone
EIF5	Eukaryotic Translation Initiation Factor 5
POLR3K	RNA Polymerase III Subunit K
BCL7B	BAF Chromatin Remodeling Complex Subunit BCL7B
HLA-DQA1	Major Histocompatibility Complex, Class II, DQ Alpha 1
BBS12	Bardet-Biedl Syndrome 12
SMARCAL1	SWI/SNF Related, Matrix Associated, Actin Dependent Regulator Of Chr
CAMP	Cathelicidin Antimicrobial Peptide
MT-TF	Mitochondrially Encoded TRNA-Phe (UUU/C)
MIR196A2	MicroRNA 196a-2
ACOT7	Acyl-CoA Thioesterase 7
EIF1	Eukaryotic Translation Initiation Factor 1
UFD1	Ubiquitin Recognition Factor In ER Associated Degradation 1
TRIP11	Thyroid Hormone Receptor Interactor 11
SDC1	Syndecan 1
TSEN34	TRNA Splicing Endonuclease Subunit 34
SCYL1	SCY1 Like Pseudokinase 1
PDIA4	Protein Disulfide Isomerase Family A Member 4
KCNN4	Potassium Calcium-Activated Channel Subfamily N Member 4
WNT3A	Wnt Family Member 3A
PGK1	Phosphoglycerate Kinase 1
PCTP	Phosphatidylcholine Transfer Protein
ACOT12	Acyl-CoA Thioesterase 12
SYNGR2	Synaptogyrin 2
LRIT1	Leucine Rich Repeat, Ig-Like And Transmembrane Domains 1
SPTSSB	Serine Palmitoyltransferase Small Subunit B
KRTAP9-9	Keratin Associated Protein 9-9
CCNT2	Cyclin T2
MRPS34	Mitochondrial Ribosomal Protein S34
RCC1L	RCC1 Like
DDI1	DNA Damage Inducible 1 Homolog 1
SLC2A2	Solute Carrier Family 2 Member 2
SLC12A6	Solute Carrier Family 12 Member 6
USF1	Upstream Transcription Factor 1
VANGL1	VANGL Planar Cell Polarity Protein 1
APAF1	Apoptotic Peptidase Activating Factor 1
ARHGAP31	Rho GTPase Activating Protein 31
IL33	Interleukin 33
CYP24A1	Cytochrome P450 Family 24 Subfamily A Member 1
LOC108964933	CYP11A1 Promoter Region
CA2	Carbonic Anhydrase 2
TRAPPC10	Trafficking Protein Particle Complex 10
MIR675	MicroRNA 675
MICA	MHC Class I Polypeptide-Related Sequence A
SDHD	Succinate Dehydrogenase Complex Subunit D
AKAP1	A-Kinase Anchoring Protein 1
CD81	CD81 Molecule
UBR4	Ubiquitin Protein Ligase E3 Component N-Recognin 4

TOP3A	DNA Topoisomerase III Alpha
HTR1A	5-Hydroxytryptamine Receptor 1A
MIR10A	MicroRNA 10a
NLRP7	NLR Family Pyrin Domain Containing 7
GLUD1	Glutamate Dehydrogenase 1
MAGI2	Membrane Associated Guanylate Kinase, WW And PDZ Domain Containing 2
VEGFD	Vascular Endothelial Growth Factor D
PRDM16	PR/SET Domain 16
GHSR	Growth Hormone Secretagogue Receptor
ADAMTS4	ADAM Metallopeptidase With Thrombospondin Type 1 Motif 4
AMER1	APC Membrane Recruitment Protein 1
DACT1	Dishevelled Binding Antagonist Of Beta Catenin 1
DDB1	Damage Specific DNA Binding Protein 1
RASA1	RAS P21 Protein Activator 1
NR1I2	Nuclear Receptor Subfamily 1 Group I Member 2
FAM111A	Family With Sequence Similarity 111 Member A
NFATC2	Nuclear Factor Of Activated T Cells 2
STAG2	Stromal Antigen 2
SDHC	Succinate Dehydrogenase Complex Subunit C
CEP104	Centrosomal Protein 104
MIR423	MicroRNA 423
TCN1	Transcobalamin 1
MB	Myoglobin
SPAST	Spastin
OXTR	Oxytocin Receptor
LINC01554	Long Intergenic Non-Protein Coding RNA 1554
MT-TH	Mitochondrially Encoded TRNA-His (CAU/C)
UBB	Ubiquitin B
C9orf72	C9orf72-SMCR8 Complex Subunit
CD80	CD80 Molecule
XRCC4	X-Ray Repair Cross Complementing 4
SSTR4	Somatostatin Receptor 4
PPP1R1B	Protein Phosphatase 1 Regulatory Inhibitor Subunit 1B
GRIA1	Glutamate Ionotropic Receptor AMPA Type Subunit 1
CAMK4	Calcium/Calmodulin Dependent Protein Kinase IV
SOX8	SRY-Box Transcription Factor 8
CLTCL1	Clathrin Heavy Chain Like 1
AKT1S1	AKT1 Substrate 1
AQP4	Aquaporin 4
RCAN1	Regulator Of Calcineurin 1
EIF6	Eukaryotic Translation Initiation Factor 6
DDIT3	DNA Damage Inducible Transcript 3
MLKL	Mixed Lineage Kinase Domain Like Pseudokinase
H19-ICR	H19/IGF2 Imprinting Control Region
DYNC2H1	Dynein Cytoplasmic 2 Heavy Chain 1
DPYD	Dihydropyrimidine Dehydrogenase
CYP2D6	Cytochrome P450 Family 2 Subfamily D Member 6
GBA2	Glucosylceramidase Beta 2
PIK3C2A	Phosphatidylinositol-4-Phosphate 3-Kinase Catalytic Subunit Type 2 A
RPS6KB2	Ribosomal Protein S6 Kinase B2
RUNX1	RUNX Family Transcription Factor 1
UNG	Uracil DNA Glycosylase
CCP110	Centriolar Coiled-Coil Protein 110
CEP97	Centrosomal Protein 97

GGT2	Gamma-Glutamyltransferase 2
GGTLC3	Gamma-Glutamyltransferase Light Chain Family Member 3
TTC16	Tetratricopeptide Repeat Domain 16
YBX1	Y-Box Binding Protein 1
LAMA3	Laminin Subunit Alpha 3
FBN2	Fibrillin 2
LOXL1	Lysyl Oxidase Like 1
APOA4	Apolipoprotein A4
SHANK3	SH3 And Multiple Ankyrin Repeat Domains 3
ABHD12	Abhydrolase Domain Containing 12, Lysophospholipase
KNG1	Kininogen 1
SLC27A2	Solute Carrier Family 27 Member 2
KRT8	Keratin 8
UGCG	UDP-Glucose Ceramide Glucosyltransferase
PHF8	PHD Finger Protein 8
ADCY1	Adenylate Cyclase 1
GET1	Guided Entry Of Tail-Anchored Proteins Factor 1
VILL	Villin Like
PAM16	Presequence Translocase Associated Motor 16
ITGAX	Integrin Subunit Alpha X
LGALS1	Galectin 1
RAB5A	RAB5A, Member RAS Oncogene Family
DKK1	Dickkopf WNT Signaling Pathway Inhibitor 1
IRF4	Interferon Regulatory Factor 4
CDK7	Cyclin Dependent Kinase 7
HLCS	Holocarboxylase Synthetase
RHBDF2	Rhomboid 5 Homolog 2
RAB3GAP1	RAB3 GTPase Activating Protein Catalytic Subunit 1
ITGA4	Integrin Subunit Alpha 4
BCOR	BCL6 Corepressor
BLZF1	Basic Leucine Zipper Nuclear Factor 1
LHX1	LIM Homeobox 1
PMM1	Phosphomannomutase 1
IL1RAPL2	Interleukin 1 Receptor Accessory Protein Like 2
HHIP	Hedgehog Interacting Protein
FERD3L	Fer3 Like BHLH Transcription Factor
ITGB2	Integrin Subunit Beta 2
GNAQ	G Protein Subunit Alpha Q
IL16	Interleukin 16
ITPR1	Inositol 1,4,5-Trisphosphate Receptor Type 1
NOTCH4	Notch Receptor 4
COL10A1	Collagen Type X Alpha 1 Chain
PHOX2B	Paired Like Homeobox 2B
MAPK10	Mitogen-Activated Protein Kinase 10
RIPK3	Receptor Interacting Serine/Threonine Kinase 3
FAF1	Fas Associated Factor 1
DHCR24	24-Dehydrocholesterol Reductase
PF4	Platelet Factor 4
DYRK1A	Dual Specificity Tyrosine Phosphorylation Regulated Kinase 1A
FUS	FUS RNA Binding Protein
STRADA	STE20 Related Adaptor Alpha
MIR33A	MicroRNA 33a
S100A1	S100 Calcium Binding Protein A1
CLDN10	Claudin 10

MIR330	MicroRNA 330
SUN2	Sad1 And UNC84 Domain Containing 2
NT5C	5', 3'-Nucleotidase, Cytosolic
PC	Pyruvate Carboxylase
CEBPB	CCAAT Enhancer Binding Protein Beta
CD86	CD86 Molecule
MMD	Monocyte To Macrophage Differentiation Associated
IFNGR1	Interferon Gamma Receptor 1
AGA	Aspartylglucosaminidase
MUC2	Mucin 2, Oligomeric Mucus/Gel-Forming
MIR122	MicroRNA 122
RPGR	Retinitis Pigmentosa GTPase Regulator
GJB1	Gap Junction Protein Beta 1
FCN2	Ficolin 2
GFI1	Growth Factor Independent 1 Transcriptional Repressor
ASL	Argininosuccinate Lyase
HS6ST1	Heparan Sulfate 6-O-Sulfotransferase 1
CEACAM16	CEA Cell Adhesion Molecule 16, Tectorial Membrane Component
HJV	Hemojuvelin BMP Co-Receptor
PPP3R1	Protein Phosphatase 3 Regulatory Subunit B, Alpha
PPP3CB	Protein Phosphatase 3 Catalytic Subunit Beta
IFT57	Intraflagellar Transport 57
TRPV5	Transient Receptor Potential Cation Channel Subfamily V Member 5
PPIB	Peptidylprolyl Isomerase B
POLR2L	RNA Polymerase II, I And III Subunit L
CTTN	Cortactin
NFATC4	Nuclear Factor Of Activated T Cells 4
HPS3	HPS3 Biogenesis Of Lysosomal Organelles Complex 2 Subunit 1
ESD	Esterase D
RPL10	Ribosomal Protein L10
PPP6C	Protein Phosphatase 6 Catalytic Subunit
ALDH2	Aldehyde Dehydrogenase 2 Family Member
SLC6A3	Solute Carrier Family 6 Member 3
DCX	Doublecortin
APC2	APC Regulator Of WNT Signaling Pathway 2
HLA-G	Major Histocompatibility Complex, Class I, G
PPP3CC	Protein Phosphatase 3 Catalytic Subunit Gamma
NFATC3	Nuclear Factor Of Activated T Cells 3
AKAP5	A-Kinase Anchoring Protein 5
AKAP7	A-Kinase Anchoring Protein 7
C1orf122	Chromosome 1 Open Reading Frame 122
CXCR2	C-X-C Motif Chemokine Receptor 2
EBAG9	Estrogen Receptor Binding Site Associated Antigen 9
CALR	Calreticulin
AQP1	Aquaporin 1 (Colton Blood Group)
PI3	Peptidase Inhibitor 3
SMARCA5	SWI/SNF Related, Matrix Associated, Actin Dependent Regulator Of Chr
ALG9	ALG9 Alpha-1,2-Mannosyltransferase
AQP3	Aquaporin 3 (Gill Blood Group)
ACTG1	Actin Gamma 1
MEST	Mesoderm Specific Transcript
LAMB3	Laminin Subunit Beta 3
KRT10	Keratin 10
ACVR1B	Activin A Receptor Type 1B

CHI3L1	Chitinase 3 Like 1
CCT3	Chaperonin Containing TCP1 Subunit 3
SLC30A5	Solute Carrier Family 30 Member 5
ARFGAP3	ADP Ribosylation Factor GTPase Activating Protein 3
SLC30A7	Solute Carrier Family 30 Member 7
ATP5MD	ATP Synthase Membrane Subunit DAPIT
CARD11	Caspase Recruitment Domain Family Member 11
VCAN	Versican
ARFGAP1	ADP Ribosylation Factor GTPase Activating Protein 1
RAB3IL1	RAB3A Interacting Protein Like 1
ARFGAP2	ADP Ribosylation Factor GTPase Activating Protein 2
AHCTF1	AT-Hook Containing Transcription Factor 1
NUP43	Nucleoporin 43
TMEM120A	Transmembrane Protein 120A
DHX9	DExH-Box Helicase 9
RPGRIP1	RPGR Interacting Protein 1
VEGFB	Vascular Endothelial Growth Factor B
GPR108	G Protein-Coupled Receptor 108
LRRC56	Leucine Rich Repeat Containing 56
PYY	Peptide YY
SAT1	Spermidine/Spermine N1-Acetyltransferase 1
SLC25A13	Solute Carrier Family 25 Member 13
SPG21	SPG21 Abhydrolase Domain Containing, Maspardin
ZFP57	ZFP57 Zinc Finger Protein
CHRNA3	Cholinergic Receptor Nicotinic Alpha 3 Subunit
SPATA7	Spermatogenesis Associated 7
ABCG1	ATP Binding Cassette Subfamily G Member 1
BTK	Bruton Tyrosine Kinase
SGPL1	Sphingosine-1-Phosphate Lyase 1
OPRM1	Opioid Receptor Mu 1
CDKN2C	Cyclin Dependent Kinase Inhibitor 2C
SLC11A1	Solute Carrier Family 11 Member 1
ZFYVE26	Zinc Finger FYVE-Type Containing 26
SNCG	Synuclein Gamma
PLEK	Pleckstrin
SNURF	SNRPN Upstream Reading Frame
HPR	Haptoglobin-Related Protein
CCL4	C-C Motif Chemokine Ligand 4
CHUK	Component Of Inhibitor Of Nuclear Factor Kappa B Kinase Complex
ICMT	Isoprenylcysteine Carboxyl Methyltransferase
PRDM1	PR/SET Domain 1
CTSG	Cathepsin G
ADCY10	Adenylate Cyclase 10
ALK	ALK Receptor Tyrosine Kinase
ANAPC1	Anaphase Promoting Complex Subunit 1
HNRNPH1	Heterogeneous Nuclear Ribonucleoprotein H1
SMARCA1	SWI/SNF Related, Matrix Associated, Actin Dependent Regulator Of Chr
COL27A1	Collagen Type XXVII Alpha 1 Chain
TGFBR3	Transforming Growth Factor Beta Receptor 3
SETX	Senataxin
HOXD9	Homeobox D9
FCGR2A	Fc Fragment Of IgG Receptor IIa
MYH9	Myosin Heavy Chain 9
IL11RA	Interleukin 11 Receptor Subunit Alpha

DDX41	DEAD-Box Helicase 41
IDO1	Indoleamine 2,3-Dioxygenase 1
RHOD	Ras Homolog Family Member D
MYCN	MYCN Proto-Oncogene, BHLH Transcription Factor
POLR2A	RNA Polymerase II Subunit A
CITED2	Cbp/P300 Interacting Transactivator With Glu/Asp Rich Carboxy-Termin
BAD	BCL2 Associated Agonist Of Cell Death
ODC1	Ornithine Decarboxylase 1
MMAA	Metabolism Of Cobalamin Associated A
CBX5	Chromobox 5
CCR2	C-C Motif Chemokine Receptor 2
DIO2	Iodothyronine Deiodinase 2
HUWE1	HECT, UBA And WWE Domain Containing E3 Ubiquitin Protein Ligase 1
PSTPIP1	Proline-Serine-Threonine Phosphatase Interacting Protein 1
CCL27	C-C Motif Chemokine Ligand 27
B3GALT4	Beta-1,3-Galactosyltransferase 4
SUN1	Sad1 And UNC84 Domain Containing 1
TLR6	Toll Like Receptor 6
CXCL13	C-X-C Motif Chemokine Ligand 13
PIK3R4	Phosphoinositide-3-Kinase Regulatory Subunit 4
UCP1	Uncoupling Protein 1
NAGLU	N-Acetyl-Alpha-Glucosaminidase
BCL6	BCL6 Transcription Repressor
CTCFL	CCCTC-Binding Factor Like
NLRP2	NLR Family Pyrin Domain Containing 2
PEG3	Paternally Expressed 3
PDE11A	Phosphodiesterase 11A
SGK1	Serum/Glucocorticoid Regulated Kinase 1
H1-1	H1.1 Linker Histone, Cluster Member
MATN1	Matrilin 1
CCN1	Cellular Communication Network Factor 1
ACCS	1-Aminocyclopropane-1-Carboxylate Synthase Homolog (Inactive)
ZNRF3	Zinc And Ring Finger 3
PRICKLE4	Prickle Planar Cell Polarity Protein 4
RTP1	Receptor Transporter Protein 1
XAGE2	X Antigen Family Member 2
SLC29A1	Solute Carrier Family 29 Member 1 (Augustine Blood Group)
NCF1	Neutrophil Cytosolic Factor 1
MIR379	MicroRNA 379
HOXA13	Homeobox A13
ABHD5	Abhydrolase Domain Containing 5, Lysophosphatidic Acid Acyltransfera
PON2	Paraoxonase 2
SLC33A1	Solute Carrier Family 33 Member 1
CUL1	Cullin 1
CSN1S1	Casein Alpha S1
FBL	Fibrillarin
OTX2	Orthodenticle Homeobox 2
PAX5	Paired Box 5
WEE1	WEE1 G2 Checkpoint Kinase
CYP2C19	Cytochrome P450 Family 2 Subfamily C Member 19
PGAP1	Post-GPI Attachment To Proteins Inositol Deacylase 1
XPNPEP3	X-Prolyl Aminopeptidase 3
TSACC	TSSK6 Activating Cochaperone
NFIB	Nuclear Factor I B

KDM1A	Lysine Demethylase 1A
IVD	Isovaleryl-CoA Dehydrogenase
NF2	Neurofibromin 2
HOXB2	Homeobox B2
EIF2AK3	Eukaryotic Translation Initiation Factor 2 Alpha Kinase 3
HNRNPH2	Heterogeneous Nuclear Ribonucleoprotein H2
UPF3B	UPF3B Regulator Of Nonsense Mediated MRNA Decay
STAT5A	Signal Transducer And Activator Of Transcription 5A
NGFR	Nerve Growth Factor Receptor
TLR7	Toll Like Receptor 7
TBX4	T-Box Transcription Factor 4
CD59	CD59 Molecule (CD59 Blood Group)
LAMP1	Lysosomal Associated Membrane Protein 1
G6PC	Glucose-6-Phosphatase Catalytic Subunit
FGF6	Fibroblast Growth Factor 6
RPS6	Ribosomal Protein S6
BTD	Biotinidase
ADAMTSL1	ADAMTS Like 1
LMOD1	Leiomodin 1
PHF1	PHD Finger Protein 1
FMR1-AS1	FMR1 Antisense RNA 1
DEFB1	Defensin Beta 1
IKZF1	IKAROS Family Zinc Finger 1
ITGA2	Integrin Subunit Alpha 2
MAD2L1	Mitotic Arrest Deficient 2 Like 1
CIITA	Class II Major Histocompatibility Complex Transactivator
FNDC5	Fibronectin Type III Domain Containing 5
REEP1	Receptor Accessory Protein 1
SYNGAP1	Synaptic Ras GTPase Activating Protein 1
CSF3R	Colony Stimulating Factor 3 Receptor
PDPK1	3-Phosphoinositide Dependent Protein Kinase 1
TUBB3	Tubulin Beta 3 Class III
BAK1	BCL2 Antagonist/Killer 1
SLC4A1	Solute Carrier Family 4 Member 1 (Diego Blood Group)
KHDC3L	KH Domain Containing 3 Like, Subcortical Maternal Complex Member
VAV1	Vav Guanine Nucleotide Exchange Factor 1
FOXC2	Forkhead Box C2
NSD2	Nuclear Receptor Binding SET Domain Protein 2
DMWD	DM1 Locus, WD Repeat Containing
CYBA	Cytochrome B-245 Alpha Chain
SIRT2	Sirtuin 2
HCRT	Hypocretin Neuropeptide Precursor
RLN1	Relaxin 1
UFSP2	UFM1 Specific Peptidase 2
NPR2	Natriuretic Peptide Receptor 2
TNFRSF17	TNF Receptor Superfamily Member 17
HLA-C	Major Histocompatibility Complex, Class I, C
KCNJ5	Potassium Inwardly Rectifying Channel Subfamily J Member 5
ADD1	Adducin 1
RPS6KA1	Ribosomal Protein S6 Kinase A1
NSDHL	NAD(P) Dependent Steroid Dehydrogenase-Like
EMG1	EMG1 N1-Specific Pseudouridine Methyltransferase
TAC3	Tachykinin Precursor 3
PPARGC1A	PPARG Coactivator 1 Alpha

CR1	Complement C3b/C4b Receptor 1 (Knops Blood Group)
MMADHC	Metabolism Of Cobalamin Associated D
CCT7	Chaperonin Containing TCP1 Subunit 7
CD47	CD47 Molecule
DYNC1H1	Dynein Cytoplasmic 1 Heavy Chain 1
KHDRBS3	KH RNA Binding Domain Containing, Signal Transduction Associated 3
CUL4A	Cullin 4A
UTRN	Utrophin
KIF1C	Kinesin Family Member 1C
ARMC5	Armadillo Repeat Containing 5
NT5C1B	5'-Nucleotidase, Cytosolic IB
CD163	CD163 Molecule
ARG1	Arginase 1
GANAB	Glucosidase II Alpha Subunit
PAM	Peptidylglycine Alpha-Amidating Monooxygenase
TMLHE	Trimethyllysine Hydroxylase, Epsilon
TULP1	TUB Like Protein 1
CSTB	Cystatin B
IL18R1	Interleukin 18 Receptor 1
EHMT2	Euchromatic Histone Lysine Methyltransferase 2
PRKAA2	Protein Kinase AMP-Activated Catalytic Subunit Alpha 2
RARS1	Arginyl-TRNA Synthetase 1
ARSL	Arylsulfatase L
STAT6	Signal Transducer And Activator Of Transcription 6
FCGR3A	Fc Fragment Of IgG Receptor IIIa
DSG2	Desmoglein 2
PYGM	Glycogen Phosphorylase, Muscle Associated
CD14	CD14 Molecule
PDSS1	Decaprenyl Diphosphate Synthase Subunit 1
BCAP31	B Cell Receptor Associated Protein 31
EPHX2	Epoxide Hydrolase 2
TLR1	Toll Like Receptor 1
RBFOX2	RNA Binding Fox-1 Homolog 2
HCFC1	Host Cell Factor C1
ATP6AP1	ATPase H+ Transporting Accessory Protein 1
TFEB	Transcription Factor EB
PLA2G2A	Phospholipase A2 Group IIA
DMC1	DNA Meiotic Recombinase 1
RPS14	Ribosomal Protein S14
RUNX3	RUNX Family Transcription Factor 3
TMPO	Thymopoietin
PTPN14	Protein Tyrosine Phosphatase Non-Receptor Type 14
PLA2G7	Phospholipase A2 Group VII
MIR16-1	MicroRNA 16-1
NR1I3	Nuclear Receptor Subfamily 1 Group I Member 3
CHRNE	Cholinergic Receptor Nicotinic Epsilon Subunit
GLS	Glutaminase
SFTPD	Surfactant Protein D
KCNE1	Potassium Voltage-Gated Channel Subfamily E Regulatory Subunit 1
IL23R	Interleukin 23 Receptor
FABP3	Fatty Acid Binding Protein 3
TLR8	Toll Like Receptor 8
SYNE3	Spectrin Repeat Containing Nuclear Envelope Family Member 3
GJA4	Gap Junction Protein Alpha 4

COL18A1	Collagen Type XVIII Alpha 1 Chain
CD63	CD63 Molecule
CEBPD	CCAAT Enhancer Binding Protein Delta
ADCYAP1	Adenylate Cyclase Activating Polypeptide 1
GALNS	Galactosamine (N-Acetyl)-6-Sulfatase
MNX1	Motor Neuron And Pancreas Homeobox 1
PTRH2	Peptidyl-TRNA Hydrolase 2
FGFRL1	Fibroblast Growth Factor Receptor Like 1
F2RL1	F2R Like Trypsin Receptor 1
MVK	Mevalonate Kinase
MASP2	Mannan Binding Lectin Serine Peptidase 2
BID	BH3 Interacting Domain Death Agonist
SAG	S-Antigen Visual Arrestin
TFAM	Transcription Factor A, Mitochondrial
NSUN2	NOP2/Sun RNA Methyltransferase 2
SMAD1	SMAD Family Member 1
PLK4	Polo Like Kinase 4
MUTYH	MutY DNA Glycosylase
GHRHR	Growth Hormone Releasing Hormone Receptor
VHL	Von Hippel-Lindau Tumor Suppressor
EPHA4	EPH Receptor A4
MIR30C1	MicroRNA 30c-1
SREBF1	Sterol Regulatory Element Binding Transcription Factor 1
COL4A3	Collagen Type IV Alpha 3 Chain
TRPS1	Transcriptional Repressor GATA Binding 1
VMA21	Vacuolar ATPase Assembly Factor VMA21
TJP1	Tight Junction Protein 1
CCR7	C-C Motif Chemokine Receptor 7
SULT2A1	Sulfotransferase Family 2A Member 1
UTP4	UTP4 Small Subunit Processome Component
SPART	Spartin
ETV6	ETS Variant Transcription Factor 6
CCR3	C-C Motif Chemokine Receptor 3
LYST	Lysosomal Trafficking Regulator
SIRT3	Sirtuin 3
RAB7A	RAB7A, Member RAS Oncogene Family
ASCL1	Achaete-Scute Family BHLH Transcription Factor 1
NCOR1	Nuclear Receptor Corepressor 1
ANOS1	Anosmin 1
REEP4	Receptor Accessory Protein 4
FTO	FTO Alpha-Ketoglutarate Dependent Dioxygenase
FGF18	Fibroblast Growth Factor 18
ADAMTSL4	ADAMTS Like 4
MTNR1B	Melatonin Receptor 1B
ELOVL1	ELOVL Fatty Acid Elongase 1
EDA2R	Ectodysplasin A2 Receptor
NLRP6	NLR Family Pyrin Domain Containing 6
DNASE1	Deoxyribonuclease 1
MC1R	Melanocortin 1 Receptor
FUCA1	Alpha-L-Fucosidase 1
SEC23B	SEC23 Homolog B, COPII Coat Complex Component
CORIN	Corin, Serine Peptidase
SMO	Smoothened, Frizzled Class Receptor
ITGA3	Integrin Subunit Alpha 3

PAFAH1B1	Platelet Activating Factor Acetylhydrolase 1b Regulatory Subunit 1
FA2H	Fatty Acid 2-Hydroxylase
CYP2C9	Cytochrome P450 Family 2 Subfamily C Member 9
XPO1	Exportin 1
CISD2	CDGSH Iron Sulfur Domain 2
NOS1	Nitric Oxide Synthase 1
IL31RA	Interleukin 31 Receptor A
KLF4	Kruppel Like Factor 4
HMGCL	3-Hydroxy-3-Methylglutaryl-CoA Lyase
BCKDK	Branched Chain Keto Acid Dehydrogenase Kinase
GNPTG	N-Acetylglucosamine-1-Phosphate Transferase Subunit Gamma
IMPDH1	Inosine Monophosphate Dehydrogenase 1
COL4A1	Collagen Type IV Alpha 1 Chain
MIR374B	MicroRNA 374b
DNMT3L	DNA Methyltransferase 3 Like
LAMA1	Laminin Subunit Alpha 1
ESPL1	Extra Spindle Pole Bodies Like 1, Separase
TNNI2	Troponin I2, Fast Skeletal Type
MIR500A	MicroRNA 500a
M6PR	Mannose-6-Phosphate Receptor, Cation Dependent
MTHFS	Methenyltetrahydrofolate Synthetase
MAP3K7	Mitogen-Activated Protein Kinase Kinase Kinase 7
NAT10	N-Acetyltransferase 10
VARS1	Valyl-TRNA Synthetase 1
THADA	THADA Armadillo Repeat Containing
CHKA	Choline Kinase Alpha
LRP6	LDL Receptor Related Protein 6
LTBP1	Latent Transforming Growth Factor Beta Binding Protein 1
KARS1	Lysyl-TRNA Synthetase 1
STOX1	Storkhead Box 1
IVL	Involucrin
SLC5A5	Solute Carrier Family 5 Member 5
TAP2	Transporter 2, ATP Binding Cassette Subfamily B Member
ARNTL	Aryl Hydrocarbon Receptor Nuclear Translocator Like
CDK8	Cyclin Dependent Kinase 8
DNM1L	Dynamin 1 Like
HSPB8	Heat Shock Protein Family B (Small) Member 8
SPRY2	Sprouty RTK Signaling Antagonist 2
RAC2	Rac Family Small GTPase 2
AKAP12	A-Kinase Anchoring Protein 12
PIK3R2	Phosphoinositide-3-Kinase Regulatory Subunit 2
S100A8	S100 Calcium Binding Protein A8
NTF3	Neurotrophin 3
UBE2T	Ubiquitin Conjugating Enzyme E2 T
BMP7	Bone Morphogenetic Protein 7
UBE2D1	Ubiquitin Conjugating Enzyme E2 D1
GGCT	Gamma-Glutamylcyclotransferase
PLCE1	Phospholipase C Epsilon 1
CEACAM6	CEA Cell Adhesion Molecule 6
FCN3	Ficolin 3
MIR378A	MicroRNA 378a
LONP1	Lon Peptidase 1, Mitochondrial
WNT11	Wnt Family Member 11
JAZF1	JAZF Zinc Finger 1

IRAK1	Interleukin 1 Receptor Associated Kinase 1
PDGFA	Platelet Derived Growth Factor Subunit A
PROCR	Protein C Receptor
AXIN2	Axin 2
GGCX	Gamma-Glutamyl Carboxylase
RBL2	RB Transcriptional Corepressor Like 2
TNFRSF25	TNF Receptor Superfamily Member 25
CRHR2	Corticotropin Releasing Hormone Receptor 2
TACR3	Tachykinin Receptor 3
GMNN	Geminin DNA Replication Inhibitor
ARHGAP32	Rho GTPase Activating Protein 32
CSTA	Cystatin A
CRB1	Crumbs Cell Polarity Complex Component 1
PRPH2	Peripherin 2
ELAVL4	ELAV Like RNA Binding Protein 4
ELAVL3	ELAV Like RNA Binding Protein 3
REEP2	Receptor Accessory Protein 2
RPS3	Ribosomal Protein S3
ENPP2	Ectonucleotide Pyrophosphatase/Phosphodiesterase 2
CRX	Cone-Rod Homeobox
CHRNG	Cholinergic Receptor Nicotinic Gamma Subunit
GRPR	Gastrin Releasing Peptide Receptor
HYMAI	Hydatidiform Mole Associated And Imprinted
GMPS	Guanine Monophosphate Synthase
RDH12	Retinol Dehydrogenase 12
TOPBP1	DNA Topoisomerase II Binding Protein 1
RPTOR	Regulatory Associated Protein Of MTOR Complex 1
IGHM	Immunoglobulin Heavy Constant Mu
SIRT6	Sirtuin 6
EPAS1	Endothelial PAS Domain Protein 1
CYP2E1	Cytochrome P450 Family 2 Subfamily E Member 1
IL21	Interleukin 21
MYO5A	Myosin VA
SMC4	Structural Maintenance Of Chromosomes 4
CD52	CD52 Molecule
HK2	Hexokinase 2
SHMT1	Serine Hydroxymethyltransferase 1
FOXC1	Forkhead Box C1
LRP1	LDL Receptor Related Protein 1
ARSA	Arylsulfatase A
CFB	Complement Factor B
MEF2A	Myocyte Enhancer Factor 2A
S100A9	S100 Calcium Binding Protein A9
CD9	CD9 Molecule
PDZK1IP1	PDZK1 Interacting Protein 1
CD68	CD68 Molecule
NAT2	N-Acetyltransferase 2
PROC	Protein C, Inactivator Of Coagulation Factors Va And VIIIa
JAM3	Junctional Adhesion Molecule 3
FAM161A	FAM161 Centrosomal Protein A
DCTN1	Dynactin Subunit 1
DNA2	DNA Replication Helicase/Nuclease 2
MMAB	Metabolism Of Cobalamin Associated B
HSD17B2	Hydroxysteroid 17-Beta Dehydrogenase 2

KMT2B	Lysine Methyltransferase 2B
GATA5	GATA Binding Protein 5
TRIM27	Tripartite Motif Containing 27
YWHAQ	Tyrosine 3-Monooxygenase/Tryptophan 5-Monooxygenase Activation Prote
RBBP4	RB Binding Protein 4, Chromatin Remodeling Factor
PRKAA1	Protein Kinase AMP-Activated Catalytic Subunit Alpha 1
ERVW-1	Endogenous Retrovirus Group W Member 1, Envelope
NUP88	Nucleoporin 88
PRSS2	Serine Protease 2
MYOG	Myogenin
SUV39H1	Suppressor Of Variegation 3-9 Homolog 1
CNN1	Calponin 1
HTRA2	HtrA Serine Peptidase 2
ITGA6	Integrin Subunit Alpha 6
IRF1	Interferon Regulatory Factor 1
SLC9A3R1	SLC9A3 Regulator 1
ACP1	Acid Phosphatase 1
TACR1	Tachykinin Receptor 1
SPRY1	Sprouty RTK Signaling Antagonist 1
BIRC3	Baculoviral IAP Repeat Containing 3
YY1	YY1 Transcription Factor
ATP2A2	ATPase Sarcoplasmic/Endoplasmic Reticulum Ca2+ Transporting 2
TFAP2B	Transcription Factor AP-2 Beta
FKBP14	FKBP Prolyl Isomerase 14
TCF3	Transcription Factor 3
HBG1	Hemoglobin Subunit Gamma 1
INPP5J	Inositol Polyphosphate-5-Phosphatase J
GUCY2D	Guanylate Cyclase 2D, Retinal
TCEA1	Transcription Elongation Factor A1
USH2A	Usherin
COL9A2	Collagen Type IX Alpha 2 Chain
ABO	ABO, Alpha 1-3-N-Acetylgalactosaminyltransferase And Alpha 1-3-Galac
PDE4D	Phosphodiesterase 4D
ACAT1	Acetyl-CoA Acetyltransferase 1
CNR1	Cannabinoid Receptor 1
IL11	Interleukin 11
EDN2	Endothelin 2
CPT1A	Carnitine Palmitoyltransferase 1A
PLA2G10	Phospholipase A2 Group X
RUNX1T1	RUNX1 Partner Transcriptional Co-Repressor 1
C4B	Complement C4B (Chido Blood Group)
LCA5	Lebercilin LCA5
TSPAN32	Tetraspanin 32
ADAM17	ADAM Metallopeptidase Domain 17
KRT1	Keratin 1
MAOA	Monoamine Oxidase A
PCMT1	Protein-L-Isoaspartate (D-Aspartate) O-Methyltransferase
HNRNPA1	Heterogeneous Nuclear Ribonucleoprotein A1
HSPB6	Heat Shock Protein Family B (Small) Member 6
S100A4	S100 Calcium Binding Protein A4
PTER	Phosphotriesterase Related
SRSF6	Serine And Arginine Rich Splicing Factor 6
FCGR2B	Fc Fragment Of IgG Receptor IIb
RICTOR	RPTOR Independent Companion Of MTOR Complex 2

ACADSB	Acyl-CoA Dehydrogenase Short/Branched Chain
MCEE	Methylmalonyl-CoA Epimerase
AIPL1	Aryl Hydrocarbon Receptor Interacting Protein Like 1
MYCBP2	MYC Binding Protein 2
LMBRD1	LMBR1 Domain Containing 1
EYA3	EYA Transcriptional Coactivator And Phosphatase 3
POC1B	POC1 Centriolar Protein B
POLD4	DNA Polymerase Delta 4, Accessory Subunit
GGACT	Gamma-Glutamylamine Cyclotransferase
CCDC92	Coiled-Coil Domain Containing 92
MAK16	MAK16 Homolog
RHNO1	RAD9-HUS1-RAD1 Interacting Nuclear Orphan 1
OR51S1	Olfactory Receptor Family 51 Subfamily S Member 1
FAAP24	FA Core Complex Associated Protein 24
CD151	CD151 Molecule (Raph Blood Group)
DSC2	Desmocollin 2
SERPINF2	Serpin Family F Member 2
ACACA	Acetyl-CoA Carboxylase Alpha
RYR3	Ryanodine Receptor 3
ATXN2	Ataxin 2
HSD17B3	Hydroxysteroid 17-Beta Dehydrogenase 3
PRKCB	Protein Kinase C Beta
ALG8	ALG8 Alpha-1,3-Glucosyltransferase
FURIN	Furin, Paired Basic Amino Acid Cleaving Enzyme
PIKFYVE	Phosphoinositide Kinase, FYVE-Type Zinc Finger Containing
LOC110806263	TERT 5' Regulatory Region
NPPC	Natriuretic Peptide C
CD58	CD58 Molecule
FABP4	Fatty Acid Binding Protein 4
FABP5	Fatty Acid Binding Protein 5
MIR19B1	MicroRNA 19b-1
HDAC6	Histone Deacetylase 6
TFF3	Trefoil Factor 3
GCN1	GCN1 Activator Of EIF2AK4
TRPC3	Transient Receptor Potential Cation Channel Subfamily C Member 3
MMP10	Matrix Metallopeptidase 10
MAP2K7	Mitogen-Activated Protein Kinase Kinase 7
RPS27A	Ribosomal Protein S27a
RRM1	Ribonucleotide Reductase Catalytic Subunit M1
GAS6	Growth Arrest Specific 6
LGALS4	Galectin 4
THRB	Thyroid Hormone Receptor Beta
PSMB9	Proteasome 20S Subunit Beta 9
SLAMF1	Signaling Lymphocytic Activation Molecule Family Member 1
DDR1	Discoidin Domain Receptor Tyrosine Kinase 1
MIR154	MicroRNA 154
MICU1	Mitochondrial Calcium Uptake 1
PPIG	Peptidylprolyl Isomerase G
FRS2	Fibroblast Growth Factor Receptor Substrate 2
SOCS1	Suppressor Of Cytokine Signaling 1
MIR1-2	MicroRNA 1-2
TARS2	Threonyl-TRNA Synthetase 2, Mitochondrial
PMEL	Premelanosome Protein
TRIP13	Thyroid Hormone Receptor Interactor 13

ADIPOR1	Adiponectin Receptor 1
STMN1	Stathmin 1
PITX2	Paired Like Homeodomain 2
RAB6A	RAB6A, Member RAS Oncogene Family
CPLANE1	Ciliogenesis And Planar Polarity Effector 1
ADNP	Activity Dependent Neuroprotector Homeobox
HAX1	HCLS1 Associated Protein X-1
ADH1B	Alcohol Dehydrogenase 1B (Class I), Beta Polypeptide
FOLR3	Folate Receptor Gamma
VAMP2	Vesicle Associated Membrane Protein 2
FLT3	Fms Related Receptor Tyrosine Kinase 3
MDK	Midkine
CASP14	Caspase 14
SIRT7	Sirtuin 7
HEY2	Hes Related Family BHLH Transcription Factor With YRPW Motif 2
EFTUD2	Elongation Factor Tu GTP Binding Domain Containing 2
EPHB1	EPH Receptor B1
MIR382	MicroRNA 382
CDT1	Chromatin Licensing And DNA Replication Factor 1
HNMT	Histamine N-Methyltransferase
NPRL3	NPR3 Like, GATOR1 Complex Subunit
NUPR1	Nuclear Protein 1, Transcriptional Regulator
SORT1	Sortilin 1
TRPM7	Transient Receptor Potential Cation Channel Subfamily M Member 7
SPTLC2	Serine Palmitoyltransferase Long Chain Base Subunit 2
TCEA3	Transcription Elongation Factor A3
REG3A	Regenerating Family Member 3 Alpha
SCD	Stearoyl-CoA Desaturase
AFG3L2	AFG3 Like Matrix AAA Peptidase Subunit 2
TYRP1	Tyrosinase Related Protein 1
SIRT5	Sirtuin 5
NFKBIB	NFKB Inhibitor Beta
NME2	NME/NM23 Nucleoside Diphosphate Kinase 2
MIR346	MicroRNA 346
PRMT1	Protein Arginine Methyltransferase 1
MAX	MYC Associated Factor X
NSMF	NMDA Receptor Synaptonuclear Signaling And Neuronal Migration Factor
COLQ	Collagen Like Tail Subunit Of Asymmetric Acetylcholinesterase
AEBP1	AE Binding Protein 1
IAPP	Islet Amyloid Polypeptide
RRM2	Ribonucleotide Reductase Regulatory Subunit M2
CLDN5	Claudin 5
BAIAP2L1	BAR/IMD Domain Containing Adaptor Protein 2 Like 1
ECE1	Endothelin Converting Enzyme 1
ULK1	Unc-51 Like Autophagy Activating Kinase 1
IGF2BP2	Insulin Like Growth Factor 2 MRNA Binding Protein 2
ACADL	Acyl-CoA Dehydrogenase Long Chain
ANLN	Anillin Actin Binding Protein
PENK	Proenkephalin
YWHAB	Tyrosine 3-Monooxygenase/Tryptophan 5-Monooxygenase Activation Prote
H2BC21	H2B Clustered Histone 21
GOLPH3	Golgi Phosphoprotein 3
YWHAZ	Tyrosine 3-Monooxygenase/Tryptophan 5-Monooxygenase Activation Prote
FGF16	Fibroblast Growth Factor 16

ISG15	ISG15 Ubiquitin Like Modifier
GUCY2C	Guanylate Cyclase 2C
DUSP19	Dual Specificity Phosphatase 19
AHSG	Alpha 2-HS Glycoprotein
PABPN1	Poly(A) Binding Protein Nuclear 1
ZNF23	Zinc Finger Protein 23
HOTTIP	HOXA Distal Transcript Antisense RNA
ABHD11	Abhydrolase Domain Containing 11
PODXL	Podocalyxin Like
SULT1A1	Sulfotransferase Family 1A Member 1
LEFTY2	Left-Right Determination Factor 2
MRPL12	Mitochondrial Ribosomal Protein L12
SOCS2	Suppressor Of Cytokine Signaling 2
SERPING1	Serpin Family G Member 1
PNKD	PNKD Metallo-Beta-Lactamase Domain Containing
GTF2H4	General Transcription Factor IIH Subunit 4
DDIT4	DNA Damage Inducible Transcript 4
SRSF1	Serine And Arginine Rich Splicing Factor 1
TRPV1	Transient Receptor Potential Cation Channel Subfamily V Member 1
PRKACA	Protein Kinase CAMP-Activated Catalytic Subunit Alpha
CHRD	Chordin
PROK1	Prokineticin 1
JAG2	Jagged Canonical Notch Ligand 2
NCOA1	Nuclear Receptor Coactivator 1
HBE1	Hemoglobin Subunit Epsilon 1
HTR3A	5-Hydroxytryptamine Receptor 3A
TMEM150B	Transmembrane Protein 150B
GAR1	GAR1 Ribonucleoprotein
TIMP4	TIMP Metallopeptidase Inhibitor 4
SACM1L	SAC1 Like Phosphatidylinositide Phosphatase
ANG	Angiogenin
MIR212	MicroRNA 212
CYP3A5	Cytochrome P450 Family 3 Subfamily A Member 5
CLDN1	Claudin 1
ADIPOR2	Adiponectin Receptor 2
APOD	Apolipoprotein D
PIP	Prolactin Induced Protein
LAMB1	Laminin Subunit Beta 1
CYP51A1	Cytochrome P450 Family 51 Subfamily A Member 1
PCBD1	Pterin-4 Alpha-Carbinolamine Dehydratase 1
NR5A2	Nuclear Receptor Subfamily 5 Group A Member 2
DMP1	Dentin Matrix Acidic Phosphoprotein 1
MAGEA3	MAGE Family Member A3
IL4R	Interleukin 4 Receptor
NOP2	NOP2 Nucleolar Protein
S100A10	S100 Calcium Binding Protein A10
UBC	Ubiquitin C
PES1	Pescadillo Ribosomal Biogenesis Factor 1
SLC25A1	Solute Carrier Family 25 Member 1
CXCR3	C-X-C Motif Chemokine Receptor 3
ASIC1	Acid Sensing Ion Channel Subunit 1
RXRA	Retinoid X Receptor Alpha
ACSL3	Acyl-CoA Synthetase Long Chain Family Member 3
FZD1	Frizzled Class Receptor 1

EPHA1	EPH Receptor A1
SOD3	Superoxide Dismutase 3
MXD1	MAX Dimerization Protein 1
BNIP3	BCL2 Interacting Protein 3
GRM1	Glutamate Metabotropic Receptor 1
SNAP25	Synaptosome Associated Protein 25
CYSLTR1	Cysteinyl Leukotriene Receptor 1
CUL3	Cullin 3
NEIL2	Nei Like DNA Glycosylase 2
MYF6	Myogenic Factor 6
TAC1	Tachykinin Precursor 1
PPARD	Peroxisome Proliferator Activated Receptor Delta
LBP	Lipopolysaccharide Binding Protein
PRDX1	Peroxiredoxin 1
TTPA	Alpha Tocopherol Transfer Protein
PDE4A	Phosphodiesterase 4A
EGR1	Early Growth Response 1
NDUFS3	NADH:Ubiquinone Oxidoreductase Core Subunit S3
LY96	Lymphocyte Antigen 96
PIGR	Polymeric Immunoglobulin Receptor
NR0B2	Nuclear Receptor Subfamily 0 Group B Member 2
SLC1A2	Solute Carrier Family 1 Member 2
RPL7A	Ribosomal Protein L7a
DNM1	Dynamin 1
CLN3	CLN3 Lysosomal/Endosomal Transmembrane Protein, Battenin
H3C14	H3 Clustered Histone 14
SFN	Stratifin
ACKR1	Atypical Chemokine Receptor 1 (Duffy Blood Group)
F13A1	Coagulation Factor XIII A Chain
HLA-E	Major Histocompatibility Complex, Class I, E
NPC1L1	NPC1 Like Intracellular Cholesterol Transporter 1
SOX18	SRY-Box Transcription Factor 18
TBCE	Tubulin Folding Cofactor E
PTPRN	Protein Tyrosine Phosphatase Receptor Type N
PATZ1	POZ/BTB And AT Hook Containing Zinc Finger 1
EPHB2	EPH Receptor B2
GNB3	G Protein Subunit Beta 3
MRPS16	Mitochondrial Ribosomal Protein S16
NARS2	Asparaginyl-TRNA Synthetase 2, Mitochondrial
NMB	Neuromedin B
GLO1	Glyoxalase I
TRO	Trophinin
DEFB4A	Defensin Beta 4A
RPA2	Replication Protein A2
FCGRT	Fc Fragment Of IgG Receptor And Transporter
CDC20	Cell Division Cycle 20
FBLN2	Fibulin 2
PLA2G6	Phospholipase A2 Group VI
CPB2	Carboxypeptidase B2
PRDX5	Peroxiredoxin 5
MYF5	Myogenic Factor 5
PIBF1	Progesterone Immunomodulatory Binding Factor 1
LOXL2	Lysyl Oxidase Like 2
MIR125B2	MicroRNA 125b-2

SETDB1	SET Domain Bifurcated Histone Lysine Methyltransferase 1
BCHE	Butyrylcholinesterase
TPH2	Tryptophan Hydroxylase 2
TNS1	Tensin 1
PLA2G4A	Phospholipase A2 Group IVA
IGFBP7	Insulin Like Growth Factor Binding Protein 7
CDK12	Cyclin Dependent Kinase 12
PRPF8	Pre-MRNA Processing Factor 8
TCL1A	TCL1 Family AKT Coactivator A
RHAG	Rh Associated Glycoprotein
AKR1C3	Aldo-Keto Reductase Family 1 Member C3
RAB3GAP2	RAB3 GTPase Activating Non-Catalytic Protein Subunit 2
GET3	Guided Entry Of Tail-Anchored Proteins Factor 3, ATPase
HSD17B7	Hydroxysteroid 17-Beta Dehydrogenase 7
HLX	H2.0 Like Homeobox
DEPTOR	DEP Domain Containing MTOR Interacting Protein
F12	Coagulation Factor XII
ID2	Inhibitor Of DNA Binding 2
GALNT17	Polypeptide N-Acetylgalactosaminyltransferase 17
CDC73	Cell Division Cycle 73
ATF2	Activating Transcription Factor 2
PARS2	Prolyl-TRNA Synthetase 2, Mitochondrial
CD70	CD70 Molecule
TSPO	Translocator Protein
GJA8	Gap Junction Protein Alpha 8
ATP6AP2	ATPase H+ Transporting Accessory Protein 2
SYK	Spleen Associated Tyrosine Kinase
GTF2H1	General Transcription Factor IIH Subunit 1
WNT8B	Wnt Family Member 8B
SLC4A4	Solute Carrier Family 4 Member 4
TLE1	TLE Family Member 1, Transcriptional Corepressor
UBA1	Ubiquitin Like Modifier Activating Enzyme 1
WDR45B	WD Repeat Domain 45B
TRIM50	Tripartite Motif Containing 50
MT2A	Metallothionein 2A
PSMB4	Proteasome 20S Subunit Beta 4
AP2B1	Adaptor Related Protein Complex 2 Subunit Beta 1
SULT1E1	Sulfotransferase Family 1E Member 1
ANTXR2	ANTXR Cell Adhesion Molecule 2
LAMC2	Laminin Subunit Gamma 2
TUBA1B	Tubulin Alpha 1b
OSM	Oncostatin M
CARTPT	CART Prepropeptide
CLSPN	Claspin
YME1L1	YME1 Like 1 ATPase
PROK2	Prokineticin 2
KAT6A	Lysine Acetyltransferase 6A
TSHB	Thyroid Stimulating Hormone Subunit Beta
CHRNA2	Cholinergic Receptor Nicotinic Alpha 2 Subunit
CHRNB2	Cholinergic Receptor Nicotinic Beta 2 Subunit
PYCARD	PYD And CARD Domain Containing
CISH	Cytokine Inducible SH2 Containing Protein
KPNB1	Karyopherin Subunit Beta 1
GABRG3	Gamma-Aminobutyric Acid Type A Receptor Subunit Gamma3

HDAC4	Histone Deacetylase 4
NRIP1	Nuclear Receptor Interacting Protein 1
ACVR2B	Activin A Receptor Type 2B
WASL	WASP Like Actin Nucleation Promoting Factor
PEG10	Paternally Expressed 10
WNT1	Wnt Family Member 1
SLC7A7	Solute Carrier Family 7 Member 7
PRKCE	Protein Kinase C Epsilon
BCL11A	BAF Chromatin Remodeling Complex Subunit BCL11A
MAGED1	MAGE Family Member D1
THBS2	Thrombospondin 2
KIF1B	Kinesin Family Member 1B
RLN2	Relaxin 2
TFPI	Tissue Factor Pathway Inhibitor
UBE2D2	Ubiquitin Conjugating Enzyme E2 D2
PRKCSH	Protein Kinase C Substrate 80K-H
SSR1	Signal Sequence Receptor Subunit 1
AOC1	Amine Oxidase Copper Containing 1
CRABP1	Cellular Retinoic Acid Binding Protein 1
MAP4	Microtubule Associated Protein 4
EXOSC9	Exosome Component 9
ASXL3	ASXL Transcriptional Regulator 3
CSN2	Casein Beta
RPS27	Ribosomal Protein S27
FTH1	Ferritin Heavy Chain 1
MAVS	Mitochondrial Antiviral Signaling Protein
DSE	Dermatan Sulfate Epimerase
DNTT	DNA Nucleotidylexotransferase
LIMS2	LIM Zinc Finger Domain Containing 2
PTS	6-Pyruvoyltetrahydropterin Synthase
GPBAR1	G Protein-Coupled Bile Acid Receptor 1
ADAM10	ADAM Metallopeptidase Domain 10
CCL19	C-C Motif Chemokine Ligand 19
SLC19A1	Solute Carrier Family 19 Member 1
CD83	CD83 Molecule
ACLY	ATP Citrate Lyase
SNX19	Sorting Nexin 19
ASH2L	ASH2 Like, Histone Lysine Methyltransferase Complex Subunit
DPY30	Dpy-30 Histone Methyltransferase Complex Regulatory Subunit
HEYL	Hes Related Family BHLH Transcription Factor With YRPW Motif Like
OBSCN	Obscurin, Cytoskeletal Calmodulin And Titin-Interacting RhoGEF
CTNNAL1	Catenin Alpha Like 1
NISCH	Nischarin
CARS1	Cysteinyl-TRNA Synthetase 1
ODR4	Odr-4 GPCR Localization Factor Homolog
CD200	CD200 Molecule
IGFBP6	Insulin Like Growth Factor Binding Protein 6
RPS6KA6	Ribosomal Protein S6 Kinase A6
MIR27B	MicroRNA 27b
CNBP	CCHC-Type Zinc Finger Nucleic Acid Binding Protein
MT-ND4	Mitochondrially Encoded NADH:Ubiquinone Oxidoreductase Core Subunit
REV3L	REV3 Like, DNA Directed Polymerase Zeta Catalytic Subunit
NEUROD1	Neuronal Differentiation 1
GPX1	Glutathione Peroxidase 1

CYP2B6	Cytochrome P450 Family 2 Subfamily B Member 6
ACE2	Angiotensin I Converting Enzyme 2
UCP2	Uncoupling Protein 2
GAS2	Growth Arrest Specific 2
SEC63	SEC63 Homolog, Protein Translocation Regulator
APELA	Apelin Receptor Early Endogenous Ligand
ID3	Inhibitor Of DNA Binding 3, HLH Protein
PCDHGA8	Protocadherin Gamma Subfamily A, 8
SAA4	Serum Amyloid A4, Constitutive
UTS2	Urotensin 2
MIR1271	MicroRNA 1271
WNK4	WNK Lysine Deficient Protein Kinase 4
PDS5B	PDS5 Cohesin Associated Factor B
OGT	O-Linked N-Acetylglucosamine (GlcNAc) Transferase
AQP5	Aquaporin 5
PSMA1	Proteasome 20S Subunit Alpha 1
VARS2	Valyl-TRNA Synthetase 2, Mitochondrial
AGPS	Alkylglycerone Phosphate Synthase
CDCA7	Cell Division Cycle Associated 7
SLC5A1	Solute Carrier Family 5 Member 1
FOXA2	Forkhead Box A2
FARS2	Phenylalanyl-TRNA Synthetase 2, Mitochondrial
CLPX	Caseinolytic Mitochondrial Matrix Peptidase Chaperone Subunit X
CLRN1	Clarin 1
SRD5A2	Steroid 5 Alpha-Reductase 2
HMBS	Hydroxymethylbilane Synthase
MSTN	Myostatin
SEM1	SEM1 26S Proteasome Complex Subunit
FES	FES Proto-Oncogene, Tyrosine Kinase
CDKAL1	CDK5 Regulatory Subunit Associated Protein 1 Like 1
MFAP2	Microfibril Associated Protein 2
DDRGK1	DDRGK Domain Containing 1
AHCY	Adenosylhomocysteinase
FUCA2	Alpha-L-Fucosidase 2
TXNL4A	Thioredoxin Like 4A
PAX4	Paired Box 4
L2HGDH	L-2-Hydroxyglutarate Dehydrogenase
NORAD	Non-Coding RNA Activated By DNA Damage
UIMC1	Ubiquitin Interaction Motif Containing 1
CEP89	Centrosomal Protein 89
CCL18	C-C Motif Chemokine Ligand 18
OPRD1	Opioid Receptor Delta 1
MT-CO2	Mitochondrially Encoded Cytochrome C Oxidase II
FGB	Fibrinogen Beta Chain
SLCO1B3	Solute Carrier Organic Anion Transporter Family Member 1B3
CKS1B	CDC28 Protein Kinase Regulatory Subunit 1B
FCER2	Fc Fragment Of IgE Receptor II
MMP11	Matrix Metallopeptidase 11
MSR1	Macrophage Scavenger Receptor 1
HTRA1	HtrA Serine Peptidase 1
ZAP70	Zeta Chain Of T Cell Receptor Associated Protein Kinase 70
PROX1	Prospero Homeobox 1
SOST	Sclerostin
ONECUT2	One Cut Homeobox 2

RIT1	Ras Like Without CAAX 1
FBN3	Fibrillin 3
CGB7	Chorionic Gonadotropin Subunit Beta 7
AMD1	Adenosylmethionine Decarboxylase 1
USP14	Ubiquitin Specific Peptidase 14
DCT	Dopachrome Tautomerase
SNX10	Sorting Nexin 10
CALD1	Caldesmon 1
GPAM	Glycerol-3-Phosphate Acyltransferase, Mitochondrial
TFDP3	Transcription Factor Dp Family Member 3
ELK1	ETS Transcription Factor ELK1
UFL1	UFM1 Specific Ligase 1
MDH2	Malate Dehydrogenase 2
NTF4	Neurotrophin 4
HELQ	Helicase, POLQ Like
ATF3	Activating Transcription Factor 3
TCF20	Transcription Factor 20
LATS1	Large Tumor Suppressor Kinase 1
CRHR1	Corticotropin Releasing Hormone Receptor 1
GABRB3	Gamma-Aminobutyric Acid Type A Receptor Subunit Beta3
MEPE	Matrix Extracellular Phosphoglycoprotein
RBL1	RB Transcriptional Corepressor Like 1
ENPP3	Ectonucleotide Pyrophosphatase/Phosphodiesterase 3
CD99	CD99 Molecule (Xg Blood Group)
PNPLA4	Patatin Like Phospholipase Domain Containing 4
RAD9A	RAD9 Checkpoint Clamp Component A
WDR62	WD Repeat Domain 62
PABPC1	Poly(A) Binding Protein Cytoplasmic 1
RNF135	Ring Finger Protein 135
CD2	CD2 Molecule
RPS20P23	Ribosomal Protein S20 Pseudogene 23
ANXA4	Annexin A4
MAGEA4	MAGE Family Member A4
MIR136	MicroRNA 136
SERPINB3	Serpin Family B Member 3
TBX21	T-Box Transcription Factor 21
APOC1	Apolipoprotein C1
NR1H3	Nuclear Receptor Subfamily 1 Group H Member 3
PLA2G1B	Phospholipase A2 Group IB
TNKS	Tankyrase
UBR5	Ubiquitin Protein Ligase E3 Component N-Recognin 5
IGF2-AS	IGF2 Antisense RNA
WNT10B	Wnt Family Member 10B
PTGDS	Prostaglandin D2 Synthase
SNAP29	Synaptosome Associated Protein 29
ARF6	ADP Ribosylation Factor 6
ZNHIT3	Zinc Finger HIT-Type Containing 3
PNPLA2	Patatin Like Phospholipase Domain Containing 2
SLC4A2	Solute Carrier Family 4 Member 2
LYN	LYN Proto-Oncogene, Src Family Tyrosine Kinase
IKBKG	Inhibitor Of Nuclear Factor Kappa B Kinase Regulatory Subunit Gamma
MAPKAPK2	MAPK Activated Protein Kinase 2
HEY1	Hes Related Family BHLH Transcription Factor With YRPW Motif 1
ITGA5	Integrin Subunit Alpha 5

HOXD10	Homeobox D10
PDE3B	Phosphodiesterase 3B
CXCL9	C-X-C Motif Chemokine Ligand 9
CALB1	Calbindin 1
POU1F1	POU Class 1 Homeobox 1
CXCR1	C-X-C Motif Chemokine Receptor 1
LAMA5	Laminin Subunit Alpha 5
CX3CL1	C-X3-C Motif Chemokine Ligand 1
MIR101-1	MicroRNA 101-1
CHD4	Chromodomain Helicase DNA Binding Protein 4
RNASEL	Ribonuclease L
POLH	DNA Polymerase Eta
NUP98	Nucleoporin 98 And 96 Precursor
FUT2	Fucosyltransferase 2
BLVRB	Biliverdin Reductase B
NEFL	Neurofilament Light
LPP	LIM Domain Containing Preferred Translocation Partner In Lipoma
GLMN	Glomulin, FKBP Associated Protein
CDH17	Cadherin 17
GIP	Gastric Inhibitory Polypeptide
CLTC	Clathrin Heavy Chain
PAK1	P21 (RAC1) Activated Kinase 1
ATP2A1	ATPase Sarcoplasmic/Endoplasmic Reticulum Ca2+ Transporting 1
STIL	STIL Centriolar Assembly Protein
TRPC4	Transient Receptor Potential Cation Channel Subfamily C Member 4
CTPS1	CTP Synthase 1
IDE	Insulin Degrading Enzyme
SOX6	SRY-Box Transcription Factor 6
SOX5	SRY-Box Transcription Factor 5
POU2AF1	POU Class 2 Homeobox Associating Factor 1
SLC22A4	Solute Carrier Family 22 Member 4
CXCL2	C-X-C Motif Chemokine Ligand 2
ACAA1	Acetyl-CoA Acyltransferase 1
MYOZ1	Myozenin 1
NOD1	Nucleotide Binding Oligomerization Domain Containing 1
NEK2	NIMA Related Kinase 2
PCSK2	Proprotein Convertase Subtilisin/Kexin Type 2
ARL6	ADP Ribosylation Factor Like GTPase 6
MCF2	MCF.2 Cell Line Derived Transforming Sequence
CASP5	Caspase 5
DBI	Diazepam Binding Inhibitor, Acyl-CoA Binding Protein
PSMC4	Proteasome 26S Subunit, ATPase 4
GPX4	Glutathione Peroxidase 4
STUB1	STIP1 Homology And U-Box Containing Protein 1
P4HB	Prolyl 4-Hydroxylase Subunit Beta
TRPC1	Transient Receptor Potential Cation Channel Subfamily C Member 1
H4-16	H4 Histone 16
UCN	Urocortin
HDAC3	Histone Deacetylase 3
TRAF2	TNF Receptor Associated Factor 2
LIN9	Lin-9 DREAM MuvB Core Complex Component
MAGEF1	MAGE Family Member F1
MIR133A1	MicroRNA 133a-1
PSAT1	Phosphoserine Aminotransferase 1

GJC1	Gap Junction Protein Gamma 1
SLC13A5	Solute Carrier Family 13 Member 5
AIM2	Absent In Melanoma 2
NAP1L4	Nucleosome Assembly Protein 1 Like 4
KRT4	Keratin 4
NR2F2	Nuclear Receptor Subfamily 2 Group F Member 2
BBC3	BCL2 Binding Component 3
CES1	Carboxylesterase 1
FAM3C	FAM3 Metabolism Regulating Signaling Molecule C
TSLP	Thymic Stromal Lymphopoietin
ROR2	Receptor Tyrosine Kinase Like Orphan Receptor 2
PLCB4	Phospholipase C Beta 4
ATF6	Activating Transcription Factor 6
AFF4	AF4/FMR2 Family Member 4
PRB1	Proline Rich Protein BstNI Subfamily 1
TPX2	TPX2 Microtubule Nucleation Factor
OLR1	Oxidized Low Density Lipoprotein Receptor 1
GPNMB	Glycoprotein Nmb
CUX1	Cut Like Homeobox 1
DLX4	Distal-Less Homeobox 4
SLC22A2	Solute Carrier Family 22 Member 2
SMAD5	SMAD Family Member 5
FOXP1	Forkhead Box P1
PGRMC2	Progesterone Receptor Membrane Component 2
DGAT1	Diacylglycerol O-Acyltransferase 1
SOAT1	Sterol O-Acyltransferase 1
FADS2	Fatty Acid Desaturase 2
ATAD2	ATPase Family AAA Domain Containing 2
ITIH2	Inter-Alpha-Trypsin Inhibitor Heavy Chain 2
CUL7	Cullin 7
ECI2	Enoyl-CoA Delta Isomerase 2
MIR133B	MicroRNA 133b
RAD18	RAD18 E3 Ubiquitin Protein Ligase
TRIM21	Tripartite Motif Containing 21
SCG5	Secretogranin V
TGFBI	Transforming Growth Factor Beta Induced
ALDH3A1	Aldehyde Dehydrogenase 3 Family Member A1
KIF5A	Kinesin Family Member 5A
DDX58	DExD/H-Box Helicase 58
OPHN1	Oligophrenin 1
GJA3	Gap Junction Protein Alpha 3
GOLGA5	Golgin A5
TFR2	Transferrin Receptor 2
HOXA4	Homeobox A4
RPL3	Ribosomal Protein L3
HDAC2	Histone Deacetylase 2
PTGER3	Prostaglandin E Receptor 3
PTBP1	Polypyrimidine Tract Binding Protein 1
PRSS23	Serine Protease 23
CYP2C8	Cytochrome P450 Family 2 Subfamily C Member 8
CRELD1	Cysteine Rich With EGF Like Domains 1
WDR5	WD Repeat Domain 5
CKMT2	Creatine Kinase, Mitochondrial 2
AGK	Acylglycerol Kinase

VAPB	VAMP Associated Protein B And C
PTGIS	Prostaglandin I2 Synthase
VASH1	Vasohibin 1
TYK2	Tyrosine Kinase 2
PDP1	Pyruvate Dehyrogenase Phosphatase Catalytic Subunit 1
GDF2	Growth Differentiation Factor 2
WASHC5	WASH Complex Subunit 5
ROS1	ROS Proto-Oncogene 1, Receptor Tyrosine Kinase
ADCY7	Adenylate Cyclase 7
BAP1	BRCA1 Associated Protein 1
RREB1	Ras Responsive Element Binding Protein 1
MIR449B	MicroRNA 449b
TNFRSF4	TNF Receptor Superfamily Member 4
HMMR	Hyaluronan Mediated Motility Receptor
PRKAR1B	Protein Kinase CAMP-Dependent Type I Regulatory Subunit Beta
LDHA	Lactate Dehydrogenase A
C19orf33	Chromosome 19 Open Reading Frame 33
SGCG	Sarcoglycan Gamma
NCS1	Neuronal Calcium Sensor 1
SLC25A6	Solute Carrier Family 25 Member 6
CELF2	CUGBP Elav-Like Family Member 2
FPGS	Folylpolyglutamate Synthase
HSF1	Heat Shock Transcription Factor 1
NRGN	Neurogranin
ASH1L	ASH1 Like Histone Lysine Methyltransferase
GCLM	Glutamate-Cysteine Ligase Modifier Subunit
ADCY2	Adenylate Cyclase 2
SMURF1	SMAD Specific E3 Ubiquitin Protein Ligase 1
DNAJB1	DnaJ Heat Shock Protein Family (Hsp40) Member B1
CRABP2	Cellular Retinoic Acid Binding Protein 2
CADM1	Cell Adhesion Molecule 1
RBFOX1	RNA Binding Fox-1 Homolog 1
F11	Coagulation Factor XI
OPTN	Optineurin
STIP1	Stress Induced Phosphoprotein 1
NOX4	NADPH Oxidase 4
TAB2	TGF-Beta Activated Kinase 1 (MAP3K7) Binding Protein 2
AHSP	Alpha Hemoglobin Stabilizing Protein
MIR19A	MicroRNA 19a
ITIH4	Inter-Alpha-Trypsin Inhibitor Heavy Chain 4
FBXO32	F-Box Protein 32
PIK3C3	Phosphatidylinositol 3-Kinase Catalytic Subunit Type 3
ATG14	Autophagy Related 14
ITGA2B	Integrin Subunit Alpha 2b
MRPL23	Mitochondrial Ribosomal Protein L23
CHST11	Carbohydrate Sulfotransferase 11
CENPA	Centromere Protein A
IDH3G	Isocitrate Dehydrogenase (NAD(+)) 3 Non-Catalytic Subunit Gamma
WAPL	WAPL Cohesin Release Factor
BMP10	Bone Morphogenetic Protein 10
CELF1	CUGBP Elav-Like Family Member 1
TXNIP	Thioredoxin Interacting Protein
HAVCR1	Hepatitis A Virus Cellular Receptor 1
TFE3	Transcription Factor Binding To IGHM Enhancer 3

ENPEP	Glutamyl Aminopeptidase
STARD3	StAR Related Lipid Transfer Domain Containing 3
SKIL	SKI Like Proto-Oncogene
PTPN6	Protein Tyrosine Phosphatase Non-Receptor Type 6
CYB561	Cytochrome B561
EIPR1	EARP Complex And GARP Complex Interacting Protein 1
RFWD3	Ring Finger And WD Repeat Domain 3
LAMC1	Laminin Subunit Gamma 1
PRKCZ	Protein Kinase C Zeta
NDUFS4	NADH:Ubiquinone Oxidoreductase Subunit S4
ADORA2A	Adenosine A2a Receptor
HES1	Hes Family BHLH Transcription Factor 1
ADH4	Alcohol Dehydrogenase 4 (Class II), Pi Polypeptide
HACD1	3-Hydroxyacyl-CoA Dehydratase 1
IL27	Interleukin 27
ISL1	ISL LIM Homeobox 1
SCAP	SREBF Chaperone
TSSC4	Tumor Suppressing Subtransferable Candidate 4
DUS3L	Dihydrouridine Synthase 3 Like
MDM4	MDM4 Regulator Of P53
PVALB	Parvalbumin
HSD17B12	Hydroxysteroid 17-Beta Dehydrogenase 12
IRF7	Interferon Regulatory Factor 7
RARRES2	Retinoic Acid Receptor Responder 2
PSMB2	Proteasome 20S Subunit Beta 2
HUS1	HUS1 Checkpoint Clamp Component
LRRK2	Leucine Rich Repeat Kinase 2
TACSTD2	Tumor Associated Calcium Signal Transducer 2
ALKBH5	AlkB Homolog 5, RNA Demethylase
SLC1A5	Solute Carrier Family 1 Member 5
HPGDS	Hematopoietic Prostaglandin D Synthase
RPSA	Ribosomal Protein SA
ST3GAL4	ST3 Beta-Galactoside Alpha-2,3-Sialyltransferase 4
UBE2L3	Ubiquitin Conjugating Enzyme E2 L3
COASY	Coenzyme A Synthase
GRHL2	Grainyhead Like Transcription Factor 2
PCM1	Pericentriolar Material 1
LCN1	Lipocalin 1
USP2	Ubiquitin Specific Peptidase 2
ZNF496	Zinc Finger Protein 496
UCP3	Uncoupling Protein 3
SLC35A1	Solute Carrier Family 35 Member A1
NEDD4L	NEDD4 Like E3 Ubiquitin Protein Ligase
TAF4B	TATA-Box Binding Protein Associated Factor 4b
ETS2	ETS Proto-Oncogene 2, Transcription Factor
REL	REL Proto-Oncogene, NF-KB Subunit
TXN2	Thioredoxin 2
CDK9	Cyclin Dependent Kinase 9
BHLHE40	Basic Helix-Loop-Helix Family Member E40
SMC2	Structural Maintenance Of Chromosomes 2
OPA1	OPA1 Mitochondrial Dynamin Like GTPase
HSP90AB1	Heat Shock Protein 90 Alpha Family Class B Member 1
YBX2	Y-Box Binding Protein 2
FXYD1	FXYD Domain Containing Ion Transport Regulator 1

AQR	Aquarius Intron-Binding Spliceosomal Factor
LMLN	Leishmanolysin Like Peptidase
FZR1	Fizzy And Cell Division Cycle 20 Related 1
KLHDC2	Kelch Domain Containing 2
GJB3	Gap Junction Protein Beta 3
SH3BP4	SH3 Domain Binding Protein 4
GJB4	Gap Junction Protein Beta 4
GSS	Glutathione Synthetase
HOXD12	Homeobox D12
PLCZ1	Phospholipase C Zeta 1
ESM1	Endothelial Cell Specific Molecule 1
MSN	Moesin
SPRY4	Sprouty RTK Signaling Antagonist 4
LHX3	LIM Homeobox 3
MKKS	McKusick-Kaufman Syndrome
TRIM37	Tripartite Motif Containing 37
GSTA3	Glutathione S-Transferase Alpha 3
PRPF31	Pre-MRNA Processing Factor 31
PAK3	P21 (RAC1) Activated Kinase 3
CXCL16	C-X-C Motif Chemokine Ligand 16
ITLN1	Intelectin 1
LDHB	Lactate Dehydrogenase B
XPO5	Exportin 5
HJURP	Holliday Junction Recognition Protein
ZMYND10	Zinc Finger MYND-Type Containing 10
EGLN1	Egl-9 Family Hypoxia Inducible Factor 1
SIL1	SIL1 Nucleotide Exchange Factor
AMELX	Amelogenin X-Linked
FRZB	Frizzled Related Protein
RAB1A	RAB1A, Member RAS Oncogene Family
SEC24B	SEC24 Homolog B, COPII Coat Complex Component
ACBD5	Acyl-CoA Binding Domain Containing 5
HIKESHI	Heat Shock Protein Nuclear Import Factor Hikeshi
FJX1	Four-Jointed Box Kinase 1
MACO1	Macoilin 1
GALNT3	Polypeptide N-Acetylgalactosaminyltransferase 3
BUB3	BUB3 Mitotic Checkpoint Protein
ACAD9	Acyl-CoA Dehydrogenase Family Member 9
ARHGDIA	Rho GDP Dissociation Inhibitor Alpha
DEFB103B	Defensin Beta 103B
MBD2	Methyl-CpG Binding Domain Protein 2
CUL4B	Cullin 4B
NUP210	Nucleoporin 210
MARCKS	Myristoylated Alanine Rich Protein Kinase C Substrate
MAP2K6	Mitogen-Activated Protein Kinase Kinase 6
CAPN2	Calpain 2
TAP1	Transporter 1, ATP Binding Cassette Subfamily B Member
SETD1B	SET Domain Containing 1B, Histone Lysine Methyltransferase
WDR70	WD Repeat Domain 70
DAPK1	Death Associated Protein Kinase 1
EIF4A3	Eukaryotic Translation Initiation Factor 4A3
SLC1A1	Solute Carrier Family 1 Member 1
KRIT1	KRIT1 Ankyrin Repeat Containing
TUBA1A	Tubulin Alpha 1a

TMEM87A	Transmembrane Protein 87A
OPRL1	Opioid Related Nociceptin Receptor 1
TP53COR1	Tumor Protein P53 Pathway Corepressor 1
NEK3	NIMA Related Kinase 3
FBXO5	F-Box Protein 5
DVL3	Dishevelled Segment Polarity Protein 3
AP1M1	Adaptor Related Protein Complex 1 Subunit Mu 1
VSIR	V-Set Immunoregulatory Receptor
ATG7	Autophagy Related 7
WNT6	Wnt Family Member 6
NEUROG1	Neurogenin 1
OAS1	2'-5'-Oligoadenylate Synthetase 1
IQGAP1	IQ Motif Containing GTPase Activating Protein 1
SLC30A8	Solute Carrier Family 30 Member 8
RPS18	Ribosomal Protein S18
IFIH1	Interferon Induced With Helicase C Domain 1
PDIA3	Protein Disulfide Isomerase Family A Member 3
TNFSF13	TNF Superfamily Member 13
PARD3B	Par-3 Family Cell Polarity Regulator Beta
SELENOS	Selenoprotein S
PTGFR	Prostaglandin F Receptor
OLIG2	Oligodendrocyte Transcription Factor 2
COL4A2	Collagen Type IV Alpha 2 Chain
DAXX	Death Domain Associated Protein
PAX9	Paired Box 9
ZNF214	Zinc Finger Protein 214
PDHX	Pyruvate Dehydrogenase Complex Component X
NPY2R	Neuropeptide Y Receptor Y2
BBS7	Bardet-Biedl Syndrome 7
SLIT2	Slit Guidance Ligand 2
UBE2I	Ubiquitin Conjugating Enzyme E2 I
TACC1	Transforming Acidic Coiled-Coil Containing Protein 1
MT-ND5	Mitochondrially Encoded NADH:Ubiquinone Oxidoreductase Core Subunit
HTT	Huntingtin
MIR411	MicroRNA 411
DUSP6	Dual Specificity Phosphatase 6
HOXB1	Homeobox B1
CANX	Calnexin
HRH2	Histamine Receptor H2
SH2B3	SH2B Adaptor Protein 3
NDUFS8	NADH:Ubiquinone Oxidoreductase Core Subunit S8
VLDLR	Very Low Density Lipoprotein Receptor
PTOV1	PTOV1 Extended AT-Hook Containing Adaptor Protein
SLC12A2	Solute Carrier Family 12 Member 2
ACVRL1	Activin A Receptor Like Type 1
CTSK	Cathepsin K
NBAS	NBAS Subunit Of NRZ Tethering Complex
IL13RA2	Interleukin 13 Receptor Subunit Alpha 2
MAOB	Monoamine Oxidase B
SULF2	Sulfatase 2
PUM1	Pumilio RNA Binding Family Member 1
CLIP1	CAP-Gly Domain Containing Linker Protein 1
CANT1	Calcium Activated Nucleotidase 1
NT5E	5'-Nucleotidase Ecto

SLC12A4	Solute Carrier Family 12 Member 4
CCL20	C-C Motif Chemokine Ligand 20
PDXDC1	Pyridoxal Dependent Decarboxylase Domain Containing 1
HMCN1	Hemicentin 1
FOSL2	FOS Like 2, AP-1 Transcription Factor Subunit
ABCC4	ATP Binding Cassette Subfamily C Member 4
ZNF695	Zinc Finger Protein 695
CERK	Ceramide Kinase
CASK	Calcium/Calmodulin Dependent Serine Protein Kinase
AKR1C2	Aldo-Keto Reductase Family 1 Member C2
MAP3K14	Mitogen-Activated Protein Kinase Kinase Kinase 14
PSMC6	Proteasome 26S Subunit, ATPase 6
DGKB	Diacylglycerol Kinase Beta
MAN1B1	Mannosidase Alpha Class 1B Member 1
SRF	Serum Response Factor
FYN	FYN Proto-Oncogene, Src Family Tyrosine Kinase
DRD1	Dopamine Receptor D1
ADAMTS16	ADAM Metallopeptidase With Thrombospondin Type 1 Motif 16
KCNK9	Potassium Two Pore Domain Channel Subfamily K Member 9
UBE2D3	Ubiquitin Conjugating Enzyme E2 D3
CCDC120	Coiled-Coil Domain Containing 120
EPHB3	EPH Receptor B3
GNAI1	G Protein Subunit Alpha I1
SLCO1B1	Solute Carrier Organic Anion Transporter Family Member 1B1
LAT	Linker For Activation Of T Cells
KCNN3	Potassium Calcium-Activated Channel Subfamily N Member 3
ACSL6	Acyl-CoA Synthetase Long Chain Family Member 6
JUNB	JunB Proto-Oncogene, AP-1 Transcription Factor Subunit
CCR9	C-C Motif Chemokine Receptor 9
GATM	Glycine Amidinotransferase
ROCK2	Rho Associated Coiled-Coil Containing Protein Kinase 2
MID2	Midline 2
DGCR8	DGCR8 Microprocessor Complex Subunit
SLIT3	Slit Guidance Ligand 3
IL18BP	Interleukin 18 Binding Protein
DRD3	Dopamine Receptor D3
EPHA3	EPH Receptor A3
DEPDC5	DEP Domain Containing 5, GATOR1 Subcomplex Subunit
TERF2IP	TERF2 Interacting Protein
PLCB1	Phospholipase C Beta 1
TXNRD1	Thioredoxin Reductase 1
ATG9A	Autophagy Related 9A
VPS35	VPS35 Retromer Complex Component
SIM2	SIM BHLH Transcription Factor 2
MUC3A	Mucin 3A, Cell Surface Associated
TACC3	Transforming Acidic Coiled-Coil Containing Protein 3
SAR1B	Secretion Associated Ras Related GTPase 1B
FALEC	Focally Amplified Long Non-Coding RNA In Epithelial Cancer
TRIM28	Tripartite Motif Containing 28
TMOD1	Tropomodulin 1
MIR1908	MicroRNA 1908
TTC8	Tetratricopeptide Repeat Domain 8
EPS15	Epidermal Growth Factor Receptor Pathway Substrate 15
TOP3B	DNA Topoisomerase III Beta

COL16A1	Collagen Type XVI Alpha 1 Chain
NAP1L5	Nucleosome Assembly Protein 1 Like 5
TMEM258	Transmembrane Protein 258
PER2	Period Circadian Regulator 2
NCOA6	Nuclear Receptor Coactivator 6
TNC	Tenascin C
KLK15	Kallikrein Related Peptidase 15
WIF1	WNT Inhibitory Factor 1
PPP1R3A	Protein Phosphatase 1 Regulatory Subunit 3A
BACH1	BTB Domain And CNC Homolog 1
SLC25A3	Solute Carrier Family 25 Member 3
HCK	HCK Proto-Oncogene, Src Family Tyrosine Kinase
MIR216A	MicroRNA 216a
SLC3A2	Solute Carrier Family 3 Member 2
CX3CR1	C-X3-C Motif Chemokine Receptor 1
PPP2R3C	Protein Phosphatase 2 Regulatory Subunit B''Gamma
ITFG1	Integrin Alpha FG-GAP Repeat Containing 1
PARK7	Parkinsonism Associated Deglycase
CEP70	Centrosomal Protein 70
MGAM	Maltase-Glucoamylase
EMP1	Epithelial Membrane Protein 1
FMO3	Flavin Containing Dimethylaniline Monoxygenase 3
CEND1	Cell Cycle Exit And Neuronal Differentiation 1
ROBO1	Roundabout Guidance Receptor 1
TMEM199	Transmembrane Protein 199
MC3R	Melanocortin 3 Receptor
HAVCR2	Hepatitis A Virus Cellular Receptor 2
AGPAT1	1-Acylglycerol-3-Phosphate O-Acyltransferase 1
GSTZ1	Glutathione S-Transferase Zeta 1
SCD5	Stearoyl-CoA Desaturase 5
VKORC1L1	Vitamin K Epoxide Reductase Complex Subunit 1 Like 1
CERS4	Ceramide Synthase 4
GPM6B	Glycoprotein M6B
LPCAT3	Lysophosphatidylcholine Acyltransferase 3
BBX	BBX High Mobility Group Box Domain Containing
PEBP1	Phosphatidylethanolamine Binding Protein 1
SRSF2	Serine And Arginine Rich Splicing Factor 2
FOXO4	Forkhead Box O4
ZNF142	Zinc Finger Protein 142
GPI	Glucose-6-Phosphate Isomerase
GNB1	G Protein Subunit Beta 1
HRH1	Histamine Receptor H1
IFI16	Interferon Gamma Inducible Protein 16
FABP12	Fatty Acid Binding Protein 12
HAS1	Hyaluronan Synthase 1
NUP93	Nucleoporin 93
PRKCI	Protein Kinase C Iota
PRDX3	Peroxiredoxin 3
BAMBI	BMP And Activin Membrane Bound Inhibitor
PHGDH	Phosphoglycerate Dehydrogenase
ELP1	Elongator Complex Protein 1
SLC6A9	Solute Carrier Family 6 Member 9
YWHAE	Tyrosine 3-Monooxygenase/Tryptophan 5-Monooxygenase Activation Prote
DKK3	Dickkopf WNT Signaling Pathway Inhibitor 3

KRT6A	Keratin 6A
NCL	Nucleolin
SUPT5H	SPT5 Homolog, DSIF Elongation Factor Subunit
SMOX	Spermine Oxidase
KAT2B	Lysine Acetyltransferase 2B
THY1	Thy-1 Cell Surface Antigen
MAP4K4	Mitogen-Activated Protein Kinase Kinase Kinase Kinase 4
IL10RA	Interleukin 10 Receptor Subunit Alpha
VAMP4	Vesicle Associated Membrane Protein 4
ICAM5	Intercellular Adhesion Molecule 5
IFNGR2	Interferon Gamma Receptor 2
ID1	Inhibitor Of DNA Binding 1, HLH Protein
TRPM4	Transient Receptor Potential Cation Channel Subfamily M Member 4
NDUFA13	NADH:Ubiquinone Oxidoreductase Subunit A13
MCM5	Minichromosome Maintenance Complex Component 5
AOPEP	Aminopeptidase O (Putative)
EHF	ETS Homologous Factor
CAP1	Cyclase Associated Actin Cytoskeleton Regulatory Protein 1
MYO6	Myosin VI
CSNK2A1	Casein Kinase 2 Alpha 1
GNLY	Granulysin
COL14A1	Collagen Type XIV Alpha 1 Chain
COIL	Coilin
CEBPA	CCAAT Enhancer Binding Protein Alpha
FUT1	Fucosyltransferase 1 (H Blood Group)
SGCB	Sarcoglycan Beta
MSMB	Microseminoprotein Beta
NRF1	Nuclear Respiratory Factor 1
PKN1	Protein Kinase N1
TRA	T Cell Receptor Alpha Locus
DDOST	Dolichyl-Diphosphooligosaccharide--Protein Glycosyltransferase Non-C
NLRP11	NLR Family Pyrin Domain Containing 11
LPO	Lactoperoxidase
HPX	Hemopexin
OPRK1	Opioid Receptor Kappa 1
VAPA	VAMP Associated Protein A
PPA2	Inorganic Pyrophosphatase 2
EEF1B2	Eukaryotic Translation Elongation Factor 1 Beta 2
PDLIM5	PDZ And LIM Domain 5
TYRO3	TYRO3 Protein Tyrosine Kinase
C1QBP	Complement C1q Binding Protein
THRA	Thyroid Hormone Receptor Alpha
KLKB1	Kallikrein B1
PSMD7	Proteasome 26S Subunit, Non-ATPase 7
CDC27	Cell Division Cycle 27
H4C1	H4 Clustered Histone 1
TNFAIP6	TNF Alpha Induced Protein 6
INTS11	Integrator Complex Subunit 11
CSK	C-Terminal Src Kinase
CCL25	C-C Motif Chemokine Ligand 25
PSMD1	Proteasome 26S Subunit, Non-ATPase 1
PTPN2	Protein Tyrosine Phosphatase Non-Receptor Type 2
NPR3	Natriuretic Peptide Receptor 3
RXRB	Retinoid X Receptor Beta

BCL2L2	BCL2 Like 2
ADCY5	Adenylate Cyclase 5
MMP26	Matrix Metallopeptidase 26
BCAT1	Branched Chain Amino Acid Transaminase 1
DTL	Denticleless E3 Ubiquitin Protein Ligase Homolog
CYP2J2	Cytochrome P450 Family 2 Subfamily J Member 2
RNF8	Ring Finger Protein 8
SIK2	Salt Inducible Kinase 2
USP44	Ubiquitin Specific Peptidase 44
CCL22	C-C Motif Chemokine Ligand 22
TRAP1	TNF Receptor Associated Protein 1
HSP90B1	Heat Shock Protein 90 Beta Family Member 1
FTL	Ferritin Light Chain
PRRT2	Proline Rich Transmembrane Protein 2
FPR1	Formyl Peptide Receptor 1
MAPK7	Mitogen-Activated Protein Kinase 7
TRIM32	Tripartite Motif Containing 32
SUMO1	Small Ubiquitin Like Modifier 1
MIR217	MicroRNA 217
CBX3	Chromobox 3
SERPINB7	Serpin Family B Member 7
MALT1	MALT1 Paracaspase
NCR3	Natural Cytotoxicity Triggering Receptor 3
CCDC114	Coiled-Coil Domain Containing 114
UBE2A	Ubiquitin Conjugating Enzyme E2 A
KLF5	Kruppel Like Factor 5
DVL1	Dishevelled Segment Polarity Protein 1
PIN1	Peptidylprolyl Cis/Trans Isomerase, NIMA-Interacting 1
GPX5	Glutathione Peroxidase 5
KCNK2	Potassium Two Pore Domain Channel Subfamily K Member 2
TM7SF3	Transmembrane 7 Superfamily Member 3
MICB	MHC Class I Polypeptide-Related Sequence B
EME1	Essential Meiotic Structure-Specific Endonuclease 1
TFB1M	Transcription Factor B1, Mitochondrial
ACVR1C	Activin A Receptor Type 1C
NXF2	Nuclear RNA Export Factor 2
CTRL	Chymotrypsin Like
IMPA1	Inositol Monophosphatase 1
IL17F	Interleukin 17F
MAP2K3	Mitogen-Activated Protein Kinase Kinase 3
ERLIN2	ER Lipid Raft Associated 2
PRKCQ	Protein Kinase C Theta
PSIP1	PC4 And SFRS1 Interacting Protein 1
PTPRU	Protein Tyrosine Phosphatase Receptor Type U
ETV5	ETS Variant Transcription Factor 5
TBK1	TANK Binding Kinase 1
CEACAM1	CEA Cell Adhesion Molecule 1
BMP3	Bone Morphogenetic Protein 3
MYH10	Myosin Heavy Chain 10
SLC25A19	Solute Carrier Family 25 Member 19
FBP1	Fructose-Bisphosphatase 1
KIF14	Kinesin Family Member 14
PNPLA3	Patatin Like Phospholipase Domain Containing 3
CXCL5	C-X-C Motif Chemokine Ligand 5

TRAF1	TNF Receptor Associated Factor 1
INHBC	Inhibin Subunit Beta C
EIF2AK2	Eukaryotic Translation Initiation Factor 2 Alpha Kinase 2
BTRC	Beta-Transducin Repeat Containing E3 Ubiquitin Protein Ligase
BCL2A1	BCL2 Related Protein A1
OVGP1	Oviductal Glycoprotein 1
CDKL5	Cyclin Dependent Kinase Like 5
MSBP1	Minisatellite Binding Protein 1
MAP1LC3A	Microtubule Associated Protein 1 Light Chain 3 Alpha
TUBB1	Tubulin Beta 1 Class VI
ARCN1	Archain 1
PSMD3	Proteasome 26S Subunit, Non-ATPase 3
PSMD11	Proteasome 26S Subunit, Non-ATPase 11
PSMC2	Proteasome 26S Subunit, ATPase 2
PLS3	Plastin 3
ADIPQTL4	Adiponectin, Serum Level Of, QTL4
TREX1	Three Prime Repair Exonuclease 1
PSMD10	Proteasome 26S Subunit, Non-ATPase 10
CRAT	Carnitine O-Acetyltransferase
BRD7	Bromodomain Containing 7
DROSHA	Drosha Ribonuclease III
FIBP	FGF1 Intracellular Binding Protein
DMBT1	Deleted In Malignant Brain Tumors 1
APOBEC3G	Apolipoprotein B MRNA Editing Enzyme Catalytic Subunit 3G
CBX1	Chromobox 1
TBL1XR1	TBL1X Receptor 1
EPM2A	EPM2A Glucan Phosphatase, Laforin
OGDH	Oxoglutarate Dehydrogenase
CYP2A6	Cytochrome P450 Family 2 Subfamily A Member 6
TMEM107	Transmembrane Protein 107
TNXA	Tenascin XA (Pseudogene)
B3GLCT	Beta 3-Glucosyltransferase
XAF1	XIAP Associated Factor 1
ECT2	Epithelial Cell Transforming 2
ADAMTS9	ADAM Metallopeptidase With Thrombospondin Type 1 Motif 9
PPIF	Peptidylprolyl Isomerase F
EIF5A	Eukaryotic Translation Initiation Factor 5A
F11R	F11 Receptor
SKP1	S-Phase Kinase Associated Protein 1
SP3	Sp3 Transcription Factor
C5AR1	Complement C5a Receptor 1
UBTF	Upstream Binding Transcription Factor
ARRB2	Arrestin Beta 2
CHFR	Checkpoint With Forkhead And Ring Finger Domains
ZFX	Zinc Finger Protein X-Linked
UGT2B7	UDP Glucuronosyltransferase Family 2 Member B7
AQP8	Aquaporin 8
GART	Phosphoribosylglycinamide Formyltransferase, Phosphoribosylglycinami
PANX1	Pannexin 1
GZMA	Granzyme A
MAP2	Microtubule Associated Protein 2
HK1	Hexokinase 1
TMEM86A	Transmembrane Protein 86A
TAPBP	TAP Binding Protein

DRD4	Dopamine Receptor D4
FDFT1	Farnesyl-Diphosphate Farnesyltransferase 1
LRPAP1	LDL Receptor Related Protein Associated Protein 1
PSMA3	Proteasome 20S Subunit Alpha 3
RAPGEF3	Rap Guanine Nucleotide Exchange Factor 3
XPO7	Exportin 7
TTF2	Transcription Termination Factor 2
SLC46A1	Solute Carrier Family 46 Member 1
FGF14	Fibroblast Growth Factor 14
KAT5	Lysine Acetyltransferase 5
ANGPTL2	Angiopoietin Like 2
MIR328	MicroRNA 328
RARG	Retinoic Acid Receptor Gamma
WNT2	Wnt Family Member 2
BST2	Bone Marrow Stromal Cell Antigen 2
IGKC	Immunoglobulin Kappa Constant
C4BPA	Complement Component 4 Binding Protein Alpha
MAGEA11	MAGE Family Member A11
SLC34A2	Solute Carrier Family 34 Member 2
CTH	Cystathionine Gamma-Lyase
MYOZ2	Myozenin 2
RAB18	RAB18, Member RAS Oncogene Family
HSPA6	Heat Shock Protein Family A (Hsp70) Member 6
MYOC	Myocilin
DOCK1	Dedicator Of Cytokinesis 1
PDYN	Prodynorphin
KDM5B	Lysine Demethylase 5B
USP15	Ubiquitin Specific Peptidase 15
VANGL2	VANGL Planar Cell Polarity Protein 2
VGF	VGF Nerve Growth Factor Inducible
EXT1	Exostosin Glycosyltransferase 1
KIR2DL1	Killer Cell Immunoglobulin Like Receptor, Two Ig Domains And Long Cy
NAT1	N-Acetyltransferase 1
DYNLL1	Dynein Light Chain LC8-Type 1
KLK2	Kallikrein Related Peptidase 2
COPB2	COPI Coat Complex Subunit Beta 2
PSMA7	Proteasome 20S Subunit Alpha 7
HSPE1	Heat Shock Protein Family E (Hsp10) Member 1
TUBB4A	Tubulin Beta 4A Class IVa
CKAP5	Cytoskeleton Associated Protein 5
TUBG1	Tubulin Gamma 1
CREM	CAMP Responsive Element Modulator
GDF1	Growth Differentiation Factor 1
BHMT	Betaine--Homocysteine S-Methyltransferase
CCM2	CCM2 Scaffold Protein
LOC108783649	AMH 5' Regulatory Region
TBX3	T-Box Transcription Factor 3
DEK	DEK Proto-Oncogene
IFNAR2	Interferon Alpha And Beta Receptor Subunit 2
PSMA2	Proteasome 20S Subunit Alpha 2
PRRC2A	Proline Rich Coiled-Coil 2A
DCTN2	Dynactin Subunit 2
COL4A6	Collagen Type IV Alpha 6 Chain
SERPINA12	Serpin Family A Member 12

ITGA1	Integrin Subunit Alpha 1
CSPG4	Chondroitin Sulfate Proteoglycan 4
NUAK2	NUAK Family Kinase 2
GP2	Glycoprotein 2
PEPD	Peptidase D
ITGB8	Integrin Subunit Beta 8
MCCC2	Methylcrotonoyl-CoA Carboxylase 2
TRA2B	Transformer 2 Beta Homolog
NCOR2	Nuclear Receptor Corepressor 2
CACNA1G	Calcium Voltage-Gated Channel Subunit Alpha1 G
SALL2	Spalt Like Transcription Factor 2
GNL3	G Protein Nucleolar 3
RNASE2	Ribonuclease A Family Member 2
IGFALS	Insulin Like Growth Factor Binding Protein Acid Labile Subunit
CA4	Carbonic Anhydrase 4
GABBR2	Gamma-Aminobutyric Acid Type B Receptor Subunit 2
SLC38A2	Solute Carrier Family 38 Member 2
DIO1	Iodothyronine Deiodinase 1
HOXB4	Homeobox B4
CAPN1	Calpain 1
STK4	Serine/Threonine Kinase 4
SCAMP1	Secretory Carrier Membrane Protein 1
SLC1A3	Solute Carrier Family 1 Member 3
B4GALT1	Beta-1,4-Galactosyltransferase 1
SMARCE1	SWI/SNF Related, Matrix Associated, Actin Dependent Regulator Of Chr
INTS4	Integrator Complex Subunit 4
DOT1L	DOT1 Like Histone Lysine Methyltransferase
PSMD9	Proteasome 26S Subunit, Non-ATPase 9
SET	SET Nuclear Proto-Oncogene
MRPL45	Mitochondrial Ribosomal Protein L45
CDK5RAP2	CDK5 Regulatory Subunit Associated Protein 2
NUDT6	Nudix Hydrolase 6
PARP2	Poly(ADP-Ribose) Polymerase 2
IL3RA	Interleukin 3 Receptor Subunit Alpha
INSRR	Insulin Receptor Related Receptor
UBE2N	Ubiquitin Conjugating Enzyme E2 N
PSMB3	Proteasome 20S Subunit Beta 3
CAST	Calpastatin
PSMB1	Proteasome 20S Subunit Beta 1
RTN1	Reticulon 1
DLX1	Distal-Less Homeobox 1
DERL1	Derlin 1
SH3KBP1	SH3 Domain Containing Kinase Binding Protein 1
TBX2	T-Box Transcription Factor 2
CD177	CD177 Molecule
EEF1A1	Eukaryotic Translation Elongation Factor 1 Alpha 1
FOSB	FosB Proto-Oncogene, AP-1 Transcription Factor Subunit
PRDX2	Peroxiredoxin 2
ITGB6	Integrin Subunit Beta 6
SLC12A7	Solute Carrier Family 12 Member 7
CBR3	Carbonyl Reductase 3
NCR2	Natural Cytotoxicity Triggering Receptor 2
HYAL1	Hyaluronidase 1
DDAH2	Dimethylarginine Dimethylaminohydrolase 2

NCR1	Natural Cytotoxicity Triggering Receptor 1
ZMYND11	Zinc Finger MYND-Type Containing 11
HOXB5	Homeobox B5
NHS	NHS Actin Remodeling Regulator
CYLD	CYLD Lysine 63 Deubiquitinase
PCBP1	Poly(RC) Binding Protein 1
CUL5	Cullin 5
KTN1	Kinectin 1
UQCRC2	Ubiquinol-Cytochrome C Reductase Core Protein 2
SLC2A6	Solute Carrier Family 2 Member 6
DRD5	Dopamine Receptor D5
RNF5	Ring Finger Protein 5
PAF1	PAF1 Homolog, Paf1/RNA Polymerase II Complex Component
ALOX15B	Arachidonate 15-Lipoxygenase Type B
PRG2	Proteoglycan 2, Pro Eosinophil Major Basic Protein
OGN	Osteoglycin
COL24A1	Collagen Type XXIV Alpha 1 Chain
GSTM2	Glutathione S-Transferase Mu 2
KNL1	Kinetochore Scaffold 1
GOPC	Golgi Associated PDZ And Coiled-Coil Motif Containing
FKBP5	FKBP Prolyl Isomerase 5
SLC6A2	Solute Carrier Family 6 Member 2
PINK1	PTEN Induced Kinase 1
ACTN3	Actinin Alpha 3
AQP9	Aquaporin 9
GDF6	Growth Differentiation Factor 6
PLEKHA1	Pleckstrin Homology Domain Containing A1
CHRNA4	Cholinergic Receptor Nicotinic Alpha 4 Subunit
IL37	Interleukin 37
NOL11	Nucleolar Protein 11
HEXB	Hexosaminidase Subunit Beta
MAPK9	Mitogen-Activated Protein Kinase 9
RPS15	Ribosomal Protein S15
CALCR	Calcitonin Receptor
ALDOA	Aldolase, Fructose-Bisphosphate A
UGT2B4	UDP Glucuronosyltransferase Family 2 Member B4
RBM8A	RNA Binding Motif Protein 8A
RALGDS	Ral Guanine Nucleotide Dissociation Stimulator
NADSYN1	NAD Synthetase 1
LMAN1	Lectin, Mannose Binding 1
ALOX15	Arachidonate 15-Lipoxygenase
TOMM40	Translocase Of Outer Mitochondrial Membrane 40
TOR1AIP1	Torsin 1A Interacting Protein 1
CHRNA7	Cholinergic Receptor Nicotinic Alpha 7 Subunit
SLC12A5	Solute Carrier Family 12 Member 5
APLNR	Apelin Receptor
PFKFB3	6-Phosphofructo-2-Kinase/Fructose-2,6-Biphosphatase 3
SEC22B	SEC22 Homolog B, Vesicle Trafficking Protein
TRIP10	Thyroid Hormone Receptor Interactor 10
CASP4	Caspase 4
CLINT1	Clathrin Interactor 1
SSR4	Signal Sequence Receptor Subunit 4
ADRB1	Adrenoceptor Beta 1
CITED4	Cbp/P300 Interacting Transactivator With Glu/Asp Rich Carboxy-Termin

CCNT1	Cyclin T1
GDF11	Growth Differentiation Factor 11
MCHR1	Melanin Concentrating Hormone Receptor 1
ANKK1	Ankyrin Repeat And Kinase Domain Containing 1
HNRNPC	Heterogeneous Nuclear Ribonucleoprotein C
IRAK4	Interleukin 1 Receptor Associated Kinase 4
RPLP0	Ribosomal Protein Lateral Stalk Subunit P0
CYC1	Cytochrome C1
HSPA1B	Heat Shock Protein Family A (Hsp70) Member 1B
CRISP3	Cysteine Rich Secretory Protein 3
IL15RA	Interleukin 15 Receptor Subunit Alpha
CWF19L2	CWF19 Like Cell Cycle Control Factor 2
MIR760	MicroRNA 760
HIC1	HIC ZBTB Transcriptional Repressor 1
PNLIPRP1	Pancreatic Lipase Related Protein 1
SP100	SP100 Nuclear Antigen
RPS13	Ribosomal Protein S13
LOC108863620	STAR 5' Regulatory Region
CLDN6	Claudin 6
PARD3	Par-3 Family Cell Polarity Regulator
TIMM50	Translocase Of Inner Mitochondrial Membrane 50
USP1	Ubiquitin Specific Peptidase 1
RAB11A	RAB11A, Member RAS Oncogene Family
TFDP1	Transcription Factor Dp-1
OAS3	2'-5'-Oligoadenylate Synthetase 3
IRF9	Interferon Regulatory Factor 9
CYP26A1	Cytochrome P450 Family 26 Subfamily A Member 1
SCARA3	Scavenger Receptor Class A Member 3
MIR208A	MicroRNA 208a
SNAP23	Synaptosome Associated Protein 23
A2M	Alpha-2-Macroglobulin
RSPO3	R-Spondin 3
TPPP3	Tubulin Polymerization Promoting Protein Family Member 3
DHRS4	Dehydrogenase/Reductase 4
RRAD	RRAD, Ras Related Glycolysis Inhibitor And Calcium Channel Regulator
TRB	T Cell Receptor Beta Locus
TGM3	Transglutaminase 3
ICAM3	Intercellular Adhesion Molecule 3
CNR2	Cannabinoid Receptor 2
INHBE	Inhibin Subunit Beta E
ALDH5A1	Aldehyde Dehydrogenase 5 Family Member A1
C7	Complement C7
PSMC1	Proteasome 26S Subunit, ATPase 1
MEOX1	Mesenchyme Homeobox 1
TNIP1	TNFAIP3 Interacting Protein 1
CSNK2A2	Casein Kinase 2 Alpha 2
FMO1	Flavin Containing Dimethylaniline Monoxygenase 1
RXFP1	Relaxin Family Peptide Receptor 1
ENHO	Energy Homeostasis Associated
STX6	Syntaxin 6
UPK1B	Uroplakin 1B
EGFL7	EGF Like Domain Multiple 7
LSP1	Lymphocyte Specific Protein 1
KMT5C	Lysine Methyltransferase 5C

AOX1	Aldehyde Oxidase 1
NCK1	NCK Adaptor Protein 1
UBA52	Ubiquitin A-52 Residue Ribosomal Protein Fusion Product 1
COX6A1	Cytochrome C Oxidase Subunit 6A1
CAMK2G	Calcium/Calmodulin Dependent Protein Kinase II Gamma
SLC20A1	Solute Carrier Family 20 Member 1
CCL8	C-C Motif Chemokine Ligand 8
POLR2H	RNA Polymerase II, I And III Subunit H
VTI1B	Vesicle Transport Through Interaction With T-SNAREs 1B
CDK5	Cyclin Dependent Kinase 5
LSM2	LSM2 Homolog, U6 Small Nuclear RNA And MRNA Degradation Associated
HMGCS2	3-Hydroxy-3-Methylglutaryl-CoA Synthase 2
UGT1A9	UDP Glucuronosyltransferase Family 1 Member A9
FAT1	FAT Atypical Cadherin 1
GOSR2	Golgi SNAP Receptor Complex Member 2
PRKAR2A	Protein Kinase CAMP-Dependent Type II Regulatory Subunit Alpha
CENPB	Centromere Protein B
ATP2C1	ATPase Secretory Pathway Ca2+ Transporting 1
IPO5	Importin 5
LRPPRC	Leucine Rich Pentatricopeptide Repeat Containing
PNPT1	Polyribonucleotide Nucleotidyltransferase 1
MCM3	Minichromosome Maintenance Complex Component 3
IFN1@	Interferon, Type 1, Cluster
ARID2	AT-Rich Interaction Domain 2
EIF4A2	Eukaryotic Translation Initiation Factor 4A2
LMO2	LIM Domain Only 2
PTK7	Protein Tyrosine Kinase 7 (Inactive)
ACR	Acrosin
TBC1D20	TBC1 Domain Family Member 20
KIF2A	Kinesin Family Member 2A
TLN1	Talin 1
AVPR1A	Arginine Vasopressin Receptor 1A
RPL32	Ribosomal Protein L32
LTA4H	Leukotriene A4 Hydrolase
SGIP1	SH3GL Interacting Endocytic Adaptor 1
NYAP2	Neuronal Tyrosine-Phosphorylated Phosphoinositide-3-Kinase Adaptor 2
CNTF	Ciliary Neurotrophic Factor
SLC5A6	Solute Carrier Family 5 Member 6
GABRB2	Gamma-Aminobutyric Acid Type A Receptor Subunit Beta2
EML1	EMAP Like 1
HRG	Histidine Rich Glycoprotein
INPP5D	Inositol Polyphosphate-5-Phosphatase D
IL22	Interleukin 22
OASL	2'-5'-Oligoadenylate Synthetase Like
NOLC1	Nucleolar And Coiled-Body Phosphoprotein 1
MGAT1	Alpha-1,3-Mannosyl-Glycoprotein 2-Beta-N-Acetylglucosaminyltransfera
GRM5	Glutamate Metabotropic Receptor 5
OSBP	Oxysterol Binding Protein
LOC109029530	HSD3B2 5' Regulatory Region
TRADD	TNFRSF1A Associated Via Death Domain
BRD9	Bromodomain Containing 9
LOXL3	Lysyl Oxidase Like 3
CHRNA5	Cholinergic Receptor Nicotinic Alpha 5 Subunit
RTN4	Reticulon 4

ACTN1	Actinin Alpha 1
ARHGAP18	Rho GTPase Activating Protein 18
CYSLTR2	Cysteinyl Leukotriene Receptor 2
CXorf56	Chromosome X Open Reading Frame 56
PTPRO	Protein Tyrosine Phosphatase Receptor Type O
ALYREF	Aly/REF Export Factor
CCL28	C-C Motif Chemokine Ligand 28
HMOX2	Heme Oxygenase 2
ABCC11	ATP Binding Cassette Subfamily C Member 11
TFCP2	Transcription Factor CP2
MAN2A1	Mannosidase Alpha Class 2A Member 1
ESYT1	Extended Synaptotagmin 1
FOXD1	Forkhead Box D1
CHRNB4	Cholinergic Receptor Nicotinic Beta 4 Subunit
ITK	IL2 Inducible T Cell Kinase
SMURF2	SMAD Specific E3 Ubiquitin Protein Ligase 2
MED15	Mediator Complex Subunit 15
NAT8	N-Acetyltransferase 8 (Putative)
MYO7A	Myosin VIIA
MFHAS1	Malignant Fibrous Histiocytoma Amplified Sequence 1
PIGN	Phosphatidylinositol Glycan Anchor Biosynthesis Class N
DLX2	Distal-Less Homeobox 2
SLC6A8	Solute Carrier Family 6 Member 8
HTR5A	5-Hydroxytryptamine Receptor 5A
MIR582	MicroRNA 582
LILRB1	Leukocyte Immunoglobulin Like Receptor B1
KAT2A	Lysine Acetyltransferase 2A
HDAC7	Histone Deacetylase 7
RBCK1	RANBP2-Type And C3HC4-Type Zinc Finger Containing 1
DNAJA3	DnaJ Heat Shock Protein Family (Hsp40) Member A3
LEFTY1	Left-Right Determination Factor 1
HECTD4	HECT Domain E3 Ubiquitin Protein Ligase 4
FMN2	Formin 2
LEPROTL1	Leptin Receptor Overlapping Transcript Like 1
MERTK	MER Proto-Oncogene, Tyrosine Kinase
PSME1	Proteasome Activator Subunit 1
FOLH1	Folate Hydrolase 1
RTRAF	RNA Transcription, Translation And Transport Factor
ENDOG	Endonuclease G
NEK4	NIMA Related Kinase 4
TAX1BP1	Tax1 Binding Protein 1
EBF2	EBF Transcription Factor 2
GDF3	Growth Differentiation Factor 3
GDF10	Growth Differentiation Factor 10
ADRA2B	Adrenoceptor Alpha 2B
ERRFI1	ERBB Receptor Feedback Inhibitor 1
SFPQ	Splicing Factor Proline And Glutamine Rich
ABCA7	ATP Binding Cassette Subfamily A Member 7
CRY2	Cryptochrome Circadian Regulator 2
DUX4	Double Homeobox 4
NDUFA4	NDUFA4 Mitochondrial Complex Associated
PTPN9	Protein Tyrosine Phosphatase Non-Receptor Type 9
ALDH1B1	Aldehyde Dehydrogenase 1 Family Member B1
CHRNA6	Cholinergic Receptor Nicotinic Alpha 6 Subunit

CHRNA9	Cholinergic Receptor Nicotinic Alpha 9 Subunit
BMP5	Bone Morphogenetic Protein 5
CHRNB3	Cholinergic Receptor Nicotinic Beta 3 Subunit
B3GNT5	UDP-GlcNAc:BetaGal Beta-1,3-N-Acetylglucosaminyltransferase 5
SLC2A12	Solute Carrier Family 2 Member 12
BMP8B	Bone Morphogenetic Protein 8b
GDF7	Growth Differentiation Factor 7
DOK5	Docking Protein 5
CHRNA10	Cholinergic Receptor Nicotinic Alpha 10 Subunit
GRK3	G Protein-Coupled Receptor Kinase 3
HIVEP1	HIVEP Zinc Finger 1
FGFR1OP2	FGFR1 Oncogene Partner 2
ZNF71	Zinc Finger Protein 71
TENT5A	Terminal Nucleotidyltransferase 5A
CFAP94	Cilia And Flagella Associated Protein 94
HSD17B10	Hydroxysteroid 17-Beta Dehydrogenase 10
ORC2	Origin Recognition Complex Subunit 2
SNRPD1	Small Nuclear Ribonucleoprotein D1 Polypeptide
CYB5B	Cytochrome B5 Type B
STX5	Syntaxin 5
STX8	Syntaxin 8
TMEM230	Transmembrane Protein 230
TMEM201	Transmembrane Protein 201
RUSF1	RUS Family Member 1
RPS6KA5	Ribosomal Protein S6 Kinase A5
SMARCC2	SWI/SNF Related, Matrix Associated, Actin Dependent Regulator Of Chr
SEL1L	SEL1L Adaptor Subunit Of ERAD E3 Ubiquitin Ligase
TMEM147	Transmembrane Protein 147
PPP1CA	Protein Phosphatase 1 Catalytic Subunit Alpha
EIF4B	Eukaryotic Translation Initiation Factor 4B
UGT1A	UDP Glucuronosyltransferase Family 1 Member A Complex Locus
GTF3A	General Transcription Factor IIIA
FZD2	Frizzled Class Receptor 2
USP4	Ubiquitin Specific Peptidase 4
SYVN1	Synoviolin 1
ANO6	Anoctamin 6
KPNA6	Karyopherin Subunit Alpha 6
NCBP1	Nuclear Cap Binding Protein Subunit 1
DAB2IP	DAB2 Interacting Protein
PPP1R14A	Protein Phosphatase 1 Regulatory Inhibitor Subunit 14A
NONO	Non-POU Domain Containing Octamer Binding
BRAT1	BRCA1 Associated ATM Activator 1
MMP25	Matrix Metallopeptidase 25
ADRA2C	Adrenoceptor Alpha 2C
CDC34	Cell Division Cycle 34, Ubiqiutin Conjugating Enzyme
COPS2	COP9 Signalosome Subunit 2
FGF20	Fibroblast Growth Factor 20
UGGT2	UDP-Glucose Glycoprotein Glucosyltransferase 2
SPRR1A	Small Proline Rich Protein 1A
ANAPC10	Anaphase Promoting Complex Subunit 10
KPNA1	Karyopherin Subunit Alpha 1
PICALM	Phosphatidylinositol Binding Clathrin Assembly Protein
TNFAIP8L2	TNF Alpha Induced Protein 8 Like 2
SLC25A11	Solute Carrier Family 25 Member 11

PAFAH1B2	Platelet Activating Factor Acetylhydrolase 1b Catalytic Subunit 2
RANBP9	RAN Binding Protein 9
MAPK11	Mitogen-Activated Protein Kinase 11
TRIM22	Tripartite Motif Containing 22
SYNGR1	Synaptogyrin 1
MLC1	Modulator Of VRAC Current 1
USO1	USO1 Vesicle Transport Factor
RRBP1	Ribosome Binding Protein 1
EMC1	ER Membrane Protein Complex Subunit 1
KPNA3	Karyopherin Subunit Alpha 3
ZC3HAV1	Zinc Finger CCCH-Type Containing, Antiviral 1
GTF2B	General Transcription Factor IIB
CUL2	Cullin 2
OS9	OS9 Endoplasmic Reticulum Lectin
EDEM1	ER Degradation Enhancing Alpha-Mannosidase Like Protein 1
LCP2	Lymphocyte Cytosolic Protein 2
TSPAN7	Tetraspanin 7
LOC109504728	Chromosome 9 Open Reading Frame 72 Repeat Instability Region
MBD1	Methyl-CpG Binding Domain Protein 1
DDX20	DEAD-Box Helicase 20
TRIM13	Tripartite Motif Containing 13
PDZD8	PDZ Domain Containing 8
AAVS1	Adeno-Associated Virus Integration Site 1

Category	Gifts	GC Id	Relevan	GeneCards Link
Protein Coding	49	GC13P032315	151.54	https://www.genecards.org/cgi-bin/ca
Protein Coding	50	GC17M043044	115.36	https://www.genecards.org/cgi-bin/ca
Protein Coding	50	GC09M124481	79.61	https://www.genecards.org/cgi-bin/ca
Protein Coding	36	GC0XM085277	73.95	https://www.genecards.org/cgi-bin/ca
Protein Coding	54	GC17M007661	73.52	https://www.genecards.org/cgi-bin/ca
Protein Coding	41	GC0XP050910	72.48	https://www.genecards.org/cgi-bin/ca
Protein Coding	40	GC03M138944	68.81	https://www.genecards.org/cgi-bin/ca
Protein Coding	49	GC02M048953	67.84	https://www.genecards.org/cgi-bin/ca
Protein Coding	35	GC07M144397	65.32	https://www.genecards.org/cgi-bin/ca
Protein Coding	48	GC11M002159	63.42	https://www.genecards.org/cgi-bin/ca
Protein Coding	52	GC10P087863	62.41	https://www.genecards.org/cgi-bin/ca
Protein Coding	44	GC0XP147913	62	https://www.genecards.org/cgi-bin/ca
Protein Coding	47	GC08M089933	59.35	https://www.genecards.org/cgi-bin/ca
Protein Coding	41	GC07P100177	58.85	https://www.genecards.org/cgi-bin/ca
Protein Coding	48	GC02M025160	58.62	https://www.genecards.org/cgi-bin/ca
Protein Coding	36	GC02M070741	58.55	https://www.genecards.org/cgi-bin/ca
Protein Coding	48	GC15M074337	57.5	https://www.genecards.org/cgi-bin/ca
Protein Coding	54	GC14M104769	56.94	https://www.genecards.org/cgi-bin/ca
Protein Coding	45	GC10M049454	56.33	https://www.genecards.org/cgi-bin/ca
Protein Coding	50	GC07P022765	56.21	https://www.genecards.org/cgi-bin/ca
Protein Coding	46	GC0XM030304	55.18	https://www.genecards.org/cgi-bin/ca
Protein Coding	53	GC06P151656	54.51	https://www.genecards.org/cgi-bin/ca
Protein Coding	48	GC03P189598	54.44	https://www.genecards.org/cgi-bin/ca
Protein Coding	47	GC01P156082	54.01	https://www.genecards.org/cgi-bin/ca
Protein Coding	39	GC05M132861	53.71	https://www.genecards.org/cgi-bin/ca
Protein Coding	48	GC15M051208	52.09	https://www.genecards.org/cgi-bin/ca
Protein Coding	49	GC11M032365	51.88	https://www.genecards.org/cgi-bin/ca
Protein Coding	53	GC03P041236	50.93	https://www.genecards.org/cgi-bin/ca
Protein Coding	48	GC10M102830	50.25	https://www.genecards.org/cgi-bin/ca
Protein Coding	39	GC01M091260	50.15	https://www.genecards.org/cgi-bin/ca
Protein Coding	40	GC20P005963	49.68	https://www.genecards.org/cgi-bin/ca
Protein Coding	49	GC14M064084	49.67	https://www.genecards.org/cgi-bin/ca
Protein Coding	50	GC04P073397	49.03	https://www.genecards.org/cgi-bin/ca
Protein Coding	43	GC19P002251	48.96	https://www.genecards.org/cgi-bin/ca
Protein Coding	43	GC0XP096684	48.62	https://www.genecards.org/cgi-bin/ca
Protein Coding	46	GC08M038145	48.28	https://www.genecards.org/cgi-bin/ca
Protein Coding	47	GC09P034860	48.19	https://www.genecards.org/cgi-bin/ca
Protein Coding	44	GC14P075002	47.63	https://www.genecards.org/cgi-bin/ca
Protein Coding	54	GC10M121478	47.6	https://www.genecards.org/cgi-bin/ca
Protein Coding	54	GC11P108222	46.46	https://www.genecards.org/cgi-bin/ca
Protein Coding	51	GC05M001253	46.28	https://www.genecards.org/cgi-bin/ca
Protein Coding	45	GC12P053423	45.19	https://www.genecards.org/cgi-bin/ca
Protein Coding	47	GC01M206767	44.93	https://www.genecards.org/cgi-bin/ca
Protein Coding	55	GC08M038400	44.44	https://www.genecards.org/cgi-bin/ca
Protein Coding	39	GC06M118813	44.32	https://www.genecards.org/cgi-bin/ca
Protein Coding	47	GC02M048647	43.82	https://www.genecards.org/cgi-bin/ca
Protein Coding	45	GC15M089316	43.03	https://www.genecards.org/cgi-bin/ca
Protein Coding	39	GC17M042572	42.79	https://www.genecards.org/cgi-bin/ca
Protein Coding	51	GC12M025204	42.5	https://www.genecards.org/cgi-bin/ca
Protein Coding	37	GC10M133553	42.44	https://www.genecards.org/cgi-bin/ca
RNA Gene	22	GC05P160485	41.97	https://www.genecards.org/cgi-bin/ca
Protein Coding	41	GC06P047322	41.92	https://www.genecards.org/cgi-bin/ca
Protein Coding	52	GC19M041301	41.73	https://www.genecards.org/cgi-bin/ca

Protein Coding	39	GC09P135561	40.97	https://www.genecards.org/cgi-bin/ca
Protein Coding	45	GC03P045405	40.95	https://www.genecards.org/cgi-bin/ca
Protein Coding	44	GC21P044285	40.9	https://www.genecards.org/cgi-bin/ca
Protein Coding	52	GC03P179148	40.6	https://www.genecards.org/cgi-bin/ca
Protein Coding	44	GC02M027364	40.42	https://www.genecards.org/cgi-bin/ca
Protein Coding	41	GC11M094444	40.36	https://www.genecards.org/cgi-bin/ca
Protein Coding	53	GC16P055390	40.08	https://www.genecards.org/cgi-bin/ca
Protein Coding	54	GC17P039687	39.84	https://www.genecards.org/cgi-bin/ca
Protein Coding	46	GC05P119452	39.7	https://www.genecards.org/cgi-bin/ca
Protein Coding	47	GC17P072121	39.55	https://www.genecards.org/cgi-bin/ca
Protein Coding	50	GC10P088969	39.54	https://www.genecards.org/cgi-bin/ca
Protein Coding	44	GC17M037686	39.4	https://www.genecards.org/cgi-bin/ca
Protein Coding	51	GC03P030623	39.15	https://www.genecards.org/cgi-bin/ca
Protein Coding	48	GC11M002130	38.71	https://www.genecards.org/cgi-bin/ca
Protein Coding	51	GC09P117704	38.66	https://www.genecards.org/cgi-bin/ca
Protein Coding	54	GC07M140719	38.48	https://www.genecards.org/cgi-bin/ca
Protein Coding	42	GC14P045135	38.05	https://www.genecards.org/cgi-bin/ca
Protein Coding	44	GC12M088492	37.9	https://www.genecards.org/cgi-bin/ca
Protein Coding	45	GC06P047333	37.88	https://www.genecards.org/cgi-bin/ca
Protein Coding	45	GC07M019020	37.83	https://www.genecards.org/cgi-bin/ca
Protein Coding	50	GC12M102395	37.81	https://www.genecards.org/cgi-bin/ca
Protein Coding	50	GC19P044906	37.63	https://www.genecards.org/cgi-bin/ca
Protein Coding	43	GC17P058692	37.53	https://www.genecards.org/cgi-bin/ca
Protein Coding	51	GC04P109912	37.46	https://www.genecards.org/cgi-bin/ca
Protein Coding	44	GC06P167111	37.35	https://www.genecards.org/cgi-bin/ca
Protein Coding	51	GC09M021967	37.27	https://www.genecards.org/cgi-bin/ca
Protein Coding	54	GC01M011106	36.97	https://www.genecards.org/cgi-bin/ca
Protein Coding	48	GC08P011676	36.96	https://www.genecards.org/cgi-bin/ca
Protein Coding	51	GC08P127735	36.54	https://www.genecards.org/cgi-bin/ca
Protein Coding	52	GC15P040694	36.43	https://www.genecards.org/cgi-bin/ca
Protein Coding	45	GC07M041668	36	https://www.genecards.org/cgi-bin/ca
Protein Coding	48	GC01M204154	35.93	https://www.genecards.org/cgi-bin/ca
Protein Coding	51	GC13M028300	35.88	https://www.genecards.org/cgi-bin/ca
Protein Coding	47	GC01M022190	35.82	https://www.genecards.org/cgi-bin/ca
Protein Coding	49	GC05P132556	35.73	https://www.genecards.org/cgi-bin/ca
Protein Coding	43	GC11P030210	35.67	https://www.genecards.org/cgi-bin/ca
Protein Coding	54	GC07P055019	35.6	https://www.genecards.org/cgi-bin/ca
Protein Coding	52	GC09P099104	35.58	https://www.genecards.org/cgi-bin/ca
Protein Coding	48	GC12M068064	35.37	https://www.genecards.org/cgi-bin/ca
Protein Coding	55	GC04P001795	35.12	https://www.genecards.org/cgi-bin/ca
Protein Coding	48	GC03P036993	35.05	https://www.genecards.org/cgi-bin/ca
Protein Coding	41	GC02M044281	34.99	https://www.genecards.org/cgi-bin/ca
Protein Coding	52	GC11M000635	34.87	https://www.genecards.org/cgi-bin/ca
Protein Coding	55	GC05M150113	34.78	https://www.genecards.org/cgi-bin/ca
Protein Coding	51	GC09M136602	34.75	https://www.genecards.org/cgi-bin/ca
Protein Coding	47	GC01P172628	34.67	https://www.genecards.org/cgi-bin/ca
Protein Coding	51	GC11M102810	34.56	https://www.genecards.org/cgi-bin/ca
Protein Coding	52	GC12P068808	34.55	https://www.genecards.org/cgi-bin/ca
Protein Coding	45	GC08P031033	34.54	https://www.genecards.org/cgi-bin/ca
Protein Coding	46	GC01P119414	34.45	https://www.genecards.org/cgi-bin/ca
Protein Coding	44	GC19P000917	34.33	https://www.genecards.org/cgi-bin/ca
Protein Coding	35	GC09M135693	34.32	https://www.genecards.org/cgi-bin/ca
Protein Coding	48	GC15P090717	34.23	https://www.genecards.org/cgi-bin/ca
Protein Coding	45	GC15M048408	33.9	https://www.genecards.org/cgi-bin/ca

Protein Coding	41	GC03P139005	33.89	https://www.genecards.org/cgi-bin/ca
Protein Coding	44	GC19P006389	33.74	https://www.genecards.org/cgi-bin/ca
Protein Coding	48	GC17P031094	33.51	https://www.genecards.org/cgi-bin/ca
Protein Coding	43	GC12P068686	33.44	https://www.genecards.org/cgi-bin/ca
Protein Coding	46	GC04P087975	33.35	https://www.genecards.org/cgi-bin/ca
Protein Coding	51	GC06P047305	33.03	https://www.genecards.org/cgi-bin/ca
Protein Coding	29	GC02P200511	32.97	https://www.genecards.org/cgi-bin/ca
Protein Coding	49	GC15P067063	32.69	https://www.genecards.org/cgi-bin/ca
Protein Coding	41	GC08M025419	32.66	https://www.genecards.org/cgi-bin/ca
Protein Coding	50	GC14P075278	32.47	https://www.genecards.org/cgi-bin/ca
Protein Coding	45	GC19M045409	32.34	https://www.genecards.org/cgi-bin/ca
Protein Coding	50	GC02M215360	32.26	https://www.genecards.org/cgi-bin/ca
Protein Coding	48	GC06P043770	32.24	https://www.genecards.org/cgi-bin/ca
Protein Coding	50	GC18P051028	31.91	https://www.genecards.org/cgi-bin/ca
Protein Coding	47	GC14M075958	31.65	https://www.genecards.org/cgi-bin/ca
Protein Coding	50	GC11M100943	31.58	https://www.genecards.org/cgi-bin/ca
Protein Coding	41	GC0XP148500	31.45	https://www.genecards.org/cgi-bin/ca
Protein Coding	49	GC14M053949	31.34	https://www.genecards.org/cgi-bin/ca
Protein Coding	52	GC03P012287	30.95	https://www.genecards.org/cgi-bin/ca
Protein Coding	47	GC20P006696	30.78	https://www.genecards.org/cgi-bin/ca
RNA Gene	16	GC17M029965	30.76	https://www.genecards.org/cgi-bin/ca
Protein Coding	53	GC22M028687	30.73	https://www.genecards.org/cgi-bin/ca
Protein Coding	44	GC05M122063	30.68	https://www.genecards.org/cgi-bin/ca
Protein Coding	51	GC22M021754	30.68	https://www.genecards.org/cgi-bin/ca
RNA Gene	24	GC17P059841	30.51	https://www.genecards.org/cgi-bin/ca
Protein Coding	53	GC04P054657	30.46	https://www.genecards.org/cgi-bin/ca
Protein Coding	48	GC07P116524	30.45	https://www.genecards.org/cgi-bin/ca
Protein Coding	51	GC14P025277	30.43	https://www.genecards.org/cgi-bin/ca
RNA Gene	28	GC11M001995	30.33	https://www.genecards.org/cgi-bin/ca
Protein Coding	45	GC07M045912	30.26	https://www.genecards.org/cgi-bin/ca
Protein Coding	50	GC01P218345	30.21	https://www.genecards.org/cgi-bin/ca
Protein Coding	52	GC02P201233	30.15	https://www.genecards.org/cgi-bin/ca
Protein Coding	50	GC06P121436	29.82	https://www.genecards.org/cgi-bin/ca
Protein Coding	49	GC19P001177	29.5	https://www.genecards.org/cgi-bin/ca
Protein Coding	50	GC16P068737	29.35	https://www.genecards.org/cgi-bin/ca
Protein Coding	43	GC02P219569	29.35	https://www.genecards.org/cgi-bin/ca
Protein Coding	53	GC02M190908	28.92	https://www.genecards.org/cgi-bin/ca
Protein Coding	36	GC15M083255	28.81	https://www.genecards.org/cgi-bin/ca
Protein Coding	49	GC13P048303	28.6	https://www.genecards.org/cgi-bin/ca
Protein Coding	50	GC20P058839	28.52	https://www.genecards.org/cgi-bin/ca
Protein Coding	42	GC06M044297	28.49	https://www.genecards.org/cgi-bin/ca
Protein Coding	51	GC20P037344	28.19	https://www.genecards.org/cgi-bin/ca
Protein Coding	44	GC06M022230	28.12	https://www.genecards.org/cgi-bin/ca
Protein Coding	51	GC03M142449	28.02	https://www.genecards.org/cgi-bin/ca
Protein Coding	53	GC0XP067544	27.6	https://www.genecards.org/cgi-bin/ca
Protein Coding	47	GC19M045349	27.38	https://www.genecards.org/cgi-bin/ca
Protein Coding	52	GC11M001752	27.3	https://www.genecards.org/cgi-bin/ca
Protein Coding	38	GC04P039184	27.17	https://www.genecards.org/cgi-bin/ca
Protein Coding	54	GC19M007112	27.13	https://www.genecards.org/cgi-bin/ca
Protein Coding	54	GC03M012583	26.82	https://www.genecards.org/cgi-bin/ca
RNA Gene	21	GC13P091350	26.81	https://www.genecards.org/cgi-bin/ca
Protein Coding	44	GC07P074027	26.7	https://www.genecards.org/cgi-bin/ca
Protein Coding	44	GC08M048854	26.64	https://www.genecards.org/cgi-bin/ca
Protein Coding	54	GC09P004985	26.52	https://www.genecards.org/cgi-bin/ca

Protein Coding	41	GC04P073740	26.42	https://www.genecards.org/cgi-bin/ca
Protein Coding	52	GC04P102501	26.37	https://www.genecards.org/cgi-bin/ca
RNA Gene	22	GC09P136670	26.29	https://www.genecards.org/cgi-bin/ca
RNA Gene	18	GC21P025573	26.26	https://www.genecards.org/cgi-bin/ca
Protein Coding	40	GC17P007613	26.16	https://www.genecards.org/cgi-bin/ca
Protein Coding	48	GC07P075899	26.15	https://www.genecards.org/cgi-bin/ca
Protein Coding	55	GC04P054229	26.13	https://www.genecards.org/cgi-bin/ca
Protein Coding	35	GC0YM002698	26.01	https://www.genecards.org/cgi-bin/ca
Protein Coding	43	GC01P040258	25.87	https://www.genecards.org/cgi-bin/ca
RNA Gene	18	GC11M002188	25.82	https://www.genecards.org/cgi-bin/ca
Protein Coding	47	GC02M226731	25.79	https://www.genecards.org/cgi-bin/ca
Protein Coding	49	GC17P047254	25.72	https://www.genecards.org/cgi-bin/ca
Protein Coding	48	GC05P179806	25.63	https://www.genecards.org/cgi-bin/ca
Protein Coding	54	GC15P066386	25.53	https://www.genecards.org/cgi-bin/ca
Protein Coding	54	GC07P116672	25.47	https://www.genecards.org/cgi-bin/ca
Protein Coding	46	GC09M127815	25.43	https://www.genecards.org/cgi-bin/ca
Protein Coding	51	GC05P068215	25.38	https://www.genecards.org/cgi-bin/ca
Protein Coding	40	GC17M035092	25.34	https://www.genecards.org/cgi-bin/ca
Protein Coding	49	GC15P040161	25.33	https://www.genecards.org/cgi-bin/ca
Protein Coding	47	GC10M101770	25.32	https://www.genecards.org/cgi-bin/ca
Protein Coding	48	GC05P112707	25.24	https://www.genecards.org/cgi-bin/ca
Protein Coding	48	GC07M041960	25.19	https://www.genecards.org/cgi-bin/ca
Protein Coding	42	GC14P054398	25.17	https://www.genecards.org/cgi-bin/ca
Protein Coding	46	GC06P047265	25.1	https://www.genecards.org/cgi-bin/ca
RNA Gene	29	GC14P104771	25.02	https://www.genecards.org/cgi-bin/ca
Protein Coding	48	GC02M112829	24.87	https://www.genecards.org/cgi-bin/ca
Protein Coding	47	GC07P128241	24.86	https://www.genecards.org/cgi-bin/ca
Protein Coding	50	GC11P125625	24.73	https://www.genecards.org/cgi-bin/ca
Protein Coding	48	GC02P219418	24.69	https://www.genecards.org/cgi-bin/ca
Protein Coding	47	GC18M017331	24.57	https://www.genecards.org/cgi-bin/ca
Protein Coding	39	GC06M131948	24.51	https://www.genecards.org/cgi-bin/ca
Protein Coding	54	GC19M040230	24.49	https://www.genecards.org/cgi-bin/ca
RNA Gene	21	GC0XP066018	24.48	https://www.genecards.org/cgi-bin/ca
Protein Coding	50	GC05P171387	24.47	https://www.genecards.org/cgi-bin/ca
Protein Coding	52	GC01M042925	24.44	https://www.genecards.org/cgi-bin/ca
Protein Coding	46	GC01M159716	24.33	https://www.genecards.org/cgi-bin/ca
Protein Coding	54	GC12M057743	24.17	https://www.genecards.org/cgi-bin/ca
Protein Coding	51	GC07P117287	24.17	https://www.genecards.org/cgi-bin/ca
Protein Coding	50	GC09M095442	24.08	https://www.genecards.org/cgi-bin/ca
RNA Gene	22	GC19M014010	24.06	https://www.genecards.org/cgi-bin/ca
Protein Coding	46	GC03M049359	23.84	https://www.genecards.org/cgi-bin/ca
Protein Coding	52	GC20P046008	23.84	https://www.genecards.org/cgi-bin/ca
RNA Gene	22	GC16P069934	23.81	https://www.genecards.org/cgi-bin/ca
Protein Coding	42	GC11M022600	23.73	https://www.genecards.org/cgi-bin/ca
Protein Coding	33	GC10P100993	23.73	https://www.genecards.org/cgi-bin/ca
Protein Coding	47	GC16M000287	23.71	https://www.genecards.org/cgi-bin/ca
Protein Coding	47	GC16P008788	23.63	https://www.genecards.org/cgi-bin/ca
Protein Coding	48	GC01P156786	23.6	https://www.genecards.org/cgi-bin/ca
RNA Gene	21	GC11M000674	23.5	https://www.genecards.org/cgi-bin/ca
Protein Coding	47	GC04M067737	23.4	https://www.genecards.org/cgi-bin/ca
Protein Coding	44	GC06P108559	23.33	https://www.genecards.org/cgi-bin/ca
Protein Coding	48	GC08M142872	23.3	https://www.genecards.org/cgi-bin/ca
Protein Coding	47	GC06P047549	23.23	https://www.genecards.org/cgi-bin/ca
Protein Coding	44	GC12P065824	23.13	https://www.genecards.org/cgi-bin/ca

RNA Gene	28	GC03M169765	23.05	https://www.genecards.org/cgi-bin/ca
Protein Coding	46	GC05P053480	23.03	https://www.genecards.org/cgi-bin/ca
Protein Coding	47	GC11M013492	23	https://www.genecards.org/cgi-bin/ca
Protein Coding	45	GC0XP077927	22.92	https://www.genecards.org/cgi-bin/ca
Protein Coding	49	GC11M128458	22.91	https://www.genecards.org/cgi-bin/ca
Protein Coding	45	GC02P203867	22.87	https://www.genecards.org/cgi-bin/ca
Protein Coding	44	GC07M074231	22.81	https://www.genecards.org/cgi-bin/ca
Protein Coding	47	GC05P042429	22.78	https://www.genecards.org/cgi-bin/ca
RNA Gene	22	GC01M009151	22.62	https://www.genecards.org/cgi-bin/ca
Protein Coding	43	GC06M087085	22.41	https://www.genecards.org/cgi-bin/ca
RNA Gene	20	GC14P104580	22.39	https://www.genecards.org/cgi-bin/ca
Protein Coding	47	GC09M095099	22.37	https://www.genecards.org/cgi-bin/ca
Protein Coding	43	GC16M023603	22.31	https://www.genecards.org/cgi-bin/ca
Protein Coding	47	GC06M031184	22.22	https://www.genecards.org/cgi-bin/ca
RNA Gene	20	GC17M001713	22.21	https://www.genecards.org/cgi-bin/ca
Protein Coding	46	GC01P207752	22.11	https://www.genecards.org/cgi-bin/ca
Protein Coding	47	GC05P174724	22.1	https://www.genecards.org/cgi-bin/ca
Protein Coding	47	GC04P122826	22.08	https://www.genecards.org/cgi-bin/ca
Protein Coding	44	GC14M091869	22.07	https://www.genecards.org/cgi-bin/ca
Protein Coding	26	GC01M149961	22.03	https://www.genecards.org/cgi-bin/ca
Protein Coding	47	GC14P061695	21.98	https://www.genecards.org/cgi-bin/ca
Protein Coding	50	GC11P034460	21.94	https://www.genecards.org/cgi-bin/ca
Protein Coding	40	GC17P040015	21.89	https://www.genecards.org/cgi-bin/ca
RNA Gene	20	GC0XM045746	21.89	https://www.genecards.org/cgi-bin/ca
Protein Coding	45	GC04P073431	21.87	https://www.genecards.org/cgi-bin/ca
Protein Coding	50	GC01P021508	21.86	https://www.genecards.org/cgi-bin/ca
Protein Coding	48	GC02P001374	21.77	https://www.genecards.org/cgi-bin/ca
Protein Coding	47	GC11M002887	21.67	https://www.genecards.org/cgi-bin/ca
Protein Coding	43	GC03P184135	21.65	https://www.genecards.org/cgi-bin/ca
Protein Coding	47	GC01M155185	21.54	https://www.genecards.org/cgi-bin/ca
Protein Coding	46	GC06M032578	21.54	https://www.genecards.org/cgi-bin/ca
Protein Coding	41	GC08M144512	21.47	https://www.genecards.org/cgi-bin/ca
Protein Coding	40	GC12M088049	21.45	https://www.genecards.org/cgi-bin/ca
Protein Coding	46	GC11M064803	21.42	https://www.genecards.org/cgi-bin/ca
Protein Coding	45	GC04M122451	21.32	https://www.genecards.org/cgi-bin/ca
Protein Coding	49	GC17P063477	21.31	https://www.genecards.org/cgi-bin/ca
Protein Coding	40	GC18M059430	21.13	https://www.genecards.org/cgi-bin/ca
Protein Coding	50	GC07P101127	21.08	https://www.genecards.org/cgi-bin/ca
Protein Coding	48	GC12M005917	20.92	https://www.genecards.org/cgi-bin/ca
Protein Coding	50	GC07M155799	20.83	https://www.genecards.org/cgi-bin/ca
Protein Coding	47	GC19P038528	20.83	https://www.genecards.org/cgi-bin/ca
Protein Coding	46	GC15P066702	20.75	https://www.genecards.org/cgi-bin/ca
Protein Coding	54	GC15P098648	20.73	https://www.genecards.org/cgi-bin/ca
Protein Coding	35	GC17P077282	20.67	https://www.genecards.org/cgi-bin/ca
Protein Coding	48	GC12M047972	20.61	https://www.genecards.org/cgi-bin/ca
Protein Coding	50	GC04M184627	20.45	https://www.genecards.org/cgi-bin/ca
Protein Coding	44	GC06P156777	20.28	https://www.genecards.org/cgi-bin/ca
RNA Gene	21	GC09P094178	20.06	https://www.genecards.org/cgi-bin/ca
Protein Coding	53	GC04M055078	20	https://www.genecards.org/cgi-bin/ca
Protein Coding	48	GC09M132891	19.95	https://www.genecards.org/cgi-bin/ca
Protein Coding	52	GC11P069641	19.95	https://www.genecards.org/cgi-bin/ca
Protein Coding	47	GC06P012290	19.94	https://www.genecards.org/cgi-bin/ca
Protein Coding	50	GC22P041091	19.88	https://www.genecards.org/cgi-bin/ca
Protein Coding	46	GC07M097854	19.84	https://www.genecards.org/cgi-bin/ca

Protein Coding	41	GC01M204190	19.83	https://www.genecards.org/cgi-bin/ca
Protein Coding	52	GC17M042313	19.82	https://www.genecards.org/cgi-bin/ca
Protein Coding	41	GC01P202193	19.82	https://www.genecards.org/cgi-bin/ca
Protein Coding	41	GC12M008637	19.82	https://www.genecards.org/cgi-bin/ca
Protein Coding	50	GC06P047460	19.72	https://www.genecards.org/cgi-bin/ca
Protein Coding	49	GC10P067884	19.71	https://www.genecards.org/cgi-bin/ca
Protein Coding	50	GC16P002436	19.63	https://www.genecards.org/cgi-bin/ca
Protein Coding	53	GC10P043081	19.59	https://www.genecards.org/cgi-bin/ca
Protein Coding	48	GC02P047402	19.55	https://www.genecards.org/cgi-bin/ca
Protein Coding	50	GC19P010932	19.55	https://www.genecards.org/cgi-bin/ca
Protein Coding	48	GC07M005973	19.46	https://www.genecards.org/cgi-bin/ca
Protein Coding	50	GC01M119911	19.44	https://www.genecards.org/cgi-bin/ca
Protein Coding	41	GC20P019759	19.43	https://www.genecards.org/cgi-bin/ca
Protein Coding	45	GC0XP047583	19.4	https://www.genecards.org/cgi-bin/ca
Protein Coding	49	GC01M226360	19.35	https://www.genecards.org/cgi-bin/ca
Protein Coding	46	GC0XP154762	19.25	https://www.genecards.org/cgi-bin/ca
Protein Coding	43	GC05M147825	19.22	https://www.genecards.org/cgi-bin/ca
Protein Coding	37	GC14P024171	19.13	https://www.genecards.org/cgi-bin/ca
Protein Coding	48	GC11P068298	19.11	https://www.genecards.org/cgi-bin/ca
Protein Coding	42	GC11M063438	19.08	https://www.genecards.org/cgi-bin/ca
Protein Coding	46	GC10P100735	19.07	https://www.genecards.org/cgi-bin/ca
Protein Coding	39	GC16M001569	19.06	https://www.genecards.org/cgi-bin/ca
Protein Coding	40	GC03P129440	19.06	https://www.genecards.org/cgi-bin/ca
Protein Coding	51	GC07M087504	19.03	https://www.genecards.org/cgi-bin/ca
Protein Coding	46	GC01M113813	19.03	https://www.genecards.org/cgi-bin/ca
Protein Coding	50	GC19P010270	18.98	https://www.genecards.org/cgi-bin/ca
Protein Coding	42	GC08P132866	18.94	https://www.genecards.org/cgi-bin/ca
RNA Gene	19	GC11M072615	18.94	https://www.genecards.org/cgi-bin/ca
Protein Coding	47	GC02M178525	18.92	https://www.genecards.org/cgi-bin/ca
Protein Coding	43	GC07P114086	18.89	https://www.genecards.org/cgi-bin/ca
Protein Coding	50	GC03P122183	18.86	https://www.genecards.org/cgi-bin/ca
Protein Coding	48	GC10M088935	18.86	https://www.genecards.org/cgi-bin/ca
Protein Coding	44	GC0XM140502	18.85	https://www.genecards.org/cgi-bin/ca
Protein Coding	48	GC09M035056	18.81	https://www.genecards.org/cgi-bin/ca
Protein Coding	48	GC16M002275	18.7	https://www.genecards.org/cgi-bin/ca
Protein Coding	38	GC02M165905	18.69	https://www.genecards.org/cgi-bin/ca
Protein Coding	47	GC17M061679	18.63	https://www.genecards.org/cgi-bin/ca
Protein Coding	53	GC12P112418	18.63	https://www.genecards.org/cgi-bin/ca
Protein Coding	43	GC02M110122	18.6	https://www.genecards.org/cgi-bin/ca
Protein Coding	49	GC18P031557	18.58	https://www.genecards.org/cgi-bin/ca
Protein Coding	40	GC01M005863	18.52	https://www.genecards.org/cgi-bin/ca
Protein Coding	46	GC05P132673	18.52	https://www.genecards.org/cgi-bin/ca
Protein Coding	47	GC0XP030671	18.48	https://www.genecards.org/cgi-bin/ca
Protein Coding	43	GC06P047777	18.45	https://www.genecards.org/cgi-bin/ca
Protein Coding	39	GC17P028854	18.44	https://www.genecards.org/cgi-bin/ca
Protein Coding	44	GC02M112773	18.42	https://www.genecards.org/cgi-bin/ca
Protein Coding	43	GC07P046552	18.36	https://www.genecards.org/cgi-bin/ca
Protein Coding	44	GC17M078852	18.31	https://www.genecards.org/cgi-bin/ca
RNA Gene	21	GC21P016551	18.29	https://www.genecards.org/cgi-bin/ca
Protein Coding	48	GC01M186640	18.29	https://www.genecards.org/cgi-bin/ca
Protein Coding	47	GC02M219054	18.25	https://www.genecards.org/cgi-bin/ca
Protein Coding	44	GC05P132073	18.23	https://www.genecards.org/cgi-bin/ca
Protein Coding	41	GC12P052232	18.19	https://www.genecards.org/cgi-bin/ca
Protein Coding	51	GC06M159669	18.18	https://www.genecards.org/cgi-bin/ca

Protein Coding	35	GC20M045541	18.18	https://www.genecards.org/cgi-bin/ca
Protein Coding	45	GC0XP153724	18.15	https://www.genecards.org/cgi-bin/ca
Protein Coding	39	GC03M132683	18.13	https://www.genecards.org/cgi-bin/ca
Protein Coding	41	GC0XP013734	18.1	https://www.genecards.org/cgi-bin/ca
Protein Coding	44	GC05M173232	18.09	https://www.genecards.org/cgi-bin/ca
Protein Coding	44	GC0XP119236	18.08	https://www.genecards.org/cgi-bin/ca
Protein Coding	47	GC02M222199	18.08	https://www.genecards.org/cgi-bin/ca
Protein Coding	42	GC02P120349	18.03	https://www.genecards.org/cgi-bin/ca
Protein Coding	38	GC0XP000624	18.02	https://www.genecards.org/cgi-bin/ca
Protein Coding	47	GC05M044340	18	https://www.genecards.org/cgi-bin/ca
Protein Coding	44	GC17M063917	17.97	https://www.genecards.org/cgi-bin/ca
Protein Coding	50	GC02P047695	17.96	https://www.genecards.org/cgi-bin/ca
Protein Coding	42	GC06P052186	17.94	https://www.genecards.org/cgi-bin/ca
Protein Coding	48	GC11M116835	17.91	https://www.genecards.org/cgi-bin/ca
Protein Coding	43	GC19P007669	17.91	https://www.genecards.org/cgi-bin/ca
Protein Coding	42	GC01M044850	17.89	https://www.genecards.org/cgi-bin/ca
Protein Coding	43	GC12M123620	17.88	https://www.genecards.org/cgi-bin/ca
Protein Coding	47	GC14M095086	17.87	https://www.genecards.org/cgi-bin/ca
Protein Coding	48	GC17P034255	17.86	https://www.genecards.org/cgi-bin/ca
Biological Regior	6	GC0XP147914	17.86	https://www.genecards.org/cgi-bin/ca
Protein Coding	41	GC01M205111	17.69	https://www.genecards.org/cgi-bin/ca
Protein Coding	53	GC03P053156	17.68	https://www.genecards.org/cgi-bin/ca
Protein Coding	49	GC01M058780	17.68	https://www.genecards.org/cgi-bin/ca
Protein Coding	50	GC17M050183	17.61	https://www.genecards.org/cgi-bin/ca
Protein Coding	36	GC0XP086148	17.57	https://www.genecards.org/cgi-bin/ca
Protein Coding	45	GC02M020956	17.55	https://www.genecards.org/cgi-bin/ca
Protein Coding	51	GC10P073909	17.51	https://www.genecards.org/cgi-bin/ca
Protein Coding	43	GC06P007726	17.51	https://www.genecards.org/cgi-bin/ca
Protein Coding	47	GC13P042562	17.45	https://www.genecards.org/cgi-bin/ca
Protein Coding	39	GC13M049531	17.45	https://www.genecards.org/cgi-bin/ca
Protein Coding	48	GC13M108207	17.43	https://www.genecards.org/cgi-bin/ca
Protein Coding	47	GC01M011785	17.41	https://www.genecards.org/cgi-bin/ca
Protein Coding	41	GC07P076398	17.4	https://www.genecards.org/cgi-bin/ca
Protein Coding	25	GC0XP035919	17.4	https://www.genecards.org/cgi-bin/ca
Protein Coding	41	GC07P100720	17.39	https://www.genecards.org/cgi-bin/ca
Protein Coding	51	GC12M047841	17.38	https://www.genecards.org/cgi-bin/ca
Protein Coding	44	GC16P072089	17.36	https://www.genecards.org/cgi-bin/ca
Protein Coding	44	GC08M006499	17.31	https://www.genecards.org/cgi-bin/ca
Protein Coding	46	GC19P038647	17.3	https://www.genecards.org/cgi-bin/ca
Protein Coding	44	GC09P100099	17.26	https://www.genecards.org/cgi-bin/ca
Protein Coding	45	GC06M031315	17.23	https://www.genecards.org/cgi-bin/ca
Protein Coding	37	GC01P075796	17.22	https://www.genecards.org/cgi-bin/ca
Protein Coding	43	GC10M077969	17.19	https://www.genecards.org/cgi-bin/ca
Protein Coding	50	GC11M065653	17.18	https://www.genecards.org/cgi-bin/ca
Protein Coding	46	GC02P232378	17.17	https://www.genecards.org/cgi-bin/ca
Protein Coding	36	GC19P013862	17.14	https://www.genecards.org/cgi-bin/ca
Protein Coding	41	GC10P071396	17.01	https://www.genecards.org/cgi-bin/ca
Protein Coding	31	GC13M036169	16.99	https://www.genecards.org/cgi-bin/ca
Protein Coding	47	GC11M027654	16.91	https://www.genecards.org/cgi-bin/ca
Protein Coding	46	GC02M214725	16.89	https://www.genecards.org/cgi-bin/ca
Protein Coding	36	GC19M008848	16.86	https://www.genecards.org/cgi-bin/ca
Protein Coding	49	GC0XM154348	16.84	https://www.genecards.org/cgi-bin/ca
Protein Coding	45	GC06P159969	16.83	https://www.genecards.org/cgi-bin/ca
Protein Coding	50	GC03P155024	16.81	https://www.genecards.org/cgi-bin/ca

Protein Coding	41	GC02P238848	16.81	https://www.genecards.org/cgi-bin/ca
Protein Coding	43	GC14P094612	16.81	https://www.genecards.org/cgi-bin/ca
Protein Coding	39	GC03M016586	16.79	https://www.genecards.org/cgi-bin/ca
Protein Coding	40	GC01P156242	16.75	https://www.genecards.org/cgi-bin/ca
Protein Coding	47	GC08M142910	16.74	https://www.genecards.org/cgi-bin/ca
Protein Coding	45	GC0XM053374	16.7	https://www.genecards.org/cgi-bin/ca
Protein Coding	46	GC0XP044873	16.69	https://www.genecards.org/cgi-bin/ca
Protein Coding	43	GC02P216632	16.67	https://www.genecards.org/cgi-bin/ca
Protein Coding	45	GC03P186842	16.65	https://www.genecards.org/cgi-bin/ca
Protein Coding	49	GC05P056815	16.65	https://www.genecards.org/cgi-bin/ca
Protein Coding	47	GC07P094394	16.64	https://www.genecards.org/cgi-bin/ca
Protein Coding	46	GC04M121667	16.61	https://www.genecards.org/cgi-bin/ca
Protein Coding	46	GC0XP137566	16.61	https://www.genecards.org/cgi-bin/ca
RNA Gene	21	GC05P149430	16.58	https://www.genecards.org/cgi-bin/ca
Protein Coding	37	GC01P075128	16.57	https://www.genecards.org/cgi-bin/ca
Protein Coding	38	GC02P238320	16.55	https://www.genecards.org/cgi-bin/ca
Protein Coding	50	GC14M029576	16.53	https://www.genecards.org/cgi-bin/ca
Protein Coding	48	GC11P046720	16.48	https://www.genecards.org/cgi-bin/ca
Protein Coding	48	GC15P050424	16.47	https://www.genecards.org/cgi-bin/ca
Protein Coding	45	GC10P094501	16.46	https://www.genecards.org/cgi-bin/ca
Protein Coding	48	GC01M201359	16.45	https://www.genecards.org/cgi-bin/ca
Protein Coding	50	GC10P017227	16.4	https://www.genecards.org/cgi-bin/ca
Protein Coding	49	GC12M006328	16.38	https://www.genecards.org/cgi-bin/ca
Protein Coding	36	GC05P129460	16.35	https://www.genecards.org/cgi-bin/ca
Protein Coding	48	GC05M142555	16.3	https://www.genecards.org/cgi-bin/ca
Protein Coding	48	GC17M075751	16.29	https://www.genecards.org/cgi-bin/ca
Protein Coding	54	GC12P056094	16.28	https://www.genecards.org/cgi-bin/ca
Protein Coding	47	GC01M008861	16.28	https://www.genecards.org/cgi-bin/ca
Protein Coding	47	GC16P078099	16.26	https://www.genecards.org/cgi-bin/ca
Protein Coding	43	GC10P102503	16.25	https://www.genecards.org/cgi-bin/ca
Protein Coding	44	GC13P021671	16.25	https://www.genecards.org/cgi-bin/ca
Protein Coding	48	GC20P041028	16.25	https://www.genecards.org/cgi-bin/ca
Protein Coding	45	GC0XM077504	16.18	https://www.genecards.org/cgi-bin/ca
Protein Coding	37	GC20P057329	16.17	https://www.genecards.org/cgi-bin/ca
Protein Coding	50	GC20M010637	16.17	https://www.genecards.org/cgi-bin/ca
Protein Coding	45	GC07M087401	16.17	https://www.genecards.org/cgi-bin/ca
Protein Coding	50	GC09P027109	16.17	https://www.genecards.org/cgi-bin/ca
Protein Coding	36	GC01M219969	16.16	https://www.genecards.org/cgi-bin/ca
Protein Coding	48	GC12P008161	16.13	https://www.genecards.org/cgi-bin/ca
Protein Coding	52	GC07M081699	16.12	https://www.genecards.org/cgi-bin/ca
Protein Coding	38	GC01P027393	16.12	https://www.genecards.org/cgi-bin/ca
Protein Coding	39	GC11M044238	16.09	https://www.genecards.org/cgi-bin/ca
Protein Coding	47	GC0XP068828	16.09	https://www.genecards.org/cgi-bin/ca
Protein Coding	37	GC19P056000	16.07	https://www.genecards.org/cgi-bin/ca
Protein Coding	49	GC16P029083	16.05	https://www.genecards.org/cgi-bin/ca
Protein Coding	48	GC16M089748	16.05	https://www.genecards.org/cgi-bin/ca
Protein Coding	47	GC11P035139	15.98	https://www.genecards.org/cgi-bin/ca
Protein Coding	45	GC22M046233	15.98	https://www.genecards.org/cgi-bin/ca
Protein Coding	40	GC05P055738	15.96	https://www.genecards.org/cgi-bin/ca
Protein Coding	48	GC07M025118	15.95	https://www.genecards.org/cgi-bin/ca
Protein Coding	41	GC10P086666	15.95	https://www.genecards.org/cgi-bin/ca
Protein Coding	40	GC01M229441	15.94	https://www.genecards.org/cgi-bin/ca
Protein Coding	37	GC22M031436	15.92	https://www.genecards.org/cgi-bin/ca
Protein Coding	37	GC08P093754	15.89	https://www.genecards.org/cgi-bin/ca

Protein Coding	31	GCMTP014749	15.85	https://www.genecards.org/cgi-bin/ca
Protein Coding	47	GC0XP136649	15.84	https://www.genecards.org/cgi-bin/ca
Protein Coding	40	GC09P000831	15.79	https://www.genecards.org/cgi-bin/ca
Protein Coding	36	GC11P061391	15.77	https://www.genecards.org/cgi-bin/ca
Protein Coding	41	GC03M121769	15.72	https://www.genecards.org/cgi-bin/ca
Protein Coding	52	GC10P102394	15.7	https://www.genecards.org/cgi-bin/ca
Protein Coding	41	GC15P049423	15.63	https://www.genecards.org/cgi-bin/ca
Protein Coding	51	GC06P047451	15.63	https://www.genecards.org/cgi-bin/ca
Protein Coding	35	GC01P047416	15.62	https://www.genecards.org/cgi-bin/ca
Protein Coding	42	GC15M034788	15.59	https://www.genecards.org/cgi-bin/ca
Protein Coding	37	GC16M021198	15.58	https://www.genecards.org/cgi-bin/ca
Protein Coding	50	GC11P067583	15.55	https://www.genecards.org/cgi-bin/ca
Protein Coding	44	GC0XP129738	15.53	https://www.genecards.org/cgi-bin/ca
Protein Coding	39	GC15M089608	15.53	https://www.genecards.org/cgi-bin/ca
Protein Coding	51	GC17P068414	15.52	https://www.genecards.org/cgi-bin/ca
Protein Coding	47	GC15P056918	15.48	https://www.genecards.org/cgi-bin/ca
Protein Coding	47	GC20M031664	15.42	https://www.genecards.org/cgi-bin/ca
Protein Coding	48	GC11M102425	15.4	https://www.genecards.org/cgi-bin/ca
Protein Coding	46	GC08M027596	15.4	https://www.genecards.org/cgi-bin/ca
Protein Coding	40	GC11P117314	15.38	https://www.genecards.org/cgi-bin/ca
Protein Coding	49	GC07P074082	15.37	https://www.genecards.org/cgi-bin/ca
Protein Coding	41	GC12M021468	15.36	https://www.genecards.org/cgi-bin/ca
Protein Coding	47	GC0XP041085	15.36	https://www.genecards.org/cgi-bin/ca
Protein Coding	41	GC01P243255	15.32	https://www.genecards.org/cgi-bin/ca
Protein Coding	50	GC11P119206	15.31	https://www.genecards.org/cgi-bin/ca
Protein Coding	37	GC22M041525	15.31	https://www.genecards.org/cgi-bin/ca
Protein Coding	39	GC02M027448	15.29	https://www.genecards.org/cgi-bin/ca
Protein Coding	45	GC08M107246	15.27	https://www.genecards.org/cgi-bin/ca
Protein Coding	50	GC10P048306	15.26	https://www.genecards.org/cgi-bin/ca
Protein Coding	43	GC17M035871	15.22	https://www.genecards.org/cgi-bin/ca
Protein Coding	47	GC04M176683	15.2	https://www.genecards.org/cgi-bin/ca
Protein Coding	49	GC16M030117	15.2	https://www.genecards.org/cgi-bin/ca
RNA Gene	21	GC19P051720	15.2	https://www.genecards.org/cgi-bin/ca
Protein Coding	47	GC02P188974	15.19	https://www.genecards.org/cgi-bin/ca
Protein Coding	40	GC06M161348	15.18	https://www.genecards.org/cgi-bin/ca
Protein Coding	37	GC22M045344	15.16	https://www.genecards.org/cgi-bin/ca
Protein Coding	45	GC14P020455	15.13	https://www.genecards.org/cgi-bin/ca
Protein Coding	45	GC10M044294	15.1	https://www.genecards.org/cgi-bin/ca
Protein Coding	40	GC15M082543	15.03	https://www.genecards.org/cgi-bin/ca
Protein Coding	39	GC14P063761	15.02	https://www.genecards.org/cgi-bin/ca
Protein Coding	41	GC12M101728	14.98	https://www.genecards.org/cgi-bin/ca
Protein Coding	44	GC15P039581	14.97	https://www.genecards.org/cgi-bin/ca
Protein Coding	41	GC0XP028605	14.96	https://www.genecards.org/cgi-bin/ca
Protein Coding	43	GC06M057187	14.95	https://www.genecards.org/cgi-bin/ca
Protein Coding	48	GC02P201182	14.95	https://www.genecards.org/cgi-bin/ca
Protein Coding	35	GC11M119097	14.92	https://www.genecards.org/cgi-bin/ca
Protein Coding	48	GC0XP134460	14.91	https://www.genecards.org/cgi-bin/ca
Protein Coding	46	GC03M172505	14.88	https://www.genecards.org/cgi-bin/ca
Protein Coding	48	GC01P154405	14.87	https://www.genecards.org/cgi-bin/ca
Protein Coding	43	GC09P128149	14.87	https://www.genecards.org/cgi-bin/ca
Protein Coding	32	GC08P047260	14.87	https://www.genecards.org/cgi-bin/ca
Protein Coding	47	GC03P010026	14.87	https://www.genecards.org/cgi-bin/ca
Protein Coding	47	GC11M102617	14.87	https://www.genecards.org/cgi-bin/ca
Protein Coding	43	GC01P109911	14.86	https://www.genecards.org/cgi-bin/ca

Protein Coding	39	GC09P021494	14.86	https://www.genecards.org/cgi-bin/ca
RNA Gene	21	GC01P001463	14.84	https://www.genecards.org/cgi-bin/ca
Protein Coding	29	GC20P001226	14.84	https://www.genecards.org/cgi-bin/ca
Protein Coding	44	GC05P132060	14.84	https://www.genecards.org/cgi-bin/ca
Protein Coding	39	GC21M044222	14.83	https://www.genecards.org/cgi-bin/ca
Protein Coding	39	GC16M053597	14.83	https://www.genecards.org/cgi-bin/ca
Protein Coding	50	GC19M010133	14.83	https://www.genecards.org/cgi-bin/ca
Protein Coding	49	GC19M015159	14.82	https://www.genecards.org/cgi-bin/ca
Protein Coding	43	GC05M039371	14.81	https://www.genecards.org/cgi-bin/ca
Protein Coding	49	GC17P059893	14.81	https://www.genecards.org/cgi-bin/ca
Protein Coding	45	GC19M035825	14.79	https://www.genecards.org/cgi-bin/ca
Protein Coding	45	GC09P134641	14.74	https://www.genecards.org/cgi-bin/ca
Protein Coding	41	GC19M042443	14.74	https://www.genecards.org/cgi-bin/ca
Protein Coding	46	GC19P000854	14.73	https://www.genecards.org/cgi-bin/ca
Protein Coding	47	GC12M091140	14.72	https://www.genecards.org/cgi-bin/ca
Protein Coding	41	GC01P228175	14.7	https://www.genecards.org/cgi-bin/ca
Protein Coding	46	GC0XM049250	14.7	https://www.genecards.org/cgi-bin/ca
Protein Coding	40	GC17M064319	14.69	https://www.genecards.org/cgi-bin/ca
Protein Coding	44	GC08M066176	14.68	https://www.genecards.org/cgi-bin/ca
Protein Coding	35	GC07P135231	14.65	https://www.genecards.org/cgi-bin/ca
Protein Coding	47	GC15M074719	14.65	https://www.genecards.org/cgi-bin/ca
Protein Coding	44	GC04M076021	14.65	https://www.genecards.org/cgi-bin/ca
Protein Coding	39	GC16M023307	14.64	https://www.genecards.org/cgi-bin/ca
Protein Coding	51	GC02M025228	14.62	https://www.genecards.org/cgi-bin/ca
Protein Coding	47	GC17P078214	14.6	https://www.genecards.org/cgi-bin/ca
Protein Coding	46	GC22M050525	14.58	https://www.genecards.org/cgi-bin/ca
Protein Coding	36	GC01P114854	14.53	https://www.genecards.org/cgi-bin/ca
Protein Coding	45	GC03P058008	14.52	https://www.genecards.org/cgi-bin/ca
Protein Coding	44	GC19M043646	14.51	https://www.genecards.org/cgi-bin/ca
Protein Coding	41	GC05P133051	14.51	https://www.genecards.org/cgi-bin/ca
Protein Coding	29	GC0XP134796	14.5	https://www.genecards.org/cgi-bin/ca
Protein Coding	43	GC11M065867	14.5	https://www.genecards.org/cgi-bin/ca
Protein Coding	32	GCMTP005906	14.48	https://www.genecards.org/cgi-bin/ca
Protein Coding	48	GC07M099759	14.47	https://www.genecards.org/cgi-bin/ca
Protein Coding	47	GC13M051930	14.47	https://www.genecards.org/cgi-bin/ca
Protein Coding	52	GC02M136114	14.44	https://www.genecards.org/cgi-bin/ca
Protein Coding	45	GC09M097635	14.44	https://www.genecards.org/cgi-bin/ca
Protein Coding	44	GC20M023026	14.43	https://www.genecards.org/cgi-bin/ca
Protein Coding	45	GC15P088813	14.4	https://www.genecards.org/cgi-bin/ca
Protein Coding	37	GC03M160256	14.39	https://www.genecards.org/cgi-bin/ca
Protein Coding	45	GC12M114353	14.37	https://www.genecards.org/cgi-bin/ca
Protein Coding	48	GC19M045769	14.36	https://www.genecards.org/cgi-bin/ca
Protein Coding	41	GC18P059220	14.35	https://www.genecards.org/cgi-bin/ca
Protein Coding	39	GC20M045252	14.35	https://www.genecards.org/cgi-bin/ca
Protein Coding	50	GC20P032762	14.35	https://www.genecards.org/cgi-bin/ca
Protein Coding	54	GC07M148807	14.35	https://www.genecards.org/cgi-bin/ca
Protein Coding	43	GC02M085657	14.34	https://www.genecards.org/cgi-bin/ca
Protein Coding	49	GC0XP123859	14.32	https://www.genecards.org/cgi-bin/ca
Protein Coding	46	GC09P122370	14.29	https://www.genecards.org/cgi-bin/ca
Protein Coding	47	GC02M049918	14.28	https://www.genecards.org/cgi-bin/ca
Protein Coding	46	GC07P018086	14.27	https://www.genecards.org/cgi-bin/ca
Protein Coding	52	GC04M101024	14.23	https://www.genecards.org/cgi-bin/ca
Protein Coding	47	GC02M127257	14.22	https://www.genecards.org/cgi-bin/ca
Protein Coding	45	GC05M096391	14.22	https://www.genecards.org/cgi-bin/ca

Protein Coding	46	GC15M038488	14.16	https://www.genecards.org/cgi-bin/ca
Protein Coding	47	GC09M125234	14.16	https://www.genecards.org/cgi-bin/ca
Protein Coding	47	GC02M197486	14.11	https://www.genecards.org/cgi-bin/ca
Protein Coding	45	GC02M026190	14.08	https://www.genecards.org/cgi-bin/ca
Protein Coding	49	GC16M069706	14.07	https://www.genecards.org/cgi-bin/ca
Protein Coding	50	GC20P044355	14.07	https://www.genecards.org/cgi-bin/ca
Protein Coding	40	GC14P104788	14.07	https://www.genecards.org/cgi-bin/ca
Protein Coding	45	GC10P060772	14.05	https://www.genecards.org/cgi-bin/ca
Protein Coding	39	GC11P001151	14.04	https://www.genecards.org/cgi-bin/ca
RNA Gene	20	GC07M129770	14.04	https://www.genecards.org/cgi-bin/ca
Protein Coding	44	GC11P067468	14.04	https://www.genecards.org/cgi-bin/ca
Protein Coding	55	GC02M211375	14.03	https://www.genecards.org/cgi-bin/ca
Protein Coding	51	GC10P086756	14.02	https://www.genecards.org/cgi-bin/ca
RNA Gene	22	GC05P149410	14	https://www.genecards.org/cgi-bin/ca
RNA Gene	20	GC01M156420	14	https://www.genecards.org/cgi-bin/ca
Protein Coding	44	GC12P053380	14	https://www.genecards.org/cgi-bin/ca
Protein Coding	49	GC05M080626	13.98	https://www.genecards.org/cgi-bin/ca
Protein Coding	43	GC01M207880	13.97	https://www.genecards.org/cgi-bin/ca
Protein Coding	44	GC01P026693	13.94	https://www.genecards.org/cgi-bin/ca
RNA Gene	19	GC17M007018	13.94	https://www.genecards.org/cgi-bin/ca
Protein Coding	42	GC07M152644	13.91	https://www.genecards.org/cgi-bin/ca
Protein Coding	45	GC0XM133535	13.89	https://www.genecards.org/cgi-bin/ca
Protein Coding	45	GC03P147393	13.89	https://www.genecards.org/cgi-bin/ca
Protein Coding	45	GC14M024630	13.87	https://www.genecards.org/cgi-bin/ca
RNA Gene	21	GC12P008229	13.87	https://www.genecards.org/cgi-bin/ca
Protein Coding	44	GC12M057623	13.83	https://www.genecards.org/cgi-bin/ca
Protein Coding	47	GC03M048173	13.83	https://www.genecards.org/cgi-bin/ca
Protein Coding	46	GC02P201117	13.8	https://www.genecards.org/cgi-bin/ca
Protein Coding	41	GC09M014730	13.79	https://www.genecards.org/cgi-bin/ca
Protein Coding	49	GC19P011061	13.78	https://www.genecards.org/cgi-bin/ca
Protein Coding	43	GC06P026087	13.78	https://www.genecards.org/cgi-bin/ca
Protein Coding	44	GC19P000571	13.75	https://www.genecards.org/cgi-bin/ca
Protein Coding	47	GC16P066366	13.75	https://www.genecards.org/cgi-bin/ca
Protein Coding	45	GC12P130871	13.74	https://www.genecards.org/cgi-bin/ca
Protein Coding	47	GC18M047809	13.74	https://www.genecards.org/cgi-bin/ca
Protein Coding	45	GC09P128340	13.74	https://www.genecards.org/cgi-bin/ca
RNA Gene	21	GC12P008231	13.72	https://www.genecards.org/cgi-bin/ca
Protein Coding	45	GC03M052452	13.65	https://www.genecards.org/cgi-bin/ca
Protein Coding	45	GC22P023786	13.61	https://www.genecards.org/cgi-bin/ca
Protein Coding	37	GC16M075536	13.61	https://www.genecards.org/cgi-bin/ca
Protein Coding	51	GC18M063123	13.59	https://www.genecards.org/cgi-bin/ca
Protein Coding	49	GC04P147480	13.59	https://www.genecards.org/cgi-bin/ca
Protein Coding	44	GC05P177380	13.58	https://www.genecards.org/cgi-bin/ca
Protein Coding	47	GC10P099782	13.57	https://www.genecards.org/cgi-bin/ca
Protein Coding	40	GC20P003068	13.56	https://www.genecards.org/cgi-bin/ca
Protein Coding	48	GC20P046118	13.55	https://www.genecards.org/cgi-bin/ca
Protein Coding	47	GC19M006677	13.55	https://www.genecards.org/cgi-bin/ca
Protein Coding	50	GC01M114704	13.53	https://www.genecards.org/cgi-bin/ca
Protein Coding	47	GC03P181711	13.53	https://www.genecards.org/cgi-bin/ca
Protein Coding	45	GC08P060678	13.51	https://www.genecards.org/cgi-bin/ca
Protein Coding	43	GC0XP103773	13.51	https://www.genecards.org/cgi-bin/ca
Protein Coding	50	GC18M027950	13.48	https://www.genecards.org/cgi-bin/ca
Protein Coding	50	GC14M035401	13.45	https://www.genecards.org/cgi-bin/ca
Protein Coding	36	GC02P171434	13.42	https://www.genecards.org/cgi-bin/ca

RNA Gene	25	GC11M002661	13.41	https://www.genecards.org/cgi-bin/ca
Protein Coding	51	GC20M005114	13.4	https://www.genecards.org/cgi-bin/ca
Protein Coding	38	GC16P003024	13.4	https://www.genecards.org/cgi-bin/ca
Protein Coding	44	GC15M043403	13.39	https://www.genecards.org/cgi-bin/ca
Protein Coding	49	GC07P006380	13.38	https://www.genecards.org/cgi-bin/ca
Protein Coding	48	GC01P063593	13.37	https://www.genecards.org/cgi-bin/ca
Protein Coding	40	GC16M070482	13.35	https://www.genecards.org/cgi-bin/ca
Protein Coding	45	GC09M110243	13.33	https://www.genecards.org/cgi-bin/ca
Protein Coding	45	GC09M136673	13.32	https://www.genecards.org/cgi-bin/ca
Protein Coding	48	GC12P052948	13.31	https://www.genecards.org/cgi-bin/ca
Protein Coding	45	GC01M169511	13.28	https://www.genecards.org/cgi-bin/ca
Protein Coding	50	GC01M064833	13.26	https://www.genecards.org/cgi-bin/ca
Protein Coding	35	GC09P129111	13.25	https://www.genecards.org/cgi-bin/ca
RNA Gene	18	GC01M198860	13.24	https://www.genecards.org/cgi-bin/ca
Protein Coding	52	GC01M243488	13.24	https://www.genecards.org/cgi-bin/ca
Protein Coding	48	GC14M102080	13.23	https://www.genecards.org/cgi-bin/ca
Protein Coding	47	GC10M033177	13.23	https://www.genecards.org/cgi-bin/ca
RNA Gene	21	GC02P176150	13.22	https://www.genecards.org/cgi-bin/ca
Protein Coding	50	GC03P024830	13.22	https://www.genecards.org/cgi-bin/ca
Protein Coding	54	GC07M092604	13.19	https://www.genecards.org/cgi-bin/ca
Protein Coding	52	GC09P130713	13.19	https://www.genecards.org/cgi-bin/ca
Protein Coding	46	GC17M042810	13.18	https://www.genecards.org/cgi-bin/ca
Protein Coding	48	GC07P106865	13.16	https://www.genecards.org/cgi-bin/ca
Protein Coding	40	GC07M073768	13.16	https://www.genecards.org/cgi-bin/ca
Protein Coding	48	GC03M196027	13.14	https://www.genecards.org/cgi-bin/ca
Protein Coding	48	GC09P121201	13.14	https://www.genecards.org/cgi-bin/ca
Protein Coding	53	GC19M004090	13.14	https://www.genecards.org/cgi-bin/ca
RNA Gene	20	GC01P209432	13.14	https://www.genecards.org/cgi-bin/ca
Protein Coding	52	GC03M123610	13.13	https://www.genecards.org/cgi-bin/ca
Protein Coding	47	GC20P041136	13.12	https://www.genecards.org/cgi-bin/ca
Protein Coding	50	GC08M023020	13.08	https://www.genecards.org/cgi-bin/ca
Protein Coding	44	GC12M002857	13.08	https://www.genecards.org/cgi-bin/ca
Protein Coding	48	GC13M040555	13.08	https://www.genecards.org/cgi-bin/ca
Protein Coding	50	GC03M038549	13.08	https://www.genecards.org/cgi-bin/ca
Protein Coding	41	GC12M000912	13.07	https://www.genecards.org/cgi-bin/ca
RNA Gene	20	GC06M071403	13.06	https://www.genecards.org/cgi-bin/ca
RNA Gene	24	GC11P065806	13.06	https://www.genecards.org/cgi-bin/ca
Protein Coding	39	GC04P015471	13.05	https://www.genecards.org/cgi-bin/ca
Protein Coding	41	GC19P023329	13.02	https://www.genecards.org/cgi-bin/ca
Protein Coding	45	GC02P111119	13.01	https://www.genecards.org/cgi-bin/ca
Protein Coding	48	GC12P012716	13.01	https://www.genecards.org/cgi-bin/ca
Protein Coding	48	GC06P160702	12.99	https://www.genecards.org/cgi-bin/ca
Protein Coding	45	GC09P137618	12.98	https://www.genecards.org/cgi-bin/ca
Protein Coding	45	GC01M094530	12.96	https://www.genecards.org/cgi-bin/ca
Protein Coding	45	GC0XM154021	12.96	https://www.genecards.org/cgi-bin/ca
Protein Coding	47	GC01M154127	12.94	https://www.genecards.org/cgi-bin/ca
Protein Coding	48	GC01M174153	12.94	https://www.genecards.org/cgi-bin/ca
Protein Coding	47	GC03M169083	12.89	https://www.genecards.org/cgi-bin/ca
Protein Coding	40	GC21M046235	12.89	https://www.genecards.org/cgi-bin/ca
Protein Coding	43	GC16P089492	12.88	https://www.genecards.org/cgi-bin/ca
Protein Coding	39	GC17P043170	12.87	https://www.genecards.org/cgi-bin/ca
Protein Coding	44	GC01P018631	12.87	https://www.genecards.org/cgi-bin/ca
Protein Coding	44	GC04M015965	12.87	https://www.genecards.org/cgi-bin/ca
RNA Gene	20	GC17M058331	12.82	https://www.genecards.org/cgi-bin/ca

Protein Coding	41	GC09P116179	12.81	https://www.genecards.org/cgi-bin/ca
Protein Coding	45	GC01P046258	12.81	https://www.genecards.org/cgi-bin/ca
Protein Coding	45	GC15P040929	12.79	https://www.genecards.org/cgi-bin/ca
Protein Coding	43	GC05M140332	12.77	https://www.genecards.org/cgi-bin/ca
Protein Coding	47	GC06P131808	12.77	https://www.genecards.org/cgi-bin/ca
Protein Coding	52	GC05M150053	12.76	https://www.genecards.org/cgi-bin/ca
Protein Coding	41	GC07P073799	12.74	https://www.genecards.org/cgi-bin/ca
Protein Coding	48	GC15P073930	12.74	https://www.genecards.org/cgi-bin/ca
Protein Coding	47	GC17M041582	12.71	https://www.genecards.org/cgi-bin/ca
Protein Coding	42	GC08M078689	12.7	https://www.genecards.org/cgi-bin/ca
Protein Coding	41	GC02M189031	12.7	https://www.genecards.org/cgi-bin/ca
Protein Coding	44	GC18P063871	12.67	https://www.genecards.org/cgi-bin/ca
Protein Coding	47	GC03P130619	12.67	https://www.genecards.org/cgi-bin/ca
Protein Coding	47	GC08P038007	12.66	https://www.genecards.org/cgi-bin/ca
Protein Coding	43	GC05P160422	12.64	https://www.genecards.org/cgi-bin/ca
Protein Coding	44	GC06M073593	12.64	https://www.genecards.org/cgi-bin/ca
Protein Coding	44	GC02M055865	12.61	https://www.genecards.org/cgi-bin/ca
Protein Coding	43	GC06M053497	12.61	https://www.genecards.org/cgi-bin/ca
Protein Coding	41	GC09M099216	12.6	https://www.genecards.org/cgi-bin/ca
Protein Coding	50	GC10M032900	12.58	https://www.genecards.org/cgi-bin/ca
Protein Coding	50	GC10M006010	12.56	https://www.genecards.org/cgi-bin/ca
Protein Coding	48	GC12M132624	12.54	https://www.genecards.org/cgi-bin/ca
Protein Coding	40	GC01P168280	12.51	https://www.genecards.org/cgi-bin/ca
Protein Coding	47	GC06M010393	12.48	https://www.genecards.org/cgi-bin/ca
Protein Coding	44	GC02M043806	12.46	https://www.genecards.org/cgi-bin/ca
Protein Coding	41	GC08P015417	12.45	https://www.genecards.org/cgi-bin/ca
Protein Coding	42	GC03M187668	12.44	https://www.genecards.org/cgi-bin/ca
RNA Gene	21	GC19M049500	12.44	https://www.genecards.org/cgi-bin/ca
Protein Coding	48	GC19P048954	12.43	https://www.genecards.org/cgi-bin/ca
Protein Coding	48	GC09M104781	12.43	https://www.genecards.org/cgi-bin/ca
Protein Coding	47	GC02P120735	12.42	https://www.genecards.org/cgi-bin/ca
RNA Gene	18	GC07M025993	12.42	https://www.genecards.org/cgi-bin/ca
Protein Coding	51	GC03P148697	12.39	https://www.genecards.org/cgi-bin/ca
Protein Coding	49	GC16P023888	12.38	https://www.genecards.org/cgi-bin/ca
Protein Coding	41	GC08P104590	12.38	https://www.genecards.org/cgi-bin/ca
Protein Coding	36	GC20P043590	12.38	https://www.genecards.org/cgi-bin/ca
Protein Coding	51	GC08M011842	12.37	https://www.genecards.org/cgi-bin/ca
Protein Coding	49	GC06P007541	12.36	https://www.genecards.org/cgi-bin/ca
Protein Coding	41	GC09M086944	12.35	https://www.genecards.org/cgi-bin/ca
Protein Coding	50	GC10P113679	12.35	https://www.genecards.org/cgi-bin/ca
Protein Coding	44	GC13M030456	12.34	https://www.genecards.org/cgi-bin/ca
Protein Coding	48	GC17P070168	12.33	https://www.genecards.org/cgi-bin/ca
Protein Coding	32	GC19P049043	12.29	https://www.genecards.org/cgi-bin/ca
Protein Coding	41	GC10M087751	12.29	https://www.genecards.org/cgi-bin/ca
Protein Coding	48	GC04P185143	12.29	https://www.genecards.org/cgi-bin/ca
Protein Coding	44	GC01P067685	12.27	https://www.genecards.org/cgi-bin/ca
Protein Coding	41	GC17P008049	12.27	https://www.genecards.org/cgi-bin/ca
Protein Coding	50	GC03P046877	12.26	https://www.genecards.org/cgi-bin/ca
Protein Coding	49	GC08M102204	12.24	https://www.genecards.org/cgi-bin/ca
Protein Coding	45	GC08M023190	12.24	https://www.genecards.org/cgi-bin/ca
Protein Coding	49	GC08P019901	12.23	https://www.genecards.org/cgi-bin/ca
Protein Coding	48	GC19M055151	12.22	https://www.genecards.org/cgi-bin/ca
Protein Coding	39	GC0XM047142	12.2	https://www.genecards.org/cgi-bin/ca
Protein Coding	47	GC03M093873	12.18	https://www.genecards.org/cgi-bin/ca

Protein Coding	48	GC15M089784	12.16	https://www.genecards.org/cgi-bin/ca
Protein Coding	51	GC02P170813	12.15	https://www.genecards.org/cgi-bin/ca
Protein Coding	49	GC17M082078	12.14	https://www.genecards.org/cgi-bin/ca
Protein Coding	40	GC10M095663	12.11	https://www.genecards.org/cgi-bin/ca
Protein Coding	49	GC12P006786	12.11	https://www.genecards.org/cgi-bin/ca
Protein Coding	44	GC04M152321	12.1	https://www.genecards.org/cgi-bin/ca
Protein Coding	50	GC12M013437	12.01	https://www.genecards.org/cgi-bin/ca
Protein Coding	41	GC08P017576	12.01	https://www.genecards.org/cgi-bin/ca
Protein Coding	44	GC06M033162	12	https://www.genecards.org/cgi-bin/ca
Protein Coding	46	GC08P031639	11.99	https://www.genecards.org/cgi-bin/ca
Protein Coding	47	GC01M229431	11.98	https://www.genecards.org/cgi-bin/ca
Protein Coding	47	GC07M065960	11.98	https://www.genecards.org/cgi-bin/ca
RNA Gene	17	GC14P104793	11.96	https://www.genecards.org/cgi-bin/ca
Protein Coding	50	GC04M098871	11.95	https://www.genecards.org/cgi-bin/ca
Protein Coding	44	GC20M023608	11.94	https://www.genecards.org/cgi-bin/ca
Protein Coding	47	GC09M022002	11.91	https://www.genecards.org/cgi-bin/ca
RNA Gene	20	GC12P062606	11.9	https://www.genecards.org/cgi-bin/ca
RNA Gene	25	GC12M053962	11.87	https://www.genecards.org/cgi-bin/ca
Protein Coding	47	GC17P044345	11.86	https://www.genecards.org/cgi-bin/ca
Protein Coding	45	GC06M158765	11.85	https://www.genecards.org/cgi-bin/ca
Protein Coding	45	GC17M064039	11.85	https://www.genecards.org/cgi-bin/ca
Protein Coding	42	GC04P168497	11.84	https://www.genecards.org/cgi-bin/ca
Protein Coding	40	GC0XM014690	11.77	https://www.genecards.org/cgi-bin/ca
Protein Coding	46	GC14M036516	11.76	https://www.genecards.org/cgi-bin/ca
Protein Coding	49	GC01M167399	11.76	https://www.genecards.org/cgi-bin/ca
Protein Coding	44	GC0XP071118	11.74	https://www.genecards.org/cgi-bin/ca
Protein Coding	44	GC13M109752	11.67	https://www.genecards.org/cgi-bin/ca
Protein Coding	49	GC03M055465	11.66	https://www.genecards.org/cgi-bin/ca
Protein Coding	36	GC08M033473	11.63	https://www.genecards.org/cgi-bin/ca
Protein Coding	43	GC11M014945	11.63	https://www.genecards.org/cgi-bin/ca
Protein Coding	47	GC08M118923	11.6	https://www.genecards.org/cgi-bin/ca
Protein Coding	46	GC22P024927	11.59	https://www.genecards.org/cgi-bin/ca
Protein Coding	47	GC10M052760	11.58	https://www.genecards.org/cgi-bin/ca
Protein Coding	39	GC12P007787	11.55	https://www.genecards.org/cgi-bin/ca
Protein Coding	45	GC16P013920	11.52	https://www.genecards.org/cgi-bin/ca
Protein Coding	36	GC07M093099	11.51	https://www.genecards.org/cgi-bin/ca
Protein Coding	43	GC06M143940	11.5	https://www.genecards.org/cgi-bin/ca
Protein Coding	50	GC11M113409	11.49	https://www.genecards.org/cgi-bin/ca
Protein Coding	44	GC17M029955	11.49	https://www.genecards.org/cgi-bin/ca
Protein Coding	45	GC01M241499	11.48	https://www.genecards.org/cgi-bin/ca
Protein Coding	47	GC19M011574	11.46	https://www.genecards.org/cgi-bin/ca
Protein Coding	43	GC22P045502	11.42	https://www.genecards.org/cgi-bin/ca
Protein Coding	47	GC16M067939	11.42	https://www.genecards.org/cgi-bin/ca
RNA Gene	16	GC14P104815	11.41	https://www.genecards.org/cgi-bin/ca
Protein Coding	41	GC17P028725	11.41	https://www.genecards.org/cgi-bin/ca
Protein Coding	44	GC12P123712	11.39	https://www.genecards.org/cgi-bin/ca
Protein Coding	51	GC22P019941	11.37	https://www.genecards.org/cgi-bin/ca
Protein Coding	40	GC12M076344	11.36	https://www.genecards.org/cgi-bin/ca
Protein Coding	43	GC01M102876	11.35	https://www.genecards.org/cgi-bin/ca
Protein Coding	46	GC03M053224	11.34	https://www.genecards.org/cgi-bin/ca
Protein Coding	49	GC01M230702	11.34	https://www.genecards.org/cgi-bin/ca
Protein Coding	45	GC04P004861	11.31	https://www.genecards.org/cgi-bin/ca
Protein Coding	47	GC05M037812	11.28	https://www.genecards.org/cgi-bin/ca
Protein Coding	43	GC14M074941	11.25	https://www.genecards.org/cgi-bin/ca

RNA Gene	23	GC11P065794	11.25	https://www.genecards.org/cgi-bin/ca
Protein Coding	47	GC14P080954	11.25	https://www.genecards.org/cgi-bin/ca
Protein Coding	43	GC21P017512	11.25	https://www.genecards.org/cgi-bin/ca
Protein Coding	49	GC11P002444	11.24	https://www.genecards.org/cgi-bin/ca
Protein Coding	45	GC08M116846	11.24	https://www.genecards.org/cgi-bin/ca
Protein Coding	41	GC11M010653	11.2	https://www.genecards.org/cgi-bin/ca
RNA Gene	22	GC22P030969	11.18	https://www.genecards.org/cgi-bin/ca
Protein Coding	43	GC03M010285	11.16	https://www.genecards.org/cgi-bin/ca
Protein Coding	50	GC05M143277	11.16	https://www.genecards.org/cgi-bin/ca
Protein Coding	48	GC01P053196	11.16	https://www.genecards.org/cgi-bin/ca
Protein Coding	41	GC02P073385	11.15	https://www.genecards.org/cgi-bin/ca
Protein Coding	51	GC04P153684	11.13	https://www.genecards.org/cgi-bin/ca
Protein Coding	40	GC09P006720	11.1	https://www.genecards.org/cgi-bin/ca
Protein Coding	43	GC17P042567	11.09	https://www.genecards.org/cgi-bin/ca
Protein Coding	40	GC19P013117	11.06	https://www.genecards.org/cgi-bin/ca
Protein Coding	47	GC0XM072329	11.05	https://www.genecards.org/cgi-bin/ca
Protein Coding	42	GC05P163438	11.05	https://www.genecards.org/cgi-bin/ca
RNA Gene	21	GC09P021994	11.05	https://www.genecards.org/cgi-bin/ca
Protein Coding	46	GC09P087725	11.01	https://www.genecards.org/cgi-bin/ca
Protein Coding	51	GC01P022057	10.93	https://www.genecards.org/cgi-bin/ca
Protein Coding	47	GC12M057757	10.91	https://www.genecards.org/cgi-bin/ca
Protein Coding	33	GC06M106969	10.89	https://www.genecards.org/cgi-bin/ca
Protein Coding	43	GC07P005592	10.85	https://www.genecards.org/cgi-bin/ca
RNA Gene	24	GC0XM073820	10.84	https://www.genecards.org/cgi-bin/ca
Protein Coding	51	GC01P055039	10.82	https://www.genecards.org/cgi-bin/ca
Protein Coding	42	GC11P002899	10.81	https://www.genecards.org/cgi-bin/ca
Protein Coding	27	GC10M049517	10.79	https://www.genecards.org/cgi-bin/ca
Protein Coding	39	GC16M003581	10.77	https://www.genecards.org/cgi-bin/ca
Protein Coding	47	GC13P113105	10.75	https://www.genecards.org/cgi-bin/ca
Protein Coding	52	GC07M100803	10.73	https://www.genecards.org/cgi-bin/ca
Protein Coding	42	GC14M075013	10.72	https://www.genecards.org/cgi-bin/ca
Protein Coding	40	GC05P060945	10.7	https://www.genecards.org/cgi-bin/ca
Protein Coding	46	GC06M166409	10.69	https://www.genecards.org/cgi-bin/ca
RNA Gene	21	GC07M130877	10.68	https://www.genecards.org/cgi-bin/ca
Protein Coding	39	GC0XP085243	10.66	https://www.genecards.org/cgi-bin/ca
Protein Coding	39	GC0XM106032	10.65	https://www.genecards.org/cgi-bin/ca
Protein Coding	46	GC02M086784	10.63	https://www.genecards.org/cgi-bin/ca
Protein Coding	44	GC01P016043	10.62	https://www.genecards.org/cgi-bin/ca
Protein Coding	44	GC01M037612	10.6	https://www.genecards.org/cgi-bin/ca
Protein Coding	49	GC11P128686	10.58	https://www.genecards.org/cgi-bin/ca
Protein Coding	47	GC11P006390	10.58	https://www.genecards.org/cgi-bin/ca
Protein Coding	48	GC19P041859	10.56	https://www.genecards.org/cgi-bin/ca
Protein Coding	47	GC12M052514	10.55	https://www.genecards.org/cgi-bin/ca
Protein Coding	42	GC07P074658	10.54	https://www.genecards.org/cgi-bin/ca
Protein Coding	49	GC07M005527	10.54	https://www.genecards.org/cgi-bin/ca
Protein Coding	46	GC02M070447	10.54	https://www.genecards.org/cgi-bin/ca
Protein Coding	41	GC03M042274	10.54	https://www.genecards.org/cgi-bin/ca
Protein Coding	44	GC15P058410	10.53	https://www.genecards.org/cgi-bin/ca
Protein Coding	37	GC0XP153179	10.51	https://www.genecards.org/cgi-bin/ca
Protein Coding	44	GC10P119651	10.5	https://www.genecards.org/cgi-bin/ca
Protein Coding	40	GC07M073440	10.5	https://www.genecards.org/cgi-bin/ca
Protein Coding	49	GC14M094376	10.48	https://www.genecards.org/cgi-bin/ca
Protein Coding	44	GC01P207454	10.46	https://www.genecards.org/cgi-bin/ca
RNA Gene	21	GC21P016539	10.46	https://www.genecards.org/cgi-bin/ca

Protein Coding	49	GC10P008045	10.44	https://www.genecards.org/cgi-bin/ca
Protein Coding	46	GC02M113215	10.41	https://www.genecards.org/cgi-bin/ca
Protein Coding	45	GC02M084423	10.39	https://www.genecards.org/cgi-bin/ca
Protein Coding	50	GC04M089724	10.37	https://www.genecards.org/cgi-bin/ca
Protein Coding	46	GC06P047417	10.37	https://www.genecards.org/cgi-bin/ca
Protein Coding	47	GC17M041754	10.37	https://www.genecards.org/cgi-bin/ca
Protein Coding	32	GC10M103878	10.36	https://www.genecards.org/cgi-bin/ca
Protein Coding	47	GC17M044905	10.34	https://www.genecards.org/cgi-bin/ca
RNA Gene	10	GC05M132631	10.33	https://www.genecards.org/cgi-bin/ca
Protein Coding	44	GC21P043169	10.32	https://www.genecards.org/cgi-bin/ca
Protein Coding	45	GC01P196621	10.32	https://www.genecards.org/cgi-bin/ca
Protein Coding	44	GC02P216107	10.3	https://www.genecards.org/cgi-bin/ca
Protein Coding	51	GC07P150990	10.29	https://www.genecards.org/cgi-bin/ca
Protein Coding	36	GC11P061362	10.27	https://www.genecards.org/cgi-bin/ca
Protein Coding	45	GC0XP154379	10.27	https://www.genecards.org/cgi-bin/ca
Protein Coding	45	GC22P041622	10.27	https://www.genecards.org/cgi-bin/ca
RNA Gene	14	GC05M142318	10.27	https://www.genecards.org/cgi-bin/ca
Protein Coding	45	GC12M027959	10.27	https://www.genecards.org/cgi-bin/ca
Protein Coding	51	GC21P031659	10.25	https://www.genecards.org/cgi-bin/ca
Protein Coding	46	GC04M109740	10.25	https://www.genecards.org/cgi-bin/ca
Protein Coding	42	GC07P074461	10.24	https://www.genecards.org/cgi-bin/ca
Protein Coding	45	GC12M004368	10.23	https://www.genecards.org/cgi-bin/ca
Protein Coding	41	GC17M040875	10.21	https://www.genecards.org/cgi-bin/ca
Protein Coding	43	GC11M117800	10.2	https://www.genecards.org/cgi-bin/ca
Protein Coding	44	GC01M169722	10.19	https://www.genecards.org/cgi-bin/ca
Protein Coding	45	GC15M028111	10.17	https://www.genecards.org/cgi-bin/ca
Protein Coding	40	GC20M037251	10.16	https://www.genecards.org/cgi-bin/ca
Protein Coding	44	GC15P024823	10.15	https://www.genecards.org/cgi-bin/ca
Protein Coding	51	GC05P177086	10.14	https://www.genecards.org/cgi-bin/ca
Protein Coding	39	GC15M044562	10.13	https://www.genecards.org/cgi-bin/ca
Protein Coding	41	GC06M152121	10.07	https://www.genecards.org/cgi-bin/ca
Protein Coding	49	GC03P133666	10.06	https://www.genecards.org/cgi-bin/ca
Protein Coding	52	GC12P055966	10.06	https://www.genecards.org/cgi-bin/ca
Protein Coding	48	GC11P003855	10.02	https://www.genecards.org/cgi-bin/ca
Protein Coding	44	GC07M092487	10	https://www.genecards.org/cgi-bin/ca
Protein Coding	52	GC0XM020149	9.96	https://www.genecards.org/cgi-bin/ca
Protein Coding	43	GC05P140076	9.92	https://www.genecards.org/cgi-bin/ca
Protein Coding	37	GC05M140537	9.9	https://www.genecards.org/cgi-bin/ca
Protein Coding	31	GC19M049024	9.9	https://www.genecards.org/cgi-bin/ca
Protein Coding	43	GC04M173524	9.9	https://www.genecards.org/cgi-bin/ca
Protein Coding	41	GC17M064477	9.86	https://www.genecards.org/cgi-bin/ca
Protein Coding	45	GC05P075336	9.86	https://www.genecards.org/cgi-bin/ca
Protein Coding	40	GC02P061017	9.86	https://www.genecards.org/cgi-bin/ca
Protein Coding	42	GC08M143916	9.85	https://www.genecards.org/cgi-bin/ca
Protein Coding	44	GC01M011858	9.85	https://www.genecards.org/cgi-bin/ca
Protein Coding	45	GC08M133237	9.83	https://www.genecards.org/cgi-bin/ca
Protein Coding	44	GC09P005450	9.82	https://www.genecards.org/cgi-bin/ca
Protein Coding	51	GC11M102835	9.81	https://www.genecards.org/cgi-bin/ca
Protein Coding	49	GC13M077895	9.8	https://www.genecards.org/cgi-bin/ca
Protein Coding	21	GC16M054845	9.77	https://www.genecards.org/cgi-bin/ca
Protein Coding	39	GC0XM137030	9.76	https://www.genecards.org/cgi-bin/ca
Protein Coding	49	GC08M047773	9.74	https://www.genecards.org/cgi-bin/ca
Protein Coding	41	GC02M151485	9.74	https://www.genecards.org/cgi-bin/ca
Protein Coding	45	GC02P218880	9.71	https://www.genecards.org/cgi-bin/ca

Protein Coding	40	GC03P014124	9.71	https://www.genecards.org/cgi-bin/ca
Protein Coding	48	GC01P011980	9.69	https://www.genecards.org/cgi-bin/ca
Protein Coding	41	GC08P073003	9.68	https://www.genecards.org/cgi-bin/ca
Protein Coding	42	GC03M184371	9.67	https://www.genecards.org/cgi-bin/ca
Protein Coding	39	GC07M073568	9.66	https://www.genecards.org/cgi-bin/ca
Protein Coding	49	GC17M027756	9.66	https://www.genecards.org/cgi-bin/ca
Protein Coding	49	GC01P032292	9.65	https://www.genecards.org/cgi-bin/ca
Protein Coding	43	GC08M076980	9.65	https://www.genecards.org/cgi-bin/ca
Protein Coding	37	GC07P074289	9.64	https://www.genecards.org/cgi-bin/ca
Protein Coding	50	GC02P202376	9.63	https://www.genecards.org/cgi-bin/ca
Protein Coding	42	GC09P113221	9.6	https://www.genecards.org/cgi-bin/ca
Protein Coding	47	GC08M140657	9.59	https://www.genecards.org/cgi-bin/ca
Protein Coding	45	GC12P120978	9.59	https://www.genecards.org/cgi-bin/ca
Protein Coding	41	GC07M027259	9.54	https://www.genecards.org/cgi-bin/ca
Protein Coding	51	GC07P076302	9.54	https://www.genecards.org/cgi-bin/ca
Protein Coding	43	GC05P177134	9.53	https://www.genecards.org/cgi-bin/ca
Protein Coding	47	GC16P015949	9.5	https://www.genecards.org/cgi-bin/ca
Protein Coding	45	GC05M055935	9.49	https://www.genecards.org/cgi-bin/ca
Protein Coding	40	GC05M177992	9.49	https://www.genecards.org/cgi-bin/ca
Protein Coding	42	GC09P097853	9.48	https://www.genecards.org/cgi-bin/ca
Protein Coding	43	GC20M033675	9.47	https://www.genecards.org/cgi-bin/ca
Protein Coding	43	GC06M032635	9.46	https://www.genecards.org/cgi-bin/ca
Protein Coding	40	GC16P004428	9.45	https://www.genecards.org/cgi-bin/ca
Protein Coding	48	GC19P029811	9.45	https://www.genecards.org/cgi-bin/ca
Protein Coding	45	GC22M018912	9.45	https://www.genecards.org/cgi-bin/ca
Protein Coding	46	GC13M047745	9.43	https://www.genecards.org/cgi-bin/ca
Protein Coding	43	GC01P045500	9.43	https://www.genecards.org/cgi-bin/ca
Protein Coding	44	GC17M078356	9.43	https://www.genecards.org/cgi-bin/ca
Protein Coding	40	GC16M069355	9.42	https://www.genecards.org/cgi-bin/ca
Protein Coding	50	GC05M151639	9.4	https://www.genecards.org/cgi-bin/ca
Protein Coding	41	GC16M020344	9.4	https://www.genecards.org/cgi-bin/ca
Protein Coding	45	GC06P033620	9.39	https://www.genecards.org/cgi-bin/ca
Protein Coding	41	GC01P109687	9.37	https://www.genecards.org/cgi-bin/ca
Protein Coding	41	GC19P041709	9.34	https://www.genecards.org/cgi-bin/ca
Protein Coding	45	GC16M015704	9.32	https://www.genecards.org/cgi-bin/ca
Protein Coding	44	GC19P000840	9.3	https://www.genecards.org/cgi-bin/ca
Protein Coding	47	GC20P059300	9.28	https://www.genecards.org/cgi-bin/ca
Protein Coding	47	GC03M047033	9.28	https://www.genecards.org/cgi-bin/ca
Protein Coding	45	GC07P144938	9.26	https://www.genecards.org/cgi-bin/ca
Protein Coding	44	GC11M112143	9.25	https://www.genecards.org/cgi-bin/ca
Protein Coding	47	GC02M038981	9.24	https://www.genecards.org/cgi-bin/ca
Pseudogene	12	GC01P119563	9.23	https://www.genecards.org/cgi-bin/ca
Protein Coding	50	GC07M044145	9.23	https://www.genecards.org/cgi-bin/ca
Protein Coding	35	GC01P016798	9.22	https://www.genecards.org/cgi-bin/ca
Protein Coding	44	GC15P032720	9.22	https://www.genecards.org/cgi-bin/ca
Protein Coding	45	GC14M023380	9.22	https://www.genecards.org/cgi-bin/ca
Protein Coding	37	GC19P042325	9.2	https://www.genecards.org/cgi-bin/ca
Protein Coding	45	GC10P112950	9.19	https://www.genecards.org/cgi-bin/ca
Protein Coding	40	GC01M165724	9.15	https://www.genecards.org/cgi-bin/ca
Protein Coding	47	GC18P000657	9.14	https://www.genecards.org/cgi-bin/ca
Protein Coding	45	GC21M047431	9.14	https://www.genecards.org/cgi-bin/ca
Protein Coding	47	GC11P072190	9.12	https://www.genecards.org/cgi-bin/ca
Protein Coding	45	GC07P024290	9.09	https://www.genecards.org/cgi-bin/ca
Protein Coding	46	GC19M048115	9.08	https://www.genecards.org/cgi-bin/ca

Protein Coding	48	GC16P050693	9.07	https://www.genecards.org/cgi-bin/ca
Protein Coding	50	GC20M056370	9.06	https://www.genecards.org/cgi-bin/ca
Protein Coding	47	GC01M155234	9.04	https://www.genecards.org/cgi-bin/ca
Protein Coding	46	GC12P049950	9.03	https://www.genecards.org/cgi-bin/ca
Protein Coding	46	GC11P061811	9.03	https://www.genecards.org/cgi-bin/ca
Protein Coding	46	GC11P060474	9.01	https://www.genecards.org/cgi-bin/ca
Protein Coding	46	GC0XM153864	9.01	https://www.genecards.org/cgi-bin/ca
Protein Coding	48	GC01M015491	9.01	https://www.genecards.org/cgi-bin/ca
Protein Coding	45	GC17P001829	9	https://www.genecards.org/cgi-bin/ca
Protein Coding	40	GC01P054998	8.99	https://www.genecards.org/cgi-bin/ca
Protein Coding	45	GC16P067433	8.96	https://www.genecards.org/cgi-bin/ca
Protein Coding	37	GC06P047481	8.95	https://www.genecards.org/cgi-bin/ca
Protein Coding	41	GC21M043092	8.92	https://www.genecards.org/cgi-bin/ca
Protein Coding	47	GC17P056593	8.92	https://www.genecards.org/cgi-bin/ca
Protein Coding	48	GC19P050329	8.92	https://www.genecards.org/cgi-bin/ca
Protein Coding	44	GC04M121816	8.91	https://www.genecards.org/cgi-bin/ca
Protein Coding	43	GC19M002439	8.9	https://www.genecards.org/cgi-bin/ca
Protein Coding	41	GC02M108894	8.89	https://www.genecards.org/cgi-bin/ca
Protein Coding	45	GC07M095297	8.89	https://www.genecards.org/cgi-bin/ca
Protein Coding	38	GC20P045469	8.88	https://www.genecards.org/cgi-bin/ca
Protein Coding	45	GC22P032800	8.88	https://www.genecards.org/cgi-bin/ca
Protein Coding	32	GCMTP003309	8.85	https://www.genecards.org/cgi-bin/ca
Protein Coding	37	GC08P098938	8.84	https://www.genecards.org/cgi-bin/ca
Protein Coding	41	GC09M114060	8.84	https://www.genecards.org/cgi-bin/ca
Protein Coding	45	GC02M168922	8.84	https://www.genecards.org/cgi-bin/ca
Protein Coding	48	GC02P233760	8.83	https://www.genecards.org/cgi-bin/ca
Protein Coding	47	GC05M038475	8.78	https://www.genecards.org/cgi-bin/ca
Protein Coding	47	GC01M150707	8.76	https://www.genecards.org/cgi-bin/ca
Protein Coding	43	GC22P029269	8.76	https://www.genecards.org/cgi-bin/ca
Protein Coding	44	GC18P022798	8.75	https://www.genecards.org/cgi-bin/ca
Protein Coding	50	GC17P066302	8.71	https://www.genecards.org/cgi-bin/ca
Protein Coding	41	GC01P011934	8.71	https://www.genecards.org/cgi-bin/ca
Protein Coding	44	GC14P055124	8.7	https://www.genecards.org/cgi-bin/ca
Protein Coding	35	GC06M049430	8.69	https://www.genecards.org/cgi-bin/ca
Protein Coding	44	GC11M069811	8.67	https://www.genecards.org/cgi-bin/ca
Protein Coding	51	GC22P023179	8.65	https://www.genecards.org/cgi-bin/ca
Protein Coding	43	GC01P241847	8.64	https://www.genecards.org/cgi-bin/ca
Protein Coding	50	GC04M088090	8.63	https://www.genecards.org/cgi-bin/ca
Protein Coding	41	GC07M073593	8.63	https://www.genecards.org/cgi-bin/ca
Protein Coding	43	GC06P128863	8.62	https://www.genecards.org/cgi-bin/ca
Protein Coding	43	GC06P047326	8.62	https://www.genecards.org/cgi-bin/ca
Protein Coding	41	GC12P065169	8.61	https://www.genecards.org/cgi-bin/ca
Protein Coding	43	GC06P109691	8.61	https://www.genecards.org/cgi-bin/ca
Protein Coding	40	GC0XM077826	8.61	https://www.genecards.org/cgi-bin/ca
Protein Coding	41	GC12M053307	8.6	https://www.genecards.org/cgi-bin/ca
Protein Coding	44	GC11M069762	8.58	https://www.genecards.org/cgi-bin/ca
Protein Coding	50	GC17M040388	8.55	https://www.genecards.org/cgi-bin/ca
Protein Coding	51	GC20M044620	8.55	https://www.genecards.org/cgi-bin/ca
Protein Coding	44	GC20P049982	8.55	https://www.genecards.org/cgi-bin/ca
Protein Coding	34	GC11M129899	8.53	https://www.genecards.org/cgi-bin/ca
Protein Coding	49	GC01P209686	8.53	https://www.genecards.org/cgi-bin/ca
Protein Coding	49	GC08M042174	8.51	https://www.genecards.org/cgi-bin/ca
Protein Coding	49	GC22P023894	8.51	https://www.genecards.org/cgi-bin/ca
Protein Coding	47	GC12M122208	8.51	https://www.genecards.org/cgi-bin/ca

Protein Coding	47	GC05P069167	8.5	https://www.genecards.org/cgi-bin/ca
Protein Coding	40	GC19P040592	8.5	https://www.genecards.org/cgi-bin/ca
RNA Gene	20	GC19M014011	8.49	https://www.genecards.org/cgi-bin/ca
Protein Coding	47	GC01M225401	8.48	https://www.genecards.org/cgi-bin/ca
Protein Coding	38	GC03M195746	8.45	https://www.genecards.org/cgi-bin/ca
Protein Coding	47	GC17P051154	8.44	https://www.genecards.org/cgi-bin/ca
Protein Coding	47	GC03M149162	8.43	https://www.genecards.org/cgi-bin/ca
Protein Coding	45	GC03P057227	8.43	https://www.genecards.org/cgi-bin/ca
Protein Coding	52	GC16M003726	8.43	https://www.genecards.org/cgi-bin/ca
Protein Coding	47	GC11P102110	8.42	https://www.genecards.org/cgi-bin/ca
Protein Coding	39	GC22M050217	8.42	https://www.genecards.org/cgi-bin/ca
Protein Coding	48	GC17P075721	8.39	https://www.genecards.org/cgi-bin/ca
Protein Coding	47	GC02P026243	8.34	https://www.genecards.org/cgi-bin/ca
Protein Coding	47	GC18P022169	8.33	https://www.genecards.org/cgi-bin/ca
Protein Coding	44	GC06M032804	8.29	https://www.genecards.org/cgi-bin/ca
Protein Coding	44	GC0XP007146	8.29	https://www.genecards.org/cgi-bin/ca
Protein Coding	46	GC01M011846	8.28	https://www.genecards.org/cgi-bin/ca
Protein Coding	45	GC16M016148	8.28	https://www.genecards.org/cgi-bin/ca
Genetic Locus	2	GC0XU901813	8.27	https://www.genecards.org/cgi-bin/ca
Protein Coding	45	GC02P210477	8.27	https://www.genecards.org/cgi-bin/ca
Protein Coding	41	GC06M010873	8.27	https://www.genecards.org/cgi-bin/ca
Protein Coding	45	GC12P008238	8.26	https://www.genecards.org/cgi-bin/ca
Protein Coding	51	GC11M002163	8.26	https://www.genecards.org/cgi-bin/ca
Protein Coding	45	GC01M169558	8.25	https://www.genecards.org/cgi-bin/ca
Protein Coding	48	GC10P031318	8.25	https://www.genecards.org/cgi-bin/ca
Protein Coding	45	GC11M125955	8.25	https://www.genecards.org/cgi-bin/ca
Protein Coding	52	GC07M050458	8.22	https://www.genecards.org/cgi-bin/ca
Protein Coding	39	GC0XM074587	8.22	https://www.genecards.org/cgi-bin/ca
Protein Coding	50	GC04P094757	8.22	https://www.genecards.org/cgi-bin/ca
Protein Coding	47	GC10P073995	8.21	https://www.genecards.org/cgi-bin/ca
Protein Coding	50	GC18M020946	8.21	https://www.genecards.org/cgi-bin/ca
Protein Coding	44	GC21M036460	8.21	https://www.genecards.org/cgi-bin/ca
Protein Coding	43	GC18M048919	8.2	https://www.genecards.org/cgi-bin/ca
Protein Coding	48	GC02P115307	8.2	https://www.genecards.org/cgi-bin/ca
Protein Coding	44	GC15M089664	8.2	https://www.genecards.org/cgi-bin/ca
Protein Coding	44	GC11M018040	8.19	https://www.genecards.org/cgi-bin/ca
Protein Coding	49	GC02M110637	8.19	https://www.genecards.org/cgi-bin/ca
Protein Coding	45	GC14M024249	8.18	https://www.genecards.org/cgi-bin/ca
Protein Coding	42	GC17P044004	8.18	https://www.genecards.org/cgi-bin/ca
Protein Coding	45	GC09P133414	8.17	https://www.genecards.org/cgi-bin/ca
Protein Coding	46	GC22M019863	8.17	https://www.genecards.org/cgi-bin/ca
Protein Coding	51	GC01P198607	8.15	https://www.genecards.org/cgi-bin/ca
Protein Coding	41	GC03P190290	8.15	https://www.genecards.org/cgi-bin/ca
Protein Coding	44	GC03M050329	8.15	https://www.genecards.org/cgi-bin/ca
Protein Coding	47	GC17P080101	8.14	https://www.genecards.org/cgi-bin/ca
Protein Coding	44	GC05P036103	8.14	https://www.genecards.org/cgi-bin/ca
Protein Coding	37	GC14P094104	8.13	https://www.genecards.org/cgi-bin/ca
Protein Coding	37	GC08P076681	8.09	https://www.genecards.org/cgi-bin/ca
Protein Coding	45	GC11M017392	8.09	https://www.genecards.org/cgi-bin/ca
Protein Coding	44	GC06P152750	8.08	https://www.genecards.org/cgi-bin/ca
Protein Coding	45	GC01M021822	8.08	https://www.genecards.org/cgi-bin/ca
Protein Coding	47	GC03P069788	8.07	https://www.genecards.org/cgi-bin/ca
Protein Coding	45	GC11M111908	8.06	https://www.genecards.org/cgi-bin/ca
Protein Coding	39	GC01M156668	8.03	https://www.genecards.org/cgi-bin/ca

Protein Coding	47	GC02P203706	8.02 https://www.genecards.org/cgi-bin/ca
Protein Coding	45	GC11P075562	7.99 https://www.genecards.org/cgi-bin/ca
Protein Coding	44	GC21M042362	7.99 https://www.genecards.org/cgi-bin/ca
Protein Coding	42	GC02M214931	7.99 https://www.genecards.org/cgi-bin/ca
Protein Coding	50	GC17M058269	7.97 https://www.genecards.org/cgi-bin/ca
Protein Coding	43	GC04P073869	7.97 https://www.genecards.org/cgi-bin/ca
Protein Coding	39	GC07M128298	7.97 https://www.genecards.org/cgi-bin/ca
Protein Coding	45	GC13P102808	7.96 https://www.genecards.org/cgi-bin/ca
Protein Coding	47	GC01P111542	7.96 https://www.genecards.org/cgi-bin/ca
Protein Coding	41	GC08P069466	7.96 https://www.genecards.org/cgi-bin/ca
Protein Coding	48	GC05M138285	7.95 https://www.genecards.org/cgi-bin/ca
Protein Coding	50	GC14P076310	7.94 https://www.genecards.org/cgi-bin/ca
Protein Coding	44	GC09M032886	7.94 https://www.genecards.org/cgi-bin/ca
Protein Coding	49	GC09P001980	7.92 https://www.genecards.org/cgi-bin/ca
Protein Coding	51	GC11M086945	7.92 https://www.genecards.org/cgi-bin/ca
Protein Coding	45	GC03M052222	7.89 https://www.genecards.org/cgi-bin/ca
Protein Coding	45	GC10P110567	7.87 https://www.genecards.org/cgi-bin/ca
Protein Coding	44	GC12M032725	7.87 https://www.genecards.org/cgi-bin/ca
Protein Coding	40	GC18M057646	7.87 https://www.genecards.org/cgi-bin/ca
Protein Coding	45	GC01P100719	7.87 https://www.genecards.org/cgi-bin/ca
Protein Coding	48	GC10P045374	7.85 https://www.genecards.org/cgi-bin/ca
Protein Coding	47	GC05P138613	7.85 https://www.genecards.org/cgi-bin/ca
Protein Coding	44	GC14P104663	7.85 https://www.genecards.org/cgi-bin/ca
Protein Coding	45	GC20P047501	7.84 https://www.genecards.org/cgi-bin/ca
Protein Coding	47	GC16M008892	7.83 https://www.genecards.org/cgi-bin/ca
Protein Coding	48	GC20M038127	7.83 https://www.genecards.org/cgi-bin/ca
Protein Coding	45	GC22P046150	7.82 https://www.genecards.org/cgi-bin/ca
Protein Coding	48	GC02M144384	7.8 https://www.genecards.org/cgi-bin/ca
Protein Coding	40	GC19M050977	7.79 https://www.genecards.org/cgi-bin/ca
Protein Coding	45	GC19M029205	7.79 https://www.genecards.org/cgi-bin/ca
Protein Coding	45	GC01P003652	7.78 https://www.genecards.org/cgi-bin/ca
RNA Gene	20	GC03M052296	7.78 https://www.genecards.org/cgi-bin/ca
Protein Coding	40	GC17M058205	7.77 https://www.genecards.org/cgi-bin/ca
Protein Coding	39	GC06M051588	7.76 https://www.genecards.org/cgi-bin/ca
Protein Coding	48	GC15P044711	7.75 https://www.genecards.org/cgi-bin/ca
Protein Coding	37	GC03M147386	7.74 https://www.genecards.org/cgi-bin/ca
Protein Coding	48	GC15M060347	7.72 https://www.genecards.org/cgi-bin/ca
Protein Coding	40	GC12M013865	7.72 https://www.genecards.org/cgi-bin/ca
Protein Coding	47	GC10M133362	7.72 https://www.genecards.org/cgi-bin/ca
Genetic Locus	3	GC02U903086	7.7 https://www.genecards.org/cgi-bin/ca
Protein Coding	42	GC08M052622	7.69 https://www.genecards.org/cgi-bin/ca
Protein Coding	38	GC19M049015	7.68 https://www.genecards.org/cgi-bin/ca
Protein Coding	42	GC01P206897	7.67 https://www.genecards.org/cgi-bin/ca
Protein Coding	45	GC14M103519	7.67 https://www.genecards.org/cgi-bin/ca
Protein Coding	46	GC10M071816	7.67 https://www.genecards.org/cgi-bin/ca
Protein Coding	47	GC10P049609	7.67 https://www.genecards.org/cgi-bin/ca
Protein Coding	42	GC03P129974	7.66 https://www.genecards.org/cgi-bin/ca
Protein Coding	48	GC02M241849	7.66 https://www.genecards.org/cgi-bin/ca
Protein Coding	52	GC08P042271	7.65 https://www.genecards.org/cgi-bin/ca
Protein Coding	44	GC18M076978	7.65 https://www.genecards.org/cgi-bin/ca
Protein Coding	51	GC15M087859	7.63 https://www.genecards.org/cgi-bin/ca
Protein Coding	49	GC01P065421	7.63 https://www.genecards.org/cgi-bin/ca
Protein Coding	45	GC01M154962	7.63 https://www.genecards.org/cgi-bin/ca
Protein Coding	46	GC16P067563	7.62 https://www.genecards.org/cgi-bin/ca

Protein Coding	49	GC07M150944	7.62	https://www.genecards.org/cgi-bin/ca
Protein Coding	42	GC11P066002	7.61	https://www.genecards.org/cgi-bin/ca
Protein Coding	46	GC05P076716	7.59	https://www.genecards.org/cgi-bin/ca
Protein Coding	48	GC07P080369	7.58	https://www.genecards.org/cgi-bin/ca
Protein Coding	45	GC03P136250	7.53	https://www.genecards.org/cgi-bin/ca
Protein Coding	44	GC19P041398	7.53	https://www.genecards.org/cgi-bin/ca
Protein Coding	48	GC02M177227	7.52	https://www.genecards.org/cgi-bin/ca
Protein Coding	50	GC0XM154531	7.51	https://www.genecards.org/cgi-bin/ca
Protein Coding	51	GC01P162631	7.5	https://www.genecards.org/cgi-bin/ca
Protein Coding	43	GC02M216672	7.48	https://www.genecards.org/cgi-bin/ca
Protein Coding	34	GC03M020159	7.47	https://www.genecards.org/cgi-bin/ca
Protein Coding	45	GC02M058127	7.46	https://www.genecards.org/cgi-bin/ca
RNA Gene	18	GC09P095086	7.46	https://www.genecards.org/cgi-bin/ca
Protein Coding	39	GC04P119212	7.45	https://www.genecards.org/cgi-bin/ca
Protein Coding	41	GC15P089243	7.45	https://www.genecards.org/cgi-bin/ca
Protein Coding	45	GC01M216503	7.44	https://www.genecards.org/cgi-bin/ca
Protein Coding	47	GC15M025333	7.44	https://www.genecards.org/cgi-bin/ca
Protein Coding	39	GC08P023102	7.43	https://www.genecards.org/cgi-bin/ca
Protein Coding	50	GC17P040309	7.43	https://www.genecards.org/cgi-bin/ca
Protein Coding	42	GC06P047303	7.42	https://www.genecards.org/cgi-bin/ca
RNA Gene	14	GC03M139232	7.42	https://www.genecards.org/cgi-bin/ca
Protein Coding	48	GC04M148078	7.41	https://www.genecards.org/cgi-bin/ca
Protein Coding	47	GC06M041934	7.41	https://www.genecards.org/cgi-bin/ca
Protein Coding	46	GC19P050854	7.4	https://www.genecards.org/cgi-bin/ca
Protein Coding	39	GC03P137764	7.39	https://www.genecards.org/cgi-bin/ca
RNA Gene	20	GC03P139104	7.39	https://www.genecards.org/cgi-bin/ca
RNA Gene	12	GC15M096772	7.39	https://www.genecards.org/cgi-bin/ca
Protein Coding	47	GC13P027921	7.39	https://www.genecards.org/cgi-bin/ca
Protein Coding	43	GC05P007851	7.39	https://www.genecards.org/cgi-bin/ca
Protein Coding	42	GC01M169690	7.39	https://www.genecards.org/cgi-bin/ca
Protein Coding	48	GC02P207529	7.39	https://www.genecards.org/cgi-bin/ca
Protein Coding	48	GC15M055202	7.38	https://www.genecards.org/cgi-bin/ca
Protein Coding	48	GC07M106248	7.36	https://www.genecards.org/cgi-bin/ca
Protein Coding	41	GC01M179519	7.36	https://www.genecards.org/cgi-bin/ca
Protein Coding	45	GC06M170282	7.36	https://www.genecards.org/cgi-bin/ca
Protein Coding	41	GC09M021384	7.34	https://www.genecards.org/cgi-bin/ca
Protein Coding	48	GC12P100473	7.34	https://www.genecards.org/cgi-bin/ca
Protein Coding	41	GC19M050943	7.33	https://www.genecards.org/cgi-bin/ca
Protein Coding	44	GC09M109375	7.33	https://www.genecards.org/cgi-bin/ca
Protein Coding	40	GC16P071392	7.32	https://www.genecards.org/cgi-bin/ca
Protein Coding	39	GC0XM072255	7.31	https://www.genecards.org/cgi-bin/ca
Protein Coding	43	GC11P117199	7.3	https://www.genecards.org/cgi-bin/ca
Protein Coding	42	GC19M050907	7.29	https://www.genecards.org/cgi-bin/ca
Protein Coding	43	GC04P074445	7.29	https://www.genecards.org/cgi-bin/ca
Protein Coding	43	GC01M151364	7.29	https://www.genecards.org/cgi-bin/ca
Protein Coding	47	GC07M134442	7.29	https://www.genecards.org/cgi-bin/ca
RNA Gene	23	GC01M173947	7.28	https://www.genecards.org/cgi-bin/ca
Protein Coding	43	GC06P080106	7.28	https://www.genecards.org/cgi-bin/ca
Protein Coding	32	GC02P232407	7.25	https://www.genecards.org/cgi-bin/ca
Protein Coding	47	GC19P003094	7.25	https://www.genecards.org/cgi-bin/ca
Protein Coding	41	GC06P047436	7.23	https://www.genecards.org/cgi-bin/ca
Protein Coding	47	GC03P050226	7.22	https://www.genecards.org/cgi-bin/ca
Protein Coding	39	GC17M075626	7.21	https://www.genecards.org/cgi-bin/ca
Protein Coding	45	GC10M070597	7.21	https://www.genecards.org/cgi-bin/ca

Protein Coding	51	GC10P050991	7.2	https://www.genecards.org/cgi-bin/ca
Protein Coding	41	GC19M007958	7.19	https://www.genecards.org/cgi-bin/ca
Protein Coding	52	GC05M180607	7.19	https://www.genecards.org/cgi-bin/ca
Protein Coding	37	GC14P067819	7.19	https://www.genecards.org/cgi-bin/ca
Protein Coding	47	GC01P207321	7.18	https://www.genecards.org/cgi-bin/ca
Protein Coding	47	GC17M049404	7.17	https://www.genecards.org/cgi-bin/ca
Protein Coding	31	GCMTP008531	7.17	https://www.genecards.org/cgi-bin/ca
Protein Coding	43	GC13M037562	7.15	https://www.genecards.org/cgi-bin/ca
Protein Coding	41	GC12M049018	7.15	https://www.genecards.org/cgi-bin/ca
Protein Coding	42	GC12M014881	7.14	https://www.genecards.org/cgi-bin/ca
Protein Coding	32	GC0XM154617	7.14	https://www.genecards.org/cgi-bin/ca
Protein Coding	50	GC03P038179	7.13	https://www.genecards.org/cgi-bin/ca
Protein Coding	47	GC01P225810	7.12	https://www.genecards.org/cgi-bin/ca
Protein Coding	41	GC10M098457	7.11	https://www.genecards.org/cgi-bin/ca
RNA Gene	20	GC22P046119	7.11	https://www.genecards.org/cgi-bin/ca
Protein Coding	44	GC11M102862	7.1	https://www.genecards.org/cgi-bin/ca
Protein Coding	46	GC05P000208	7.1	https://www.genecards.org/cgi-bin/ca
Protein Coding	44	GC04P105145	7.09	https://www.genecards.org/cgi-bin/ca
Protein Coding	47	GC11M031784	7.08	https://www.genecards.org/cgi-bin/ca
Protein Coding	47	GC12M006346	7.07	https://www.genecards.org/cgi-bin/ca
Protein Coding	45	GC13P100089	7.06	https://www.genecards.org/cgi-bin/ca
Protein Coding	47	GC04P026165	7.05	https://www.genecards.org/cgi-bin/ca
RNA Gene	20	GC11P057641	7.04	https://www.genecards.org/cgi-bin/ca
Protein Coding	42	GC11M005372	7.04	https://www.genecards.org/cgi-bin/ca
Protein Coding	41	GC11P001244	7.04	https://www.genecards.org/cgi-bin/ca
Protein Coding	48	GC17M008864	7.04	https://www.genecards.org/cgi-bin/ca
Protein Coding	44	GC0XM120426	7.03	https://www.genecards.org/cgi-bin/ca
Protein Coding	41	GC02P176092	7.02	https://www.genecards.org/cgi-bin/ca
Protein Coding	41	GC09M021077	7.01	https://www.genecards.org/cgi-bin/ca
Protein Coding	46	GC0XP139530	7	https://www.genecards.org/cgi-bin/ca
Protein Coding	39	GC11M061140	7	https://www.genecards.org/cgi-bin/ca
Protein Coding	41	GC03P046576	7	https://www.genecards.org/cgi-bin/ca
Protein Coding	43	GC02M190189	6.97	https://www.genecards.org/cgi-bin/ca
Protein Coding	42	GC01M023527	6.97	https://www.genecards.org/cgi-bin/ca
Protein Coding	44	GC01M068428	6.97	https://www.genecards.org/cgi-bin/ca
Protein Coding	40	GC12M013857	6.96	https://www.genecards.org/cgi-bin/ca
Genetic Locus	4	GC02U990267	6.96	https://www.genecards.org/cgi-bin/ca
Protein Coding	48	GC13P113122	6.94	https://www.genecards.org/cgi-bin/ca
Protein Coding	43	GC05P143096	6.94	https://www.genecards.org/cgi-bin/ca
Protein Coding	45	GC0XM154835	6.93	https://www.genecards.org/cgi-bin/ca
Protein Coding	47	GC01M223109	6.9	https://www.genecards.org/cgi-bin/ca
Protein Coding	44	GC11P118436	6.9	https://www.genecards.org/cgi-bin/ca
Protein Coding	43	GC09M035073	6.89	https://www.genecards.org/cgi-bin/ca
Protein Coding	39	GC03M158095	6.89	https://www.genecards.org/cgi-bin/ca
Protein Coding	40	GC17P041812	6.89	https://www.genecards.org/cgi-bin/ca
Protein Coding	52	GC14P073136	6.88	https://www.genecards.org/cgi-bin/ca
Protein Coding	47	GC05P035852	6.86	https://www.genecards.org/cgi-bin/ca
Protein Coding	45	GC04P070992	6.86	https://www.genecards.org/cgi-bin/ca
Protein Coding	44	GC05M132541	6.85	https://www.genecards.org/cgi-bin/ca
Protein Coding	50	GC01M115285	6.84	https://www.genecards.org/cgi-bin/ca
Protein Coding	48	GC0XM101393	6.84	https://www.genecards.org/cgi-bin/ca
Protein Coding	47	GC18P079395	6.84	https://www.genecards.org/cgi-bin/ca
Protein Coding	41	GC0XM138615	6.83	https://www.genecards.org/cgi-bin/ca
Protein Coding	43	GC0XP153494	6.82	https://www.genecards.org/cgi-bin/ca

Protein Coding	47	GC04M154583	6.81	https://www.genecards.org/cgi-bin/ca
Protein Coding	46	GC11M071428	6.81	https://www.genecards.org/cgi-bin/ca
Protein Coding	37	GC03P093980	6.81	https://www.genecards.org/cgi-bin/ca
Protein Coding	42	GC16P001373	6.81	https://www.genecards.org/cgi-bin/ca
Protein Coding	42	GC02P043828	6.8	https://www.genecards.org/cgi-bin/ca
Protein Coding	44	GC03M048564	6.79	https://www.genecards.org/cgi-bin/ca
Protein Coding	40	GC05P036876	6.79	https://www.genecards.org/cgi-bin/ca
Protein Coding	40	GC13P020566	6.78	https://www.genecards.org/cgi-bin/ca
Protein Coding	48	GC05M035048	6.78	https://www.genecards.org/cgi-bin/ca
Protein Coding	30	GC07P073685	6.77	https://www.genecards.org/cgi-bin/ca
Protein Coding	46	GC16P031550	6.77	https://www.genecards.org/cgi-bin/ca
RNA Gene	23	GC15P079209	6.76	https://www.genecards.org/cgi-bin/ca
Protein Coding	36	GC12P055718	6.74	https://www.genecards.org/cgi-bin/ca
Protein Coding	47	GC01P012167	6.74	https://www.genecards.org/cgi-bin/ca
Protein Coding	40	GC06M100386	6.74	https://www.genecards.org/cgi-bin/ca
Protein Coding	47	GC17M030194	6.74	https://www.genecards.org/cgi-bin/ca
Protein Coding	52	GC22P035380	6.73	https://www.genecards.org/cgi-bin/ca
Protein Coding	39	GC12P123671	6.73	https://www.genecards.org/cgi-bin/ca
Protein Coding	45	GC12P008144	6.7	https://www.genecards.org/cgi-bin/ca
Protein Coding	40	GC05P076952	6.7	https://www.genecards.org/cgi-bin/ca
Protein Coding	47	GC13M020187	6.7	https://www.genecards.org/cgi-bin/ca
Protein Coding	43	GC06M070215	6.69	https://www.genecards.org/cgi-bin/ca
RNA Gene	16	GC08M041660	6.69	https://www.genecards.org/cgi-bin/ca
Protein Coding	45	GC11P036514	6.69	https://www.genecards.org/cgi-bin/ca
Protein Coding	45	GC16M001444	6.68	https://www.genecards.org/cgi-bin/ca
Protein Coding	47	GC02P218781	6.68	https://www.genecards.org/cgi-bin/ca
Protein Coding	44	GC18M074250	6.68	https://www.genecards.org/cgi-bin/ca
Protein Coding	40	GC11P111913	6.67	https://www.genecards.org/cgi-bin/ca
Protein Coding	33	GC0YP009469	6.67	https://www.genecards.org/cgi-bin/ca
Protein Coding	45	GC15P074748	6.65	https://www.genecards.org/cgi-bin/ca
Protein Coding	42	GC01P012063	6.65	https://www.genecards.org/cgi-bin/ca
Protein Coding	45	GC09P126614	6.64	https://www.genecards.org/cgi-bin/ca
Protein Coding	44	GC10M014897	6.64	https://www.genecards.org/cgi-bin/ca
Protein Coding	46	GC07P002512	6.64	https://www.genecards.org/cgi-bin/ca
Protein Coding	41	GC06M160531	6.63	https://www.genecards.org/cgi-bin/ca
Protein Coding	47	GC0XP124227	6.62	https://www.genecards.org/cgi-bin/ca
Protein Coding	45	GC11M065823	6.61	https://www.genecards.org/cgi-bin/ca
Protein Coding	45	GC12M120210	6.6	https://www.genecards.org/cgi-bin/ca
Protein Coding	31	GC01P054643	6.59	https://www.genecards.org/cgi-bin/ca
Protein Coding	42	GC11P018267	6.59	https://www.genecards.org/cgi-bin/ca
Protein Coding	44	GC09M035672	6.58	https://www.genecards.org/cgi-bin/ca
Protein Coding	47	GC18M023506	6.58	https://www.genecards.org/cgi-bin/ca
Protein Coding	53	GC09P084668	6.58	https://www.genecards.org/cgi-bin/ca
Protein Coding	43	GC0XM075053	6.58	https://www.genecards.org/cgi-bin/ca
Protein Coding	45	GC11P057788	6.56	https://www.genecards.org/cgi-bin/ca
Protein Coding	40	GC12P085876	6.56	https://www.genecards.org/cgi-bin/ca
Protein Coding	46	GC0XP048786	6.56	https://www.genecards.org/cgi-bin/ca
Protein Coding	49	GC17M075318	6.55	https://www.genecards.org/cgi-bin/ca
Protein Coding	42	GC11P065883	6.55	https://www.genecards.org/cgi-bin/ca
Protein Coding	39	GC01M197084	6.54	https://www.genecards.org/cgi-bin/ca
Protein Coding	40	GC04P141636	6.54	https://www.genecards.org/cgi-bin/ca
Protein Coding	48	GC07M151466	6.53	https://www.genecards.org/cgi-bin/ca
Protein Coding	43	GC22P041833	6.53	https://www.genecards.org/cgi-bin/ca
Protein Coding	46	GC01P228082	6.53	https://www.genecards.org/cgi-bin/ca

Protein Coding	48	GC09P130444	6.52	https://www.genecards.org/cgi-bin/ca
Protein Coding	40	GC02P233691	6.51	https://www.genecards.org/cgi-bin/ca
Protein Coding	52	GC15M090083	6.5	https://www.genecards.org/cgi-bin/ca
Protein Coding	34	GC0XP003006	6.5	https://www.genecards.org/cgi-bin/ca
RNA Gene	18	GC01M071067	6.5	https://www.genecards.org/cgi-bin/ca
Protein Coding	44	GC10M116056	6.49	https://www.genecards.org/cgi-bin/ca
Protein Coding	44	GC16M051135	6.46	https://www.genecards.org/cgi-bin/ca
Protein Coding	45	GC02M169127	6.46	https://www.genecards.org/cgi-bin/ca
Protein Coding	38	GC11P066499	6.46	https://www.genecards.org/cgi-bin/ca
Protein Coding	40	GC17P041712	6.46	https://www.genecards.org/cgi-bin/ca
Protein Coding	48	GC0XP114584	6.46	https://www.genecards.org/cgi-bin/ca
Protein Coding	48	GC12P001970	6.44	https://www.genecards.org/cgi-bin/ca
Protein Coding	35	GC11M077659	6.43	https://www.genecards.org/cgi-bin/ca
Protein Coding	41	GC15M023686	6.43	https://www.genecards.org/cgi-bin/ca
Protein Coding	42	GC02P232525	6.42	https://www.genecards.org/cgi-bin/ca
Protein Coding	43	GC0XM049187	6.4	https://www.genecards.org/cgi-bin/ca
Protein Coding	33	GC01P200739	6.4	https://www.genecards.org/cgi-bin/ca
Protein Coding	41	GC05P070924	6.4	https://www.genecards.org/cgi-bin/ca
Protein Coding	45	GC07M083955	6.38	https://www.genecards.org/cgi-bin/ca
Protein Coding	40	GC09P036036	6.38	https://www.genecards.org/cgi-bin/ca
Protein Coding	45	GC18P062325	6.37	https://www.genecards.org/cgi-bin/ca
Protein Coding	37	GC03P052350	6.36	https://www.genecards.org/cgi-bin/ca
Protein Coding	45	GC12M008602	6.36	https://www.genecards.org/cgi-bin/ca
Protein Coding	44	GC10M093591	6.36	https://www.genecards.org/cgi-bin/ca
Protein Coding	46	GC22P039525	6.35	https://www.genecards.org/cgi-bin/ca
Protein Coding	45	GC0XP047562	6.35	https://www.genecards.org/cgi-bin/ca
Protein Coding	45	GC01M147756	6.35	https://www.genecards.org/cgi-bin/ca
Protein Coding	45	GC15P073994	6.35	https://www.genecards.org/cgi-bin/ca
Protein Coding	45	GC01P011013	6.34	https://www.genecards.org/cgi-bin/ca
Protein Coding	39	GC17M036088	6.34	https://www.genecards.org/cgi-bin/ca
Protein Coding	43	GC15M063608	6.34	https://www.genecards.org/cgi-bin/ca
Protein Coding	29	GC10M087860	6.34	https://www.genecards.org/cgi-bin/ca
Protein Coding	39	GC02P169480	6.32	https://www.genecards.org/cgi-bin/ca
Protein Coding	39	GC12P123633	6.3	https://www.genecards.org/cgi-bin/ca
Protein Coding	45	GC02P073892	6.29	https://www.genecards.org/cgi-bin/ca
Protein Coding	47	GC02M031294	6.29	https://www.genecards.org/cgi-bin/ca
Protein Coding	41	GC19M012684	6.27	https://www.genecards.org/cgi-bin/ca
Protein Coding	35	GC0XM044146	6.27	https://www.genecards.org/cgi-bin/ca
Protein Coding	50	GC03M119821	6.27	https://www.genecards.org/cgi-bin/ca
Protein Coding	48	GC06P137866	6.27	https://www.genecards.org/cgi-bin/ca
Protein Coding	44	GC05P132656	6.26	https://www.genecards.org/cgi-bin/ca
Protein Coding	48	GC05P148825	6.25	https://www.genecards.org/cgi-bin/ca
RNA Gene	18	GC13P091432	6.25	https://www.genecards.org/cgi-bin/ca
Protein Coding	45	GC17P007295	6.24	https://www.genecards.org/cgi-bin/ca
Protein Coding	46	GC10P026216	6.23	https://www.genecards.org/cgi-bin/ca
Protein Coding	45	GC02P102136	6.23	https://www.genecards.org/cgi-bin/ca
Protein Coding	36	GC07P030595	6.22	https://www.genecards.org/cgi-bin/ca
Protein Coding	45	GC18P003411	6.21	https://www.genecards.org/cgi-bin/ca
Protein Coding	45	GC15M072199	6.21	https://www.genecards.org/cgi-bin/ca
Protein Coding	41	GC02M001635	6.2	https://www.genecards.org/cgi-bin/ca
RNA Gene	17	GC14P104814	6.19	https://www.genecards.org/cgi-bin/ca
Protein Coding	41	GC04M079406	6.19	https://www.genecards.org/cgi-bin/ca
Protein Coding	33	GC16P011250	6.19	https://www.genecards.org/cgi-bin/ca
Protein Coding	42	GC15M082536	6.18	https://www.genecards.org/cgi-bin/ca

Protein Coding	35	GC02P233618	6.18	https://www.genecards.org/cgi-bin/ca
Protein Coding	44	GC19M015236	6.18	https://www.genecards.org/cgi-bin/ca
Protein Coding	44	GC06M166929	6.18	https://www.genecards.org/cgi-bin/ca
Protein Coding	37	GC17M008120	6.17	https://www.genecards.org/cgi-bin/ca
Protein Coding	47	GC07P044807	6.17	https://www.genecards.org/cgi-bin/ca
Protein Coding	45	GC05P096935	6.16	https://www.genecards.org/cgi-bin/ca
Protein Coding	42	GC17P042680	6.15	https://www.genecards.org/cgi-bin/ca
Protein Coding	44	GC06M032180	6.15	https://www.genecards.org/cgi-bin/ca
Protein Coding	46	GC18M055222	6.14	https://www.genecards.org/cgi-bin/ca
Protein Coding	43	GC17M041500	6.13	https://www.genecards.org/cgi-bin/ca
Protein Coding	42	GC05P069369	6.13	https://www.genecards.org/cgi-bin/ca
Protein Coding	41	GC11P095789	6.11	https://www.genecards.org/cgi-bin/ca
Protein Coding	44	GC02P073926	6.1	https://www.genecards.org/cgi-bin/ca
Protein Coding	37	GC04P087799	6.1	https://www.genecards.org/cgi-bin/ca
Protein Coding	45	GC04P006271	6.1	https://www.genecards.org/cgi-bin/ca
Protein Coding	42	GC13P099981	6.1	https://www.genecards.org/cgi-bin/ca
Protein Coding	41	GC02M219075	6.07	https://www.genecards.org/cgi-bin/ca
Protein Coding	36	GC17P082519	6.07	https://www.genecards.org/cgi-bin/ca
Protein Coding	32	GC19M042476	6.07	https://www.genecards.org/cgi-bin/ca
Protein Coding	41	GC12M049094	6.06	https://www.genecards.org/cgi-bin/ca
Protein Coding	45	GC17P068035	6.06	https://www.genecards.org/cgi-bin/ca
Protein Coding	44	GC14P067359	6.05	https://www.genecards.org/cgi-bin/ca
Protein Coding	42	GC08P074315	6.05	https://www.genecards.org/cgi-bin/ca
RNA Gene	13	GC03M045525	6.05	https://www.genecards.org/cgi-bin/ca
Protein Coding	42	GC10M070431	6.05	https://www.genecards.org/cgi-bin/ca
Protein Coding	45	GC17P047941	6.05	https://www.genecards.org/cgi-bin/ca
Protein Coding	37	GC01P196943	6.04	https://www.genecards.org/cgi-bin/ca
Protein Coding	37	GC09P083980	6.04	https://www.genecards.org/cgi-bin/ca
RNA Gene	21	GC20P034990	6.04	https://www.genecards.org/cgi-bin/ca
Protein Coding	36	GC17M019334	6.03	https://www.genecards.org/cgi-bin/ca
Protein Coding	38	GC07P145133	6.02	https://www.genecards.org/cgi-bin/ca
Protein Coding	48	GC16M064882	6.01	https://www.genecards.org/cgi-bin/ca
Protein Coding	43	GC07P120273	6.01	https://www.genecards.org/cgi-bin/ca
Protein Coding	40	GC19M041354	5.99	https://www.genecards.org/cgi-bin/ca
Protein Coding	49	GC03M015784	5.98	https://www.genecards.org/cgi-bin/ca
Protein Coding	41	GC22P024011	5.97	https://www.genecards.org/cgi-bin/ca
Protein Coding	42	GC06P047332	5.97	https://www.genecards.org/cgi-bin/ca
Protein Coding	44	GC02M199269	5.97	https://www.genecards.org/cgi-bin/ca
Protein Coding	40	GC06M030896	5.95	https://www.genecards.org/cgi-bin/ca
Protein Coding	39	GC0XM108720	5.95	https://www.genecards.org/cgi-bin/ca
Protein Coding	37	GC11M002928	5.94	https://www.genecards.org/cgi-bin/ca
Protein Coding	42	GC22P040951	5.94	https://www.genecards.org/cgi-bin/ca
Protein Coding	49	GC01P164524	5.93	https://www.genecards.org/cgi-bin/ca
Protein Coding	33	GC0YP002841	5.93	https://www.genecards.org/cgi-bin/ca
Protein Coding	41	GC16M056467	5.93	https://www.genecards.org/cgi-bin/ca
Protein Coding	44	GC05P150997	5.92	https://www.genecards.org/cgi-bin/ca
Protein Coding	47	GC03P009751	5.92	https://www.genecards.org/cgi-bin/ca
RNA Gene	16	GC13M050049	5.91	https://www.genecards.org/cgi-bin/ca
Protein Coding	50	GC11M102942	5.91	https://www.genecards.org/cgi-bin/ca
RNA Gene	12	GC20M026161	5.91	https://www.genecards.org/cgi-bin/ca
Protein Coding	45	GC14P090396	5.91	https://www.genecards.org/cgi-bin/ca
Protein Coding	43	GC15M074919	5.9	https://www.genecards.org/cgi-bin/ca
Protein Coding	48	GC19M010486	5.9	https://www.genecards.org/cgi-bin/ca
Protein Coding	41	GC06M135283	5.89	https://www.genecards.org/cgi-bin/ca

Protein Coding	40	GC04P125315	5.87	https://www.genecards.org/cgi-bin/ca
Protein Coding	47	GC11M036467	5.87	https://www.genecards.org/cgi-bin/ca
Protein Coding	47	GC17P073165	5.86	https://www.genecards.org/cgi-bin/ca
Protein Coding	48	GC17P056834	5.86	https://www.genecards.org/cgi-bin/ca
Protein Coding	44	GC02M237324	5.86	https://www.genecards.org/cgi-bin/ca
Protein Coding	45	GC02P147844	5.85	https://www.genecards.org/cgi-bin/ca
Protein Coding	37	GC08P144354	5.84	https://www.genecards.org/cgi-bin/ca
Protein Coding	48	GC03M033013	5.83	https://www.genecards.org/cgi-bin/ca
Protein Coding	41	GC14M049570	5.83	https://www.genecards.org/cgi-bin/ca
Protein Coding	44	GC11P010304	5.82	https://www.genecards.org/cgi-bin/ca
Protein Coding	44	GC06M031857	5.82	https://www.genecards.org/cgi-bin/ca
Protein Coding	43	GC20P063696	5.81	https://www.genecards.org/cgi-bin/ca
Protein Coding	42	GC19P012996	5.81	https://www.genecards.org/cgi-bin/ca
Protein Coding	40	GC19P048872	5.8	https://www.genecards.org/cgi-bin/ca
Protein Coding	47	GC11P070203	5.79	https://www.genecards.org/cgi-bin/ca
Protein Coding	48	GC07P016916	5.78	https://www.genecards.org/cgi-bin/ca
Protein Coding	45	GC02M174747	5.78	https://www.genecards.org/cgi-bin/ca
Protein Coding	46	GC12P120843	5.77	https://www.genecards.org/cgi-bin/ca
Protein Coding	49	GC06M032840	5.76	https://www.genecards.org/cgi-bin/ca
Protein Coding	43	GC13M103043	5.76	https://www.genecards.org/cgi-bin/ca
Protein Coding	37	GC19M051611	5.75	https://www.genecards.org/cgi-bin/ca
Protein Coding	44	GC19M018058	5.75	https://www.genecards.org/cgi-bin/ca
Protein Coding	41	GC04M150264	5.74	https://www.genecards.org/cgi-bin/ca
Protein Coding	44	GC17M074862	5.74	https://www.genecards.org/cgi-bin/ca
Protein Coding	41	GC14M020446	5.72	https://www.genecards.org/cgi-bin/ca
Protein Coding	45	GC17P040287	5.72	https://www.genecards.org/cgi-bin/ca
Protein Coding	41	GC02M027309	5.72	https://www.genecards.org/cgi-bin/ca
Protein Coding	40	GC06P158168	5.72	https://www.genecards.org/cgi-bin/ca
Protein Coding	52	GC04P186059	5.71	https://www.genecards.org/cgi-bin/ca
Protein Coding	41	GC08P144502	5.71	https://www.genecards.org/cgi-bin/ca
Protein Coding	43	GC02M226967	5.71	https://www.genecards.org/cgi-bin/ca
Protein Coding	45	GC0XP024054	5.71	https://www.genecards.org/cgi-bin/ca
Protein Coding	45	GC17P019648	5.71	https://www.genecards.org/cgi-bin/ca
Protein Coding	50	GC16P001153	5.7	https://www.genecards.org/cgi-bin/ca
Protein Coding	43	GC0XM053176	5.7	https://www.genecards.org/cgi-bin/ca
Protein Coding	45	GC11P047237	5.7	https://www.genecards.org/cgi-bin/ca
Protein Coding	47	GC01M017020	5.7	https://www.genecards.org/cgi-bin/ca
Protein Coding	49	GC0XM130129	5.69	https://www.genecards.org/cgi-bin/ca
Protein Coding	45	GC05M033986	5.68	https://www.genecards.org/cgi-bin/ca
RNA Gene	19	GC13P091434	5.68	https://www.genecards.org/cgi-bin/ca
Protein Coding	43	GC02P232456	5.68	https://www.genecards.org/cgi-bin/ca
Protein Coding	39	GC04P078056	5.68	https://www.genecards.org/cgi-bin/ca
Protein Coding	41	GC05P148025	5.68	https://www.genecards.org/cgi-bin/ca
Protein Coding	36	GC07P139344	5.67	https://www.genecards.org/cgi-bin/ca
Protein Coding	44	GC11M103907	5.67	https://www.genecards.org/cgi-bin/ca
Protein Coding	39	GC09P005846	5.66	https://www.genecards.org/cgi-bin/ca
Protein Coding	46	GC16P056961	5.64	https://www.genecards.org/cgi-bin/ca
Protein Coding	51	GC19M017824	5.63	https://www.genecards.org/cgi-bin/ca
Protein Coding	49	GC11P072223	5.63	https://www.genecards.org/cgi-bin/ca
Protein Coding	41	GC15P072686	5.63	https://www.genecards.org/cgi-bin/ca
Protein Coding	44	GC05M037288	5.63	https://www.genecards.org/cgi-bin/ca
Protein Coding	37	GC04P087608	5.61	https://www.genecards.org/cgi-bin/ca
Protein Coding	49	GC06P003064	5.61	https://www.genecards.org/cgi-bin/ca
Protein Coding	45	GC05M078000	5.6	https://www.genecards.org/cgi-bin/ca

Protein Coding	41	GC11M005693	5.6	https://www.genecards.org/cgi-bin/ca
Protein Coding	40	GC08P006406	5.59	https://www.genecards.org/cgi-bin/ca
Protein Coding	43	GC17P040443	5.59	https://www.genecards.org/cgi-bin/ca
Protein Coding	42	GC03M136336	5.58	https://www.genecards.org/cgi-bin/ca
Protein Coding	37	GC01M204400	5.57	https://www.genecards.org/cgi-bin/ca
Protein Coding	47	GC16M088810	5.57	https://www.genecards.org/cgi-bin/ca
Protein Coding	43	GC17P008018	5.57	https://www.genecards.org/cgi-bin/ca
Protein Coding	39	GC07P033112	5.57	https://www.genecards.org/cgi-bin/ca
Protein Coding	43	GC20M051784	5.57	https://www.genecards.org/cgi-bin/ca
Protein Coding	26	GC14M109515	5.55	https://www.genecards.org/cgi-bin/ca
Protein Coding	47	GC12P006913	5.55	https://www.genecards.org/cgi-bin/ca
Protein Coding	41	GC02P240586	5.54	https://www.genecards.org/cgi-bin/ca
Protein Coding	50	GC07M151556	5.53	https://www.genecards.org/cgi-bin/ca
Protein Coding	40	GC10M095237	5.53	https://www.genecards.org/cgi-bin/ca
Protein Coding	51	GC02M157736	5.52	https://www.genecards.org/cgi-bin/ca
Genetic Locus	2	GC03U990254	5.52	https://www.genecards.org/cgi-bin/ca
Protein Coding	40	GC11M124919	5.51	https://www.genecards.org/cgi-bin/ca
Protein Coding	51	GC21M025880	5.51	https://www.genecards.org/cgi-bin/ca
Protein Coding	43	GC16M081081	5.51	https://www.genecards.org/cgi-bin/ca
Protein Coding	44	GC15P039934	5.5	https://www.genecards.org/cgi-bin/ca
Protein Coding	43	GC02M135787	5.5	https://www.genecards.org/cgi-bin/ca
Protein Coding	44	GC14P064388	5.48	https://www.genecards.org/cgi-bin/ca
Protein Coding	39	GC04P147732	5.47	https://www.genecards.org/cgi-bin/ca
Protein Coding	46	GC22P020917	5.47	https://www.genecards.org/cgi-bin/ca
Protein Coding	53	GC01P009629	5.45	https://www.genecards.org/cgi-bin/ca
Protein Coding	42	GC0XM025021	5.44	https://www.genecards.org/cgi-bin/ca
Protein Coding	43	GC11P116829	5.42	https://www.genecards.org/cgi-bin/ca
Protein Coding	46	GC12P052022	5.42	https://www.genecards.org/cgi-bin/ca
Protein Coding	49	GC17M042199	5.41	https://www.genecards.org/cgi-bin/ca
Protein Coding	44	GC17P034980	5.41	https://www.genecards.org/cgi-bin/ca
Protein Coding	44	GC06M112107	5.4	https://www.genecards.org/cgi-bin/ca
Protein Coding	36	GC05P033441	5.4	https://www.genecards.org/cgi-bin/ca
Protein Coding	48	GC02M038034	5.39	https://www.genecards.org/cgi-bin/ca
Protein Coding	44	GC01M154582	5.39	https://www.genecards.org/cgi-bin/ca
Protein Coding	45	GC17P007219	5.39	https://www.genecards.org/cgi-bin/ca
Protein Coding	38	GC17M063894	5.39	https://www.genecards.org/cgi-bin/ca
Protein Coding	47	GC0XP038353	5.37	https://www.genecards.org/cgi-bin/ca
Protein Coding	49	GC01M182350	5.36	https://www.genecards.org/cgi-bin/ca
Protein Coding	49	GC01P226870	5.36	https://www.genecards.org/cgi-bin/ca
Protein Coding	48	GC19M011377	5.36	https://www.genecards.org/cgi-bin/ca
Protein Coding	41	GC03P048449	5.35	https://www.genecards.org/cgi-bin/ca
Protein Coding	43	GC10P022326	5.35	https://www.genecards.org/cgi-bin/ca
Protein Coding	42	GC0XM153929	5.35	https://www.genecards.org/cgi-bin/ca
Protein Coding	35	GC11M000626	5.35	https://www.genecards.org/cgi-bin/ca
Protein Coding	30	GCMTP009209	5.35	https://www.genecards.org/cgi-bin/ca
Protein Coding	43	GC03M098576	5.34	https://www.genecards.org/cgi-bin/ca
Protein Coding	44	GC03M049121	5.34	https://www.genecards.org/cgi-bin/ca
Protein Coding	50	GC06P135180	5.34	https://www.genecards.org/cgi-bin/ca
Protein Coding	43	GC22M030240	5.33	https://www.genecards.org/cgi-bin/ca
Protein Coding	43	GC0XM101345	5.33	https://www.genecards.org/cgi-bin/ca
Protein Coding	44	GC17M066212	5.32	https://www.genecards.org/cgi-bin/ca
Protein Coding	48	GC22M037125	5.31	https://www.genecards.org/cgi-bin/ca
Protein Coding	43	GC12P106357	5.31	https://www.genecards.org/cgi-bin/ca
Protein Coding	40	GC06M042222	5.3	https://www.genecards.org/cgi-bin/ca

Protein Coding	36	GC09M133152	5.3	https://www.genecards.org/cgi-bin/ca
Protein Coding	41	GC03P157436	5.29	https://www.genecards.org/cgi-bin/ca
Protein Coding	40	GC11P110429	5.29	https://www.genecards.org/cgi-bin/ca
Protein Coding	39	GC02M086144	5.29	https://www.genecards.org/cgi-bin/ca
Protein Coding	43	GC04M071741	5.29	https://www.genecards.org/cgi-bin/ca
Protein Coding	37	GC09P095871	5.29	https://www.genecards.org/cgi-bin/ca
Protein Coding	39	GC01P054542	5.28	https://www.genecards.org/cgi-bin/ca
Protein Coding	37	GC12P110614	5.28	https://www.genecards.org/cgi-bin/ca
Protein Coding	46	GC09P133061	5.28	https://www.genecards.org/cgi-bin/ca
Protein Coding	43	GC03M046435	5.28	https://www.genecards.org/cgi-bin/ca
Protein Coding	46	GC02P186589	5.27	https://www.genecards.org/cgi-bin/ca
Protein Coding	41	GC02M162142	5.27	https://www.genecards.org/cgi-bin/ca
Protein Coding	39	GC22M019037	5.27	https://www.genecards.org/cgi-bin/ca
Protein Coding	47	GC03M128479	5.27	https://www.genecards.org/cgi-bin/ca
Protein Coding	43	GC07M120787	5.26	https://www.genecards.org/cgi-bin/ca
Protein Coding	45	GC22M028794	5.26	https://www.genecards.org/cgi-bin/ca
Protein Coding	43	GC21P045981	5.26	https://www.genecards.org/cgi-bin/ca
Protein Coding	43	GC21P034111	5.26	https://www.genecards.org/cgi-bin/ca
Protein Coding	41	GC01P009234	5.24	https://www.genecards.org/cgi-bin/ca
Protein Coding	41	GC20M005303	5.24	https://www.genecards.org/cgi-bin/ca
Protein Coding	40	GC20M034088	5.24	https://www.genecards.org/cgi-bin/ca
Protein Coding	43	GC07P107660	5.24	https://www.genecards.org/cgi-bin/ca
Protein Coding	44	GC09M129812	5.23	https://www.genecards.org/cgi-bin/ca
RNA Gene	20	GC09P124692	5.23	https://www.genecards.org/cgi-bin/ca
Protein Coding	37	GC19M038770	5.23	https://www.genecards.org/cgi-bin/ca
Protein Coding	47	GC04M108047	5.22	https://www.genecards.org/cgi-bin/ca
Protein Coding	44	GC01P161197	5.22	https://www.genecards.org/cgi-bin/ca
Protein Coding	43	GC03M179397	5.21	https://www.genecards.org/cgi-bin/ca
Protein Coding	36	GC17M039211	5.21	https://www.genecards.org/cgi-bin/ca
Protein Coding	45	GC12M124776	5.21	https://www.genecards.org/cgi-bin/ca
Protein Coding	49	GC06P030720	5.21	https://www.genecards.org/cgi-bin/ca
Protein Coding	40	GC01P230642	5.2	https://www.genecards.org/cgi-bin/ca
Protein Coding	35	GC04P118850	5.2	https://www.genecards.org/cgi-bin/ca
Protein Coding	45	GC13P108251	5.19	https://www.genecards.org/cgi-bin/ca
Protein Coding	45	GC08P022042	5.19	https://www.genecards.org/cgi-bin/ca
Protein Coding	44	GC12M094898	5.18	https://www.genecards.org/cgi-bin/ca
Protein Coding	44	GC17P001761	5.18	https://www.genecards.org/cgi-bin/ca
Protein Coding	45	GC14M075079	5.18	https://www.genecards.org/cgi-bin/ca
Protein Coding	41	GC05M132689	5.18	https://www.genecards.org/cgi-bin/ca
Protein Coding	50	GC02M161992	5.17	https://www.genecards.org/cgi-bin/ca
Protein Coding	36	GC0XM154477	5.17	https://www.genecards.org/cgi-bin/ca
Biological Region	1	GC05P132629	5.17	https://www.genecards.org/cgi-bin/ca
Protein Coding	43	GC14P038207	5.17	https://www.genecards.org/cgi-bin/ca
Protein Coding	43	GC0XP108439	5.17	https://www.genecards.org/cgi-bin/ca
Protein Coding	45	GC17M041523	5.16	https://www.genecards.org/cgi-bin/ca
Protein Coding	43	GC12P004649	5.16	https://www.genecards.org/cgi-bin/ca
Protein Coding	50	GC11M105025	5.16	https://www.genecards.org/cgi-bin/ca
Protein Coding	44	GC10M128096	5.15	https://www.genecards.org/cgi-bin/ca
Protein Coding	34	GC02M224378	5.15	https://www.genecards.org/cgi-bin/ca
Protein Coding	39	GC17P075205	5.15	https://www.genecards.org/cgi-bin/ca
Protein Coding	45	GC22M037204	5.14	https://www.genecards.org/cgi-bin/ca
Protein Coding	52	GC01P032251	5.14	https://www.genecards.org/cgi-bin/ca
Protein Coding	40	GC11P066029	5.14	https://www.genecards.org/cgi-bin/ca
Protein Coding	45	GC17M041619	5.13	https://www.genecards.org/cgi-bin/ca

Protein Coding	38	GC19P003880	5.13	https://www.genecards.org/cgi-bin/ca
Protein Coding	44	GC01M011674	5.13	https://www.genecards.org/cgi-bin/ca
Protein Coding	46	GC19P041877	5.13	https://www.genecards.org/cgi-bin/ca
Protein Coding	43	GC01P119507	5.11	https://www.genecards.org/cgi-bin/ca
Protein Coding	43	GC17P034285	5.11	https://www.genecards.org/cgi-bin/ca
Protein Coding	39	GC02P201387	5.1	https://www.genecards.org/cgi-bin/ca
RNA Gene	19	GC14P104655	5.1	https://www.genecards.org/cgi-bin/ca
Protein Coding	47	GC19M042401	5.1	https://www.genecards.org/cgi-bin/ca
Protein Coding	43	GC14M094341	5.09	https://www.genecards.org/cgi-bin/ca
Protein Coding	36	GC09P017124	5.08	https://www.genecards.org/cgi-bin/ca
Protein Coding	47	GC12P057460	5.08	https://www.genecards.org/cgi-bin/ca
Protein Coding	44	GC05P006633	5.07	https://www.genecards.org/cgi-bin/ca
Protein Coding	50	GC09P133636	5.07	https://www.genecards.org/cgi-bin/ca
Protein Coding	50	GC17P045894	5.06	https://www.genecards.org/cgi-bin/ca
Protein Coding	45	GC01M023795	5.06	https://www.genecards.org/cgi-bin/ca
Protein Coding	40	GC14P032203	5.05	https://www.genecards.org/cgi-bin/ca
Protein Coding	41	GC16P001072	5.05	https://www.genecards.org/cgi-bin/ca
Protein Coding	44	GC01M093992	5.05	https://www.genecards.org/cgi-bin/ca
Protein Coding	44	GC17P003472	5.05	https://www.genecards.org/cgi-bin/ca
Protein Coding	44	GC13P033016	5.04	https://www.genecards.org/cgi-bin/ca
Protein Coding	32	GC17M063872	5.04	https://www.genecards.org/cgi-bin/ca
Protein Coding	43	GC17M075827	5.03	https://www.genecards.org/cgi-bin/ca
Protein Coding	45	GC07M100889	5.03	https://www.genecards.org/cgi-bin/ca
Protein Coding	47	GC13M046831	5.02	https://www.genecards.org/cgi-bin/ca
Protein Coding	45	GC03P184314	5.02	https://www.genecards.org/cgi-bin/ca
Protein Coding	45	GC05M087311	5.01	https://www.genecards.org/cgi-bin/ca
Protein Coding	43	GC08M056067	5	https://www.genecards.org/cgi-bin/ca
Protein Coding	32	GC17M042404	5	https://www.genecards.org/cgi-bin/ca
Protein Coding	39	GC14M103556	4.97	https://www.genecards.org/cgi-bin/ca
Protein Coding	44	GC11P068684	4.97	https://www.genecards.org/cgi-bin/ca
Protein Coding	44	GC07P143316	4.97	https://www.genecards.org/cgi-bin/ca
Protein Coding	45	GC17P050634	4.97	https://www.genecards.org/cgi-bin/ca
Protein Coding	43	GC21P046098	4.96	https://www.genecards.org/cgi-bin/ca
Protein Coding	32	GC19P038790	4.96	https://www.genecards.org/cgi-bin/ca
Protein Coding	45	GC04M039502	4.96	https://www.genecards.org/cgi-bin/ca
Protein Coding	39	GC02P223957	4.96	https://www.genecards.org/cgi-bin/ca
Protein Coding	44	GC02P038665	4.94	https://www.genecards.org/cgi-bin/ca
Protein Coding	43	GC02P063840	4.94	https://www.genecards.org/cgi-bin/ca
Protein Coding	43	GC01P162561	4.94	https://www.genecards.org/cgi-bin/ca
Protein Coding	41	GC15P049155	4.94	https://www.genecards.org/cgi-bin/ca
Protein Coding	39	GC19M042451	4.94	https://www.genecards.org/cgi-bin/ca
Protein Coding	33	GC20M000603	4.94	https://www.genecards.org/cgi-bin/ca
Protein Coding	48	GC03M049958	4.94	https://www.genecards.org/cgi-bin/ca
Protein Coding	45	GC16P030472	4.93	https://www.genecards.org/cgi-bin/ca
Protein Coding	36	GC17M029952	4.93	https://www.genecards.org/cgi-bin/ca
Protein Coding	41	GC17M008849	4.92	https://www.genecards.org/cgi-bin/ca
Protein Coding	45	GC19M003777	4.92	https://www.genecards.org/cgi-bin/ca
Protein Coding	45	GC03M015787	4.91	https://www.genecards.org/cgi-bin/ca
Protein Coding	46	GC01P236795	4.91	https://www.genecards.org/cgi-bin/ca
Protein Coding	43	GC17M008851	4.91	https://www.genecards.org/cgi-bin/ca
Protein Coding	45	GC02P028752	4.9	https://www.genecards.org/cgi-bin/ca
Protein Coding	44	GC01P091500	4.89	https://www.genecards.org/cgi-bin/ca
Protein Coding	32	GC17P018807	4.88	https://www.genecards.org/cgi-bin/ca
Protein Coding	28	GC10P079512	4.88	https://www.genecards.org/cgi-bin/ca

Protein Coding	40	GC11P062405	4.88	https://www.genecards.org/cgi-bin/ca
Protein Coding	43	GC17P042548	4.88	https://www.genecards.org/cgi-bin/ca
Protein Coding	41	GC02P057907	4.88	https://www.genecards.org/cgi-bin/ca
Protein Coding	48	GC01M027622	4.88	https://www.genecards.org/cgi-bin/ca
Protein Coding	44	GC14M023016	4.88	https://www.genecards.org/cgi-bin/ca
Protein Coding	42	GC14P092923	4.87	https://www.genecards.org/cgi-bin/ca
Protein Coding	44	GC05M159314	4.87	https://www.genecards.org/cgi-bin/ca
Protein Coding	41	GC19M017816	4.87	https://www.genecards.org/cgi-bin/ca
Protein Coding	50	GC22M045657	4.87	https://www.genecards.org/cgi-bin/ca
Protein Coding	45	GC20P045890	4.87	https://www.genecards.org/cgi-bin/ca
Protein Coding	39	GC11M074012	4.87	https://www.genecards.org/cgi-bin/ca
Protein Coding	48	GC03M151336	4.86	https://www.genecards.org/cgi-bin/ca
Protein Coding	45	GC01P002228	4.85	https://www.genecards.org/cgi-bin/ca
Protein Coding	43	GC01P025574	4.84	https://www.genecards.org/cgi-bin/ca
Protein Coding	47	GC09M006522	4.84	https://www.genecards.org/cgi-bin/ca
Protein Coding	47	GC11M123057	4.84	https://www.genecards.org/cgi-bin/ca
Protein Coding	42	GC07M094585	4.84	https://www.genecards.org/cgi-bin/ca
Protein Coding	45	GC19P050385	4.83	https://www.genecards.org/cgi-bin/ca
Protein Coding	46	GC07M142871	4.83	https://www.genecards.org/cgi-bin/ca
Protein Coding	48	GC08M030678	4.82	https://www.genecards.org/cgi-bin/ca
Protein Coding	43	GC10M104031	4.82	https://www.genecards.org/cgi-bin/ca
Protein Coding	48	GC21M043053	4.82	https://www.genecards.org/cgi-bin/ca
Protein Coding	43	GC10M062811	4.8	https://www.genecards.org/cgi-bin/ca
Protein Coding	45	GC08P022164	4.8	https://www.genecards.org/cgi-bin/ca
Protein Coding	41	GC12M108784	4.79	https://www.genecards.org/cgi-bin/ca
Protein Coding	47	GC17M007189	4.79	https://www.genecards.org/cgi-bin/ca
Protein Coding	47	GC20M035433	4.79	https://www.genecards.org/cgi-bin/ca
Protein Coding	46	GC03P046383	4.79	https://www.genecards.org/cgi-bin/ca
Protein Coding	46	GC20M001369	4.78	https://www.genecards.org/cgi-bin/ca
Protein Coding	41	GC17P012665	4.78	https://www.genecards.org/cgi-bin/ca
Protein Coding	47	GC16P068637	4.78	https://www.genecards.org/cgi-bin/ca
Protein Coding	45	GC07M026174	4.78	https://www.genecards.org/cgi-bin/ca
Protein Coding	46	GC09P069035	4.77	https://www.genecards.org/cgi-bin/ca
Protein Coding	37	GC0XM129645	4.77	https://www.genecards.org/cgi-bin/ca
Protein Coding	45	GC03M146069	4.77	https://www.genecards.org/cgi-bin/ca
Protein Coding	42	GC14M094768	4.76	https://www.genecards.org/cgi-bin/ca
Protein Coding	43	GC0XP116170	4.76	https://www.genecards.org/cgi-bin/ca
Protein Coding	40	GC03P170418	4.76	https://www.genecards.org/cgi-bin/ca
Protein Coding	36	GC11P094493	4.75	https://www.genecards.org/cgi-bin/ca
Protein Coding	43	GC17M004270	4.75	https://www.genecards.org/cgi-bin/ca
Protein Coding	37	GC10M116671	4.75	https://www.genecards.org/cgi-bin/ca
Protein Coding	47	GC11P089177	4.75	https://www.genecards.org/cgi-bin/ca
Protein Coding	40	GC19P018831	4.75	https://www.genecards.org/cgi-bin/ca
Protein Coding	45	GC18M060371	4.74	https://www.genecards.org/cgi-bin/ca
Protein Coding	31	GC08P089901	4.74	https://www.genecards.org/cgi-bin/ca
Protein Coding	35	GC06M029504	4.73	https://www.genecards.org/cgi-bin/ca
Protein Coding	41	GC04M083261	4.73	https://www.genecards.org/cgi-bin/ca
Protein Coding	42	GC16P082626	4.73	https://www.genecards.org/cgi-bin/ca
Protein Coding	40	GC17P008027	4.73	https://www.genecards.org/cgi-bin/ca
Protein Coding	40	GC06M017615	4.73	https://www.genecards.org/cgi-bin/ca
Protein Coding	42	GC17M063832	4.73	https://www.genecards.org/cgi-bin/ca
Protein Coding	45	GC11P112961	4.72	https://www.genecards.org/cgi-bin/ca
Protein Coding	32	GC22Mi00270	4.71	https://www.genecards.org/cgi-bin/ca
Protein Coding	47	GC08P042338	4.71	https://www.genecards.org/cgi-bin/ca

Protein Coding	35	GC0XP101098	4.71	https://www.genecards.org/cgi-bin/ca
Protein Coding	45	GC02M191029	4.7	https://www.genecards.org/cgi-bin/ca
Protein Coding	45	GC11P102347	4.69	https://www.genecards.org/cgi-bin/ca
Biological Region	1	GC17P030235	4.68	https://www.genecards.org/cgi-bin/ca
Protein Coding	34	GC02M177548	4.66	https://www.genecards.org/cgi-bin/ca
Protein Coding	44	GC15P085381	4.65	https://www.genecards.org/cgi-bin/ca
Protein Coding	36	GC13M023328	4.65	https://www.genecards.org/cgi-bin/ca
Protein Coding	41	GC14P103333	4.64	https://www.genecards.org/cgi-bin/ca
Protein Coding	39	GC16M000046	4.64	https://www.genecards.org/cgi-bin/ca
Protein Coding	36	GC07M073536	4.64	https://www.genecards.org/cgi-bin/ca
Protein Coding	42	GC06P047340	4.63	https://www.genecards.org/cgi-bin/ca
Protein Coding	35	GC04P122702	4.63	https://www.genecards.org/cgi-bin/ca
Protein Coding	43	GC02P216412	4.63	https://www.genecards.org/cgi-bin/ca
Protein Coding	41	GC03P048266	4.63	https://www.genecards.org/cgi-bin/ca
RNA Gene	14	GCMTP000580	4.63	https://www.genecards.org/cgi-bin/ca
RNA Gene	21	GC12P054200	4.62	https://www.genecards.org/cgi-bin/ca
Protein Coding	41	GC01M006265	4.62	https://www.genecards.org/cgi-bin/ca
Protein Coding	37	GC17P041688	4.62	https://www.genecards.org/cgi-bin/ca
Protein Coding	35	GC22M019450	4.61	https://www.genecards.org/cgi-bin/ca
Protein Coding	42	GC14M091965	4.61	https://www.genecards.org/cgi-bin/ca
Protein Coding	43	GC02M020200	4.61	https://www.genecards.org/cgi-bin/ca
Protein Coding	39	GC19P055554	4.61	https://www.genecards.org/cgi-bin/ca
Protein Coding	41	GC11P065525	4.61	https://www.genecards.org/cgi-bin/ca
Protein Coding	42	GC07M149003	4.6	https://www.genecards.org/cgi-bin/ca
Protein Coding	48	GC19M043767	4.6	https://www.genecards.org/cgi-bin/ca
Protein Coding	47	GC01P228127	4.6	https://www.genecards.org/cgi-bin/ca
Protein Coding	48	GC0XP077928	4.59	https://www.genecards.org/cgi-bin/ca
Protein Coding	40	GC17P055750	4.59	https://www.genecards.org/cgi-bin/ca
Protein Coding	38	GC05M081309	4.59	https://www.genecards.org/cgi-bin/ca
Protein Coding	37	GC17P078168	4.59	https://www.genecards.org/cgi-bin/ca
Protein Coding	35	GC10M084231	4.59	https://www.genecards.org/cgi-bin/ca
Protein Coding	32	GC03M161344	4.59	https://www.genecards.org/cgi-bin/ca
Protein Coding	27	GC17P041255	4.59	https://www.genecards.org/cgi-bin/ca
Protein Coding	39	GC02P134918	4.59	https://www.genecards.org/cgi-bin/ca
Protein Coding	37	GC16M001771	4.58	https://www.genecards.org/cgi-bin/ca
Protein Coding	24	GC07M075029	4.58	https://www.genecards.org/cgi-bin/ca
Protein Coding	34	GC11P104036	4.58	https://www.genecards.org/cgi-bin/ca
Protein Coding	48	GC03M170996	4.56	https://www.genecards.org/cgi-bin/ca
Protein Coding	48	GC15M034229	4.55	https://www.genecards.org/cgi-bin/ca
Protein Coding	43	GC01M161039	4.55	https://www.genecards.org/cgi-bin/ca
Protein Coding	43	GC01P115641	4.55	https://www.genecards.org/cgi-bin/ca
Protein Coding	47	GC12P098645	4.55	https://www.genecards.org/cgi-bin/ca
Protein Coding	40	GC03P119294	4.54	https://www.genecards.org/cgi-bin/ca
Protein Coding	40	GC09P006206	4.54	https://www.genecards.org/cgi-bin/ca
Protein Coding	47	GC20M054153	4.53	https://www.genecards.org/cgi-bin/ca
Biological Region	1	GC15P074370	4.53	https://www.genecards.org/cgi-bin/ca
Protein Coding	51	GC08P085463	4.53	https://www.genecards.org/cgi-bin/ca
Protein Coding	37	GC21P044012	4.52	https://www.genecards.org/cgi-bin/ca
RNA Gene	17	GC11M001997	4.52	https://www.genecards.org/cgi-bin/ca
Protein Coding	39	GC06P031399	4.52	https://www.genecards.org/cgi-bin/ca
Protein Coding	44	GC11P112087	4.52	https://www.genecards.org/cgi-bin/ca
Protein Coding	39	GC17P057085	4.52	https://www.genecards.org/cgi-bin/ca
Protein Coding	47	GC11P002377	4.52	https://www.genecards.org/cgi-bin/ca
Protein Coding	39	GC01M019074	4.51	https://www.genecards.org/cgi-bin/ca

Protein Coding	42	GC17M018271	4.51	https://www.genecards.org/cgi-bin/ca
Protein Coding	47	GC05M063960	4.5	https://www.genecards.org/cgi-bin/ca
RNA Gene	21	GC17M048579	4.5	https://www.genecards.org/cgi-bin/ca
Protein Coding	43	GC19M054923	4.5	https://www.genecards.org/cgi-bin/ca
Protein Coding	50	GC10M087050	4.5	https://www.genecards.org/cgi-bin/ca
Protein Coding	43	GC07M078017	4.49	https://www.genecards.org/cgi-bin/ca
Protein Coding	34	GC0XM015345	4.49	https://www.genecards.org/cgi-bin/ca
Protein Coding	45	GC01P003068	4.48	https://www.genecards.org/cgi-bin/ca
Protein Coding	47	GC03M172443	4.48	https://www.genecards.org/cgi-bin/ca
Protein Coding	42	GC01M161184	4.48	https://www.genecards.org/cgi-bin/ca
Protein Coding	37	GC0XM064185	4.47	https://www.genecards.org/cgi-bin/ca
Protein Coding	39	GC14P058633	4.47	https://www.genecards.org/cgi-bin/ca
Protein Coding	41	GC11M061563	4.46	https://www.genecards.org/cgi-bin/ca
Protein Coding	46	GC05P087267	4.46	https://www.genecards.org/cgi-bin/ca
Protein Coding	45	GC03P119780	4.45	https://www.genecards.org/cgi-bin/ca
Protein Coding	37	GC11P059142	4.45	https://www.genecards.org/cgi-bin/ca
Protein Coding	45	GC20M051386	4.45	https://www.genecards.org/cgi-bin/ca
Protein Coding	44	GC0XP123960	4.45	https://www.genecards.org/cgi-bin/ca
Protein Coding	44	GC01P161314	4.45	https://www.genecards.org/cgi-bin/ca
Protein Coding	36	GC01M003812	4.45	https://www.genecards.org/cgi-bin/ca
RNA Gene	18	GC17P030117	4.44	https://www.genecards.org/cgi-bin/ca
Protein Coding	40	GC11M061283	4.44	https://www.genecards.org/cgi-bin/ca
Protein Coding	43	GC22M035606	4.43	https://www.genecards.org/cgi-bin/ca
Protein Coding	40	GC02P032063	4.43	https://www.genecards.org/cgi-bin/ca
Protein Coding	45	GC03M008767	4.42	https://www.genecards.org/cgi-bin/ca
RNA Gene	21	GC05P095838	4.42	https://www.genecards.org/cgi-bin/ca
RNA Gene	13	GCMTP012140	4.42	https://www.genecards.org/cgi-bin/ca
Protein Coding	43	GC17P016380	4.41	https://www.genecards.org/cgi-bin/ca
Protein Coding	41	GC09M027539	4.41	https://www.genecards.org/cgi-bin/ca
Protein Coding	41	GC03M119524	4.41	https://www.genecards.org/cgi-bin/ca
Protein Coding	43	GC05P083077	4.41	https://www.genecards.org/cgi-bin/ca
Protein Coding	41	GC20P023035	4.4	https://www.genecards.org/cgi-bin/ca
Protein Coding	43	GC17P039626	4.39	https://www.genecards.org/cgi-bin/ca
Protein Coding	48	GC05P153467	4.39	https://www.genecards.org/cgi-bin/ca
Protein Coding	45	GC05P111223	4.39	https://www.genecards.org/cgi-bin/ca
Protein Coding	37	GC16P000981	4.38	https://www.genecards.org/cgi-bin/ca
Protein Coding	41	GC22M019171	4.38	https://www.genecards.org/cgi-bin/ca
Protein Coding	40	GC19M049869	4.38	https://www.genecards.org/cgi-bin/ca
Protein Coding	45	GC18M026852	4.37	https://www.genecards.org/cgi-bin/ca
Protein Coding	43	GC21M034513	4.35	https://www.genecards.org/cgi-bin/ca
Protein Coding	40	GC20M035278	4.35	https://www.genecards.org/cgi-bin/ca
Protein Coding	45	GC12M057516	4.35	https://www.genecards.org/cgi-bin/ca
Protein Coding	41	GC16M074672	4.35	https://www.genecards.org/cgi-bin/ca
Biological Regior	5	GC11P001999	4.34	https://www.genecards.org/cgi-bin/ca
Protein Coding	38	GC11P103109	4.34	https://www.genecards.org/cgi-bin/ca
Protein Coding	51	GC01M097015	4.34	https://www.genecards.org/cgi-bin/ca
Protein Coding	48	GC22M042126	4.33	https://www.genecards.org/cgi-bin/ca
Protein Coding	41	GC09M035726	4.33	https://www.genecards.org/cgi-bin/ca
Protein Coding	47	GC11M017191	4.31	https://www.genecards.org/cgi-bin/ca
Protein Coding	46	GC11P067428	4.31	https://www.genecards.org/cgi-bin/ca
Protein Coding	48	GC21M034787	4.29	https://www.genecards.org/cgi-bin/ca
Protein Coding	46	GC12P109097	4.29	https://www.genecards.org/cgi-bin/ca
Protein Coding	36	GC16P019536	4.29	https://www.genecards.org/cgi-bin/ca
Protein Coding	34	GC03P101723	4.29	https://www.genecards.org/cgi-bin/ca

Protein Coding	31	GC22M021207	4.29	https://www.genecards.org/cgi-bin/ca
Protein Coding	20	GC22M018516	4.29	https://www.genecards.org/cgi-bin/ca
Protein Coding	30	GC09P127716	4.29	https://www.genecards.org/cgi-bin/ca
Protein Coding	39	GC01P042682	4.29	https://www.genecards.org/cgi-bin/ca
Protein Coding	44	GC18P023689	4.28	https://www.genecards.org/cgi-bin/ca
Protein Coding	41	GC05M128257	4.28	https://www.genecards.org/cgi-bin/ca
Protein Coding	43	GC15P073925	4.28	https://www.genecards.org/cgi-bin/ca
Protein Coding	42	GC11M116820	4.27	https://www.genecards.org/cgi-bin/ca
Protein Coding	40	GC22P050674	4.27	https://www.genecards.org/cgi-bin/ca
Protein Coding	40	GC20M025294	4.26	https://www.genecards.org/cgi-bin/ca
Protein Coding	44	GC03P186717	4.26	https://www.genecards.org/cgi-bin/ca
Protein Coding	45	GC15P050182	4.26	https://www.genecards.org/cgi-bin/ca
Protein Coding	47	GC12M052897	4.25	https://www.genecards.org/cgi-bin/ca
Protein Coding	43	GC09P111896	4.25	https://www.genecards.org/cgi-bin/ca
Protein Coding	41	GC0XM053936	4.24	https://www.genecards.org/cgi-bin/ca
Protein Coding	48	GC07P045580	4.24	https://www.genecards.org/cgi-bin/ca
Protein Coding	27	GC21P039377	4.24	https://www.genecards.org/cgi-bin/ca
Protein Coding	35	GC03P037989	4.24	https://www.genecards.org/cgi-bin/ca
Protein Coding	36	GC16M004332	4.24	https://www.genecards.org/cgi-bin/ca
Protein Coding	44	GC16P031570	4.23	https://www.genecards.org/cgi-bin/ca
Protein Coding	43	GC22P037675	4.23	https://www.genecards.org/cgi-bin/ca
Protein Coding	45	GC03P019963	4.23	https://www.genecards.org/cgi-bin/ca
Protein Coding	45	GC10P052314	4.23	https://www.genecards.org/cgi-bin/ca
Protein Coding	43	GC06P000391	4.23	https://www.genecards.org/cgi-bin/ca
Protein Coding	46	GC05P069243	4.22	https://www.genecards.org/cgi-bin/ca
Protein Coding	43	GC21M036750	4.22	https://www.genecards.org/cgi-bin/ca
Protein Coding	41	GC17M076470	4.22	https://www.genecards.org/cgi-bin/ca
Protein Coding	40	GC02P135052	4.21	https://www.genecards.org/cgi-bin/ca
Protein Coding	48	GC02P181456	4.21	https://www.genecards.org/cgi-bin/ca
Protein Coding	41	GC0XM040049	4.21	https://www.genecards.org/cgi-bin/ca
Protein Coding	39	GC01P169367	4.21	https://www.genecards.org/cgi-bin/ca
Protein Coding	42	GC17P036937	4.21	https://www.genecards.org/cgi-bin/ca
Protein Coding	41	GC22M041576	4.21	https://www.genecards.org/cgi-bin/ca
Protein Coding	37	GC0XP104566	4.2	https://www.genecards.org/cgi-bin/ca
Protein Coding	41	GC04P144645	4.2	https://www.genecards.org/cgi-bin/ca
Protein Coding	33	GC07M019184	4.2	https://www.genecards.org/cgi-bin/ca
Protein Coding	50	GC21M044885	4.18	https://www.genecards.org/cgi-bin/ca
Protein Coding	49	GC09M077716	4.17	https://www.genecards.org/cgi-bin/ca
Protein Coding	42	GC15P081159	4.17	https://www.genecards.org/cgi-bin/ca
Protein Coding	48	GC03P004486	4.16	https://www.genecards.org/cgi-bin/ca
Protein Coding	45	GC06M032648	4.16	https://www.genecards.org/cgi-bin/ca
Protein Coding	43	GC06M116118	4.15	https://www.genecards.org/cgi-bin/ca
Protein Coding	43	GC04M041746	4.15	https://www.genecards.org/cgi-bin/ca
Protein Coding	51	GC04M085990	4.15	https://www.genecards.org/cgi-bin/ca
Protein Coding	43	GC14M024336	4.15	https://www.genecards.org/cgi-bin/ca
Protein Coding	43	GC01M050439	4.14	https://www.genecards.org/cgi-bin/ca
Protein Coding	45	GC01M054849	4.14	https://www.genecards.org/cgi-bin/ca
Protein Coding	41	GC04M073980	4.14	https://www.genecards.org/cgi-bin/ca
Protein Coding	50	GC21P037365	4.14	https://www.genecards.org/cgi-bin/ca
Protein Coding	44	GC16P031180	4.14	https://www.genecards.org/cgi-bin/ca
Protein Coding	45	GC17M063683	4.14	https://www.genecards.org/cgi-bin/ca
RNA Gene	18	GC22P041900	4.13	https://www.genecards.org/cgi-bin/ca
Protein Coding	41	GC01P153627	4.13	https://www.genecards.org/cgi-bin/ca
Protein Coding	41	GC13P095433	4.13	https://www.genecards.org/cgi-bin/ca

RNA Gene	18	GC19M045663	4.13	https://www.genecards.org/cgi-bin/ca
Protein Coding	37	GC22M045390	4.12	https://www.genecards.org/cgi-bin/ca
Protein Coding	41	GC17M075130	4.11	https://www.genecards.org/cgi-bin/ca
Protein Coding	47	GC11M066848	4.11	https://www.genecards.org/cgi-bin/ca
Protein Coding	43	GC20P050190	4.11	https://www.genecards.org/cgi-bin/ca
Protein Coding	43	GC03P122055	4.1	https://www.genecards.org/cgi-bin/ca
Protein Coding	35	GC17M055392	4.09	https://www.genecards.org/cgi-bin/ca
Protein Coding	50	GC06M137197	4.09	https://www.genecards.org/cgi-bin/ca
Protein Coding	45	GC04M177430	4.08	https://www.genecards.org/cgi-bin/ca
Protein Coding	38	GC11P001074	4.08	https://www.genecards.org/cgi-bin/ca
RNA Gene	21	GC18P058451	4.07	https://www.genecards.org/cgi-bin/ca
Protein Coding	40	GC0XM038269	4.07	https://www.genecards.org/cgi-bin/ca
Protein Coding	47	GC0XP071215	4.07	https://www.genecards.org/cgi-bin/ca
Protein Coding	40	GC09P134864	4.06	https://www.genecards.org/cgi-bin/ca
Protein Coding	41	GC01M092474	4.06	https://www.genecards.org/cgi-bin/ca
Protein Coding	45	GC07P066075	4.05	https://www.genecards.org/cgi-bin/ca
Protein Coding	43	GC02M128236	4.05	https://www.genecards.org/cgi-bin/ca
Protein Coding	33	GC19P044699	4.05	https://www.genecards.org/cgi-bin/ca
Protein Coding	35	GC01M146018	4.04	https://www.genecards.org/cgi-bin/ca
Protein Coding	45	GC02M068143	4.04	https://www.genecards.org/cgi-bin/ca
Protein Coding	44	GC10M073436	4.04	https://www.genecards.org/cgi-bin/ca
Protein Coding	39	GC03M108160	4.04	https://www.genecards.org/cgi-bin/ca
Protein Coding	45	GC07M142908	4.04	https://www.genecards.org/cgi-bin/ca
Protein Coding	50	GC15M064155	4.03	https://www.genecards.org/cgi-bin/ca
Protein Coding	41	GC11M000829	4.02	https://www.genecards.org/cgi-bin/ca
Protein Coding	43	GC11P070398	4.02	https://www.genecards.org/cgi-bin/ca
Protein Coding	44	GC14P024365	4.01	https://www.genecards.org/cgi-bin/ca
Protein Coding	40	GC03P149129	4.01	https://www.genecards.org/cgi-bin/ca
Protein Coding	44	GC13M046771	4.01	https://www.genecards.org/cgi-bin/ca
Protein Coding	45	GC0XP154389	4.01	https://www.genecards.org/cgi-bin/ca
Protein Coding	43	GC09M125147	4	https://www.genecards.org/cgi-bin/ca
Protein Coding	50	GC12P111766	4	https://www.genecards.org/cgi-bin/ca
Protein Coding	49	GC05M001392	4	https://www.genecards.org/cgi-bin/ca
Protein Coding	45	GC0XM111293	4	https://www.genecards.org/cgi-bin/ca
Protein Coding	40	GC19P001494	3.99	https://www.genecards.org/cgi-bin/ca
Protein Coding	44	GC06P047256	3.99	https://www.genecards.org/cgi-bin/ca
Protein Coding	44	GC08P022440	3.99	https://www.genecards.org/cgi-bin/ca
Protein Coding	43	GC16P068119	3.99	https://www.genecards.org/cgi-bin/ca
Protein Coding	39	GC14P064465	3.99	https://www.genecards.org/cgi-bin/ca
Protein Coding	38	GC06P131126	3.99	https://www.genecards.org/cgi-bin/ca
Protein Coding	29	GC01P037806	3.99	https://www.genecards.org/cgi-bin/ca
Protein Coding	48	GC02P218125	3.99	https://www.genecards.org/cgi-bin/ca
Protein Coding	38	GC08P109536	3.98	https://www.genecards.org/cgi-bin/ca
Protein Coding	51	GC19P012938	3.98	https://www.genecards.org/cgi-bin/ca
Protein Coding	45	GC07P030911	3.97	https://www.genecards.org/cgi-bin/ca
Protein Coding	39	GC20P045174	3.97	https://www.genecards.org/cgi-bin/ca
Protein Coding	40	GC04P143513	3.97	https://www.genecards.org/cgi-bin/ca
Protein Coding	41	GC11M111872	3.96	https://www.genecards.org/cgi-bin/ca
Protein Coding	47	GC09M033431	3.96	https://www.genecards.org/cgi-bin/ca
Protein Coding	50	GC17M081509	3.95	https://www.genecards.org/cgi-bin/ca
Protein Coding	39	GC07P130486	3.95	https://www.genecards.org/cgi-bin/ca
Protein Coding	45	GC01M209614	3.95	https://www.genecards.org/cgi-bin/ca
Protein Coding	42	GC17M040818	3.95	https://www.genecards.org/cgi-bin/ca
Protein Coding	47	GC12P051951	3.94	https://www.genecards.org/cgi-bin/ca

Protein Coding	43	GC01M203148	3.93	https://www.genecards.org/cgi-bin/ca
Protein Coding	41	GC01M156308	3.93	https://www.genecards.org/cgi-bin/ca
Protein Coding	41	GC05P069093	3.92	https://www.genecards.org/cgi-bin/ca
Protein Coding	40	GC22M042796	3.92	https://www.genecards.org/cgi-bin/ca
Protein Coding	39	GC01P100824	3.92	https://www.genecards.org/cgi-bin/ca
Protein Coding	28	GC10M103390	3.92	https://www.genecards.org/cgi-bin/ca
Protein Coding	47	GC07M002906	3.91	https://www.genecards.org/cgi-bin/ca
Protein Coding	47	GC05P083471	3.91	https://www.genecards.org/cgi-bin/ca
Protein Coding	42	GC20P063272	3.9	https://www.genecards.org/cgi-bin/ca
Protein Coding	37	GC11M061897	3.9	https://www.genecards.org/cgi-bin/ca
Protein Coding	36	GC11M061114	3.9	https://www.genecards.org/cgi-bin/ca
Protein Coding	36	GC01M246840	3.9	https://www.genecards.org/cgi-bin/ca
Protein Coding	34	GC06M149724	3.9	https://www.genecards.org/cgi-bin/ca
Protein Coding	34	GC07M075986	3.9	https://www.genecards.org/cgi-bin/ca
Protein Coding	40	GC01P182839	3.9	https://www.genecards.org/cgi-bin/ca
Protein Coding	40	GC14P021290	3.89	https://www.genecards.org/cgi-bin/ca
Protein Coding	44	GC11P064234	3.89	https://www.genecards.org/cgi-bin/ca
Protein Coding	36	GC19M006729	3.89	https://www.genecards.org/cgi-bin/ca
Protein Coding	36	GC11P000518	3.88	https://www.genecards.org/cgi-bin/ca
Protein Coding	42	GC17M043952	3.87	https://www.genecards.org/cgi-bin/ca
Protein Coding	45	GC0XP023784	3.87	https://www.genecards.org/cgi-bin/ca
Protein Coding	46	GC07M096120	3.86	https://www.genecards.org/cgi-bin/ca
Protein Coding	40	GC15M064963	3.86	https://www.genecards.org/cgi-bin/ca
Protein Coding	39	GC06M029672	3.85	https://www.genecards.org/cgi-bin/ca
Protein Coding	44	GC15M078594	3.85	https://www.genecards.org/cgi-bin/ca
Protein Coding	37	GC14P088384	3.85	https://www.genecards.org/cgi-bin/ca
Protein Coding	43	GC21P042199	3.85	https://www.genecards.org/cgi-bin/ca
Protein Coding	53	GC0XM101349	3.84	https://www.genecards.org/cgi-bin/ca
Protein Coding	44	GC10P070815	3.84	https://www.genecards.org/cgi-bin/ca
Protein Coding	48	GC06P154075	3.84	https://www.genecards.org/cgi-bin/ca
Protein Coding	45	GC01P050960	3.84	https://www.genecards.org/cgi-bin/ca
Protein Coding	47	GC02P218382	3.84	https://www.genecards.org/cgi-bin/ca
Protein Coding	39	GC14M067727	3.84	https://www.genecards.org/cgi-bin/ca
Protein Coding	42	GC10P086957	3.84	https://www.genecards.org/cgi-bin/ca
Protein Coding	40	GC02P068365	3.84	https://www.genecards.org/cgi-bin/ca
Protein Coding	32	GC15P024954	3.84	https://www.genecards.org/cgi-bin/ca
Protein Coding	40	GC16P072097	3.83	https://www.genecards.org/cgi-bin/ca
Protein Coding	40	GC17P036103	3.83	https://www.genecards.org/cgi-bin/ca
Protein Coding	52	GC10M100188	3.83	https://www.genecards.org/cgi-bin/ca
Protein Coding	41	GC01M006222	3.83	https://www.genecards.org/cgi-bin/ca
Protein Coding	45	GC06P105993	3.82	https://www.genecards.org/cgi-bin/ca
Protein Coding	44	GC14M024573	3.82	https://www.genecards.org/cgi-bin/ca
Protein Coding	45	GC01M167809	3.82	https://www.genecards.org/cgi-bin/ca
Protein Coding	51	GC02M029156	3.82	https://www.genecards.org/cgi-bin/ca
Protein Coding	40	GC02M111611	3.81	https://www.genecards.org/cgi-bin/ca
Protein Coding	38	GC05M179614	3.81	https://www.genecards.org/cgi-bin/ca
Protein Coding	39	GC0XM129447	3.81	https://www.genecards.org/cgi-bin/ca
Protein Coding	40	GC09P114827	3.8	https://www.genecards.org/cgi-bin/ca
Protein Coding	45	GC01M091680	3.8	https://www.genecards.org/cgi-bin/ca
Protein Coding	41	GC09M132261	3.8	https://www.genecards.org/cgi-bin/ca
Protein Coding	40	GC02P176122	3.8	https://www.genecards.org/cgi-bin/ca
Protein Coding	45	GC01P161505	3.79	https://www.genecards.org/cgi-bin/ca
Protein Coding	49	GC22M036281	3.79	https://www.genecards.org/cgi-bin/ca
Protein Coding	44	GC09P034650	3.78	https://www.genecards.org/cgi-bin/ca

Protein Coding	43	GC05M177511	3.78	https://www.genecards.org/cgi-bin/ca
Protein Coding	45	GC08P039891	3.77	https://www.genecards.org/cgi-bin/ca
Protein Coding	39	GC11P067057	3.76	https://www.genecards.org/cgi-bin/ca
Protein Coding	46	GC02P015949	3.75	https://www.genecards.org/cgi-bin/ca
Protein Coding	44	GC17P008025	3.75	https://www.genecards.org/cgi-bin/ca
Protein Coding	44	GC06M139371	3.75	https://www.genecards.org/cgi-bin/ca
Protein Coding	46	GC11M064273	3.75	https://www.genecards.org/cgi-bin/ca
Protein Coding	47	GC02M010432	3.75	https://www.genecards.org/cgi-bin/ca
Protein Coding	40	GC04P145607	3.74	https://www.genecards.org/cgi-bin/ca
Protein Coding	44	GC12M054230	3.74	https://www.genecards.org/cgi-bin/ca
Protein Coding	45	GC03P046356	3.74	https://www.genecards.org/cgi-bin/ca
Protein Coding	40	GC14M080197	3.73	https://www.genecards.org/cgi-bin/ca
Protein Coding	44	GC0XM053532	3.73	https://www.genecards.org/cgi-bin/ca
Protein Coding	45	GC15P076993	3.73	https://www.genecards.org/cgi-bin/ca
Protein Coding	37	GC09M034662	3.72	https://www.genecards.org/cgi-bin/ca
Protein Coding	40	GC06P033277	3.71	https://www.genecards.org/cgi-bin/ca
Protein Coding	37	GC07P000857	3.71	https://www.genecards.org/cgi-bin/ca
Protein Coding	44	GC04M038828	3.7	https://www.genecards.org/cgi-bin/ca
Protein Coding	41	GC04P077511	3.7	https://www.genecards.org/cgi-bin/ca
Protein Coding	47	GC03M130678	3.7	https://www.genecards.org/cgi-bin/ca
Protein Coding	44	GC04M140559	3.69	https://www.genecards.org/cgi-bin/ca
Protein Coding	43	GC17P042535	3.68	https://www.genecards.org/cgi-bin/ca
Protein Coding	45	GC03M187721	3.68	https://www.genecards.org/cgi-bin/ca
Protein Coding	39	GC20M057495	3.68	https://www.genecards.org/cgi-bin/ca
Protein Coding	41	GC19P054953	3.67	https://www.genecards.org/cgi-bin/ca
Protein Coding	38	GC19M056810	3.67	https://www.genecards.org/cgi-bin/ca
Protein Coding	46	GC02M177624	3.67	https://www.genecards.org/cgi-bin/ca
Protein Coding	48	GC06M134169	3.67	https://www.genecards.org/cgi-bin/ca
Protein Coding	31	GC06M026018	3.66	https://www.genecards.org/cgi-bin/ca
Protein Coding	39	GC01M030711	3.66	https://www.genecards.org/cgi-bin/ca
Protein Coding	31	GC01P085581	3.66	https://www.genecards.org/cgi-bin/ca
Protein Coding	38	GC11P044045	3.65	https://www.genecards.org/cgi-bin/ca
Protein Coding	35	GC22P028803	3.65	https://www.genecards.org/cgi-bin/ca
Protein Coding	36	GC06P041780	3.64	https://www.genecards.org/cgi-bin/ca
Protein Coding	31	GC03P187197	3.64	https://www.genecards.org/cgi-bin/ca
Protein Coding	29	GC0XP052368	3.64	https://www.genecards.org/cgi-bin/ca
Protein Coding	48	GC06P044219	3.63	https://www.genecards.org/cgi-bin/ca
Protein Coding	48	GC07P074773	3.63	https://www.genecards.org/cgi-bin/ca
RNA Gene	16	GC14P104795	3.62	https://www.genecards.org/cgi-bin/ca
Protein Coding	43	GC07M027263	3.62	https://www.genecards.org/cgi-bin/ca
Protein Coding	45	GC03P043707	3.62	https://www.genecards.org/cgi-bin/ca
Protein Coding	42	GC07M095404	3.62	https://www.genecards.org/cgi-bin/ca
Protein Coding	43	GC03M155821	3.61	https://www.genecards.org/cgi-bin/ca
Protein Coding	44	GC07P148697	3.61	https://www.genecards.org/cgi-bin/ca
Protein Coding	34	GC04P069932	3.6	https://www.genecards.org/cgi-bin/ca
Protein Coding	43	GC19M039834	3.6	https://www.genecards.org/cgi-bin/ca
Protein Coding	45	GC14M056799	3.6	https://www.genecards.org/cgi-bin/ca
Protein Coding	46	GC09M036828	3.6	https://www.genecards.org/cgi-bin/ca
Protein Coding	46	GC11P009573	3.59	https://www.genecards.org/cgi-bin/ca
Protein Coding	46	GC10P094762	3.59	https://www.genecards.org/cgi-bin/ca
Protein Coding	40	GC02M196833	3.59	https://www.genecards.org/cgi-bin/ca
Protein Coding	41	GC22P040857	3.59	https://www.genecards.org/cgi-bin/ca
Protein Coding	29	GC01P156337	3.59	https://www.genecards.org/cgi-bin/ca
Protein Coding	43	GC09M014077	3.58	https://www.genecards.org/cgi-bin/ca

Protein Coding	47	GC01P023019	3.58	https://www.genecards.org/cgi-bin/ca
Protein Coding	45	GC15P040405	3.57	https://www.genecards.org/cgi-bin/ca
Protein Coding	48	GC22P029603	3.57	https://www.genecards.org/cgi-bin/ca
Protein Coding	40	GC17M048540	3.57	https://www.genecards.org/cgi-bin/ca
Protein Coding	48	GC02M088637	3.57	https://www.genecards.org/cgi-bin/ca
Protein Coding	37	GC0XP101408	3.57	https://www.genecards.org/cgi-bin/ca
Protein Coding	39	GC0XM119805	3.56	https://www.genecards.org/cgi-bin/ca
Protein Coding	45	GC17P042287	3.56	https://www.genecards.org/cgi-bin/ca
Protein Coding	45	GC17P049495	3.56	https://www.genecards.org/cgi-bin/ca
Protein Coding	46	GC0XP012867	3.56	https://www.genecards.org/cgi-bin/ca
Protein Coding	41	GC17P061451	3.56	https://www.genecards.org/cgi-bin/ca
Protein Coding	46	GC11M033704	3.56	https://www.genecards.org/cgi-bin/ca
Protein Coding	43	GC13P113297	3.56	https://www.genecards.org/cgi-bin/ca
Protein Coding	44	GC17P042900	3.56	https://www.genecards.org/cgi-bin/ca
Protein Coding	40	GC12M004413	3.56	https://www.genecards.org/cgi-bin/ca
Protein Coding	44	GC09M019375	3.55	https://www.genecards.org/cgi-bin/ca
Protein Coding	44	GC03P015621	3.55	https://www.genecards.org/cgi-bin/ca
Protein Coding	41	GC09P017906	3.54	https://www.genecards.org/cgi-bin/ca
Protein Coding	40	GC01M201896	3.54	https://www.genecards.org/cgi-bin/ca
Protein Coding	40	GC06P047388	3.54	https://www.genecards.org/cgi-bin/ca
RNA Gene	16	GC0XM147910	3.54	https://www.genecards.org/cgi-bin/ca
Protein Coding	39	GC08M006870	3.54	https://www.genecards.org/cgi-bin/ca
Protein Coding	47	GC07P050303	3.54	https://www.genecards.org/cgi-bin/ca
Protein Coding	45	GC05P052989	3.53	https://www.genecards.org/cgi-bin/ca
Protein Coding	45	GC04M120055	3.52	https://www.genecards.org/cgi-bin/ca
Protein Coding	45	GC16P010879	3.52	https://www.genecards.org/cgi-bin/ca
Protein Coding	34	GC01M032864	3.52	https://www.genecards.org/cgi-bin/ca
Protein Coding	39	GC02M086213	3.51	https://www.genecards.org/cgi-bin/ca
Protein Coding	43	GC06P033419	3.51	https://www.genecards.org/cgi-bin/ca
Protein Coding	47	GC01M036466	3.51	https://www.genecards.org/cgi-bin/ca
Protein Coding	48	GC16P002537	3.51	https://www.genecards.org/cgi-bin/ca
Protein Coding	49	GC16P089919	3.51	https://www.genecards.org/cgi-bin/ca
Protein Coding	44	GC06M033572	3.51	https://www.genecards.org/cgi-bin/ca
Protein Coding	47	GC17M044282	3.5	https://www.genecards.org/cgi-bin/ca
Protein Coding	33	GC06P073362	3.5	https://www.genecards.org/cgi-bin/ca
Protein Coding	47	GC19P006772	3.5	https://www.genecards.org/cgi-bin/ca
Protein Coding	44	GC16P086567	3.5	https://www.genecards.org/cgi-bin/ca
Protein Coding	35	GC04P001872	3.49	https://www.genecards.org/cgi-bin/ca
Protein Coding	35	GC19M045782	3.49	https://www.genecards.org/cgi-bin/ca
Protein Coding	47	GC16M088643	3.49	https://www.genecards.org/cgi-bin/ca
Protein Coding	48	GC19M038878	3.49	https://www.genecards.org/cgi-bin/ca
Protein Coding	40	GC17M042185	3.48	https://www.genecards.org/cgi-bin/ca
Protein Coding	35	GC09M005309	3.48	https://www.genecards.org/cgi-bin/ca
Protein Coding	40	GC04M185399	3.48	https://www.genecards.org/cgi-bin/ca
Protein Coding	47	GC09P035782	3.48	https://www.genecards.org/cgi-bin/ca
Protein Coding	44	GC16P011965	3.46	https://www.genecards.org/cgi-bin/ca
Protein Coding	44	GC06M031272	3.45	https://www.genecards.org/cgi-bin/ca
Protein Coding	47	GC11P128891	3.45	https://www.genecards.org/cgi-bin/ca
Protein Coding	44	GC04P002855	3.45	https://www.genecards.org/cgi-bin/ca
Protein Coding	50	GC01P026540	3.44	https://www.genecards.org/cgi-bin/ca
Protein Coding	44	GC0XP152830	3.43	https://www.genecards.org/cgi-bin/ca
Protein Coding	41	GC12P006970	3.43	https://www.genecards.org/cgi-bin/ca
Protein Coding	45	GC12M057009	3.43	https://www.genecards.org/cgi-bin/ca
Protein Coding	46	GC04M023755	3.43	https://www.genecards.org/cgi-bin/ca

Protein Coding	44	GC01P207496	3.43	https://www.genecards.org/cgi-bin/ca
Protein Coding	42	GC02M149569	3.42	https://www.genecards.org/cgi-bin/ca
Protein Coding	40	GC02P073233	3.42	https://www.genecards.org/cgi-bin/ca
Protein Coding	43	GC03M108043	3.42	https://www.genecards.org/cgi-bin/ca
Protein Coding	44	GC14P104635	3.42	https://www.genecards.org/cgi-bin/ca
Protein Coding	37	GC08P135457	3.41	https://www.genecards.org/cgi-bin/ca
Protein Coding	41	GC13P113208	3.41	https://www.genecards.org/cgi-bin/ca
Protein Coding	40	GC06P144285	3.41	https://www.genecards.org/cgi-bin/ca
Protein Coding	42	GC17P004998	3.41	https://www.genecards.org/cgi-bin/ca
Protein Coding	37	GC16P031576	3.41	https://www.genecards.org/cgi-bin/ca
Protein Coding	37	GC02M018562	3.41	https://www.genecards.org/cgi-bin/ca
Protein Coding	42	GC12M007471	3.41	https://www.genecards.org/cgi-bin/ca
Protein Coding	50	GC06P131473	3.41	https://www.genecards.org/cgi-bin/ca
Protein Coding	44	GC11M063430	3.41	https://www.genecards.org/cgi-bin/ca
Protein Coding	43	GC05P102753	3.41	https://www.genecards.org/cgi-bin/ca
Protein Coding	43	GC0XM155489	3.41	https://www.genecards.org/cgi-bin/ca
Protein Coding	42	GC06M042216	3.41	https://www.genecards.org/cgi-bin/ca
Protein Coding	45	GC21M043772	3.4	https://www.genecards.org/cgi-bin/ca
Protein Coding	43	GC02P102311	3.4	https://www.genecards.org/cgi-bin/ca
Protein Coding	45	GC06M031879	3.4	https://www.genecards.org/cgi-bin/ca
Protein Coding	50	GC01P056645	3.4	https://www.genecards.org/cgi-bin/ca
Protein Coding	35	GC05P168487	3.4	https://www.genecards.org/cgi-bin/ca
Protein Coding	33	GC0XM002934	3.4	https://www.genecards.org/cgi-bin/ca
Protein Coding	50	GC12M057095	3.4	https://www.genecards.org/cgi-bin/ca
Protein Coding	44	GC01M161541	3.39	https://www.genecards.org/cgi-bin/ca
Protein Coding	45	GC18P031498	3.39	https://www.genecards.org/cgi-bin/ca
Protein Coding	46	GC11M064746	3.39	https://www.genecards.org/cgi-bin/ca
Protein Coding	44	GC05M140631	3.39	https://www.genecards.org/cgi-bin/ca
Protein Coding	42	GC10P026697	3.39	https://www.genecards.org/cgi-bin/ca
Protein Coding	41	GC0XM153701	3.38	https://www.genecards.org/cgi-bin/ca
Protein Coding	47	GC08P027490	3.38	https://www.genecards.org/cgi-bin/ca
Protein Coding	47	GC04M038797	3.38	https://www.genecards.org/cgi-bin/ca
Protein Coding	38	GC22M035738	3.38	https://www.genecards.org/cgi-bin/ca
Protein Coding	46	GC0XM153947	3.37	https://www.genecards.org/cgi-bin/ca
Protein Coding	41	GC0XP154428	3.37	https://www.genecards.org/cgi-bin/ca
Protein Coding	42	GC06M042295	3.37	https://www.genecards.org/cgi-bin/ca
Protein Coding	45	GC01M019975	3.37	https://www.genecards.org/cgi-bin/ca
Protein Coding	41	GC22M045386	3.36	https://www.genecards.org/cgi-bin/ca
Protein Coding	44	GC05M150443	3.36	https://www.genecards.org/cgi-bin/ca
Protein Coding	43	GC01M024899	3.36	https://www.genecards.org/cgi-bin/ca
Protein Coding	45	GC12P098515	3.35	https://www.genecards.org/cgi-bin/ca
Protein Coding	43	GC01M214348	3.35	https://www.genecards.org/cgi-bin/ca
Protein Coding	50	GC06M046704	3.35	https://www.genecards.org/cgi-bin/ca
RNA Gene	21	GC13M050048	3.35	https://www.genecards.org/cgi-bin/ca
Protein Coding	44	GC01M161229	3.34	https://www.genecards.org/cgi-bin/ca
Protein Coding	43	GC17M004897	3.34	https://www.genecards.org/cgi-bin/ca
Protein Coding	46	GC02P190880	3.34	https://www.genecards.org/cgi-bin/ca
Protein Coding	44	GC10M079937	3.33	https://www.genecards.org/cgi-bin/ca
Protein Coding	44	GC21M034446	3.33	https://www.genecards.org/cgi-bin/ca
Protein Coding	44	GC01P067138	3.33	https://www.genecards.org/cgi-bin/ca
Protein Coding	44	GC01M031365	3.33	https://www.genecards.org/cgi-bin/ca
Protein Coding	47	GC0XP012924	3.33	https://www.genecards.org/cgi-bin/ca
Protein Coding	34	GC14M095408	3.32	https://www.genecards.org/cgi-bin/ca
Protein Coding	43	GC01P034792	3.32	https://www.genecards.org/cgi-bin/ca

Protein Coding	45	GC21P045405	3.31	https://www.genecards.org/cgi-bin/ca
Protein Coding	41	GC12M055725	3.3	https://www.genecards.org/cgi-bin/ca
Protein Coding	38	GC08M047759	3.3	https://www.genecards.org/cgi-bin/ca
Protein Coding	41	GC18P000895	3.3	https://www.genecards.org/cgi-bin/ca
Protein Coding	44	GC16M088813	3.3	https://www.genecards.org/cgi-bin/ca
Protein Coding	41	GC07M156994	3.3	https://www.genecards.org/cgi-bin/ca
Protein Coding	44	GC17M059674	3.3	https://www.genecards.org/cgi-bin/ca
Protein Coding	42	GC04P001011	3.29	https://www.genecards.org/cgi-bin/ca
Protein Coding	45	GC05P076818	3.29	https://www.genecards.org/cgi-bin/ca
Protein Coding	48	GC12P109573	3.29	https://www.genecards.org/cgi-bin/ca
Protein Coding	44	GC01M011026	3.28	https://www.genecards.org/cgi-bin/ca
Protein Coding	45	GC22M017734	3.28	https://www.genecards.org/cgi-bin/ca
Protein Coding	44	GC02P233311	3.27	https://www.genecards.org/cgi-bin/ca
Protein Coding	43	GC10P058385	3.27	https://www.genecards.org/cgi-bin/ca
Protein Coding	42	GC05M006599	3.27	https://www.genecards.org/cgi-bin/ca
Protein Coding	44	GC04P145481	3.27	https://www.genecards.org/cgi-bin/ca
Protein Coding	47	GC04P127880	3.26	https://www.genecards.org/cgi-bin/ca
Protein Coding	44	GC01M045329	3.26	https://www.genecards.org/cgi-bin/ca
Protein Coding	43	GC07P030978	3.25	https://www.genecards.org/cgi-bin/ca
Protein Coding	47	GC03P010211	3.25	https://www.genecards.org/cgi-bin/ca
Protein Coding	50	GC02M221418	3.24	https://www.genecards.org/cgi-bin/ca
RNA Gene	21	GC01P040757	3.23	https://www.genecards.org/cgi-bin/ca
Protein Coding	44	GC17M017810	3.23	https://www.genecards.org/cgi-bin/ca
Protein Coding	44	GC02P227164	3.23	https://www.genecards.org/cgi-bin/ca
Protein Coding	45	GC08M115408	3.23	https://www.genecards.org/cgi-bin/ca
Protein Coding	35	GC0XP151396	3.23	https://www.genecards.org/cgi-bin/ca
Protein Coding	43	GC15M029699	3.22	https://www.genecards.org/cgi-bin/ca
Protein Coding	45	GC17M040556	3.22	https://www.genecards.org/cgi-bin/ca
Protein Coding	44	GC19M047870	3.21	https://www.genecards.org/cgi-bin/ca
Protein Coding	31	GC16P069132	3.21	https://www.genecards.org/cgi-bin/ca
Protein Coding	31	GC13M036302	3.21	https://www.genecards.org/cgi-bin/ca
Protein Coding	46	GC12P011649	3.2	https://www.genecards.org/cgi-bin/ca
Protein Coding	47	GC03P046227	3.2	https://www.genecards.org/cgi-bin/ca
Protein Coding	38	GC01M235661	3.2	https://www.genecards.org/cgi-bin/ca
Protein Coding	48	GC11M000215	3.2	https://www.genecards.org/cgi-bin/ca
Protein Coding	48	GC03P128749	3.2	https://www.genecards.org/cgi-bin/ca
Protein Coding	44	GC12P102957	3.19	https://www.genecards.org/cgi-bin/ca
Protein Coding	43	GC17M016029	3.19	https://www.genecards.org/cgi-bin/ca
Protein Coding	36	GC0XM008528	3.19	https://www.genecards.org/cgi-bin/ca
Protein Coding	36	GC08M022138	3.19	https://www.genecards.org/cgi-bin/ca
Protein Coding	44	GC16P053737	3.19	https://www.genecards.org/cgi-bin/ca
Protein Coding	41	GC05P171419	3.19	https://www.genecards.org/cgi-bin/ca
Protein Coding	38	GC01P150549	3.19	https://www.genecards.org/cgi-bin/ca
Protein Coding	46	GC11P092969	3.18	https://www.genecards.org/cgi-bin/ca
Protein Coding	41	GC01M043363	3.18	https://www.genecards.org/cgi-bin/ca
Protein Coding	43	GC0XM066595	3.17	https://www.genecards.org/cgi-bin/ca
Protein Coding	38	GC11P000269	3.17	https://www.genecards.org/cgi-bin/ca
Protein Coding	41	GC16P003611	3.17	https://www.genecards.org/cgi-bin/ca
Protein Coding	47	GC16P089912	3.17	https://www.genecards.org/cgi-bin/ca
Protein Coding	47	GC01M023845	3.17	https://www.genecards.org/cgi-bin/ca
Protein Coding	43	GC20P018507	3.17	https://www.genecards.org/cgi-bin/ca
Protein Coding	44	GC04M047596	3.16	https://www.genecards.org/cgi-bin/ca
Protein Coding	48	GC07P129463	3.16	https://www.genecards.org/cgi-bin/ca
Protein Coding	46	GC17P050055	3.16	https://www.genecards.org/cgi-bin/ca

Protein Coding	45	GC17P002593	3.16	https://www.genecards.org/cgi-bin/ca
Protein Coding	44	GC16M074746	3.15	https://www.genecards.org/cgi-bin/ca
Protein Coding	48	GC10P094938	3.15	https://www.genecards.org/cgi-bin/ca
Protein Coding	45	GC02M061445	3.15	https://www.genecards.org/cgi-bin/ca
Protein Coding	41	GC04P102868	3.15	https://www.genecards.org/cgi-bin/ca
Protein Coding	49	GC12M117208	3.15	https://www.genecards.org/cgi-bin/ca
Protein Coding	43	GC05P055840	3.15	https://www.genecards.org/cgi-bin/ca
Protein Coding	45	GC09M107484	3.15	https://www.genecards.org/cgi-bin/ca
Protein Coding	46	GC01M023801	3.15	https://www.genecards.org/cgi-bin/ca
Protein Coding	45	GC16P031543	3.15	https://www.genecards.org/cgi-bin/ca
Protein Coding	39	GC16P001351	3.14	https://www.genecards.org/cgi-bin/ca
Protein Coding	48	GC07M128392	3.14	https://www.genecards.org/cgi-bin/ca
Protein Coding	47	GC13M110148	3.14	https://www.genecards.org/cgi-bin/ca
RNA Gene	16	GC0XM074227	3.14	https://www.genecards.org/cgi-bin/ca
Protein Coding	41	GC21M044246	3.13	https://www.genecards.org/cgi-bin/ca
Protein Coding	45	GC18M006941	3.13	https://www.genecards.org/cgi-bin/ca
Protein Coding	42	GC12P053268	3.13	https://www.genecards.org/cgi-bin/ca
Protein Coding	44	GC11P001839	3.13	https://www.genecards.org/cgi-bin/ca
RNA Gene	16	GC0XP050008	3.13	https://www.genecards.org/cgi-bin/ca
Protein Coding	43	GC12M008955	3.12	https://www.genecards.org/cgi-bin/ca
Protein Coding	44	GC15M079833	3.12	https://www.genecards.org/cgi-bin/ca
Protein Coding	51	GC06M090513	3.11	https://www.genecards.org/cgi-bin/ca
Protein Coding	39	GC11P034105	3.11	https://www.genecards.org/cgi-bin/ca
Protein Coding	35	GC06M032626	3.11	https://www.genecards.org/cgi-bin/ca
Protein Coding	37	GC02M043193	3.1	https://www.genecards.org/cgi-bin/ca
Protein Coding	41	GC11M068052	3.1	https://www.genecards.org/cgi-bin/ca
Protein Coding	48	GC12M013893	3.09	https://www.genecards.org/cgi-bin/ca
Protein Coding	43	GC02P032915	3.09	https://www.genecards.org/cgi-bin/ca
Protein Coding	38	GC16M075628	3.09	https://www.genecards.org/cgi-bin/ca
Protein Coding	39	GC10P068827	3.08	https://www.genecards.org/cgi-bin/ca
Protein Coding	37	GC01P152881	3.08	https://www.genecards.org/cgi-bin/ca
Protein Coding	45	GC19P023315	3.08	https://www.genecards.org/cgi-bin/ca
Protein Coding	44	GC06M032821	3.07	https://www.genecards.org/cgi-bin/ca
Protein Coding	42	GC11P013276	3.07	https://www.genecards.org/cgi-bin/ca
Protein Coding	47	GC13P026254	3.07	https://www.genecards.org/cgi-bin/ca
Protein Coding	47	GC12P032679	3.07	https://www.genecards.org/cgi-bin/ca
Protein Coding	44	GC12P119178	3.07	https://www.genecards.org/cgi-bin/ca
Protein Coding	46	GC13M080335	3.06	https://www.genecards.org/cgi-bin/ca
Protein Coding	51	GC22M037227	3.06	https://www.genecards.org/cgi-bin/ca
Protein Coding	40	GC06P151239	3.06	https://www.genecards.org/cgi-bin/ca
Protein Coding	49	GC19P018153	3.06	https://www.genecards.org/cgi-bin/ca
Protein Coding	42	GC01M153391	3.06	https://www.genecards.org/cgi-bin/ca
Protein Coding	43	GC12P005432	3.05	https://www.genecards.org/cgi-bin/ca
Protein Coding	43	GC01M202300	3.05	https://www.genecards.org/cgi-bin/ca
Protein Coding	45	GC20M057168	3.04	https://www.genecards.org/cgi-bin/ca
Protein Coding	45	GC10P058334	3.04	https://www.genecards.org/cgi-bin/ca
Protein Coding	39	GC07M030496	3.03	https://www.genecards.org/cgi-bin/ca
Protein Coding	44	GC10P093993	3.03	https://www.genecards.org/cgi-bin/ca
Protein Coding	40	GC19P041750	3.02	https://www.genecards.org/cgi-bin/ca
Protein Coding	41	GC01M027379	3.02	https://www.genecards.org/cgi-bin/ca
RNA Gene	18	GC05P149732	3.02	https://www.genecards.org/cgi-bin/ca
Protein Coding	43	GC19M005691	3.02	https://www.genecards.org/cgi-bin/ca
Protein Coding	45	GC11M076186	3.01	https://www.genecards.org/cgi-bin/ca
Protein Coding	39	GC07M027830	3.01	https://www.genecards.org/cgi-bin/ca

Protein Coding	50	GC0XM154010	3.01	https://www.genecards.org/cgi-bin/ca
Protein Coding	44	GC07M000497	3.01	https://www.genecards.org/cgi-bin/ca
Protein Coding	42	GC20P035182	3	https://www.genecards.org/cgi-bin/ca
Protein Coding	48	GC17M065528	3	https://www.genecards.org/cgi-bin/ca
Protein Coding	46	GC02M085544	3	https://www.genecards.org/cgi-bin/ca
Protein Coding	43	GC16P053433	3	https://www.genecards.org/cgi-bin/ca
Protein Coding	43	GC01M006460	2.99	https://www.genecards.org/cgi-bin/ca
Protein Coding	42	GC07M030651	2.99	https://www.genecards.org/cgi-bin/ca
Protein Coding	50	GC04M103586	2.98	https://www.genecards.org/cgi-bin/ca
Protein Coding	44	GC06P024779	2.98	https://www.genecards.org/cgi-bin/ca
Protein Coding	39	GC11M128965	2.97	https://www.genecards.org/cgi-bin/ca
Protein Coding	44	GC03P122325	2.97	https://www.genecards.org/cgi-bin/ca
Protein Coding	43	GC01P197170	2.97	https://www.genecards.org/cgi-bin/ca
Protein Coding	39	GC06M043145	2.97	https://www.genecards.org/cgi-bin/ca
Protein Coding	39	GC01P050025	2.96	https://www.genecards.org/cgi-bin/ca
Protein Coding	37	GC19M011451	2.96	https://www.genecards.org/cgi-bin/ca
Protein Coding	41	GC05P138439	2.96	https://www.genecards.org/cgi-bin/ca
Protein Coding	44	GC11P075836	2.96	https://www.genecards.org/cgi-bin/ca
Protein Coding	44	GC08M119556	2.95	https://www.genecards.org/cgi-bin/ca
Protein Coding	43	GC19P047819	2.94	https://www.genecards.org/cgi-bin/ca
Protein Coding	40	GC02P232539	2.94	https://www.genecards.org/cgi-bin/ca
Protein Coding	42	GC0XP016141	2.94	https://www.genecards.org/cgi-bin/ca
RNA Gene	21	GC06M144002	2.94	https://www.genecards.org/cgi-bin/ca
Protein Coding	44	GC03P155870	2.94	https://www.genecards.org/cgi-bin/ca
Protein Coding	45	GC14P067701	2.93	https://www.genecards.org/cgi-bin/ca
Protein Coding	41	GC03M133600	2.93	https://www.genecards.org/cgi-bin/ca
Protein Coding	44	GC17P080544	2.92	https://www.genecards.org/cgi-bin/ca
Protein Coding	31	GC14M109532	2.92	https://www.genecards.org/cgi-bin/ca
Protein Coding	45	GC19M004174	2.92	https://www.genecards.org/cgi-bin/ca
Protein Coding	48	GC02P046293	2.92	https://www.genecards.org/cgi-bin/ca
Protein Coding	45	GC10P133520	2.91	https://www.genecards.org/cgi-bin/ca
Protein Coding	43	GC04M122612	2.91	https://www.genecards.org/cgi-bin/ca
Protein Coding	44	GC15M060775	2.91	https://www.genecards.org/cgi-bin/ca
Protein Coding	39	GC03P160399	2.9	https://www.genecards.org/cgi-bin/ca
Protein Coding	36	GC01P026317	2.9	https://www.genecards.org/cgi-bin/ca
Protein Coding	47	GC02P074833	2.9	https://www.genecards.org/cgi-bin/ca
Protein Coding	45	GC17M019857	2.9	https://www.genecards.org/cgi-bin/ca
Protein Coding	41	GC06P001610	2.89	https://www.genecards.org/cgi-bin/ca
Protein Coding	47	GC12P057128	2.89	https://www.genecards.org/cgi-bin/ca
Protein Coding	47	GC22M050622	2.89	https://www.genecards.org/cgi-bin/ca
Protein Coding	45	GC06P031945	2.89	https://www.genecards.org/cgi-bin/ca
Protein Coding	47	GC15P099565	2.88	https://www.genecards.org/cgi-bin/ca
Protein Coding	43	GC01P153357	2.88	https://www.genecards.org/cgi-bin/ca
Protein Coding	44	GC12P008124	2.88	https://www.genecards.org/cgi-bin/ca
Protein Coding	35	GC01M047183	2.88	https://www.genecards.org/cgi-bin/ca
Protein Coding	40	GC17P007579	2.87	https://www.genecards.org/cgi-bin/ca
Protein Coding	43	GC08P018391	2.87	https://www.genecards.org/cgi-bin/ca
Protein Coding	49	GC02P127418	2.87	https://www.genecards.org/cgi-bin/ca
Protein Coding	43	GC11P134068	2.87	https://www.genecards.org/cgi-bin/ca
Protein Coding	35	GC02M061792	2.87	https://www.genecards.org/cgi-bin/ca
Protein Coding	46	GC02M074361	2.87	https://www.genecards.org/cgi-bin/ca
Protein Coding	41	GC10M068414	2.87	https://www.genecards.org/cgi-bin/ca
Protein Coding	45	GC12M109553	2.86	https://www.genecards.org/cgi-bin/ca
Protein Coding	44	GC16P082068	2.86	https://www.genecards.org/cgi-bin/ca

Protein Coding	39	GC19P038227	2.86	https://www.genecards.org/cgi-bin/ca
Protein Coding	39	GC20M062464	2.86	https://www.genecards.org/cgi-bin/ca
Protein Coding	41	GC06M028903	2.86	https://www.genecards.org/cgi-bin/ca
Protein Coding	47	GC02M009583	2.85	https://www.genecards.org/cgi-bin/ca
Protein Coding	43	GC01P032651	2.85	https://www.genecards.org/cgi-bin/ca
Protein Coding	47	GC05M040759	2.85	https://www.genecards.org/cgi-bin/ca
Protein Coding	32	GC07M092468	2.84	https://www.genecards.org/cgi-bin/ca
Protein Coding	39	GC17M005366	2.84	https://www.genecards.org/cgi-bin/ca
Protein Coding	39	GC07P144959	2.84	https://www.genecards.org/cgi-bin/ca
Protein Coding	39	GC01M203083	2.84	https://www.genecards.org/cgi-bin/ca
Protein Coding	43	GC0XP048698	2.84	https://www.genecards.org/cgi-bin/ca
Protein Coding	41	GC19P011539	2.83	https://www.genecards.org/cgi-bin/ca
Protein Coding	47	GC02P074529	2.83	https://www.genecards.org/cgi-bin/ca
Protein Coding	49	GC02P172427	2.83	https://www.genecards.org/cgi-bin/ca
Protein Coding	47	GC05M132481	2.83	https://www.genecards.org/cgi-bin/ca
Protein Coding	44	GC17P074749	2.83	https://www.genecards.org/cgi-bin/ca
Protein Coding	44	GC02P000254	2.83	https://www.genecards.org/cgi-bin/ca
Protein Coding	45	GC02M075010	2.83	https://www.genecards.org/cgi-bin/ca
Protein Coding	42	GC04P123396	2.82	https://www.genecards.org/cgi-bin/ca
Protein Coding	46	GC11P102317	2.82	https://www.genecards.org/cgi-bin/ca
Protein Coding	48	GC14P100238	2.82	https://www.genecards.org/cgi-bin/ca
Protein Coding	51	GC12P110280	2.81	https://www.genecards.org/cgi-bin/ca
Protein Coding	44	GC06P050894	2.81	https://www.genecards.org/cgi-bin/ca
Protein Coding	40	GC07M030010	2.81	https://www.genecards.org/cgi-bin/ca
Protein Coding	45	GC19M001609	2.8	https://www.genecards.org/cgi-bin/ca
Protein Coding	42	GC11M005355	2.8	https://www.genecards.org/cgi-bin/ca
Protein Coding	40	GC22P031109	2.8	https://www.genecards.org/cgi-bin/ca
Protein Coding	43	GC17P008002	2.8	https://www.genecards.org/cgi-bin/ca
Protein Coding	37	GC08M053966	2.8	https://www.genecards.org/cgi-bin/ca
Protein Coding	38	GC01M215622	2.8	https://www.genecards.org/cgi-bin/ca
Protein Coding	42	GC01M040300	2.79	https://www.genecards.org/cgi-bin/ca
Protein Coding	37	GC09M133250	2.79	https://www.genecards.org/cgi-bin/ca
Protein Coding	48	GC05M058969	2.79	https://www.genecards.org/cgi-bin/ca
Protein Coding	49	GC11P108121	2.79	https://www.genecards.org/cgi-bin/ca
Protein Coding	46	GC06M088139	2.79	https://www.genecards.org/cgi-bin/ca
Protein Coding	41	GC19M055364	2.79	https://www.genecards.org/cgi-bin/ca
Protein Coding	40	GC01M041478	2.79	https://www.genecards.org/cgi-bin/ca
Protein Coding	48	GC11M068754	2.79	https://www.genecards.org/cgi-bin/ca
Protein Coding	44	GC16M014672	2.78	https://www.genecards.org/cgi-bin/ca
Protein Coding	39	GC08M091954	2.78	https://www.genecards.org/cgi-bin/ca
Protein Coding	41	GC06P032014	2.78	https://www.genecards.org/cgi-bin/ca
Protein Coding	39	GC06M079484	2.78	https://www.genecards.org/cgi-bin/ca
Protein Coding	36	GC11P002302	2.78	https://www.genecards.org/cgi-bin/ca
Protein Coding	51	GC02M009488	2.78	https://www.genecards.org/cgi-bin/ca
Protein Coding	44	GC12M052674	2.77	https://www.genecards.org/cgi-bin/ca
Protein Coding	50	GC0XP043654	2.77	https://www.genecards.org/cgi-bin/ca
Protein Coding	40	GC06P149749	2.77	https://www.genecards.org/cgi-bin/ca
Protein Coding	45	GC12P054280	2.77	https://www.genecards.org/cgi-bin/ca
Protein Coding	39	GC19M042410	2.77	https://www.genecards.org/cgi-bin/ca
Protein Coding	44	GC01M153543	2.77	https://www.genecards.org/cgi-bin/ca
Protein Coding	37	GC10P016436	2.76	https://www.genecards.org/cgi-bin/ca
Protein Coding	39	GC20P043457	2.76	https://www.genecards.org/cgi-bin/ca
Protein Coding	47	GC01P161663	2.76	https://www.genecards.org/cgi-bin/ca
Protein Coding	44	GC05M038939	2.76	https://www.genecards.org/cgi-bin/ca

Protein Coding	46	GC10P123008	2.76	https://www.genecards.org/cgi-bin/ca
Protein Coding	43	GC02M071110	2.76	https://www.genecards.org/cgi-bin/ca
Protein Coding	42	GC17M006393	2.76	https://www.genecards.org/cgi-bin/ca
Protein Coding	39	GC13M077044	2.76	https://www.genecards.org/cgi-bin/ca
Protein Coding	39	GC06M069675	2.76	https://www.genecards.org/cgi-bin/ca
Protein Coding	37	GC01M027970	2.76	https://www.genecards.org/cgi-bin/ca
Protein Coding	37	GC12M089419	2.76	https://www.genecards.org/cgi-bin/ca
Protein Coding	37	GC11M067350	2.76	https://www.genecards.org/cgi-bin/ca
Protein Coding	35	GC13M100530	2.76	https://www.genecards.org/cgi-bin/ca
Protein Coding	35	GC12M123918	2.76	https://www.genecards.org/cgi-bin/ca
Protein Coding	34	GC08P033464	2.76	https://www.genecards.org/cgi-bin/ca
Protein Coding	31	GC12P002876	2.76	https://www.genecards.org/cgi-bin/ca
Protein Coding	31	GC11M004848	2.76	https://www.genecards.org/cgi-bin/ca
Protein Coding	30	GC19P032975	2.76	https://www.genecards.org/cgi-bin/ca
Protein Coding	45	GC11P000895	2.75	https://www.genecards.org/cgi-bin/ca
Protein Coding	45	GC18M031058	2.75	https://www.genecards.org/cgi-bin/ca
Protein Coding	44	GC17P001742	2.75	https://www.genecards.org/cgi-bin/ca
Protein Coding	48	GC17M037084	2.75	https://www.genecards.org/cgi-bin/ca
Protein Coding	41	GC15P033310	2.75	https://www.genecards.org/cgi-bin/ca
Protein Coding	42	GC12M111443	2.75	https://www.genecards.org/cgi-bin/ca
Protein Coding	47	GC09M096236	2.75	https://www.genecards.org/cgi-bin/ca
Protein Coding	47	GC16P023892	2.74	https://www.genecards.org/cgi-bin/ca
Protein Coding	44	GC11M078100	2.74	https://www.genecards.org/cgi-bin/ca
Protein Coding	46	GC15P090868	2.74	https://www.genecards.org/cgi-bin/ca
Protein Coding	47	GC02P208266	2.74	https://www.genecards.org/cgi-bin/ca
Biological Regior	1	GC05P001294	2.74	https://www.genecards.org/cgi-bin/ca
Protein Coding	40	GC02M231921	2.73	https://www.genecards.org/cgi-bin/ca
Protein Coding	40	GC01M116514	2.73	https://www.genecards.org/cgi-bin/ca
Protein Coding	44	GC08M081478	2.73	https://www.genecards.org/cgi-bin/ca
Protein Coding	41	GC08P081282	2.73	https://www.genecards.org/cgi-bin/ca
RNA Gene	17	GC13P091435	2.72	https://www.genecards.org/cgi-bin/ca
Protein Coding	51	GC0XP048801	2.72	https://www.genecards.org/cgi-bin/ca
Protein Coding	41	GC21M042311	2.72	https://www.genecards.org/cgi-bin/ca
Protein Coding	28	GC12M120128	2.72	https://www.genecards.org/cgi-bin/ca
Protein Coding	48	GC04M121879	2.72	https://www.genecards.org/cgi-bin/ca
Protein Coding	46	GC11M102770	2.7	https://www.genecards.org/cgi-bin/ca
Protein Coding	45	GC19P007903	2.7	https://www.genecards.org/cgi-bin/ca
Protein Coding	43	GC02P055231	2.7	https://www.genecards.org/cgi-bin/ca
Protein Coding	47	GC11P004115	2.7	https://www.genecards.org/cgi-bin/ca
Protein Coding	44	GC13M113820	2.69	https://www.genecards.org/cgi-bin/ca
Protein Coding	38	GC19M042441	2.68	https://www.genecards.org/cgi-bin/ca
Protein Coding	50	GC03M024117	2.68	https://www.genecards.org/cgi-bin/ca
Protein Coding	46	GC06P047342	2.67	https://www.genecards.org/cgi-bin/ca
Protein Coding	40	GC01M160608	2.67	https://www.genecards.org/cgi-bin/ca
Protein Coding	47	GC06P047288	2.66	https://www.genecards.org/cgi-bin/ca
RNA Gene	18	GC14P104778	2.65	https://www.genecards.org/cgi-bin/ca
Protein Coding	39	GC10M072367	2.65	https://www.genecards.org/cgi-bin/ca
Protein Coding	41	GC02P169584	2.65	https://www.genecards.org/cgi-bin/ca
Protein Coding	41	GC12P069471	2.65	https://www.genecards.org/cgi-bin/ca
Protein Coding	43	GC16M011255	2.65	https://www.genecards.org/cgi-bin/ca
RNA Gene	17	GC18M021828	2.65	https://www.genecards.org/cgi-bin/ca
Protein Coding	43	GC01P150459	2.65	https://www.genecards.org/cgi-bin/ca
Protein Coding	37	GC12M055954	2.64	https://www.genecards.org/cgi-bin/ca
Protein Coding	40	GC05P000892	2.63	https://www.genecards.org/cgi-bin/ca

Protein Coding	44	GC01M202940	2.63	https://www.genecards.org/cgi-bin/ca
Protein Coding	44	GC01M025884	2.63	https://www.genecards.org/cgi-bin/ca
Protein Coding	47	GC04M110617	2.63	https://www.genecards.org/cgi-bin/ca
Protein Coding	40	GC11M073676	2.63	https://www.genecards.org/cgi-bin/ca
Protein Coding	28	GC05M037065	2.63	https://www.genecards.org/cgi-bin/ca
Protein Coding	41	GC20M050888	2.62	https://www.genecards.org/cgi-bin/ca
Protein Coding	43	GC01P154273	2.62	https://www.genecards.org/cgi-bin/ca
Protein Coding	43	GC04M099304	2.61	https://www.genecards.org/cgi-bin/ca
Protein Coding	37	GC11P072114	2.61	https://www.genecards.org/cgi-bin/ca
Protein Coding	43	GC17M008854	2.61	https://www.genecards.org/cgi-bin/ca
Protein Coding	52	GC13M028003	2.61	https://www.genecards.org/cgi-bin/ca
Protein Coding	43	GC11P046402	2.6	https://www.genecards.org/cgi-bin/ca
Protein Coding	44	GC19P015049	2.6	https://www.genecards.org/cgi-bin/ca
Protein Coding	41	GC17M081911	2.6	https://www.genecards.org/cgi-bin/ca
Protein Coding	38	GC06P125730	2.6	https://www.genecards.org/cgi-bin/ca
Protein Coding	42	GC17M044852	2.6	https://www.genecards.org/cgi-bin/ca
Protein Coding	47	GC03P134598	2.6	https://www.genecards.org/cgi-bin/ca
RNA Gene	15	GC14P104799	2.6	https://www.genecards.org/cgi-bin/ca
Protein Coding	43	GC16P088803	2.59	https://www.genecards.org/cgi-bin/ca
Protein Coding	45	GC02P137964	2.59	https://www.genecards.org/cgi-bin/ca
Protein Coding	38	GC16M000084	2.58	https://www.genecards.org/cgi-bin/ca
Protein Coding	35	GC16M028548	2.58	https://www.genecards.org/cgi-bin/ca
Protein Coding	43	GC01M109310	2.58	https://www.genecards.org/cgi-bin/ca
Protein Coding	45	GC15M050552	2.58	https://www.genecards.org/cgi-bin/ca
Protein Coding	48	GC14M077505	2.58	https://www.genecards.org/cgi-bin/ca
Protein Coding	36	GC01M023382	2.57	https://www.genecards.org/cgi-bin/ca
Protein Coding	39	GC02M079157	2.57	https://www.genecards.org/cgi-bin/ca
Protein Coding	48	GC10P100347	2.56	https://www.genecards.org/cgi-bin/ca
Protein Coding	44	GC18M012328	2.56	https://www.genecards.org/cgi-bin/ca
Protein Coding	46	GC09P012683	2.56	https://www.genecards.org/cgi-bin/ca
Protein Coding	45	GC06P013574	2.56	https://www.genecards.org/cgi-bin/ca
Protein Coding	41	GC19P038899	2.55	https://www.genecards.org/cgi-bin/ca
Protein Coding	46	GC17P051165	2.55	https://www.genecards.org/cgi-bin/ca
RNA Gene	18	GC10M086264	2.54	https://www.genecards.org/cgi-bin/ca
Protein Coding	48	GC19P049675	2.54	https://www.genecards.org/cgi-bin/ca
Protein Coding	48	GC14M065009	2.53	https://www.genecards.org/cgi-bin/ca
Protein Coding	38	GC09M137447	2.53	https://www.genecards.org/cgi-bin/ca
Protein Coding	39	GC03M015815	2.53	https://www.genecards.org/cgi-bin/ca
Protein Coding	39	GC07P044106	2.53	https://www.genecards.org/cgi-bin/ca
Protein Coding	40	GC12P021354	2.53	https://www.genecards.org/cgi-bin/ca
Protein Coding	48	GC02P010123	2.52	https://www.genecards.org/cgi-bin/ca
Protein Coding	41	GC22M019523	2.52	https://www.genecards.org/cgi-bin/ca
Protein Coding	37	GC07M098294	2.52	https://www.genecards.org/cgi-bin/ca
Protein Coding	47	GC01M021217	2.52	https://www.genecards.org/cgi-bin/ca
Protein Coding	45	GC12P131894	2.52	https://www.genecards.org/cgi-bin/ca
Protein Coding	44	GC03M185643	2.52	https://www.genecards.org/cgi-bin/ca
Protein Coding	43	GC02M210187	2.52	https://www.genecards.org/cgi-bin/ca
Protein Coding	41	GC07P036389	2.52	https://www.genecards.org/cgi-bin/ca
Protein Coding	38	GC08M056436	2.52	https://www.genecards.org/cgi-bin/ca
Protein Coding	48	GC20P044885	2.51	https://www.genecards.org/cgi-bin/ca
Protein Coding	31	GC01M149950	2.51	https://www.genecards.org/cgi-bin/ca
Protein Coding	39	GC05M032124	2.51	https://www.genecards.org/cgi-bin/ca
Protein Coding	48	GC08M100917	2.51	https://www.genecards.org/cgi-bin/ca
Protein Coding	41	GC0XP077447	2.51	https://www.genecards.org/cgi-bin/ca

Protein Coding	47	GC01P001001	2.51	https://www.genecards.org/cgi-bin/ca
Protein Coding	45	GC12M014612	2.5	https://www.genecards.org/cgi-bin/ca
Protein Coding	39	GC02P183078	2.5	https://www.genecards.org/cgi-bin/ca
Protein Coding	44	GC03P186612	2.5	https://www.genecards.org/cgi-bin/ca
Protein Coding	44	GC14P025379	2.49	https://www.genecards.org/cgi-bin/ca
Protein Coding	38	GC16M071463	2.49	https://www.genecards.org/cgi-bin/ca
RNA Gene	22	GC07P027198	2.49	https://www.genecards.org/cgi-bin/ca
Protein Coding	36	GC07M073736	2.49	https://www.genecards.org/cgi-bin/ca
Protein Coding	41	GC07M131500	2.49	https://www.genecards.org/cgi-bin/ca
Protein Coding	43	GC16M028606	2.48	https://www.genecards.org/cgi-bin/ca
Protein Coding	43	GC01M225937	2.48	https://www.genecards.org/cgi-bin/ca
Protein Coding	38	GC17P081704	2.48	https://www.genecards.org/cgi-bin/ca
Protein Coding	43	GC12P093569	2.48	https://www.genecards.org/cgi-bin/ca
Protein Coding	46	GC11P057597	2.48	https://www.genecards.org/cgi-bin/ca
Protein Coding	42	GC02P218270	2.48	https://www.genecards.org/cgi-bin/ca
Protein Coding	40	GC06P047289	2.47	https://www.genecards.org/cgi-bin/ca
Protein Coding	44	GC10P072273	2.47	https://www.genecards.org/cgi-bin/ca
Protein Coding	41	GC17M058000	2.47	https://www.genecards.org/cgi-bin/ca
Protein Coding	46	GC17M003565	2.47	https://www.genecards.org/cgi-bin/ca
Protein Coding	51	GC19M014092	2.47	https://www.genecards.org/cgi-bin/ca
Protein Coding	41	GC03P184380	2.47	https://www.genecards.org/cgi-bin/ca
Protein Coding	36	GC01P110451	2.47	https://www.genecards.org/cgi-bin/ca
Protein Coding	42	GC14M105140	2.46	https://www.genecards.org/cgi-bin/ca
Protein Coding	44	GC02P024492	2.46	https://www.genecards.org/cgi-bin/ca
Protein Coding	38	GC11M005268	2.45	https://www.genecards.org/cgi-bin/ca
Protein Coding	45	GC11P113974	2.45	https://www.genecards.org/cgi-bin/ca
Protein Coding	28	GC19M055308	2.45	https://www.genecards.org/cgi-bin/ca
Protein Coding	36	GC04P109815	2.45	https://www.genecards.org/cgi-bin/ca
Protein Coding	40	GC03M012153	2.45	https://www.genecards.org/cgi-bin/ca
Protein Coding	39	GC03P045730	2.45	https://www.genecards.org/cgi-bin/ca
Protein Coding	45	GC14P020830	2.45	https://www.genecards.org/cgi-bin/ca
RNA Gene	19	GC17M002050	2.44	https://www.genecards.org/cgi-bin/ca
Protein Coding	47	GC07M099648	2.44	https://www.genecards.org/cgi-bin/ca
Protein Coding	47	GC03M190305	2.44	https://www.genecards.org/cgi-bin/ca
Protein Coding	43	GC12P001670	2.44	https://www.genecards.org/cgi-bin/ca
Protein Coding	43	GC03M195568	2.44	https://www.genecards.org/cgi-bin/ca
Protein Coding	37	GC07P143132	2.44	https://www.genecards.org/cgi-bin/ca
Protein Coding	47	GC07M107923	2.44	https://www.genecards.org/cgi-bin/ca
Protein Coding	44	GC07M092112	2.43	https://www.genecards.org/cgi-bin/ca
Protein Coding	45	GC10M070882	2.43	https://www.genecards.org/cgi-bin/ca
Protein Coding	45	GC01P199996	2.43	https://www.genecards.org/cgi-bin/ca
Protein Coding	42	GC04P087650	2.43	https://www.genecards.org/cgi-bin/ca
Protein Coding	36	GC0XP152698	2.43	https://www.genecards.org/cgi-bin/ca
Protein Coding	47	GC16P027325	2.43	https://www.genecards.org/cgi-bin/ca
Protein Coding	36	GC12M006556	2.42	https://www.genecards.org/cgi-bin/ca
Protein Coding	45	GC01M151955	2.42	https://www.genecards.org/cgi-bin/ca
Protein Coding	43	GC12M124911	2.42	https://www.genecards.org/cgi-bin/ca
Protein Coding	38	GC22M030576	2.42	https://www.genecards.org/cgi-bin/ca
Protein Coding	46	GC22M019176	2.42	https://www.genecards.org/cgi-bin/ca
Protein Coding	44	GC0XM071615	2.42	https://www.genecards.org/cgi-bin/ca
Protein Coding	43	GC12P050057	2.42	https://www.genecards.org/cgi-bin/ca
Protein Coding	49	GC09P134317	2.41	https://www.genecards.org/cgi-bin/ca
Protein Coding	40	GC02P222860	2.41	https://www.genecards.org/cgi-bin/ca
Protein Coding	45	GC07P091264	2.41	https://www.genecards.org/cgi-bin/ca

Protein Coding	47	GC07M143390	2.4	https://www.genecards.org/cgi-bin/ca
Protein Coding	40	GC04P024798	2.4	https://www.genecards.org/cgi-bin/ca
Protein Coding	37	GC02P069897	2.4	https://www.genecards.org/cgi-bin/ca
Protein Coding	42	GC10M131966	2.4	https://www.genecards.org/cgi-bin/ca
Protein Coding	50	GC06P145973	2.4	https://www.genecards.org/cgi-bin/ca
Protein Coding	49	GC20P010218	2.4	https://www.genecards.org/cgi-bin/ca
Protein Coding	43	GC0XM078271	2.38	https://www.genecards.org/cgi-bin/ca
Protein Coding	46	GC02M224470	2.38	https://www.genecards.org/cgi-bin/ca
Protein Coding	39	GC08P011769	2.38	https://www.genecards.org/cgi-bin/ca
Protein Coding	41	GC12P080707	2.38	https://www.genecards.org/cgi-bin/ca
Protein Coding	43	GC07P097731	2.37	https://www.genecards.org/cgi-bin/ca
Protein Coding	47	GC06P047433	2.37	https://www.genecards.org/cgi-bin/ca
Protein Coding	44	GC20P038346	2.36	https://www.genecards.org/cgi-bin/ca
Protein Coding	49	GC01M045511	2.36	https://www.genecards.org/cgi-bin/ca
Protein Coding	41	GC08M063048	2.36	https://www.genecards.org/cgi-bin/ca
Protein Coding	44	GC19P010416	2.36	https://www.genecards.org/cgi-bin/ca
Protein Coding	44	GC05P138465	2.36	https://www.genecards.org/cgi-bin/ca
Protein Coding	46	GC11P047567	2.35	https://www.genecards.org/cgi-bin/ca
Protein Coding	42	GC08P073991	2.35	https://www.genecards.org/cgi-bin/ca
Protein Coding	41	GC01M206928	2.35	https://www.genecards.org/cgi-bin/ca
Protein Coding	43	GC01M026922	2.35	https://www.genecards.org/cgi-bin/ca
Protein Coding	48	GC11M035272	2.34	https://www.genecards.org/cgi-bin/ca
Protein Coding	42	GC09P133348	2.34	https://www.genecards.org/cgi-bin/ca
Protein Coding	50	GC09P128191	2.34	https://www.genecards.org/cgi-bin/ca
Protein Coding	43	GC16M028466	2.34	https://www.genecards.org/cgi-bin/ca
Protein Coding	29	GC01M149963	2.34	https://www.genecards.org/cgi-bin/ca
Protein Coding	46	GC01P026873	2.33	https://www.genecards.org/cgi-bin/ca
Protein Coding	37	GC01P159203	2.33	https://www.genecards.org/cgi-bin/ca
Protein Coding	45	GC06M006144	2.33	https://www.genecards.org/cgi-bin/ca
Protein Coding	41	GC06P047281	2.33	https://www.genecards.org/cgi-bin/ca
Protein Coding	44	GC07M044512	2.33	https://www.genecards.org/cgi-bin/ca
Protein Coding	40	GC20M064047	2.32	https://www.genecards.org/cgi-bin/ca
Protein Coding	39	GC01P235335	2.32	https://www.genecards.org/cgi-bin/ca
Protein Coding	44	GC02M219289	2.32	https://www.genecards.org/cgi-bin/ca
Protein Coding	38	GC22M031325	2.3	https://www.genecards.org/cgi-bin/ca
Protein Coding	51	GC01P022710	2.3	https://www.genecards.org/cgi-bin/ca
Protein Coding	47	GC12P006839	2.3	https://www.genecards.org/cgi-bin/ca
Protein Coding	43	GC10M073248	2.3	https://www.genecards.org/cgi-bin/ca
Protein Coding	43	GC11M078435	2.3	https://www.genecards.org/cgi-bin/ca
Protein Coding	41	GC15M084655	2.3	https://www.genecards.org/cgi-bin/ca
Protein Coding	45	GC06M042239	2.3	https://www.genecards.org/cgi-bin/ca
Protein Coding	37	GC0XP054920	2.3	https://www.genecards.org/cgi-bin/ca
Protein Coding	37	GC08P007895	2.3	https://www.genecards.org/cgi-bin/ca
Protein Coding	44	GC01M027902	2.29	https://www.genecards.org/cgi-bin/ca
Protein Coding	40	GC19P049506	2.29	https://www.genecards.org/cgi-bin/ca
Protein Coding	44	GC01P043358	2.29	https://www.genecards.org/cgi-bin/ca
Protein Coding	43	GC03P013565	2.29	https://www.genecards.org/cgi-bin/ca
Protein Coding	47	GC22M046185	2.29	https://www.genecards.org/cgi-bin/ca
Protein Coding	44	GC13M046053	2.29	https://www.genecards.org/cgi-bin/ca
Protein Coding	46	GC11P064317	2.28	https://www.genecards.org/cgi-bin/ca
Protein Coding	40	GC12P080716	2.27	https://www.genecards.org/cgi-bin/ca
Protein Coding	40	GC13P072782	2.27	https://www.genecards.org/cgi-bin/ca
Protein Coding	44	GC08M023296	2.27	https://www.genecards.org/cgi-bin/ca
RNA Gene	21	GC21P016590	2.27	https://www.genecards.org/cgi-bin/ca

Protein Coding	41	GC01P150926	2.26	https://www.genecards.org/cgi-bin/ca
Protein Coding	48	GC03M165772	2.26	https://www.genecards.org/cgi-bin/ca
Protein Coding	48	GC12P071938	2.26	https://www.genecards.org/cgi-bin/ca
Protein Coding	39	GC02M217799	2.26	https://www.genecards.org/cgi-bin/ca
Protein Coding	49	GC01P186798	2.26	https://www.genecards.org/cgi-bin/ca
Protein Coding	45	GC04M057030	2.26	https://www.genecards.org/cgi-bin/ca
Protein Coding	40	GC17P039461	2.26	https://www.genecards.org/cgi-bin/ca
Protein Coding	41	GC17M001650	2.26	https://www.genecards.org/cgi-bin/ca
Protein Coding	43	GC14M095709	2.25	https://www.genecards.org/cgi-bin/ca
Protein Coding	44	GC06M049605	2.25	https://www.genecards.org/cgi-bin/ca
Protein Coding	47	GC10P005035	2.25	https://www.genecards.org/cgi-bin/ca
Protein Coding	39	GC01M220149	2.25	https://www.genecards.org/cgi-bin/ca
Protein Coding	33	GC19P012737	2.25	https://www.genecards.org/cgi-bin/ca
Protein Coding	43	GC01P162790	2.24	https://www.genecards.org/cgi-bin/ca
Protein Coding	40	GC01P220879	2.24	https://www.genecards.org/cgi-bin/ca
Protein Coding	40	GC08P119873	2.24	https://www.genecards.org/cgi-bin/ca
Protein Coding	48	GC05M177402	2.24	https://www.genecards.org/cgi-bin/ca
Protein Coding	44	GC02P008772	2.23	https://www.genecards.org/cgi-bin/ca
Protein Coding	31	GC07P071133	2.23	https://www.genecards.org/cgi-bin/ca
Protein Coding	44	GC01P193121	2.23	https://www.genecards.org/cgi-bin/ca
Protein Coding	45	GC02M175072	2.23	https://www.genecards.org/cgi-bin/ca
Protein Coding	41	GC01M054756	2.23	https://www.genecards.org/cgi-bin/ca
Protein Coding	43	GC19M006583	2.23	https://www.genecards.org/cgi-bin/ca
Protein Coding	43	GC22P043151	2.23	https://www.genecards.org/cgi-bin/ca
Protein Coding	45	GC01P147902	2.23	https://www.genecards.org/cgi-bin/ca
Protein Coding	44	GC0XP040582	2.23	https://www.genecards.org/cgi-bin/ca
Protein Coding	50	GC09P091171	2.22	https://www.genecards.org/cgi-bin/ca
Protein Coding	43	GC11P018343	2.22	https://www.genecards.org/cgi-bin/ca
Protein Coding	41	GC10P100463	2.22	https://www.genecards.org/cgi-bin/ca
Protein Coding	46	GC04P071063	2.22	https://www.genecards.org/cgi-bin/ca
Protein Coding	44	GC09M081583	2.22	https://www.genecards.org/cgi-bin/ca
Protein Coding	46	GC0XP047190	2.22	https://www.genecards.org/cgi-bin/ca
Protein Coding	37	GC17M082614	2.22	https://www.genecards.org/cgi-bin/ca
Protein Coding	32	GC07M073312	2.22	https://www.genecards.org/cgi-bin/ca
Protein Coding	42	GC16P056627	2.22	https://www.genecards.org/cgi-bin/ca
Protein Coding	45	GC01P151372	2.22	https://www.genecards.org/cgi-bin/ca
Protein Coding	42	GC17P035578	2.22	https://www.genecards.org/cgi-bin/ca
Protein Coding	44	GC04M069841	2.22	https://www.genecards.org/cgi-bin/ca
Protein Coding	47	GC04M079901	2.22	https://www.genecards.org/cgi-bin/ca
Protein Coding	45	GC01P183155	2.21	https://www.genecards.org/cgi-bin/ca
Protein Coding	42	GC12M049127	2.21	https://www.genecards.org/cgi-bin/ca
Protein Coding	43	GC22M030262	2.21	https://www.genecards.org/cgi-bin/ca
Protein Coding	44	GC05P071719	2.21	https://www.genecards.org/cgi-bin/ca
Protein Coding	40	GC01M035720	2.2	https://www.genecards.org/cgi-bin/ca
Protein Coding	42	GC10M027110	2.2	https://www.genecards.org/cgi-bin/ca
Protein Coding	44	GC03M071771	2.2	https://www.genecards.org/cgi-bin/ca
Protein Coding	43	GC08M041929	2.19	https://www.genecards.org/cgi-bin/ca
Protein Coding	42	GC01P115029	2.19	https://www.genecards.org/cgi-bin/ca
Protein Coding	46	GC08M027459	2.19	https://www.genecards.org/cgi-bin/ca
Protein Coding	44	GC01P154568	2.19	https://www.genecards.org/cgi-bin/ca
Protein Coding	43	GC16M031201	2.19	https://www.genecards.org/cgi-bin/ca
Protein Coding	44	GC03M050618	2.19	https://www.genecards.org/cgi-bin/ca
Protein Coding	43	GC17P047649	2.19	https://www.genecards.org/cgi-bin/ca
Protein Coding	42	GC15P026971	2.19	https://www.genecards.org/cgi-bin/ca

Protein Coding	51	GC02M239048	2.19	https://www.genecards.org/cgi-bin/ca
Protein Coding	43	GC21M014961	2.18	https://www.genecards.org/cgi-bin/ca
Protein Coding	50	GC03P038453	2.18	https://www.genecards.org/cgi-bin/ca
Protein Coding	41	GC07M123681	2.18	https://www.genecards.org/cgi-bin/ca
Protein Coding	40	GC07P094656	2.18	https://www.genecards.org/cgi-bin/ca
Protein Coding	46	GC12P049053	2.18	https://www.genecards.org/cgi-bin/ca
Protein Coding	47	GC14M022773	2.18	https://www.genecards.org/cgi-bin/ca
Protein Coding	50	GC02P045651	2.17	https://www.genecards.org/cgi-bin/ca
Protein Coding	43	GC02M060451	2.17	https://www.genecards.org/cgi-bin/ca
Protein Coding	42	GC0XP051803	2.17	https://www.genecards.org/cgi-bin/ca
Protein Coding	45	GC06M169215	2.17	https://www.genecards.org/cgi-bin/ca
Protein Coding	44	GC01P010210	2.16	https://www.genecards.org/cgi-bin/ca
Protein Coding	37	GC09M005306	2.16	https://www.genecards.org/cgi-bin/ca
Protein Coding	44	GC02M187464	2.16	https://www.genecards.org/cgi-bin/ca
Protein Coding	44	GC05P139526	2.16	https://www.genecards.org/cgi-bin/ca
Protein Coding	43	GC19P011435	2.16	https://www.genecards.org/cgi-bin/ca
Protein Coding	40	GC06M007268	2.16	https://www.genecards.org/cgi-bin/ca
Protein Coding	40	GC07P150824	2.15	https://www.genecards.org/cgi-bin/ca
Protein Coding	41	GC15P078340	2.15	https://www.genecards.org/cgi-bin/ca
Protein Coding	41	GC03M047850	2.15	https://www.genecards.org/cgi-bin/ca
Protein Coding	40	GC04P121801	2.15	https://www.genecards.org/cgi-bin/ca
Protein Coding	35	GC18P033578	2.15	https://www.genecards.org/cgi-bin/ca
Protein Coding	36	GC04M069955	2.15	https://www.genecards.org/cgi-bin/ca
Protein Coding	41	GC01P153991	2.15	https://www.genecards.org/cgi-bin/ca
Protein Coding	50	GC11M061959	2.14	https://www.genecards.org/cgi-bin/ca
Protein Coding	40	GC20P003827	2.14	https://www.genecards.org/cgi-bin/ca
Protein Coding	41	GC06P116255	2.14	https://www.genecards.org/cgi-bin/ca
Protein Coding	42	GC10P096304	2.14	https://www.genecards.org/cgi-bin/ca
Protein Coding	40	GC02M127638	2.14	https://www.genecards.org/cgi-bin/ca
Protein Coding	47	GC11P112226	2.13	https://www.genecards.org/cgi-bin/ca
Protein Coding	39	GC02P218259	2.13	https://www.genecards.org/cgi-bin/ca
Protein Coding	52	GC15M058588	2.13	https://www.genecards.org/cgi-bin/ca
Protein Coding	41	GC09M034692	2.13	https://www.genecards.org/cgi-bin/ca
Protein Coding	45	GC21M045493	2.13	https://www.genecards.org/cgi-bin/ca
Protein Coding	39	GC06P014117	2.13	https://www.genecards.org/cgi-bin/ca
Protein Coding	45	GC17M041866	2.13	https://www.genecards.org/cgi-bin/ca
Protein Coding	36	GC11M130868	2.13	https://www.genecards.org/cgi-bin/ca
Protein Coding	41	GC08P038104	2.13	https://www.genecards.org/cgi-bin/ca
Protein Coding	37	GC02M031867	2.13	https://www.genecards.org/cgi-bin/ca
Protein Coding	39	GC01M039623	2.13	https://www.genecards.org/cgi-bin/ca
Protein Coding	40	GC01P228208	2.12	https://www.genecards.org/cgi-bin/ca
Protein Coding	39	GC09M108942	2.12	https://www.genecards.org/cgi-bin/ca
Protein Coding	41	GC03P052455	2.12	https://www.genecards.org/cgi-bin/ca
Protein Coding	32	GC11M003000	2.12	https://www.genecards.org/cgi-bin/ca
Protein Coding	25	GC01P186376	2.12	https://www.genecards.org/cgi-bin/ca
Protein Coding	39	GC03P112332	2.11	https://www.genecards.org/cgi-bin/ca
Protein Coding	42	GC12P053097	2.11	https://www.genecards.org/cgi-bin/ca
Protein Coding	43	GC0XM084058	2.11	https://www.genecards.org/cgi-bin/ca
RNA Gene	21	GC09P095097	2.11	https://www.genecards.org/cgi-bin/ca
Protein Coding	41	GC03M129167	2.11	https://www.genecards.org/cgi-bin/ca
Protein Coding	31	GCMTP010762	2.11	https://www.genecards.org/cgi-bin/ca
Protein Coding	44	GC06M111299	2.11	https://www.genecards.org/cgi-bin/ca
Protein Coding	44	GC02M181673	2.1	https://www.genecards.org/cgi-bin/ca
Protein Coding	48	GC03M049368	2.1	https://www.genecards.org/cgi-bin/ca

Protein Coding	47	GC19P040991	2.1	https://www.genecards.org/cgi-bin/ca
Protein Coding	48	GC0XM015562	2.1	https://www.genecards.org/cgi-bin/ca
Protein Coding	45	GC11M073974	2.1	https://www.genecards.org/cgi-bin/ca
Protein Coding	39	GC11P022626	2.1	https://www.genecards.org/cgi-bin/ca
Protein Coding	43	GC06M107867	2.1	https://www.genecards.org/cgi-bin/ca
Protein Coding	19	GC04P164877	2.09	https://www.genecards.org/cgi-bin/ca
Protein Coding	41	GC01M023557	2.09	https://www.genecards.org/cgi-bin/ca
Protein Coding	31	GC05P143061	2.09	https://www.genecards.org/cgi-bin/ca
Protein Coding	39	GC11M018234	2.08	https://www.genecards.org/cgi-bin/ca
Protein Coding	41	GC01M007843	2.08	https://www.genecards.org/cgi-bin/ca
RNA Gene	14	GC05P176367	2.08	https://www.genecards.org/cgi-bin/ca
Protein Coding	44	GC17P043993	2.08	https://www.genecards.org/cgi-bin/ca
Protein Coding	35	GC13P032586	2.08	https://www.genecards.org/cgi-bin/ca
Protein Coding	44	GC0XP071534	2.07	https://www.genecards.org/cgi-bin/ca
Protein Coding	45	GC12P049961	2.07	https://www.genecards.org/cgi-bin/ca
Protein Coding	44	GC11M014505	2.07	https://www.genecards.org/cgi-bin/ca
Protein Coding	43	GC06P047290	2.07	https://www.genecards.org/cgi-bin/ca
Protein Coding	43	GC02P177392	2.06	https://www.genecards.org/cgi-bin/ca
Protein Coding	42	GC02P173354	2.06	https://www.genecards.org/cgi-bin/ca
Protein Coding	49	GC22P032043	2.06	https://www.genecards.org/cgi-bin/ca
Protein Coding	45	GC20M022581	2.06	https://www.genecards.org/cgi-bin/ca
Protein Coding	44	GC06P005261	2.06	https://www.genecards.org/cgi-bin/ca
Protein Coding	39	GC15M065148	2.06	https://www.genecards.org/cgi-bin/ca
Protein Coding	35	GC03M150926	2.06	https://www.genecards.org/cgi-bin/ca
Protein Coding	43	GC02M031522	2.06	https://www.genecards.org/cgi-bin/ca
Protein Coding	44	GC11P119084	2.06	https://www.genecards.org/cgi-bin/ca
Protein Coding	45	GC02M190055	2.06	https://www.genecards.org/cgi-bin/ca
Protein Coding	32	GC07M096483	2.06	https://www.genecards.org/cgi-bin/ca
Protein Coding	48	GC15P090883	2.05	https://www.genecards.org/cgi-bin/ca
Protein Coding	39	GC06P020534	2.05	https://www.genecards.org/cgi-bin/ca
Protein Coding	41	GC01M016974	2.05	https://www.genecards.org/cgi-bin/ca
Protein Coding	37	GC20M003191	2.05	https://www.genecards.org/cgi-bin/ca
Protein Coding	50	GC20M034276	2.05	https://www.genecards.org/cgi-bin/ca
Protein Coding	40	GC06M143494	2.04	https://www.genecards.org/cgi-bin/ca
Protein Coding	43	GC18M079970	2.04	https://www.genecards.org/cgi-bin/ca
Protein Coding	43	GC07M127610	2.04	https://www.genecards.org/cgi-bin/ca
Protein Coding	40	GC14M050237	2.04	https://www.genecards.org/cgi-bin/ca
RNA Gene	14	GC20M036054	2.04	https://www.genecards.org/cgi-bin/ca
Protein Coding	41	GC05M176905	2.03	https://www.genecards.org/cgi-bin/ca
Protein Coding	36	GC19M032878	2.03	https://www.genecards.org/cgi-bin/ca
Protein Coding	36	GC17P036064	2.03	https://www.genecards.org/cgi-bin/ca
Protein Coding	44	GC01P028812	2.03	https://www.genecards.org/cgi-bin/ca
Protein Coding	32	GCMTP007587	2.03	https://www.genecards.org/cgi-bin/ca
Protein Coding	45	GC04P154564	2.03	https://www.genecards.org/cgi-bin/ca
Protein Coding	44	GC12P020810	2.03	https://www.genecards.org/cgi-bin/ca
Protein Coding	41	GC01P154974	2.03	https://www.genecards.org/cgi-bin/ca
Protein Coding	45	GC19M007689	2.03	https://www.genecards.org/cgi-bin/ca
Protein Coding	45	GC22P023768	2.03	https://www.genecards.org/cgi-bin/ca
Protein Coding	46	GC08M016107	2.02	https://www.genecards.org/cgi-bin/ca
Protein Coding	43	GC10P122461	2.02	https://www.genecards.org/cgi-bin/ca
Protein Coding	51	GC02P097696	2.02	https://www.genecards.org/cgi-bin/ca
Protein Coding	42	GC01P213983	2.02	https://www.genecards.org/cgi-bin/ca
Protein Coding	44	GC17M043753	2.01	https://www.genecards.org/cgi-bin/ca
Protein Coding	35	GC18P057436	2.01	https://www.genecards.org/cgi-bin/ca

Protein Coding	45	GC01M155897	2.01	https://www.genecards.org/cgi-bin/ca
Protein Coding	36	GC19M008065	2.01	https://www.genecards.org/cgi-bin/ca
Protein Coding	30	GC19M049054	2.01	https://www.genecards.org/cgi-bin/ca
Protein Coding	43	GC06P110814	2.01	https://www.genecards.org/cgi-bin/ca
Protein Coding	45	GC18P000158	2.01	https://www.genecards.org/cgi-bin/ca
Protein Coding	41	GC13M094436	2.01	https://www.genecards.org/cgi-bin/ca
Protein Coding	41	GC07P026291	2.01	https://www.genecards.org/cgi-bin/ca
Protein Coding	42	GC07P134744	2.01	https://www.genecards.org/cgi-bin/ca
Protein Coding	43	GC10M112148	2.01	https://www.genecards.org/cgi-bin/ca
Protein Coding	31	GC0XM133216	2	https://www.genecards.org/cgi-bin/ca
Protein Coding	44	GC0XM047635	2	https://www.genecards.org/cgi-bin/ca
Protein Coding	35	GC06P096521	1.99	https://www.genecards.org/cgi-bin/ca
Protein Coding	48	GC07P076048	1.99	https://www.genecards.org/cgi-bin/ca
Protein Coding	44	GC19M049073	1.99	https://www.genecards.org/cgi-bin/ca
Protein Coding	36	GC04M083407	1.99	https://www.genecards.org/cgi-bin/ca
Protein Coding	44	GC01P212565	1.98	https://www.genecards.org/cgi-bin/ca
Protein Coding	37	GC22M042160	1.98	https://www.genecards.org/cgi-bin/ca
Protein Coding	44	GC06M149658	1.97	https://www.genecards.org/cgi-bin/ca
Protein Coding	45	GC17P045784	1.97	https://www.genecards.org/cgi-bin/ca
Protein Coding	48	GC15M026543	1.97	https://www.genecards.org/cgi-bin/ca
Protein Coding	35	GC04P087821	1.97	https://www.genecards.org/cgi-bin/ca
Protein Coding	41	GC20M036996	1.97	https://www.genecards.org/cgi-bin/ca
Protein Coding	43	GC06P131617	1.97	https://www.genecards.org/cgi-bin/ca
Protein Coding	40	GC0XP002691	1.97	https://www.genecards.org/cgi-bin/ca
Protein Coding	37	GC0XM007898	1.96	https://www.genecards.org/cgi-bin/ca
Protein Coding	40	GC11P067318	1.96	https://www.genecards.org/cgi-bin/ca
Protein Coding	38	GC19P036054	1.96	https://www.genecards.org/cgi-bin/ca
Protein Coding	41	GC08M100685	1.96	https://www.genecards.org/cgi-bin/ca
Protein Coding	40	GC17P030969	1.96	https://www.genecards.org/cgi-bin/ca
Protein Coding	44	GC01P116754	1.96	https://www.genecards.org/cgi-bin/ca
Pseudogene	6	GC08M100776	1.95	https://www.genecards.org/cgi-bin/ca
Protein Coding	44	GC02P069644	1.95	https://www.genecards.org/cgi-bin/ca
Protein Coding	39	GC0XP151912	1.94	https://www.genecards.org/cgi-bin/ca
RNA Gene	19	GC14P104582	1.94	https://www.genecards.org/cgi-bin/ca
Protein Coding	42	GC18M063655	1.94	https://www.genecards.org/cgi-bin/ca
Protein Coding	45	GC17P047733	1.94	https://www.genecards.org/cgi-bin/ca
Protein Coding	40	GC19P044914	1.94	https://www.genecards.org/cgi-bin/ca
Protein Coding	47	GC11P047248	1.94	https://www.genecards.org/cgi-bin/ca
Protein Coding	46	GC12M120322	1.93	https://www.genecards.org/cgi-bin/ca
Protein Coding	43	GC08P009555	1.93	https://www.genecards.org/cgi-bin/ca
Protein Coding	42	GC08M102252	1.93	https://www.genecards.org/cgi-bin/ca
RNA Gene	24	GC11P002140	1.93	https://www.genecards.org/cgi-bin/ca
Protein Coding	48	GC12M048965	1.93	https://www.genecards.org/cgi-bin/ca
Protein Coding	45	GC09P136982	1.93	https://www.genecards.org/cgi-bin/ca
Protein Coding	42	GC22P020859	1.93	https://www.genecards.org/cgi-bin/ca
Protein Coding	44	GC14P049895	1.92	https://www.genecards.org/cgi-bin/ca
Protein Coding	36	GC17P036486	1.92	https://www.genecards.org/cgi-bin/ca
Protein Coding	44	GC11P000896	1.92	https://www.genecards.org/cgi-bin/ca
Protein Coding	43	GC07P151057	1.92	https://www.genecards.org/cgi-bin/ca
Protein Coding	50	GC08P055879	1.92	https://www.genecards.org/cgi-bin/ca
Protein Coding	48	GC0XP154541	1.92	https://www.genecards.org/cgi-bin/ca
Protein Coding	48	GC01P206684	1.91	https://www.genecards.org/cgi-bin/ca
Protein Coding	43	GC08M079764	1.91	https://www.genecards.org/cgi-bin/ca
Protein Coding	48	GC12M054396	1.91	https://www.genecards.org/cgi-bin/ca

Protein Coding	40	GC02P176108	1.91	https://www.genecards.org/cgi-bin/ca
Protein Coding	43	GC11P014643	1.91	https://www.genecards.org/cgi-bin/ca
Protein Coding	39	GC04M076001	1.91	https://www.genecards.org/cgi-bin/ca
Protein Coding	41	GC08M090058	1.9	https://www.genecards.org/cgi-bin/ca
Protein Coding	43	GC03M087259	1.9	https://www.genecards.org/cgi-bin/ca
Protein Coding	43	GC02M218162	1.9	https://www.genecards.org/cgi-bin/ca
Protein Coding	42	GC20M062307	1.89	https://www.genecards.org/cgi-bin/ca
Protein Coding	42	GC16P057372	1.89	https://www.genecards.org/cgi-bin/ca
RNA Gene	18	GC01M065058	1.89	https://www.genecards.org/cgi-bin/ca
Protein Coding	44	GC12M006570	1.89	https://www.genecards.org/cgi-bin/ca
Protein Coding	44	GC01M182542	1.89	https://www.genecards.org/cgi-bin/ca
Protein Coding	48	GC06P043576	1.89	https://www.genecards.org/cgi-bin/ca
Protein Coding	42	GC11M003671	1.88	https://www.genecards.org/cgi-bin/ca
Protein Coding	44	GC19P048695	1.88	https://www.genecards.org/cgi-bin/ca
Protein Coding	40	GC19M040447	1.88	https://www.genecards.org/cgi-bin/ca
Protein Coding	46	GC08M024950	1.88	https://www.genecards.org/cgi-bin/ca
Protein Coding	42	GC03P188153	1.88	https://www.genecards.org/cgi-bin/ca
Protein Coding	40	GC01M092246	1.88	https://www.genecards.org/cgi-bin/ca
Protein Coding	41	GC08M094127	1.88	https://www.genecards.org/cgi-bin/ca
Protein Coding	39	GC17M048958	1.88	https://www.genecards.org/cgi-bin/ca
Protein Coding	46	GC17P059619	1.87	https://www.genecards.org/cgi-bin/ca
Protein Coding	47	GC11M077321	1.87	https://www.genecards.org/cgi-bin/ca
Protein Coding	48	GC16P029020	1.87	https://www.genecards.org/cgi-bin/ca
Protein Coding	41	GC01M047250	1.87	https://www.genecards.org/cgi-bin/ca
Protein Coding	44	GC13M037636	1.87	https://www.genecards.org/cgi-bin/ca
Protein Coding	44	GC01P040979	1.87	https://www.genecards.org/cgi-bin/ca
Protein Coding	48	GC10M092451	1.87	https://www.genecards.org/cgi-bin/ca
Protein Coding	42	GC11M015949	1.86	https://www.genecards.org/cgi-bin/ca
Protein Coding	46	GC12M023529	1.86	https://www.genecards.org/cgi-bin/ca
Protein Coding	37	GC11M111352	1.86	https://www.genecards.org/cgi-bin/ca
Protein Coding	43	GC05P132294	1.86	https://www.genecards.org/cgi-bin/ca
Protein Coding	40	GC04M074097	1.86	https://www.genecards.org/cgi-bin/ca
Protein Coding	43	GC03M038103	1.86	https://www.genecards.org/cgi-bin/ca
Protein Coding	36	GC10M073631	1.86	https://www.genecards.org/cgi-bin/ca
Protein Coding	44	GC07M030424	1.86	https://www.genecards.org/cgi-bin/ca
Protein Coding	50	GC01M211658	1.86	https://www.genecards.org/cgi-bin/ca
Protein Coding	41	GC20P017226	1.86	https://www.genecards.org/cgi-bin/ca
Protein Coding	42	GC03P097764	1.85	https://www.genecards.org/cgi-bin/ca
Protein Coding	40	GC0XM139581	1.85	https://www.genecards.org/cgi-bin/ca
Protein Coding	44	GC11M104995	1.85	https://www.genecards.org/cgi-bin/ca
Protein Coding	45	GC02P119366	1.85	https://www.genecards.org/cgi-bin/ca
Protein Coding	40	GC19P039972	1.85	https://www.genecards.org/cgi-bin/ca
Protein Coding	47	GC19P001103	1.84	https://www.genecards.org/cgi-bin/ca
Protein Coding	45	GC16P001405	1.84	https://www.genecards.org/cgi-bin/ca
Protein Coding	49	GC17M081843	1.84	https://www.genecards.org/cgi-bin/ca
Protein Coding	42	GC03P142724	1.83	https://www.genecards.org/cgi-bin/ca
Protein Coding	33	GC12M014768	1.83	https://www.genecards.org/cgi-bin/ca
Protein Coding	37	GC02M027308	1.83	https://www.genecards.org/cgi-bin/ca
Protein Coding	49	GC05M141583	1.83	https://www.genecards.org/cgi-bin/ca
Protein Coding	44	GC09P136881	1.83	https://www.genecards.org/cgi-bin/ca
Protein Coding	37	GC01M226231	1.82	https://www.genecards.org/cgi-bin/ca
Protein Coding	34	GC03M184710	1.82	https://www.genecards.org/cgi-bin/ca
RNA Gene	17	GC18M021826	1.82	https://www.genecards.org/cgi-bin/ca
Protein Coding	48	GC09P078297	1.82	https://www.genecards.org/cgi-bin/ca

Protein Coding	42	GC17M044800	1.81	https://www.genecards.org/cgi-bin/ca
Protein Coding	42	GC17M006684	1.81	https://www.genecards.org/cgi-bin/ca
Protein Coding	41	GC01M159062	1.81	https://www.genecards.org/cgi-bin/ca
Protein Coding	39	GC11M002944	1.81	https://www.genecards.org/cgi-bin/ca
Protein Coding	43	GC12M052806	1.81	https://www.genecards.org/cgi-bin/ca
Protein Coding	48	GC15P096325	1.81	https://www.genecards.org/cgi-bin/ca
Protein Coding	41	GC19M047220	1.81	https://www.genecards.org/cgi-bin/ca
Protein Coding	46	GC16M055836	1.8	https://www.genecards.org/cgi-bin/ca
Protein Coding	39	GC07M121349	1.8	https://www.genecards.org/cgi-bin/ca
Protein Coding	38	GC05P111070	1.8	https://www.genecards.org/cgi-bin/ca
Protein Coding	48	GC09M091564	1.79	https://www.genecards.org/cgi-bin/ca
Protein Coding	47	GC20P009024	1.79	https://www.genecards.org/cgi-bin/ca
Protein Coding	47	GC01P161766	1.79	https://www.genecards.org/cgi-bin/ca
Protein Coding	41	GC05M132875	1.79	https://www.genecards.org/cgi-bin/ca
Protein Coding	34	GC12M013889	1.79	https://www.genecards.org/cgi-bin/ca
Protein Coding	41	GC20P031739	1.78	https://www.genecards.org/cgi-bin/ca
Protein Coding	44	GC12M013864	1.78	https://www.genecards.org/cgi-bin/ca
Protein Coding	44	GC07P023238	1.78	https://www.genecards.org/cgi-bin/ca
Protein Coding	43	GC07P101815	1.78	https://www.genecards.org/cgi-bin/ca
Protein Coding	39	GC17P049968	1.78	https://www.genecards.org/cgi-bin/ca
Protein Coding	44	GC06M160173	1.78	https://www.genecards.org/cgi-bin/ca
Protein Coding	41	GC05P136132	1.78	https://www.genecards.org/cgi-bin/ca
Protein Coding	45	GC03M070926	1.78	https://www.genecards.org/cgi-bin/ca
Protein Coding	39	GC04M128269	1.78	https://www.genecards.org/cgi-bin/ca
Protein Coding	47	GC08M144316	1.78	https://www.genecards.org/cgi-bin/ca
Protein Coding	44	GC01P179262	1.78	https://www.genecards.org/cgi-bin/ca
Protein Coding	44	GC11P061792	1.78	https://www.genecards.org/cgi-bin/ca
Protein Coding	39	GC08M123319	1.78	https://www.genecards.org/cgi-bin/ca
Protein Coding	38	GC10P007703	1.77	https://www.genecards.org/cgi-bin/ca
Protein Coding	42	GC06M043037	1.77	https://www.genecards.org/cgi-bin/ca
Protein Coding	40	GC06M004115	1.77	https://www.genecards.org/cgi-bin/ca
RNA Gene	21	GC06P052148	1.77	https://www.genecards.org/cgi-bin/ca
Protein Coding	41	GC03M008775	1.76	https://www.genecards.org/cgi-bin/ca
Protein Coding	42	GC11M004384	1.76	https://www.genecards.org/cgi-bin/ca
Protein Coding	38	GC15P032641	1.76	https://www.genecards.org/cgi-bin/ca
Protein Coding	45	GC05P136027	1.76	https://www.genecards.org/cgi-bin/ca
Protein Coding	44	GC17M019737	1.76	https://www.genecards.org/cgi-bin/ca
Protein Coding	44	GC12P057549	1.75	https://www.genecards.org/cgi-bin/ca
Protein Coding	47	GC09M032455	1.75	https://www.genecards.org/cgi-bin/ca
Protein Coding	42	GC0XM068042	1.75	https://www.genecards.org/cgi-bin/ca
Protein Coding	41	GC13M020139	1.75	https://www.genecards.org/cgi-bin/ca
Protein Coding	39	GC14P092794	1.75	https://www.genecards.org/cgi-bin/ca
Protein Coding	44	GC07M100620	1.75	https://www.genecards.org/cgi-bin/ca
Protein Coding	39	GC07M027128	1.75	https://www.genecards.org/cgi-bin/ca
Protein Coding	41	GC22M045394	1.75	https://www.genecards.org/cgi-bin/ca
Protein Coding	51	GC06M113933	1.74	https://www.genecards.org/cgi-bin/ca
Protein Coding	46	GC01M070852	1.74	https://www.genecards.org/cgi-bin/ca
Protein Coding	41	GC19P000797	1.74	https://www.genecards.org/cgi-bin/ca
Protein Coding	37	GC11P086791	1.74	https://www.genecards.org/cgi-bin/ca
Protein Coding	48	GC10M095038	1.74	https://www.genecards.org/cgi-bin/ca
Protein Coding	39	GC03P009960	1.73	https://www.genecards.org/cgi-bin/ca
Protein Coding	44	GC09P134135	1.73	https://www.genecards.org/cgi-bin/ca
Protein Coding	43	GC05P081232	1.73	https://www.genecards.org/cgi-bin/ca
Protein Coding	41	GC07P141551	1.73	https://www.genecards.org/cgi-bin/ca

Protein Coding	45	GC20P058389	1.72	https://www.genecards.org/cgi-bin/ca
Protein Coding	47	GC20M049503	1.72	https://www.genecards.org/cgi-bin/ca
Protein Coding	37	GC14P076761	1.72	https://www.genecards.org/cgi-bin/ca
Protein Coding	52	GC19M010350	1.72	https://www.genecards.org/cgi-bin/ca
Protein Coding	45	GC08P093857	1.71	https://www.genecards.org/cgi-bin/ca
Protein Coding	42	GC10P047322	1.71	https://www.genecards.org/cgi-bin/ca
Protein Coding	32	GC08M128215	1.71	https://www.genecards.org/cgi-bin/ca
Protein Coding	45	GC06M117287	1.71	https://www.genecards.org/cgi-bin/ca
Protein Coding	45	GC16P050363	1.71	https://www.genecards.org/cgi-bin/ca
Protein Coding	45	GC03M052401	1.71	https://www.genecards.org/cgi-bin/ca
Protein Coding	43	GC06P007107	1.71	https://www.genecards.org/cgi-bin/ca
RNA Gene	16	GC05M055172	1.7	https://www.genecards.org/cgi-bin/ca
Protein Coding	43	GC01M001211	1.7	https://www.genecards.org/cgi-bin/ca
Protein Coding	42	GC05P163480	1.7	https://www.genecards.org/cgi-bin/ca
Protein Coding	46	GC07M000549	1.7	https://www.genecards.org/cgi-bin/ca
Protein Coding	51	GC11P018394	1.7	https://www.genecards.org/cgi-bin/ca
Protein Coding	30	GC19P038304	1.7	https://www.genecards.org/cgi-bin/ca
Protein Coding	42	GC13P023160	1.7	https://www.genecards.org/cgi-bin/ca
Protein Coding	41	GC09P130172	1.7	https://www.genecards.org/cgi-bin/ca
Protein Coding	43	GC0XM001386	1.69	https://www.genecards.org/cgi-bin/ca
Protein Coding	41	GC10P010462	1.69	https://www.genecards.org/cgi-bin/ca
Protein Coding	43	GC09P127794	1.69	https://www.genecards.org/cgi-bin/ca
Protein Coding	44	GC08P144291	1.69	https://www.genecards.org/cgi-bin/ca
Protein Coding	39	GC11P124739	1.69	https://www.genecards.org/cgi-bin/ca
Protein Coding	41	GC01M155335	1.69	https://www.genecards.org/cgi-bin/ca
Protein Coding	41	GC01M093885	1.69	https://www.genecards.org/cgi-bin/ca
Protein Coding	44	GC05P007396	1.69	https://www.genecards.org/cgi-bin/ca
Protein Coding	45	GC07M099027	1.69	https://www.genecards.org/cgi-bin/ca
Protein Coding	44	GC19M014514	1.68	https://www.genecards.org/cgi-bin/ca
Protein Coding	43	GC01M156701	1.68	https://www.genecards.org/cgi-bin/ca
Protein Coding	43	GC11M115169	1.67	https://www.genecards.org/cgi-bin/ca
Protein Coding	37	GC16P005240	1.67	https://www.genecards.org/cgi-bin/ca
Protein Coding	45	GC04P186265	1.67	https://www.genecards.org/cgi-bin/ca
Protein Coding	44	GC10P013099	1.67	https://www.genecards.org/cgi-bin/ca
Protein Coding	43	GC11P064203	1.67	https://www.genecards.org/cgi-bin/ca
Protein Coding	42	GC11M089324	1.67	https://www.genecards.org/cgi-bin/ca
Protein Coding	47	GC06P149218	1.67	https://www.genecards.org/cgi-bin/ca
Protein Coding	35	GC16P031527	1.66	https://www.genecards.org/cgi-bin/ca
RNA Gene	19	GC13P091433	1.66	https://www.genecards.org/cgi-bin/ca
Protein Coding	42	GC03M052812	1.65	https://www.genecards.org/cgi-bin/ca
Protein Coding	40	GC08M123498	1.65	https://www.genecards.org/cgi-bin/ca
Protein Coding	49	GC18P041955	1.65	https://www.genecards.org/cgi-bin/ca
Protein Coding	36	GC14M055366	1.65	https://www.genecards.org/cgi-bin/ca
Protein Coding	50	GC17M044388	1.65	https://www.genecards.org/cgi-bin/ca
Protein Coding	36	GC11P001948	1.65	https://www.genecards.org/cgi-bin/ca
Protein Coding	41	GC12P104455	1.65	https://www.genecards.org/cgi-bin/ca
Protein Coding	39	GC02P026750	1.64	https://www.genecards.org/cgi-bin/ca
Protein Coding	41	GC0XM153785	1.64	https://www.genecards.org/cgi-bin/ca
Protein Coding	31	GC10M086436	1.64	https://www.genecards.org/cgi-bin/ca
Protein Coding	38	GC02M068865	1.64	https://www.genecards.org/cgi-bin/ca
Protein Coding	37	GC11M061128	1.64	https://www.genecards.org/cgi-bin/ca
Protein Coding	37	GC01M145992	1.64	https://www.genecards.org/cgi-bin/ca
Protein Coding	41	GC05M157028	1.64	https://www.genecards.org/cgi-bin/ca
Protein Coding	43	GC0XM049028	1.64	https://www.genecards.org/cgi-bin/ca

Protein Coding	44	GC04P110365	1.63	https://www.genecards.org/cgi-bin/ca
Protein Coding	39	GC17P039637	1.63	https://www.genecards.org/cgi-bin/ca
Protein Coding	44	GC03P170357	1.63	https://www.genecards.org/cgi-bin/ca
Protein Coding	50	GC12P008223	1.63	https://www.genecards.org/cgi-bin/ca
Protein Coding	41	GC17M063432	1.63	https://www.genecards.org/cgi-bin/ca
Protein Coding	27	GC02M003188	1.62	https://www.genecards.org/cgi-bin/ca
Protein Coding	40	GC16M074621	1.62	https://www.genecards.org/cgi-bin/ca
Protein Coding	43	GC01P182992	1.62	https://www.genecards.org/cgi-bin/ca
Protein Coding	48	GC01P002050	1.62	https://www.genecards.org/cgi-bin/ca
Protein Coding	44	GC05P053560	1.62	https://www.genecards.org/cgi-bin/ca
Protein Coding	45	GC22P024417	1.62	https://www.genecards.org/cgi-bin/ca
Protein Coding	43	GC03P194136	1.62	https://www.genecards.org/cgi-bin/ca
Protein Coding	44	GC04M099123	1.62	https://www.genecards.org/cgi-bin/ca
Protein Coding	36	GC10M017589	1.62	https://www.genecards.org/cgi-bin/ca
Protein Coding	38	GC16M028511	1.61	https://www.genecards.org/cgi-bin/ca
Protein Coding	45	GC05P051383	1.61	https://www.genecards.org/cgi-bin/ca
Protein Coding	41	GC03M047413	1.61	https://www.genecards.org/cgi-bin/ca
Protein Coding	33	GC11P002400	1.61	https://www.genecards.org/cgi-bin/ca
Protein Coding	34	GC19M005786	1.6	https://www.genecards.org/cgi-bin/ca
Protein Coding	43	GC01P204516	1.6	https://www.genecards.org/cgi-bin/ca
Protein Coding	39	GC22M036800	1.6	https://www.genecards.org/cgi-bin/ca
Protein Coding	40	GC11P043636	1.6	https://www.genecards.org/cgi-bin/ca
Protein Coding	46	GC11M000612	1.6	https://www.genecards.org/cgi-bin/ca
Protein Coding	39	GC07M150333	1.6	https://www.genecards.org/cgi-bin/ca
Protein Coding	41	GC01M035599	1.59	https://www.genecards.org/cgi-bin/ca
Protein Coding	41	GC07M047974	1.59	https://www.genecards.org/cgi-bin/ca
Protein Coding	49	GC12P040196	1.59	https://www.genecards.org/cgi-bin/ca
Protein Coding	43	GC01M058575	1.59	https://www.genecards.org/cgi-bin/ca
Protein Coding	33	GC17P018183	1.59	https://www.genecards.org/cgi-bin/ca
Protein Coding	43	GC19M046760	1.59	https://www.genecards.org/cgi-bin/ca
Protein Coding	42	GC04M094298	1.59	https://www.genecards.org/cgi-bin/ca
Protein Coding	44	GC03P039406	1.59	https://www.genecards.org/cgi-bin/ca
Protein Coding	41	GC11P126355	1.59	https://www.genecards.org/cgi-bin/ca
Protein Coding	45	GC22P021549	1.58	https://www.genecards.org/cgi-bin/ca
Protein Coding	45	GC17P042561	1.58	https://www.genecards.org/cgi-bin/ca
Protein Coding	40	GC08P101492	1.58	https://www.genecards.org/cgi-bin/ca
Protein Coding	41	GC08P017922	1.58	https://www.genecards.org/cgi-bin/ca
Protein Coding	39	GC09P135521	1.58	https://www.genecards.org/cgi-bin/ca
Protein Coding	43	GC11M119355	1.58	https://www.genecards.org/cgi-bin/ca
Protein Coding	35	GC01M247297	1.58	https://www.genecards.org/cgi-bin/ca
Protein Coding	43	GC11M074000	1.58	https://www.genecards.org/cgi-bin/ca
Protein Coding	40	GC06P087470	1.58	https://www.genecards.org/cgi-bin/ca
Protein Coding	46	GC18P058044	1.58	https://www.genecards.org/cgi-bin/ca
Protein Coding	42	GC18P026225	1.57	https://www.genecards.org/cgi-bin/ca
Protein Coding	42	GC21P038805	1.57	https://www.genecards.org/cgi-bin/ca
Protein Coding	46	GC02P060881	1.57	https://www.genecards.org/cgi-bin/ca
Protein Coding	45	GC22M036467	1.56	https://www.genecards.org/cgi-bin/ca
Protein Coding	45	GC09P127814	1.56	https://www.genecards.org/cgi-bin/ca
Protein Coding	41	GC03P004997	1.56	https://www.genecards.org/cgi-bin/ca
Protein Coding	39	GC09P104094	1.56	https://www.genecards.org/cgi-bin/ca
Protein Coding	44	GC03P193594	1.56	https://www.genecards.org/cgi-bin/ca
Protein Coding	45	GC06P044246	1.55	https://www.genecards.org/cgi-bin/ca
Protein Coding	39	GC17M007288	1.55	https://www.genecards.org/cgi-bin/ca
Protein Coding	37	GC19P038214	1.55	https://www.genecards.org/cgi-bin/ca

Protein Coding	36	GC15M034851	1.55	https://www.genecards.org/cgi-bin/ca
Protein Coding	35	GC03P197961	1.55	https://www.genecards.org/cgi-bin/ca
Protein Coding	42	GC19P003506	1.55	https://www.genecards.org/cgi-bin/ca
Protein Coding	36	GC14P049767	1.55	https://www.genecards.org/cgi-bin/ca
Protein Coding	44	GC01P034781	1.54	https://www.genecards.org/cgi-bin/ca
Protein Coding	41	GC02P234951	1.54	https://www.genecards.org/cgi-bin/ca
Protein Coding	40	GC01P034759	1.54	https://www.genecards.org/cgi-bin/ca
Protein Coding	47	GC20M034928	1.54	https://www.genecards.org/cgi-bin/ca
Protein Coding	35	GC02P176099	1.54	https://www.genecards.org/cgi-bin/ca
Protein Coding	43	GC12M018604	1.54	https://www.genecards.org/cgi-bin/ca
Protein Coding	40	GC05M054977	1.54	https://www.genecards.org/cgi-bin/ca
Protein Coding	47	GC0XP065588	1.53	https://www.genecards.org/cgi-bin/ca
Protein Coding	44	GC05M142310	1.53	https://www.genecards.org/cgi-bin/ca
Protein Coding	41	GC09M136196	1.53	https://www.genecards.org/cgi-bin/ca
Protein Coding	39	GC20M010412	1.53	https://www.genecards.org/cgi-bin/ca
Protein Coding	42	GC17M058982	1.53	https://www.genecards.org/cgi-bin/ca
Protein Coding	44	GC06M052896	1.52	https://www.genecards.org/cgi-bin/ca
Protein Coding	42	GC19P055363	1.52	https://www.genecards.org/cgi-bin/ca
Protein Coding	50	GC0XP110944	1.52	https://www.genecards.org/cgi-bin/ca
Protein Coding	40	GC17M004733	1.52	https://www.genecards.org/cgi-bin/ca
Protein Coding	39	GC01M160876	1.52	https://www.genecards.org/cgi-bin/ca
Protein Coding	46	GC12M021635	1.52	https://www.genecards.org/cgi-bin/ca
Protein Coding	40	GC06M043522	1.52	https://www.genecards.org/cgi-bin/ca
Protein Coding	35	GC02M233834	1.52	https://www.genecards.org/cgi-bin/ca
Protein Coding	39	GC03M050389	1.51	https://www.genecards.org/cgi-bin/ca
Protein Coding	48	GC01M231363	1.51	https://www.genecards.org/cgi-bin/ca
Protein Coding	41	GC05M138957	1.51	https://www.genecards.org/cgi-bin/ca
Protein Coding	36	GC0XP011293	1.51	https://www.genecards.org/cgi-bin/ca
Protein Coding	41	GC02M182833	1.51	https://www.genecards.org/cgi-bin/ca
Protein Coding	41	GC02M065048	1.51	https://www.genecards.org/cgi-bin/ca
Protein Coding	40	GC04P109433	1.51	https://www.genecards.org/cgi-bin/ca
Protein Coding	39	GC10M027182	1.51	https://www.genecards.org/cgi-bin/ca
Protein Coding	32	GC11P086303	1.51	https://www.genecards.org/cgi-bin/ca
Protein Coding	31	GC11P035618	1.51	https://www.genecards.org/cgi-bin/ca
Protein Coding	27	GC01P025430	1.51	https://www.genecards.org/cgi-bin/ca
Protein Coding	45	GC02M165747	1.5	https://www.genecards.org/cgi-bin/ca
Protein Coding	47	GC10P123154	1.5	https://www.genecards.org/cgi-bin/ca
Protein Coding	43	GC03P130611	1.5	https://www.genecards.org/cgi-bin/ca
Protein Coding	47	GC17M081867	1.5	https://www.genecards.org/cgi-bin/ca
Protein Coding	30	GC08M007430	1.5	https://www.genecards.org/cgi-bin/ca
Protein Coding	40	GC18M054151	1.5	https://www.genecards.org/cgi-bin/ca
Protein Coding	43	GC0XM120524	1.5	https://www.genecards.org/cgi-bin/ca
Protein Coding	39	GC03M015777	1.49	https://www.genecards.org/cgi-bin/ca
Protein Coding	39	GC06P113857	1.49	https://www.genecards.org/cgi-bin/ca
Protein Coding	47	GC17P069414	1.49	https://www.genecards.org/cgi-bin/ca
Protein Coding	47	GC01P223701	1.49	https://www.genecards.org/cgi-bin/ca
Protein Coding	47	GC06M032865	1.49	https://www.genecards.org/cgi-bin/ca
Protein Coding	35	GC12P122842	1.48	https://www.genecards.org/cgi-bin/ca
Protein Coding	33	GC05P037387	1.48	https://www.genecards.org/cgi-bin/ca
Protein Coding	48	GC09P087497	1.48	https://www.genecards.org/cgi-bin/ca
Protein Coding	43	GC17M080135	1.48	https://www.genecards.org/cgi-bin/ca
Protein Coding	47	GC09P004490	1.47	https://www.genecards.org/cgi-bin/ca
Protein Coding	41	GC07M092198	1.47	https://www.genecards.org/cgi-bin/ca
Protein Coding	48	GC12M049184	1.47	https://www.genecards.org/cgi-bin/ca

Protein Coding	34	GC15M042210	1.47	https://www.genecards.org/cgi-bin/ca
Protein Coding	45	GC20P064080	1.47	https://www.genecards.org/cgi-bin/ca
RNA Gene	8	GC06U903133	1.47	https://www.genecards.org/cgi-bin/ca
Protein Coding	41	GC13M052132	1.46	https://www.genecards.org/cgi-bin/ca
Protein Coding	37	GC06M152970	1.46	https://www.genecards.org/cgi-bin/ca
Protein Coding	45	GC03P184155	1.46	https://www.genecards.org/cgi-bin/ca
Protein Coding	40	GC19P023267	1.45	https://www.genecards.org/cgi-bin/ca
Protein Coding	32	GC10M071748	1.45	https://www.genecards.org/cgi-bin/ca
Protein Coding	43	GC03P011273	1.45	https://www.genecards.org/cgi-bin/ca
Protein Coding	41	GC02P218859	1.44	https://www.genecards.org/cgi-bin/ca
Protein Coding	40	GC05M135581	1.44	https://www.genecards.org/cgi-bin/ca
Protein Coding	45	GC12P112906	1.44	https://www.genecards.org/cgi-bin/ca
Protein Coding	43	GC15P090388	1.44	https://www.genecards.org/cgi-bin/ca
Protein Coding	42	GC08P116950	1.44	https://www.genecards.org/cgi-bin/ca
Protein Coding	40	GC06P047372	1.44	https://www.genecards.org/cgi-bin/ca
Protein Coding	47	GC02M162267	1.44	https://www.genecards.org/cgi-bin/ca
Protein Coding	44	GC15P043746	1.44	https://www.genecards.org/cgi-bin/ca
Protein Coding	44	GC17P007558	1.44	https://www.genecards.org/cgi-bin/ca
Protein Coding	35	GC02P204545	1.44	https://www.genecards.org/cgi-bin/ca
Protein Coding	31	GC15M103936	1.43	https://www.genecards.org/cgi-bin/ca
Protein Coding	44	GC01P078303	1.43	https://www.genecards.org/cgi-bin/ca
Protein Coding	40	GC21P033025	1.43	https://www.genecards.org/cgi-bin/ca
Protein Coding	44	GC13P110305	1.43	https://www.genecards.org/cgi-bin/ca
Protein Coding	44	GC06M033318	1.43	https://www.genecards.org/cgi-bin/ca
Protein Coding	46	GC14P036657	1.43	https://www.genecards.org/cgi-bin/ca
Protein Coding	33	GC11M006996	1.43	https://www.genecards.org/cgi-bin/ca
Protein Coding	47	GC11P034894	1.43	https://www.genecards.org/cgi-bin/ca
Protein Coding	43	GC04P155173	1.43	https://www.genecards.org/cgi-bin/ca
Protein Coding	38	GC04M121824	1.42	https://www.genecards.org/cgi-bin/ca
Protein Coding	45	GC04P020287	1.42	https://www.genecards.org/cgi-bin/ca
Protein Coding	48	GC16P001415	1.42	https://www.genecards.org/cgi-bin/ca
Protein Coding	40	GC08P038728	1.42	https://www.genecards.org/cgi-bin/ca
Protein Coding	31	GCMTP012339	1.42	https://www.genecards.org/cgi-bin/ca
Protein Coding	43	GC04P003041	1.42	https://www.genecards.org/cgi-bin/ca
RNA Gene	14	GC14P104808	1.42	https://www.genecards.org/cgi-bin/ca
Protein Coding	48	GC12M089347	1.42	https://www.genecards.org/cgi-bin/ca
Protein Coding	43	GC17M048528	1.42	https://www.genecards.org/cgi-bin/ca
Protein Coding	44	GC05P179678	1.41	https://www.genecards.org/cgi-bin/ca
Protein Coding	44	GC05P175659	1.41	https://www.genecards.org/cgi-bin/ca
Protein Coding	46	GC12P111405	1.41	https://www.genecards.org/cgi-bin/ca
Protein Coding	45	GC11P068030	1.41	https://www.genecards.org/cgi-bin/ca
Protein Coding	50	GC09P002611	1.41	https://www.genecards.org/cgi-bin/ca
Protein Coding	36	GC19P049850	1.41	https://www.genecards.org/cgi-bin/ca
Protein Coding	46	GC05P128083	1.41	https://www.genecards.org/cgi-bin/ca
Protein Coding	50	GC12P051906	1.41	https://www.genecards.org/cgi-bin/ca
Protein Coding	48	GC01M150796	1.41	https://www.genecards.org/cgi-bin/ca
Protein Coding	39	GC02M014998	1.4	https://www.genecards.org/cgi-bin/ca
Protein Coding	41	GC0XM115003	1.4	https://www.genecards.org/cgi-bin/ca
Protein Coding	43	GC0XM043766	1.4	https://www.genecards.org/cgi-bin/ca
Protein Coding	40	GC20M047656	1.4	https://www.genecards.org/cgi-bin/ca
Protein Coding	41	GC01M030931	1.4	https://www.genecards.org/cgi-bin/ca
Protein Coding	44	GC12M122271	1.4	https://www.genecards.org/cgi-bin/ca
Protein Coding	44	GC17M078992	1.4	https://www.genecards.org/cgi-bin/ca
Protein Coding	51	GC06P085449	1.4	https://www.genecards.org/cgi-bin/ca

Protein Coding	43	GC16M067944	1.4	https://www.genecards.org/cgi-bin/ca
Protein Coding	43	GC02P227813	1.4	https://www.genecards.org/cgi-bin/ca
Protein Coding	37	GC16P014974	1.39	https://www.genecards.org/cgi-bin/ca
Protein Coding	38	GC01P185734	1.39	https://www.genecards.org/cgi-bin/ca
Protein Coding	41	GC02P028392	1.39	https://www.genecards.org/cgi-bin/ca
Protein Coding	45	GC13M095019	1.38	https://www.genecards.org/cgi-bin/ca
Protein Coding	34	GC01M246945	1.38	https://www.genecards.org/cgi-bin/ca
Protein Coding	42	GC22M046684	1.38	https://www.genecards.org/cgi-bin/ca
Protein Coding	48	GC0XM041514	1.38	https://www.genecards.org/cgi-bin/ca
Protein Coding	47	GC10M004987	1.37	https://www.genecards.org/cgi-bin/ca
Protein Coding	44	GC17M045263	1.37	https://www.genecards.org/cgi-bin/ca
Protein Coding	40	GC14P052707	1.37	https://www.genecards.org/cgi-bin/ca
Protein Coding	45	GC07M014145	1.37	https://www.genecards.org/cgi-bin/ca
Protein Coding	46	GC09P137086	1.36	https://www.genecards.org/cgi-bin/ca
Protein Coding	41	GC06P043171	1.36	https://www.genecards.org/cgi-bin/ca
Protein Coding	48	GC06M111660	1.36	https://www.genecards.org/cgi-bin/ca
Protein Coding	44	GC05M175440	1.36	https://www.genecards.org/cgi-bin/ca
Protein Coding	38	GC05P005140	1.36	https://www.genecards.org/cgi-bin/ca
Protein Coding	46	GC08M139585	1.36	https://www.genecards.org/cgi-bin/ca
Protein Coding	47	GC04M102794	1.36	https://www.genecards.org/cgi-bin/ca
Protein Coding	33	GC0XP049112	1.35	https://www.genecards.org/cgi-bin/ca
Protein Coding	44	GC03P184561	1.35	https://www.genecards.org/cgi-bin/ca
Protein Coding	46	GC07P079769	1.35	https://www.genecards.org/cgi-bin/ca
Protein Coding	48	GC12P021132	1.35	https://www.genecards.org/cgi-bin/ca
Protein Coding	45	GC16P029115	1.35	https://www.genecards.org/cgi-bin/ca
Protein Coding	44	GC01M154697	1.35	https://www.genecards.org/cgi-bin/ca
Protein Coding	39	GC05M131949	1.35	https://www.genecards.org/cgi-bin/ca
Protein Coding	41	GC19P012791	1.34	https://www.genecards.org/cgi-bin/ca
Protein Coding	40	GC03P045903	1.34	https://www.genecards.org/cgi-bin/ca
Protein Coding	45	GC15M045361	1.34	https://www.genecards.org/cgi-bin/ca
Protein Coding	47	GC02M011192	1.34	https://www.genecards.org/cgi-bin/ca
Protein Coding	41	GC0XP107825	1.34	https://www.genecards.org/cgi-bin/ca
Protein Coding	40	GC22P020080	1.34	https://www.genecards.org/cgi-bin/ca
Protein Coding	41	GC05M168661	1.34	https://www.genecards.org/cgi-bin/ca
Protein Coding	39	GC11P071998	1.33	https://www.genecards.org/cgi-bin/ca
Protein Coding	44	GC03M114128	1.33	https://www.genecards.org/cgi-bin/ca
Protein Coding	45	GC03P089077	1.33	https://www.genecards.org/cgi-bin/ca
Protein Coding	40	GC22P031753	1.33	https://www.genecards.org/cgi-bin/ca
Protein Coding	42	GC16P075647	1.33	https://www.genecards.org/cgi-bin/ca
Protein Coding	48	GC20P008061	1.33	https://www.genecards.org/cgi-bin/ca
Protein Coding	47	GC12P104215	1.33	https://www.genecards.org/cgi-bin/ca
Protein Coding	39	GC02M219219	1.33	https://www.genecards.org/cgi-bin/ca
Protein Coding	43	GC16M046704	1.33	https://www.genecards.org/cgi-bin/ca
Protein Coding	38	GC21P036699	1.33	https://www.genecards.org/cgi-bin/ca
Protein Coding	34	GC07P100949	1.33	https://www.genecards.org/cgi-bin/ca
Protein Coding	43	GC04P001723	1.32	https://www.genecards.org/cgi-bin/ca
Protein Coding	44	GC05M134601	1.32	https://www.genecards.org/cgi-bin/ca
RNA Gene	14	GC01P150515	1.32	https://www.genecards.org/cgi-bin/ca
Protein Coding	43	GC19P058544	1.31	https://www.genecards.org/cgi-bin/ca
Protein Coding	37	GC09P097501	1.31	https://www.genecards.org/cgi-bin/ca
RNA Gene	16	GC11M061815	1.31	https://www.genecards.org/cgi-bin/ca
Protein Coding	40	GC14P089053	1.31	https://www.genecards.org/cgi-bin/ca
Protein Coding	45	GC01M051354	1.3	https://www.genecards.org/cgi-bin/ca
Protein Coding	40	GC22M021955	1.3	https://www.genecards.org/cgi-bin/ca

Protein Coding	39	GC01M031653	1.3	https://www.genecards.org/cgi-bin/ca
Protein Coding	36	GC04M088695	1.3	https://www.genecards.org/cgi-bin/ca
Protein Coding	31	GC11M061768	1.3	https://www.genecards.org/cgi-bin/ca
Protein Coding	43	GC02M238244	1.3	https://www.genecards.org/cgi-bin/ca
Protein Coding	39	GC20M034696	1.3	https://www.genecards.org/cgi-bin/ca
Protein Coding	48	GC09M115019	1.3	https://www.genecards.org/cgi-bin/ca
Protein Coding	41	GC19M050825	1.3	https://www.genecards.org/cgi-bin/ca
Protein Coding	45	GC12M065050	1.3	https://www.genecards.org/cgi-bin/ca
Protein Coding	42	GC07M113876	1.3	https://www.genecards.org/cgi-bin/ca
Protein Coding	41	GC21P029194	1.3	https://www.genecards.org/cgi-bin/ca
Protein Coding	45	GC12P098593	1.3	https://www.genecards.org/cgi-bin/ca
Protein Coding	49	GC20P032052	1.29	https://www.genecards.org/cgi-bin/ca
RNA Gene	19	GC02M055988	1.29	https://www.genecards.org/cgi-bin/ca
Protein Coding	42	GC11P062856	1.29	https://www.genecards.org/cgi-bin/ca
Protein Coding	44	GC03M039279	1.29	https://www.genecards.org/cgi-bin/ca
Protein Coding	39	GC14M035085	1.29	https://www.genecards.org/cgi-bin/ca
Protein Coding	36	GC16M047156	1.29	https://www.genecards.org/cgi-bin/ca
Protein Coding	45	GC01P007983	1.29	https://www.genecards.org/cgi-bin/ca
Protein Coding	36	GC03M138494	1.28	https://www.genecards.org/cgi-bin/ca
Protein Coding	41	GC07P145052	1.28	https://www.genecards.org/cgi-bin/ca
Protein Coding	35	GC12P013196	1.28	https://www.genecards.org/cgi-bin/ca
Protein Coding	46	GC01P171090	1.27	https://www.genecards.org/cgi-bin/ca
Protein Coding	35	GC11M000787	1.27	https://www.genecards.org/cgi-bin/ca
Protein Coding	44	GC03M078597	1.27	https://www.genecards.org/cgi-bin/ca
Protein Coding	34	GC17P028358	1.27	https://www.genecards.org/cgi-bin/ca
Protein Coding	43	GC20P056248	1.27	https://www.genecards.org/cgi-bin/ca
Protein Coding	43	GC05M157063	1.27	https://www.genecards.org/cgi-bin/ca
Protein Coding	41	GC06M032168	1.27	https://www.genecards.org/cgi-bin/ca
Protein Coding	41	GC14P077320	1.27	https://www.genecards.org/cgi-bin/ca
Protein Coding	40	GC04M082629	1.27	https://www.genecards.org/cgi-bin/ca
Protein Coding	38	GC07P065871	1.27	https://www.genecards.org/cgi-bin/ca
Protein Coding	37	GC19P008206	1.27	https://www.genecards.org/cgi-bin/ca
Protein Coding	37	GC0XM013789	1.27	https://www.genecards.org/cgi-bin/ca
Protein Coding	36	GC12M006976	1.27	https://www.genecards.org/cgi-bin/ca
Protein Coding	35	GC03P107522	1.27	https://www.genecards.org/cgi-bin/ca
Protein Coding	44	GC12P118135	1.26	https://www.genecards.org/cgi-bin/ca
Protein Coding	40	GC17M076734	1.26	https://www.genecards.org/cgi-bin/ca
Protein Coding	43	GC0XP071095	1.26	https://www.genecards.org/cgi-bin/ca
Protein Coding	35	GC02M218637	1.25	https://www.genecards.org/cgi-bin/ca
Protein Coding	46	GC19P034359	1.25	https://www.genecards.org/cgi-bin/ca
Protein Coding	45	GC01M001785	1.25	https://www.genecards.org/cgi-bin/ca
Protein Coding	45	GC03P011113	1.25	https://www.genecards.org/cgi-bin/ca
Protein Coding	42	GC01P158969	1.24	https://www.genecards.org/cgi-bin/ca
Protein Coding	31	GC08M081524	1.24	https://www.genecards.org/cgi-bin/ca
Protein Coding	39	GC19M051714	1.24	https://www.genecards.org/cgi-bin/ca
Protein Coding	40	GC16P056731	1.24	https://www.genecards.org/cgi-bin/ca
Protein Coding	48	GC03P170222	1.24	https://www.genecards.org/cgi-bin/ca
Protein Coding	43	GC10M119167	1.23	https://www.genecards.org/cgi-bin/ca
Protein Coding	43	GC10P028685	1.23	https://www.genecards.org/cgi-bin/ca
Protein Coding	49	GC01P119660	1.23	https://www.genecards.org/cgi-bin/ca
Protein Coding	33	GC09M108868	1.23	https://www.genecards.org/cgi-bin/ca
Protein Coding	48	GC01M043991	1.22	https://www.genecards.org/cgi-bin/ca
Protein Coding	50	GC17M001346	1.22	https://www.genecards.org/cgi-bin/ca
Protein Coding	40	GC11M011962	1.22	https://www.genecards.org/cgi-bin/ca

Protein Coding	44	GC12M052488	1.22	https://www.genecards.org/cgi-bin/ca
Protein Coding	42	GC02M231453	1.21	https://www.genecards.org/cgi-bin/ca
Protein Coding	39	GC19P039436	1.21	https://www.genecards.org/cgi-bin/ca
Protein Coding	39	GC20P004120	1.21	https://www.genecards.org/cgi-bin/ca
Protein Coding	48	GC03P020081	1.21	https://www.genecards.org/cgi-bin/ca
Protein Coding	44	GC11M119417	1.21	https://www.genecards.org/cgi-bin/ca
Protein Coding	47	GC02P101773	1.2	https://www.genecards.org/cgi-bin/ca
Protein Coding	45	GC11P117987	1.2	https://www.genecards.org/cgi-bin/ca
Protein Coding	42	GC01M171700	1.2	https://www.genecards.org/cgi-bin/ca
Protein Coding	39	GC19P010289	1.2	https://www.genecards.org/cgi-bin/ca
Protein Coding	43	GC21P033402	1.2	https://www.genecards.org/cgi-bin/ca
Protein Coding	43	GC20P031605	1.2	https://www.genecards.org/cgi-bin/ca
Protein Coding	45	GC19P049157	1.19	https://www.genecards.org/cgi-bin/ca
Protein Coding	44	GC19P019515	1.19	https://www.genecards.org/cgi-bin/ca
Protein Coding	44	GC22P035400	1.19	https://www.genecards.org/cgi-bin/ca
Protein Coding	29	GC09P094728	1.19	https://www.genecards.org/cgi-bin/ca
Protein Coding	40	GC11P034621	1.19	https://www.genecards.org/cgi-bin/ca
Protein Coding	41	GC01P040041	1.18	https://www.genecards.org/cgi-bin/ca
Protein Coding	45	GC06P075749	1.18	https://www.genecards.org/cgi-bin/ca
Protein Coding	51	GC20M000472	1.17	https://www.genecards.org/cgi-bin/ca
Protein Coding	38	GC02P085685	1.17	https://www.genecards.org/cgi-bin/ca
Protein Coding	42	GC08P120062	1.17	https://www.genecards.org/cgi-bin/ca
Protein Coding	40	GC17M056938	1.17	https://www.genecards.org/cgi-bin/ca
Protein Coding	47	GC19M033299	1.16	https://www.genecards.org/cgi-bin/ca
Protein Coding	40	GC19M048748	1.16	https://www.genecards.org/cgi-bin/ca
Protein Coding	40	GC04M052019	1.16	https://www.genecards.org/cgi-bin/ca
Protein Coding	40	GC10M046033	1.16	https://www.genecards.org/cgi-bin/ca
Protein Coding	43	GC07P129611	1.16	https://www.genecards.org/cgi-bin/ca
Protein Coding	45	GC19P014433	1.16	https://www.genecards.org/cgi-bin/ca
Protein Coding	17	GC14P021621	1.16	https://www.genecards.org/cgi-bin/ca
Protein Coding	45	GC01M020651	1.16	https://www.genecards.org/cgi-bin/ca
Protein Coding	35	GC19M055785	1.15	https://www.genecards.org/cgi-bin/ca
Protein Coding	39	GC17P058218	1.14	https://www.genecards.org/cgi-bin/ca
Protein Coding	39	GC11M006435	1.14	https://www.genecards.org/cgi-bin/ca
Protein Coding	45	GC08M053227	1.14	https://www.genecards.org/cgi-bin/ca
Protein Coding	43	GC18P009904	1.13	https://www.genecards.org/cgi-bin/ca
Protein Coding	41	GC04M105369	1.13	https://www.genecards.org/cgi-bin/ca
Protein Coding	42	GC02P206159	1.13	https://www.genecards.org/cgi-bin/ca
Protein Coding	39	GC04P094451	1.13	https://www.genecards.org/cgi-bin/ca
Protein Coding	47	GC15P041557	1.13	https://www.genecards.org/cgi-bin/ca
Protein Coding	45	GC17M005432	1.13	https://www.genecards.org/cgi-bin/ca
Protein Coding	48	GC17P040058	1.12	https://www.genecards.org/cgi-bin/ca
Protein Coding	47	GC04P186208	1.12	https://www.genecards.org/cgi-bin/ca
Protein Coding	43	GC16P074296	1.12	https://www.genecards.org/cgi-bin/ca
Protein Coding	41	GC17M047117	1.12	https://www.genecards.org/cgi-bin/ca
Protein Coding	31	GC06P026041	1.12	https://www.genecards.org/cgi-bin/ca
Protein Coding	40	GC02P151357	1.12	https://www.genecards.org/cgi-bin/ca
Protein Coding	28	GC01M001375	1.12	https://www.genecards.org/cgi-bin/ca
Protein Coding	48	GC15P074782	1.11	https://www.genecards.org/cgi-bin/ca
Protein Coding	39	GC19P008117	1.11	https://www.genecards.org/cgi-bin/ca
Protein Coding	40	GC02P231056	1.11	https://www.genecards.org/cgi-bin/ca
Protein Coding	46	GC18M017325	1.11	https://www.genecards.org/cgi-bin/ca
Protein Coding	44	GC05P032689	1.11	https://www.genecards.org/cgi-bin/ca
Protein Coding	47	GC06M033193	1.11	https://www.genecards.org/cgi-bin/ca

Protein Coding	44	GC14P025306	1.1 https://www.genecards.org/cgi-bin/ca
Protein Coding	48	GC03M123282	1.1 https://www.genecards.org/cgi-bin/ca
Protein Coding	34	GC11P004706	1.1 https://www.genecards.org/cgi-bin/ca
Protein Coding	46	GC12M024732	1.1 https://www.genecards.org/cgi-bin/ca
Protein Coding	37	GC01P212035	1.1 https://www.genecards.org/cgi-bin/ca
Protein Coding	44	GC01M059893	1.09 https://www.genecards.org/cgi-bin/ca
Protein Coding	41	GC06P047472	1.09 https://www.genecards.org/cgi-bin/ca
Protein Coding	45	GC11P111605	1.09 https://www.genecards.org/cgi-bin/ca
Protein Coding	38	GC12M095516	1.09 https://www.genecards.org/cgi-bin/ca
Protein Coding	38	GC16P057359	1.09 https://www.genecards.org/cgi-bin/ca
Protein Coding	43	GC16M003652	1.09 https://www.genecards.org/cgi-bin/ca
Protein Coding	45	GC12P103930	1.09 https://www.genecards.org/cgi-bin/ca
Protein Coding	47	GC19P048965	1.08 https://www.genecards.org/cgi-bin/ca
Protein Coding	39	GC16P029811	1.08 https://www.genecards.org/cgi-bin/ca
Protein Coding	47	GC19M051745	1.08 https://www.genecards.org/cgi-bin/ca
Protein Coding	48	GC17P019379	1.08 https://www.genecards.org/cgi-bin/ca
Protein Coding	43	GC09P116687	1.07 https://www.genecards.org/cgi-bin/ca
Protein Coding	46	GC02M202206	1.07 https://www.genecards.org/cgi-bin/ca
RNA Gene	18	GC02M055982	1.07 https://www.genecards.org/cgi-bin/ca
Protein Coding	43	GC07P026201	1.07 https://www.genecards.org/cgi-bin/ca
Protein Coding	40	GC18P063752	1.07 https://www.genecards.org/cgi-bin/ca
Protein Coding	47	GC18P058671	1.07 https://www.genecards.org/cgi-bin/ca
Protein Coding	39	GC06M031588	1.06 https://www.genecards.org/cgi-bin/ca
Protein Coding	36	GC19M048296	1.06 https://www.genecards.org/cgi-bin/ca
Protein Coding	45	GC0XP119574	1.06 https://www.genecards.org/cgi-bin/ca
Protein Coding	44	GC13P073054	1.06 https://www.genecards.org/cgi-bin/ca
Protein Coding	47	GC01M001335	1.06 https://www.genecards.org/cgi-bin/ca
Protein Coding	47	GC19P009835	1.06 https://www.genecards.org/cgi-bin/ca
Protein Coding	38	GC06P028525	1.06 https://www.genecards.org/cgi-bin/ca
Protein Coding	41	GC01P215005	1.06 https://www.genecards.org/cgi-bin/ca
Protein Coding	35	GC12M026971	1.06 https://www.genecards.org/cgi-bin/ca
Protein Coding	41	GC06P047300	1.06 https://www.genecards.org/cgi-bin/ca
Protein Coding	40	GC17P050373	1.06 https://www.genecards.org/cgi-bin/ca
Protein Coding	42	GC06M155247	1.05 https://www.genecards.org/cgi-bin/ca
Protein Coding	40	GC02M157526	1.05 https://www.genecards.org/cgi-bin/ca
Protein Coding	33	GC0XP102247	1.05 https://www.genecards.org/cgi-bin/ca
Protein Coding	40	GC16M067927	1.05 https://www.genecards.org/cgi-bin/ca
Protein Coding	48	GC08M081656	1.05 https://www.genecards.org/cgi-bin/ca
Protein Coding	41	GC06M052209	1.05 https://www.genecards.org/cgi-bin/ca
Protein Coding	49	GC17P026749	1.05 https://www.genecards.org/cgi-bin/ca
Protein Coding	40	GC08P037736	1.05 https://www.genecards.org/cgi-bin/ca
Protein Coding	49	GC10M006393	1.04 https://www.genecards.org/cgi-bin/ca
Protein Coding	39	GC09M015456	1.04 https://www.genecards.org/cgi-bin/ca
Protein Coding	41	GC01P029236	1.04 https://www.genecards.org/cgi-bin/ca
Protein Coding	39	GC03M186046	1.04 https://www.genecards.org/cgi-bin/ca
Protein Coding	49	GC12P064451	1.04 https://www.genecards.org/cgi-bin/ca
Protein Coding	43	GC19M042507	1.04 https://www.genecards.org/cgi-bin/ca
Protein Coding	42	GC04P081030	1.04 https://www.genecards.org/cgi-bin/ca
Protein Coding	46	GC17M008474	1.04 https://www.genecards.org/cgi-bin/ca
Protein Coding	43	GC17M075273	1.04 https://www.genecards.org/cgi-bin/ca
Protein Coding	50	GC09M094603	1.03 https://www.genecards.org/cgi-bin/ca
Protein Coding	39	GC01M200521	1.03 https://www.genecards.org/cgi-bin/ca
Protein Coding	41	GC22P043923	1.03 https://www.genecards.org/cgi-bin/ca
Protein Coding	40	GC04M073995	1.03 https://www.genecards.org/cgi-bin/ca

Protein Coding	43	GC09M120902	1.03	https://www.genecards.org/cgi-bin/ca
Protein Coding	37	GC12P057434	1.02	https://www.genecards.org/cgi-bin/ca
Protein Coding	45	GC02M037099	1.02	https://www.genecards.org/cgi-bin/ca
Protein Coding	46	GC10P101354	1.02	https://www.genecards.org/cgi-bin/ca
Protein Coding	41	GC15M079961	1.02	https://www.genecards.org/cgi-bin/ca
Protein Coding	40	GC01M111414	1.02	https://www.genecards.org/cgi-bin/ca
Protein Coding	43	GC0XP018425	1.02	https://www.genecards.org/cgi-bin/ca
Protein Coding	4	GC00U990213	1.02	https://www.genecards.org/cgi-bin/ca
Protein Coding	43	GC20P034546	1.02	https://www.genecards.org/cgi-bin/ca
Protein Coding	47	GC20P059020	1.02	https://www.genecards.org/cgi-bin/ca
Protein Coding	41	GC11P118572	1.02	https://www.genecards.org/cgi-bin/ca
Protein Coding	41	GC17P039980	1.01	https://www.genecards.org/cgi-bin/ca
Protein Coding	41	GC17P032444	1.01	https://www.genecards.org/cgi-bin/ca
Protein Coding	40	GC07P103344	1.01	https://www.genecards.org/cgi-bin/ca
Protein Coding	41	GC0XP115560	1.01	https://www.genecards.org/cgi-bin/ca
Genetic Locus	2	GC11U901278	1.01	https://www.genecards.org/cgi-bin/ca
Protein Coding	42	GC03P048466	1	https://www.genecards.org/cgi-bin/ca
Protein Coding	40	GC0XM108084	1	https://www.genecards.org/cgi-bin/ca
Protein Coding	44	GC09M129094	1	https://www.genecards.org/cgi-bin/ca
Protein Coding	41	GC16M050313	1	https://www.genecards.org/cgi-bin/ca
Protein Coding	41	GC05M031401	1	https://www.genecards.org/cgi-bin/ca
Protein Coding	41	GC11M065901	1	https://www.genecards.org/cgi-bin/ca
Protein Coding	40	GC10P122560	1	https://www.genecards.org/cgi-bin/ca
Protein Coding	41	GC22P039078	0.99	https://www.genecards.org/cgi-bin/ca
Protein Coding	40	GC17M048070	0.99	https://www.genecards.org/cgi-bin/ca
Protein Coding	44	GC03M177019	0.99	https://www.genecards.org/cgi-bin/ca
Protein Coding	43	GC06M145382	0.99	https://www.genecards.org/cgi-bin/ca
Protein Coding	45	GC07P044606	0.98	https://www.genecards.org/cgi-bin/ca
Protein Coding	47	GC19M040843	0.98	https://www.genecards.org/cgi-bin/ca
Protein Coding	36	GC17M008866	0.98	https://www.genecards.org/cgi-bin/ca
Pseudogene	23	GC06M032638	0.98	https://www.genecards.org/cgi-bin/ca
Protein Coding	33	GC13P031200	0.98	https://www.genecards.org/cgi-bin/ca
Protein Coding	39	GC17P006757	0.97	https://www.genecards.org/cgi-bin/ca
Protein Coding	40	GC03P172750	0.97	https://www.genecards.org/cgi-bin/ca
Protein Coding	37	GC03M064501	0.97	https://www.genecards.org/cgi-bin/ca
Protein Coding	43	GC10P083661	0.97	https://www.genecards.org/cgi-bin/ca
Protein Coding	43	GC17P007306	0.97	https://www.genecards.org/cgi-bin/ca
Protein Coding	43	GC01M160995	0.97	https://www.genecards.org/cgi-bin/ca
Protein Coding	43	GC05M134148	0.97	https://www.genecards.org/cgi-bin/ca
Protein Coding	42	GC02M173882	0.97	https://www.genecards.org/cgi-bin/ca
Protein Coding	43	GC19P047290	0.96	https://www.genecards.org/cgi-bin/ca
Protein Coding	42	GC17M044205	0.96	https://www.genecards.org/cgi-bin/ca
Protein Coding	44	GC17P004711	0.95	https://www.genecards.org/cgi-bin/ca
Protein Coding	40	GC12M132822	0.95	https://www.genecards.org/cgi-bin/ca
Protein Coding	37	GC0XP024148	0.95	https://www.genecards.org/cgi-bin/ca
Protein Coding	44	GC04P069051	0.95	https://www.genecards.org/cgi-bin/ca
Protein Coding	37	GC16P026377	0.94	https://www.genecards.org/cgi-bin/ca
Protein Coding	41	GC21M033503	0.94	https://www.genecards.org/cgi-bin/ca
Protein Coding	43	GC11P094128	0.94	https://www.genecards.org/cgi-bin/ca
Protein Coding	42	GC05P055102	0.94	https://www.genecards.org/cgi-bin/ca
Protein Coding	42	GC02P209424	0.94	https://www.genecards.org/cgi-bin/ca
Protein Coding	51	GC10P069269	0.94	https://www.genecards.org/cgi-bin/ca
Protein Coding	29	GC11P018693	0.93	https://www.genecards.org/cgi-bin/ca
Protein Coding	43	GC06M033299	0.93	https://www.genecards.org/cgi-bin/ca

Protein Coding	46	GC11P000660	0.93	https://www.genecards.org/cgi-bin/ca
Protein Coding	44	GC08P011795	0.93	https://www.genecards.org/cgi-bin/ca
Protein Coding	43	GC04M003508	0.93	https://www.genecards.org/cgi-bin/ca
Protein Coding	44	GC14P058244	0.93	https://www.genecards.org/cgi-bin/ca
Protein Coding	44	GC12M047736	0.93	https://www.genecards.org/cgi-bin/ca
Protein Coding	37	GC08P021919	0.93	https://www.genecards.org/cgi-bin/ca
Protein Coding	39	GC01P117060	0.93	https://www.genecards.org/cgi-bin/ca
Protein Coding	44	GC17M029956	0.93	https://www.genecards.org/cgi-bin/ca
Protein Coding	44	GC13M101710	0.92	https://www.genecards.org/cgi-bin/ca
Protein Coding	46	GC11P065711	0.92	https://www.genecards.org/cgi-bin/ca
Protein Coding	36	GC09M127087	0.92	https://www.genecards.org/cgi-bin/ca
RNA Gene	18	GC16M067203	0.92	https://www.genecards.org/cgi-bin/ca
Protein Coding	47	GC12M053210	0.92	https://www.genecards.org/cgi-bin/ca
Protein Coding	44	GC07M117276	0.92	https://www.genecards.org/cgi-bin/ca
Protein Coding	39	GC19M017403	0.92	https://www.genecards.org/cgi-bin/ca
Protein Coding	32	GC02M089142	0.92	https://www.genecards.org/cgi-bin/ca
Protein Coding	40	GC01P207105	0.92	https://www.genecards.org/cgi-bin/ca
Protein Coding	37	GC0XP149688	0.91	https://www.genecards.org/cgi-bin/ca
Protein Coding	45	GC04P025657	0.91	https://www.genecards.org/cgi-bin/ca
Protein Coding	50	GC01P070411	0.91	https://www.genecards.org/cgi-bin/ca
Protein Coding	39	GC04P119135	0.91	https://www.genecards.org/cgi-bin/ca
Protein Coding	40	GC10P027504	0.91	https://www.genecards.org/cgi-bin/ca
Protein Coding	44	GC01P161524	0.91	https://www.genecards.org/cgi-bin/ca
Protein Coding	42	GC01M171604	0.91	https://www.genecards.org/cgi-bin/ca
Protein Coding	44	GC10P126905	0.9	https://www.genecards.org/cgi-bin/ca
Protein Coding	43	GC20M001978	0.9	https://www.genecards.org/cgi-bin/ca
Protein Coding	43	GC01M202696	0.9	https://www.genecards.org/cgi-bin/ca
Protein Coding	47	GC12P062260	0.9	https://www.genecards.org/cgi-bin/ca
Protein Coding	43	GC01P160370	0.9	https://www.genecards.org/cgi-bin/ca
Protein Coding	38	GC07M101162	0.9	https://www.genecards.org/cgi-bin/ca
Protein Coding	48	GC08M117798	0.9	https://www.genecards.org/cgi-bin/ca
Protein Coding	36	GC19P055437	0.9	https://www.genecards.org/cgi-bin/ca
Protein Coding	44	GC08P018179	0.89	https://www.genecards.org/cgi-bin/ca
Protein Coding	42	GC12P120469	0.89	https://www.genecards.org/cgi-bin/ca
Protein Coding	43	GC19P050861	0.88	https://www.genecards.org/cgi-bin/ca
Protein Coding	44	GC03M139355	0.88	https://www.genecards.org/cgi-bin/ca
Protein Coding	44	GC20M062136	0.88	https://www.genecards.org/cgi-bin/ca
Protein Coding	40	GC02P197501	0.88	https://www.genecards.org/cgi-bin/ca
Protein Coding	45	GC19M006496	0.88	https://www.genecards.org/cgi-bin/ca
Protein Coding	40	GC11M061109	0.87	https://www.genecards.org/cgi-bin/ca
Protein Coding	47	GC17P042609	0.87	https://www.genecards.org/cgi-bin/ca
Protein Coding	41	GC10P035126	0.87	https://www.genecards.org/cgi-bin/ca
Protein Coding	37	GC19M018843	0.87	https://www.genecards.org/cgi-bin/ca
Protein Coding	41	GC05P079111	0.87	https://www.genecards.org/cgi-bin/ca
Protein Coding	42	GC07P044999	0.87	https://www.genecards.org/cgi-bin/ca
Biological Regior	1	GC19P002253	0.87	https://www.genecards.org/cgi-bin/ca
Protein Coding	45	GC12M114670	0.87	https://www.genecards.org/cgi-bin/ca
Protein Coding	40	GC06M018224	0.87	https://www.genecards.org/cgi-bin/ca
Protein Coding	48	GC21P033229	0.87	https://www.genecards.org/cgi-bin/ca
Protein Coding	42	GC07M042916	0.87	https://www.genecards.org/cgi-bin/ca
Protein Coding	33	GC06P047307	0.86	https://www.genecards.org/cgi-bin/ca
Protein Coding	41	GC12M057530	0.86	https://www.genecards.org/cgi-bin/ca
Protein Coding	44	GC0XM108155	0.86	https://www.genecards.org/cgi-bin/ca
Protein Coding	38	GC14M094526	0.85	https://www.genecards.org/cgi-bin/ca

Protein Coding	41	GC05P052788	0.85	https://www.genecards.org/cgi-bin/ca
Protein Coding	43	GC15M075674	0.85	https://www.genecards.org/cgi-bin/ca
Protein Coding	40	GC01M205302	0.85	https://www.genecards.org/cgi-bin/ca
Protein Coding	39	GC16M020309	0.85	https://www.genecards.org/cgi-bin/ca
Protein Coding	45	GC19M033386	0.85	https://www.genecards.org/cgi-bin/ca
Protein Coding	41	GC07P020329	0.84	https://www.genecards.org/cgi-bin/ca
Protein Coding	44	GC05P071587	0.84	https://www.genecards.org/cgi-bin/ca
Protein Coding	39	GC03M185914	0.84	https://www.genecards.org/cgi-bin/ca
Protein Coding	43	GC12M124324	0.84	https://www.genecards.org/cgi-bin/ca
Protein Coding	48	GC17P050561	0.84	https://www.genecards.org/cgi-bin/ca
Protein Coding	42	GC14M021521	0.84	https://www.genecards.org/cgi-bin/ca
Protein Coding	40	GC03P052681	0.84	https://www.genecards.org/cgi-bin/ca
Protein Coding	37	GC14P021246	0.84	https://www.genecards.org/cgi-bin/ca
Protein Coding	44	GC16M001790	0.84	https://www.genecards.org/cgi-bin/ca
Protein Coding	47	GC17P060149	0.84	https://www.genecards.org/cgi-bin/ca
Protein Coding	47	GC09M098288	0.83	https://www.genecards.org/cgi-bin/ca
Protein Coding	41	GC12M046358	0.83	https://www.genecards.org/cgi-bin/ca
Protein Coding	40	GC01P053891	0.83	https://www.genecards.org/cgi-bin/ca
Protein Coding	40	GC17M048575	0.82	https://www.genecards.org/cgi-bin/ca
Protein Coding	49	GC11P065198	0.82	https://www.genecards.org/cgi-bin/ca
Protein Coding	48	GC20P044966	0.81	https://www.genecards.org/cgi-bin/ca
Protein Coding	39	GC05P078360	0.81	https://www.genecards.org/cgi-bin/ca
Protein Coding	50	GC05P036606	0.81	https://www.genecards.org/cgi-bin/ca
Protein Coding	44	GC09M033100	0.81	https://www.genecards.org/cgi-bin/ca
Protein Coding	45	GC17M040624	0.81	https://www.genecards.org/cgi-bin/ca
Protein Coding	36	GC11M077878	0.81	https://www.genecards.org/cgi-bin/ca
Protein Coding	44	GC19P002164	0.81	https://www.genecards.org/cgi-bin/ca
Protein Coding	43	GC12P122847	0.81	https://www.genecards.org/cgi-bin/ca
Protein Coding	47	GC09P128684	0.81	https://www.genecards.org/cgi-bin/ca
Protein Coding	34	GC17P038297	0.8	https://www.genecards.org/cgi-bin/ca
Protein Coding	42	GC09M120388	0.8	https://www.genecards.org/cgi-bin/ca
Protein Coding	37	GC04M122888	0.8	https://www.genecards.org/cgi-bin/ca
Protein Coding	46	GC14P020343	0.8	https://www.genecards.org/cgi-bin/ca
Protein Coding	42	GC0XP001336	0.79	https://www.genecards.org/cgi-bin/ca
Protein Coding	42	GC01M156840	0.79	https://www.genecards.org/cgi-bin/ca
Protein Coding	47	GC12M093406	0.79	https://www.genecards.org/cgi-bin/ca
Protein Coding	41	GC17P038752	0.79	https://www.genecards.org/cgi-bin/ca
Protein Coding	45	GC05P096525	0.79	https://www.genecards.org/cgi-bin/ca
Protein Coding	43	GC06M170535	0.79	https://www.genecards.org/cgi-bin/ca
Protein Coding	38	GC14M059595	0.79	https://www.genecards.org/cgi-bin/ca
Protein Coding	39	GC02P172084	0.79	https://www.genecards.org/cgi-bin/ca
Protein Coding	36	GC08M123013	0.79	https://www.genecards.org/cgi-bin/ca
Protein Coding	41	GC0XM019552	0.79	https://www.genecards.org/cgi-bin/ca
Protein Coding	45	GC17P061399	0.79	https://www.genecards.org/cgi-bin/ca
Protein Coding	39	GC19P043353	0.79	https://www.genecards.org/cgi-bin/ca
Protein Coding	43	GC06M073515	0.78	https://www.genecards.org/cgi-bin/ca
Protein Coding	41	GC19P045467	0.78	https://www.genecards.org/cgi-bin/ca
Protein Coding	46	GC19M012796	0.78	https://www.genecards.org/cgi-bin/ca
Protein Coding	47	GC02M160099	0.78	https://www.genecards.org/cgi-bin/ca
Protein Coding	44	GC05M001050	0.77	https://www.genecards.org/cgi-bin/ca
Protein Coding	44	GC21P036134	0.77	https://www.genecards.org/cgi-bin/ca
Protein Coding	39	GC06P047497	0.77	https://www.genecards.org/cgi-bin/ca
Protein Coding	45	GC03M050299	0.77	https://www.genecards.org/cgi-bin/ca
Protein Coding	42	GC06M031727	0.77	https://www.genecards.org/cgi-bin/ca

Protein Coding	37	GC19P054906	0.77	https://www.genecards.org/cgi-bin/ca
Protein Coding	40	GC10P000134	0.76	https://www.genecards.org/cgi-bin/ca
Protein Coding	39	GC17M048591	0.76	https://www.genecards.org/cgi-bin/ca
Protein Coding	37	GC0XP017393	0.76	https://www.genecards.org/cgi-bin/ca
Protein Coding	48	GC16P050742	0.76	https://www.genecards.org/cgi-bin/ca
Protein Coding	41	GC02P070087	0.76	https://www.genecards.org/cgi-bin/ca
Protein Coding	44	GC11P108008	0.75	https://www.genecards.org/cgi-bin/ca
Protein Coding	39	GC14P055559	0.75	https://www.genecards.org/cgi-bin/ca
Protein Coding	46	GC16P021963	0.75	https://www.genecards.org/cgi-bin/ca
Protein Coding	41	GC09M133471	0.75	https://www.genecards.org/cgi-bin/ca
Protein Coding	47	GC04P009783	0.75	https://www.genecards.org/cgi-bin/ca
Protein Coding	41	GC06P047337	0.75	https://www.genecards.org/cgi-bin/ca
Protein Coding	36	GC19M039385	0.74	https://www.genecards.org/cgi-bin/ca
Protein Coding	41	GC17P008039	0.74	https://www.genecards.org/cgi-bin/ca
Protein Coding	40	GC11M057386	0.74	https://www.genecards.org/cgi-bin/ca
Protein Coding	39	GC09M092383	0.74	https://www.genecards.org/cgi-bin/ca
Protein Coding	36	GC01M085729	0.74	https://www.genecards.org/cgi-bin/ca
Protein Coding	41	GC01P109668	0.73	https://www.genecards.org/cgi-bin/ca
Protein Coding	33	GC15P040595	0.73	https://www.genecards.org/cgi-bin/ca
Protein Coding	41	GC06M117560	0.73	https://www.genecards.org/cgi-bin/ca
Protein Coding	45	GC06M042217	0.73	https://www.genecards.org/cgi-bin/ca
Protein Coding	48	GC16P055656	0.73	https://www.genecards.org/cgi-bin/ca
Protein Coding	47	GC01P020634	0.73	https://www.genecards.org/cgi-bin/ca
Protein Coding	39	GC11P066546	0.73	https://www.genecards.org/cgi-bin/ca
Protein Coding	43	GC15P058138	0.73	https://www.genecards.org/cgi-bin/ca
Protein Coding	43	GC08M096142	0.72	https://www.genecards.org/cgi-bin/ca
Protein Coding	41	GC10P122374	0.72	https://www.genecards.org/cgi-bin/ca
Protein Coding	48	GC20M063343	0.72	https://www.genecards.org/cgi-bin/ca
Protein Coding	37	GC02P115304	0.72	https://www.genecards.org/cgi-bin/ca
Protein Coding	33	GC17P067717	0.72	https://www.genecards.org/cgi-bin/ca
Protein Coding	47	GC05P074640	0.72	https://www.genecards.org/cgi-bin/ca
Protein Coding	48	GC05M180234	0.72	https://www.genecards.org/cgi-bin/ca
Protein Coding	41	GC19P001438	0.72	https://www.genecards.org/cgi-bin/ca
Protein Coding	48	GC07M093424	0.72	https://www.genecards.org/cgi-bin/ca
Protein Coding	48	GC16P030064	0.71	https://www.genecards.org/cgi-bin/ca
Protein Coding	41	GC04M069484	0.71	https://www.genecards.org/cgi-bin/ca
Protein Coding	40	GC01M145921	0.71	https://www.genecards.org/cgi-bin/ca
Protein Coding	41	GC09M133097	0.71	https://www.genecards.org/cgi-bin/ca
Protein Coding	40	GC11P071454	0.71	https://www.genecards.org/cgi-bin/ca
Protein Coding	45	GC18M059327	0.71	https://www.genecards.org/cgi-bin/ca
Protein Coding	44	GC17M004630	0.71	https://www.genecards.org/cgi-bin/ca
Protein Coding	40	GC19P044890	0.7	https://www.genecards.org/cgi-bin/ca
Protein Coding	39	GC01P179882	0.7	https://www.genecards.org/cgi-bin/ca
Protein Coding	45	GC15P031923	0.7	https://www.genecards.org/cgi-bin/ca
Protein Coding	46	GC20P046021	0.7	https://www.genecards.org/cgi-bin/ca
Protein Coding	43	GC11M057233	0.7	https://www.genecards.org/cgi-bin/ca
Protein Coding	44	GC10P006144	0.7	https://www.genecards.org/cgi-bin/ca
Protein Coding	35	GC01M120150	0.69	https://www.genecards.org/cgi-bin/ca
Protein Coding	40	GC19P006737	0.69	https://www.genecards.org/cgi-bin/ca
Protein Coding	46	GC11M104942	0.69	https://www.genecards.org/cgi-bin/ca
Protein Coding	38	GC05M157785	0.69	https://www.genecards.org/cgi-bin/ca
Protein Coding	42	GC0XP153793	0.69	https://www.genecards.org/cgi-bin/ca
Protein Coding	48	GC10P114044	0.69	https://www.genecards.org/cgi-bin/ca
Protein Coding	31	GC01M040861	0.69	https://www.genecards.org/cgi-bin/ca

Protein Coding	41	GC12M048688	0.69	https://www.genecards.org/cgi-bin/ca
Protein Coding	40	GC12P055743	0.69	https://www.genecards.org/cgi-bin/ca
Protein Coding	40	GC22P040679	0.69	https://www.genecards.org/cgi-bin/ca
Protein Coding	37	GC11P113387	0.69	https://www.genecards.org/cgi-bin/ca
Protein Coding	41	GC14M021210	0.69	https://www.genecards.org/cgi-bin/ca
Protein Coding	48	GC12P043758	0.69	https://www.genecards.org/cgi-bin/ca
Protein Coding	41	GC12M120196	0.68	https://www.genecards.org/cgi-bin/ca
Protein Coding	45	GC08P144095	0.68	https://www.genecards.org/cgi-bin/ca
Protein Coding	40	GC06P047325	0.68	https://www.genecards.org/cgi-bin/ca
Protein Coding	37	GC06M049727	0.68	https://www.genecards.org/cgi-bin/ca
Protein Coding	41	GC10M005943	0.68	https://www.genecards.org/cgi-bin/ca
Protein Coding	32	GC11M107326	0.68	https://www.genecards.org/cgi-bin/ca
RNA Gene	18	GC01P093846	0.67	https://www.genecards.org/cgi-bin/ca
Protein Coding	40	GC17P002054	0.67	https://www.genecards.org/cgi-bin/ca
Protein Coding	41	GC10P116590	0.67	https://www.genecards.org/cgi-bin/ca
Protein Coding	41	GC02P230415	0.66	https://www.genecards.org/cgi-bin/ca
Protein Coding	42	GC11M017179	0.66	https://www.genecards.org/cgi-bin/ca
Biological Regior	1	GC08P038150	0.66	https://www.genecards.org/cgi-bin/ca
Protein Coding	40	GC16M003014	0.66	https://www.genecards.org/cgi-bin/ca
Protein Coding	41	GC10M034110	0.66	https://www.genecards.org/cgi-bin/ca
Protein Coding	38	GC19P039480	0.65	https://www.genecards.org/cgi-bin/ca
Protein Coding	44	GC01P062436	0.65	https://www.genecards.org/cgi-bin/ca
Protein Coding	46	GC15P072880	0.65	https://www.genecards.org/cgi-bin/ca
Protein Coding	44	GC13P113584	0.65	https://www.genecards.org/cgi-bin/ca
Protein Coding	40	GC12P112938	0.65	https://www.genecards.org/cgi-bin/ca
Protein Coding	43	GC14P024161	0.65	https://www.genecards.org/cgi-bin/ca
Protein Coding	44	GC10P093073	0.65	https://www.genecards.org/cgi-bin/ca
Protein Coding	36	GC08P027633	0.65	https://www.genecards.org/cgi-bin/ca
RNA Gene	18	GC14M023388	0.65	https://www.genecards.org/cgi-bin/ca
Protein Coding	44	GC15P042491	0.65	https://www.genecards.org/cgi-bin/ca
Protein Coding	45	GC12M009067	0.65	https://www.genecards.org/cgi-bin/ca
Protein Coding	38	GC06P127118	0.65	https://www.genecards.org/cgi-bin/ca
Protein Coding	37	GC16M067389	0.64	https://www.genecards.org/cgi-bin/ca
Protein Coding	44	GC14P023953	0.64	https://www.genecards.org/cgi-bin/ca
Protein Coding	39	GC16M066925	0.64	https://www.genecards.org/cgi-bin/ca
Protein Coding	14	GC07P145138	0.64	https://www.genecards.org/cgi-bin/ca
Protein Coding	42	GC20P002276	0.64	https://www.genecards.org/cgi-bin/ca
Protein Coding	41	GC19M010335	0.64	https://www.genecards.org/cgi-bin/ca
Protein Coding	45	GC01M023870	0.64	https://www.genecards.org/cgi-bin/ca
Protein Coding	37	GC12P057452	0.64	https://www.genecards.org/cgi-bin/ca
Protein Coding	46	GC06P024494	0.64	https://www.genecards.org/cgi-bin/ca
Protein Coding	42	GC05P040909	0.63	https://www.genecards.org/cgi-bin/ca
Protein Coding	42	GC14P090256	0.63	https://www.genecards.org/cgi-bin/ca
Protein Coding	42	GC17M043640	0.63	https://www.genecards.org/cgi-bin/ca
Protein Coding	41	GC05M151029	0.63	https://www.genecards.org/cgi-bin/ca
Protein Coding	47	GC16M058157	0.63	https://www.genecards.org/cgi-bin/ca
Protein Coding	43	GC01P171248	0.63	https://www.genecards.org/cgi-bin/ca
Protein Coding	44	GC04P158315	0.63	https://www.genecards.org/cgi-bin/ca
Protein Coding	30	GC09M034516	0.63	https://www.genecards.org/cgi-bin/ca
Protein Coding	40	GC01M180972	0.63	https://www.genecards.org/cgi-bin/ca
Protein Coding	36	GC03P119173	0.62	https://www.genecards.org/cgi-bin/ca
Protein Coding	39	GC09P136658	0.62	https://www.genecards.org/cgi-bin/ca
Protein Coding	41	GC11P001852	0.62	https://www.genecards.org/cgi-bin/ca
Protein Coding	29	GC19P055464	0.62	https://www.genecards.org/cgi-bin/ca

Protein Coding	44	GC02P200585	0.62	https://www.genecards.org/cgi-bin/ca
Protein Coding	45	GC03P136862	0.62	https://www.genecards.org/cgi-bin/ca
Protein Coding	41	GC19P018563	0.62	https://www.genecards.org/cgi-bin/ca
Protein Coding	44	GC12P120439	0.62	https://www.genecards.org/cgi-bin/ca
Protein Coding	47	GC10M073812	0.62	https://www.genecards.org/cgi-bin/ca
Protein Coding	44	GC02P115301	0.62	https://www.genecards.org/cgi-bin/ca
Protein Coding	39	GC17P034319	0.61	https://www.genecards.org/cgi-bin/ca
Protein Coding	40	GC03P184361	0.61	https://www.genecards.org/cgi-bin/ca
Protein Coding	42	GC14M067647	0.61	https://www.genecards.org/cgi-bin/ca
Protein Coding	52	GC07M151053	0.61	https://www.genecards.org/cgi-bin/ca
Protein Coding	40	GC06M032632	0.61	https://www.genecards.org/cgi-bin/ca
Protein Coding	44	GC01M119747	0.61	https://www.genecards.org/cgi-bin/ca
Protein Coding	43	GC02P233671	0.61	https://www.genecards.org/cgi-bin/ca
Protein Coding	39	GC04M186587	0.61	https://www.genecards.org/cgi-bin/ca
Protein Coding	43	GC17P046924	0.6	https://www.genecards.org/cgi-bin/ca
Protein Coding	46	GC03M048744	0.6	https://www.genecards.org/cgi-bin/ca
Protein Coding	37	GC20M003783	0.6	https://www.genecards.org/cgi-bin/ca
Protein Coding	45	GC03P130850	0.6	https://www.genecards.org/cgi-bin/ca
Protein Coding	39	GC13P097953	0.6	https://www.genecards.org/cgi-bin/ca
Protein Coding	42	GC02M043850	0.59	https://www.genecards.org/cgi-bin/ca
Protein Coding	42	GC02M055634	0.59	https://www.genecards.org/cgi-bin/ca
Protein Coding	45	GC06M052264	0.59	https://www.genecards.org/cgi-bin/ca
Gene Cluster	4	GC09U990039	0.59	https://www.genecards.org/cgi-bin/ca
Protein Coding	41	GC12P045729	0.58	https://www.genecards.org/cgi-bin/ca
Protein Coding	45	GC03P186783	0.58	https://www.genecards.org/cgi-bin/ca
Protein Coding	42	GC11M033858	0.58	https://www.genecards.org/cgi-bin/ca
Protein Coding	43	GC06P043076	0.58	https://www.genecards.org/cgi-bin/ca
Protein Coding	38	GC22P050738	0.58	https://www.genecards.org/cgi-bin/ca
Protein Coding	38	GC20M000435	0.58	https://www.genecards.org/cgi-bin/ca
Protein Coding	42	GC05P062306	0.58	https://www.genecards.org/cgi-bin/ca
Protein Coding	41	GC09M035687	0.58	https://www.genecards.org/cgi-bin/ca
Protein Coding	45	GC12M063142	0.58	https://www.genecards.org/cgi-bin/ca
Protein Coding	39	GC03M012834	0.57	https://www.genecards.org/cgi-bin/ca
Protein Coding	46	GC12M096000	0.57	https://www.genecards.org/cgi-bin/ca
Protein Coding	36	GC01P066533	0.57	https://www.genecards.org/cgi-bin/ca
Protein Coding	32	GC02P225400	0.57	https://www.genecards.org/cgi-bin/ca
Protein Coding	41	GC11P058622	0.56	https://www.genecards.org/cgi-bin/ca
Protein Coding	44	GC02M027201	0.56	https://www.genecards.org/cgi-bin/ca
Protein Coding	47	GC05M161288	0.56	https://www.genecards.org/cgi-bin/ca
Protein Coding	40	GC14P099737	0.56	https://www.genecards.org/cgi-bin/ca
Protein Coding	41	GC03P186660	0.56	https://www.genecards.org/cgi-bin/ca
Protein Coding	45	GC02P233059	0.56	https://www.genecards.org/cgi-bin/ca
Protein Coding	41	GC12M068248	0.55	https://www.genecards.org/cgi-bin/ca
Protein Coding	40	GC12M121057	0.55	https://www.genecards.org/cgi-bin/ca
Protein Coding	38	GC10P102152	0.55	https://www.genecards.org/cgi-bin/ca
Protein Coding	43	GC05M180841	0.55	https://www.genecards.org/cgi-bin/ca
Protein Coding	47	GC11M088504	0.55	https://www.genecards.org/cgi-bin/ca
Protein Coding	39	GC11M061253	0.55	https://www.genecards.org/cgi-bin/ca
Biological Region	1	GC01P119415	0.54	https://www.genecards.org/cgi-bin/ca
Protein Coding	43	GC16M067154	0.54	https://www.genecards.org/cgi-bin/ca
Protein Coding	37	GC05M000866	0.54	https://www.genecards.org/cgi-bin/ca
Protein Coding	41	GC02M074532	0.54	https://www.genecards.org/cgi-bin/ca
Protein Coding	44	GC15P078565	0.54	https://www.genecards.org/cgi-bin/ca
Protein Coding	44	GC02M054934	0.53	https://www.genecards.org/cgi-bin/ca

Protein Coding	50	GC14M068874	0.53	https://www.genecards.org/cgi-bin/ca
Protein Coding	36	GC06M129576	0.53	https://www.genecards.org/cgi-bin/ca
Protein Coding	47	GC13P048653	0.52	https://www.genecards.org/cgi-bin/ca
Protein Coding	35	GC0XM119538	0.52	https://www.genecards.org/cgi-bin/ca
Protein Coding	45	GC12P015366	0.51	https://www.genecards.org/cgi-bin/ca
Protein Coding	37	GC17M081887	0.51	https://www.genecards.org/cgi-bin/ca
Protein Coding	40	GC05M043356	0.5	https://www.genecards.org/cgi-bin/ca
Protein Coding	47	GC16P004474	0.5	https://www.genecards.org/cgi-bin/ca
Protein Coding	41	GC16M048166	0.5	https://www.genecards.org/cgi-bin/ca
Protein Coding	40	GC12M051093	0.49	https://www.genecards.org/cgi-bin/ca
Protein Coding	42	GC05P109689	0.49	https://www.genecards.org/cgi-bin/ca
Protein Coding	37	GC12P056189	0.49	https://www.genecards.org/cgi-bin/ca
Protein Coding	36	GC05M073444	0.49	https://www.genecards.org/cgi-bin/ca
Protein Coding	43	GC15M078624	0.49	https://www.genecards.org/cgi-bin/ca
Protein Coding	51	GC05P157158	0.49	https://www.genecards.org/cgi-bin/ca
Protein Coding	43	GC17M064542	0.49	https://www.genecards.org/cgi-bin/ca
Protein Coding	39	GC22P020496	0.48	https://www.genecards.org/cgi-bin/ca
Protein Coding	37	GC02M073640	0.48	https://www.genecards.org/cgi-bin/ca
Protein Coding	42	GC11P077128	0.47	https://www.genecards.org/cgi-bin/ca
Protein Coding	35	GC08M008783	0.46	https://www.genecards.org/cgi-bin/ca
Protein Coding	40	GC18M061905	0.46	https://www.genecards.org/cgi-bin/ca
Protein Coding	39	GC02M172099	0.46	https://www.genecards.org/cgi-bin/ca
Protein Coding	45	GC0XP153688	0.46	https://www.genecards.org/cgi-bin/ca
Protein Coding	42	GC07P155070	0.46	https://www.genecards.org/cgi-bin/ca
RNA Gene	17	GC05M059703	0.45	https://www.genecards.org/cgi-bin/ca
Protein Coding	43	GC19P055408	0.45	https://www.genecards.org/cgi-bin/ca
Protein Coding	47	GC17M042113	0.45	https://www.genecards.org/cgi-bin/ca
Protein Coding	45	GC12M047782	0.45	https://www.genecards.org/cgi-bin/ca
Protein Coding	43	GC20P000407	0.44	https://www.genecards.org/cgi-bin/ca
Protein Coding	40	GC16P004425	0.44	https://www.genecards.org/cgi-bin/ca
Protein Coding	41	GC01M225887	0.44	https://www.genecards.org/cgi-bin/ca
Protein Coding	31	GC12M112160	0.44	https://www.genecards.org/cgi-bin/ca
Protein Coding	40	GC01P240014	0.44	https://www.genecards.org/cgi-bin/ca
Protein Coding	36	GC08P030095	0.44	https://www.genecards.org/cgi-bin/ca
Protein Coding	50	GC02P111898	0.44	https://www.genecards.org/cgi-bin/ca
Protein Coding	41	GC14P024136	0.43	https://www.genecards.org/cgi-bin/ca
Protein Coding	46	GC11M061160	0.43	https://www.genecards.org/cgi-bin/ca
Protein Coding	28	GC14P051992	0.42	https://www.genecards.org/cgi-bin/ca
Protein Coding	43	GC09P128818	0.42	https://www.genecards.org/cgi-bin/ca
Protein Coding	40	GC03M052708	0.42	https://www.genecards.org/cgi-bin/ca
Protein Coding	40	GC07P027739	0.42	https://www.genecards.org/cgi-bin/ca
Protein Coding	36	GC08M025841	0.42	https://www.genecards.org/cgi-bin/ca
Protein Coding	41	GC12M007656	0.42	https://www.genecards.org/cgi-bin/ca
Protein Coding	37	GC10P047300	0.42	https://www.genecards.org/cgi-bin/ca
Protein Coding	45	GC02M096112	0.41	https://www.genecards.org/cgi-bin/ca
Protein Coding	37	GC01M008004	0.41	https://www.genecards.org/cgi-bin/ca
Protein Coding	43	GC01M035176	0.41	https://www.genecards.org/cgi-bin/ca
Protein Coding	43	GC19P001040	0.41	https://www.genecards.org/cgi-bin/ca
Protein Coding	41	GC11P045986	0.4	https://www.genecards.org/cgi-bin/ca
Protein Coding	29	GC04P190173	0.4	https://www.genecards.org/cgi-bin/ca
Protein Coding	42	GC07M010938	0.4	https://www.genecards.org/cgi-bin/ca
Protein Coding	40	GC15M075463	0.4	https://www.genecards.org/cgi-bin/ca
Protein Coding	44	GC09P038392	0.39	https://www.genecards.org/cgi-bin/ca
Protein Coding	42	GC08M042752	0.39	https://www.genecards.org/cgi-bin/ca

Protein Coding	42	GC04P040337	0.39 https://www.genecards.org/cgi-bin/ca
Protein Coding	41	GC06M055728	0.39 https://www.genecards.org/cgi-bin/ca
Protein Coding	41	GC08P042697	0.39 https://www.genecards.org/cgi-bin/ca
Protein Coding	40	GC03P183253	0.39 https://www.genecards.org/cgi-bin/ca
Protein Coding	40	GC06M133987	0.39 https://www.genecards.org/cgi-bin/ca
Protein Coding	39	GC01M039757	0.39 https://www.genecards.org/cgi-bin/ca
Protein Coding	39	GC02P020666	0.39 https://www.genecards.org/cgi-bin/ca
Protein Coding	39	GC20P054476	0.39 https://www.genecards.org/cgi-bin/ca
Protein Coding	38	GC11M003666	0.39 https://www.genecards.org/cgi-bin/ca
Protein Coding	38	GC22P025565	0.39 https://www.genecards.org/cgi-bin/ca
Protein Coding	37	GC06P012009	0.39 https://www.genecards.org/cgi-bin/ca
Protein Coding	36	GC12P026939	0.39 https://www.genecards.org/cgi-bin/ca
Protein Coding	33	GC19P056595	0.39 https://www.genecards.org/cgi-bin/ca
Protein Coding	29	GC06M081495	0.39 https://www.genecards.org/cgi-bin/ca
Protein Coding	24	GC12M025108	0.39 https://www.genecards.org/cgi-bin/ca
Protein Coding	45	GC0XM053431	0.39 https://www.genecards.org/cgi-bin/ca
Protein Coding	39	GC02M200908	0.39 https://www.genecards.org/cgi-bin/ca
Protein Coding	39	GC18P021612	0.39 https://www.genecards.org/cgi-bin/ca
Protein Coding	41	GC16P069424	0.39 https://www.genecards.org/cgi-bin/ca
Protein Coding	40	GC11M062806	0.39 https://www.genecards.org/cgi-bin/ca
Protein Coding	39	GC17M009250	0.39 https://www.genecards.org/cgi-bin/ca
Protein Coding	36	GC20M005069	0.39 https://www.genecards.org/cgi-bin/ca
Protein Coding	35	GC01P009588	0.39 https://www.genecards.org/cgi-bin/ca
Protein Coding	26	GC16M031495	0.39 https://www.genecards.org/cgi-bin/ca
Protein Coding	45	GC14M090847	0.38 https://www.genecards.org/cgi-bin/ca
Protein Coding	44	GC12M056161	0.38 https://www.genecards.org/cgi-bin/ca
Protein Coding	41	GC14M081471	0.38 https://www.genecards.org/cgi-bin/ca
Protein Coding	34	GC19P035545	0.38 https://www.genecards.org/cgi-bin/ca
Protein Coding	48	GC11M067415	0.38 https://www.genecards.org/cgi-bin/ca
Protein Coding	41	GC12P053006	0.37 https://www.genecards.org/cgi-bin/ca
Uncategorized	9	GC02P233586	0.37 https://www.genecards.org/cgi-bin/ca
Protein Coding	36	GC13P027427	0.37 https://www.genecards.org/cgi-bin/ca
Protein Coding	48	GC17P044557	0.37 https://www.genecards.org/cgi-bin/ca
Protein Coding	43	GC03M049277	0.37 https://www.genecards.org/cgi-bin/ca
Protein Coding	40	GC11M065122	0.37 https://www.genecards.org/cgi-bin/ca
Protein Coding	39	GC12P045215	0.37 https://www.genecards.org/cgi-bin/ca
Protein Coding	39	GC01P032108	0.37 https://www.genecards.org/cgi-bin/ca
Protein Coding	39	GC09P097633	0.36 https://www.genecards.org/cgi-bin/ca
Protein Coding	39	GC09P121566	0.36 https://www.genecards.org/cgi-bin/ca
Protein Coding	40	GC19M038251	0.36 https://www.genecards.org/cgi-bin/ca
Protein Coding	44	GC0XP071283	0.36 https://www.genecards.org/cgi-bin/ca
Protein Coding	38	GC07M002582	0.35 https://www.genecards.org/cgi-bin/ca
Protein Coding	39	GC16P004241	0.35 https://www.genecards.org/cgi-bin/ca
Protein Coding	45	GC04P003766	0.34 https://www.genecards.org/cgi-bin/ca
Protein Coding	47	GC19P000532	0.34 https://www.genecards.org/cgi-bin/ca
Protein Coding	37	GC15M049106	0.33 https://www.genecards.org/cgi-bin/ca
Protein Coding	40	GC08M016992	0.33 https://www.genecards.org/cgi-bin/ca
Protein Coding	37	GC13M095801	0.32 https://www.genecards.org/cgi-bin/ca
Protein Coding	36	GC01P152984	0.32 https://www.genecards.org/cgi-bin/ca
Protein Coding	39	GC04M144831	0.32 https://www.genecards.org/cgi-bin/ca
Protein Coding	42	GC03M122421	0.31 https://www.genecards.org/cgi-bin/ca
Protein Coding	43	GC11M085957	0.29 https://www.genecards.org/cgi-bin/ca
Protein Coding	33	GC01P151129	0.29 https://www.genecards.org/cgi-bin/ca
Protein Coding	43	GC17M004937	0.29 https://www.genecards.org/cgi-bin/ca

Protein Coding	44	GC11P117144	0.28	https://www.genecards.org/cgi-bin/ca
Protein Coding	41	GC06M013621	0.28	https://www.genecards.org/cgi-bin/ca
Protein Coding	48	GC22M050263	0.28	https://www.genecards.org/cgi-bin/ca
Protein Coding	39	GC11P005689	0.27	https://www.genecards.org/cgi-bin/ca
Protein Coding	41	GC22P039350	0.27	https://www.genecards.org/cgi-bin/ca
Protein Coding	41	GC22M050059	0.27	https://www.genecards.org/cgi-bin/ca
Protein Coding	38	GC04P075724	0.27	https://www.genecards.org/cgi-bin/ca
Protein Coding	39	GC20M017613	0.24	https://www.genecards.org/cgi-bin/ca
Protein Coding	35	GC01M019215	0.24	https://www.genecards.org/cgi-bin/ca
Protein Coding	43	GC13M049699	0.23	https://www.genecards.org/cgi-bin/ca
Protein Coding	37	GC07M139044	0.22	https://www.genecards.org/cgi-bin/ca
Protein Coding	42	GC01M088853	0.21	https://www.genecards.org/cgi-bin/ca
Protein Coding	42	GC10M035046	0.21	https://www.genecards.org/cgi-bin/ca
Protein Coding	40	GC12P057693	0.21	https://www.genecards.org/cgi-bin/ca
Protein Coding	42	GC03P005187	0.2	https://www.genecards.org/cgi-bin/ca
Protein Coding	42	GC05M170246	0.2	https://www.genecards.org/cgi-bin/ca
Protein Coding	42	GC0XP038561	0.2	https://www.genecards.org/cgi-bin/ca
Biological Regior	1	GC09P027577	0.2	https://www.genecards.org/cgi-bin/ca
Protein Coding	40	GC18M050266	0.2	https://www.genecards.org/cgi-bin/ca
Protein Coding	41	GC01P111755	0.18	https://www.genecards.org/cgi-bin/ca
Protein Coding	36	GC13P049995	0.18	https://www.genecards.org/cgi-bin/ca
Protein Coding	35	GC10M117277	0.18	https://www.genecards.org/cgi-bin/ca
Genetic Locus	6	GC19U990001	0.18	https://www.genecards.org/cgi-bin/ca

ırddisp.pl?gene=BRCA2
ırddisp.pl?gene=BRCA1
ırddisp.pl?gene=NR5A1
ırddisp.pl?gene=POF1B
ırddisp.pl?gene=TP53
ırddisp.pl?gene=BMP15
ırddisp.pl?gene=FOXL2
ırddisp.pl?gene=FSHR
ırddisp.pl?gene=NOBOX
ırddisp.pl?gene=INS
ırddisp.pl?gene=PTEN
ırddisp.pl?gene=FMR1
ırddisp.pl?gene=NBN
ırddisp.pl?gene=STAG3
ırddisp.pl?gene=POMC
ırddisp.pl?gene=FIGLA
ırddisp.pl?gene=CYP11A1
ırddisp.pl?gene=AKT1
ırddisp.pl?gene=ERCC6
ırddisp.pl?gene=IL6
ırddisp.pl?gene=NR0B1
ırddisp.pl?gene=ESR1
ırddisp.pl?gene=TP63
ırddisp.pl?gene=LMNA
ırddisp.pl?gene=GDF9
ırddisp.pl?gene=CYP19A1
ırddisp.pl?gene=WT1
ırddisp.pl?gene=CTNNB1
ırddisp.pl?gene=CYP17A1
ırddisp.pl?gene=HFM1
ırddisp.pl?gene=MCM8
ırddisp.pl?gene=ESR2
ırddisp.pl?gene=ALB
ırddisp.pl?gene=AMH
ırddisp.pl?gene=DIAPH2
ırddisp.pl?gene=STAR
ırddisp.pl?gene=GALT
ırddisp.pl?gene=EIF2B2
ırddisp.pl?gene=FGFR2
ırddisp.pl?gene=ATM
ırddisp.pl?gene=TERT
ırddisp.pl?gene=AMHR2
ırddisp.pl?gene=IL10
ırddisp.pl?gene=FGFR1
ırddisp.pl?gene=MCM9
ırddisp.pl?gene=LHCGR
ırddisp.pl?gene=POLG
ırddisp.pl?gene=PSMC3IP
ırddisp.pl?gene=KRAS
ırddisp.pl?gene=SYCE1
ırddisp.pl?gene=MIR146A
ırddisp.pl?gene=MSH5
ırddisp.pl?gene=TGFB1

ırddisp.pl?gene=PAEP
ırddisp.pl?gene=LARS2
ırddisp.pl?gene=AIRE
ırddisp.pl?gene=PIK3CA
ırddisp.pl?gene=EIF2B4
ırddisp.pl?gene=MRE11
ırddisp.pl?gene=MMP2
ırddisp.pl?gene=ERBB2
ırddisp.pl?gene=HSD17B4
ırddisp.pl?gene=SOX9
ırddisp.pl?gene=FAS
ırddisp.pl?gene=HNF1B
ırddisp.pl?gene=TGFBR2
ırddisp.pl?gene=IGF2
ırddisp.pl?gene=TLR4
ırddisp.pl?gene=BRAF
ırddisp.pl?gene=FANCM
ırddisp.pl?gene=KITLG
ırddisp.pl?gene=CYP21A2
ırddisp.pl?gene=TWIST1
ırddisp.pl?gene=IGF1
ırddisp.pl?gene=APOE
ırddisp.pl?gene=RAD51C
ırddisp.pl?gene=EGF
ırddisp.pl?gene=CCR6
ırddisp.pl?gene=CDKN2A
ırddisp.pl?gene=MTOR
ırddisp.pl?gene=GATA4
ırddisp.pl?gene=MYC
ırddisp.pl?gene=RAD51
ırddisp.pl?gene=INHBA
ırddisp.pl?gene=REN
ırddisp.pl?gene=FLT1
ırddisp.pl?gene=WNT4
ırddisp.pl?gene=RAD50
ırddisp.pl?gene=FSHB
ırddisp.pl?gene=EGFR
ırddisp.pl?gene=TGFBR1
ırddisp.pl?gene=IFNG
ırddisp.pl?gene=FGFR3
ırddisp.pl?gene=MLH1
ırddisp.pl?gene=PREPL
ırddisp.pl?gene=HRAS
ırddisp.pl?gene=PDGFRB
ırddisp.pl?gene=NOTCH1
ırddisp.pl?gene=FASLG
ırddisp.pl?gene=MMP1
ırddisp.pl?gene=MDM2
ırddisp.pl?gene=WRN
ırddisp.pl?gene=HSD3B2
ırddisp.pl?gene=KISS1R
ırddisp.pl?gene=SOHLH1
ırddisp.pl?gene=BLM
ırddisp.pl?gene=FBN1

ırddisp.pl?gene=MRPS22
ırddisp.pl?gene=CLPP
ırddisp.pl?gene=NF1
ırddisp.pl?gene=NUP107
ırddisp.pl?gene=SPP1
ırddisp.pl?gene=TNF
ırddisp.pl?gene=SGO2
ırddisp.pl?gene=SMAD3
ırddisp.pl?gene=GNRH1
ırddisp.pl?gene=FOS
ırddisp.pl?gene=ERCC1
ırddisp.pl?gene=FN1
ırddisp.pl?gene=VEGFA
ırddisp.pl?gene=SMAD4
ırddisp.pl?gene=TGFB3
ırddisp.pl?gene=PGR
ırddisp.pl?gene=AFF2
ırddisp.pl?gene=BMP4
ırddisp.pl?gene=PPARG
ırddisp.pl?gene=BMP2
ırddisp.pl?gene=MIR144
ırddisp.pl?gene=CHEK2
ırddisp.pl?gene=LOX
ırddisp.pl?gene=MAPK1
ırddisp.pl?gene=MIR21
ırddisp.pl?gene=KIT
ırddisp.pl?gene=CAV1
ırddisp.pl?gene=MMP14
ırddisp.pl?gene=H19
ırddisp.pl?gene=IGFBP3
ırddisp.pl?gene=TGFB2
ırddisp.pl?gene=CASP8
ırddisp.pl?gene=GJA1
ırddisp.pl?gene=STK11
ırddisp.pl?gene=CDH1
ırddisp.pl?gene=INHA
ırddisp.pl?gene=STAT1
ırddisp.pl?gene=BNC1
ırddisp.pl?gene=RB1
ırddisp.pl?gene=GNAS
ırddisp.pl?gene=AARS2
ırddisp.pl?gene=SRC
ırddisp.pl?gene=PRL
ırddisp.pl?gene=ATR
ırddisp.pl?gene=AR
ırddisp.pl?gene=ERCC2
ırddisp.pl?gene=CTSD
ırddisp.pl?gene=WDR19
ırddisp.pl?gene=INSR
ırddisp.pl?gene=RAF1
ırddisp.pl?gene=MIR17
ırddisp.pl?gene=ELN
ırddisp.pl?gene=SNAI2
ırddisp.pl?gene=JAK2

rddisp.pl?gene=CXCL8
rddisp.pl?gene=NFKB1
rddisp.pl?gene=MIR126
rddisp.pl?gene=MIR155
rddisp.pl?gene=SHBG
rddisp.pl?gene=POR
rddisp.pl?gene=PDGFRA
rddisp.pl?gene=SRY
rddisp.pl?gene=ZMPSTE24
rddisp.pl?gene=MIR483
rddisp.pl?gene=IRS1
rddisp.pl?gene=ITGB3
rddisp.pl?gene=SQSTM1
rddisp.pl?gene=MAP2K1
rddisp.pl?gene=MET
rddisp.pl?gene=ENG
rddisp.pl?gene=PIK3R1
rddisp.pl?gene=RAD51D
rddisp.pl?gene=BUB1B
rddisp.pl?gene=FGF8
rddisp.pl?gene=APC
rddisp.pl?gene=GLI3
rddisp.pl?gene=CDKN3
rddisp.pl?gene=HLA-A
rddisp.pl?gene=MEG3
rddisp.pl?gene=IL1B
rddisp.pl?gene=LEP
rddisp.pl?gene=CHEK1
rddisp.pl?gene=DES
rddisp.pl?gene=MC2R
rddisp.pl?gene=CCN2
rddisp.pl?gene=AKT2
rddisp.pl?gene=MIR223
rddisp.pl?gene=NPM1
rddisp.pl?gene=SLC2A1
rddisp.pl?gene=CRP
rddisp.pl?gene=CDK4
rddisp.pl?gene=CFTR
rddisp.pl?gene=PTCH1
rddisp.pl?gene=MIR27A
rddisp.pl?gene=RHOA
rddisp.pl?gene=MMP9
rddisp.pl?gene=MIR140
rddisp.pl?gene=FANCF
rddisp.pl?gene=TWNK
rddisp.pl?gene=AXIN1
rddisp.pl?gene=PMM2
rddisp.pl?gene=NTRK1
rddisp.pl?gene=MIR210
rddisp.pl?gene=GNRHR
rddisp.pl?gene=FOXO3
rddisp.pl?gene=CYP11B1
rddisp.pl?gene=RUNX2
rddisp.pl?gene=HMGA2

ırddisp.pl?gene=TERC
ırddisp.pl?gene=FST
ırddisp.pl?gene=PTH
ırddisp.pl?gene=ATP7A
ırddisp.pl?gene=ETS1
ırddisp.pl?gene=CTLA4
ırddisp.pl?gene=RFC2
ırddisp.pl?gene=GHR
ırddisp.pl?gene=MIR34A
ırddisp.pl?gene=CGA
ırddisp.pl?gene=MIR127
ırddisp.pl?gene=FANCC
ırddisp.pl?gene=PALB2
ırddisp.pl?gene=POU5F1
ırddisp.pl?gene=MIR22
ırddisp.pl?gene=CD46
ırddisp.pl?gene=MSX2
ırddisp.pl?gene=FGF2
ırddisp.pl?gene=FBLN5
ırddisp.pl?gene=H2AC18
ırddisp.pl?gene=HIF1A
ırddisp.pl?gene=CAT
ırddisp.pl?gene=CSF3
ırddisp.pl?gene=MIR221
ırddisp.pl?gene=AFP
ırddisp.pl?gene=ALPL
ırddisp.pl?gene=TPO
ırddisp.pl?gene=CDKN1C
ırddisp.pl?gene=EIF2B5
ırddisp.pl?gene=MUC1
ırddisp.pl?gene=HLA-DRB1
ırddisp.pl?gene=RECQL4
ırddisp.pl?gene=CEP290
ırddisp.pl?gene=MEN1
ırddisp.pl?gene=IL2
ırddisp.pl?gene=ACE
ırddisp.pl?gene=CCBE1
ırddisp.pl?gene=SERPINE1
ırddisp.pl?gene=VWF
ırddisp.pl?gene=SHH
ırddisp.pl?gene=RYR1
ırddisp.pl?gene=SMAD6
ırddisp.pl?gene=IGF1R
ırddisp.pl?gene=SEPTIN9
ırddisp.pl?gene=COL2A1
ırddisp.pl?gene=CASP3
ırddisp.pl?gene=ARID1B
ırddisp.pl?gene=MIRLET7D
ırddisp.pl?gene=KDR
ırddisp.pl?gene=TSC1
ırddisp.pl?gene=CCND1
ırddisp.pl?gene=EDN1
ırddisp.pl?gene=EP300
ırddisp.pl?gene=ASNS

ırddisp.pl?gene=KISS1
ırddisp.pl?gene=STAT3
ırddisp.pl?gene=LGR6
ırddisp.pl?gene=MFAP5
ırddisp.pl?gene=CDKN1A
ırddisp.pl?gene=SIRT1
ırddisp.pl?gene=TSC2
ırddisp.pl?gene=RET
ırddisp.pl?gene=MSH2
ırddisp.pl?gene=SMARCA4
ırddisp.pl?gene=PMS2
ırddisp.pl?gene=NOTCH2
ırddisp.pl?gene=RIN2
ırddisp.pl?gene=TIMP1
ırddisp.pl?gene=PARP1
ırddisp.pl?gene=DKC1
ırddisp.pl?gene=SPINK1
ırddisp.pl?gene=REC8
ırddisp.pl?gene=LRP5
ırddisp.pl?gene=BSCL2
ırddisp.pl?gene=PAX2
ırddisp.pl?gene=IFT140
ırddisp.pl?gene=IFT122
ırddisp.pl?gene=ABCB1
ırddisp.pl?gene=PTPN22
ırddisp.pl?gene=ICAM1
ırddisp.pl?gene=TG
ırddisp.pl?gene=MIR139
ırddisp.pl?gene=TTN
ırddisp.pl?gene=FOXP2
ırddisp.pl?gene=CASR
ırddisp.pl?gene=ACTA2
ırddisp.pl?gene=SOX3
ırddisp.pl?gene=VCP
ırddisp.pl?gene=ABCA3
ırddisp.pl?gene=TTC21B
ırddisp.pl?gene=BRIP1
ırddisp.pl?gene=PTPN11
ırddisp.pl?gene=NPHP1
ırddisp.pl?gene=TTR
ırddisp.pl?gene=NPHP4
ırddisp.pl?gene=IL4
ırddisp.pl?gene=GK
ırddisp.pl?gene=CD2AP
ırddisp.pl?gene=ERAL1
ırddisp.pl?gene=IL1A
ırddisp.pl?gene=IGFBP1
ırddisp.pl?gene=TIMP2
ırddisp.pl?gene=MIRLET7C
ırddisp.pl?gene=PTGS2
ırddisp.pl?gene=IHH
ırddisp.pl?gene=CSF2
ırddisp.pl?gene=KRT7
ırddisp.pl?gene=SOD2

ırddisp.pl?gene=EPPIN
ırddisp.pl?gene=ABCD1
ırddisp.pl?gene=NPHP3
ırddisp.pl?gene=OFD1
ırddisp.pl?gene=NKX2-5
ırddisp.pl?gene=PGRMC1
ırddisp.pl?gene=PAX3
ırddisp.pl?gene=INHBB
ırddisp.pl?gene=SHOX
ırddisp.pl?gene=FGF10
ırddisp.pl?gene=GH1
ırddisp.pl?gene=MSH6
ırddisp.pl?gene=IL17A
ırddisp.pl?gene=APOA1
ırddisp.pl?gene=RETN
ırddisp.pl?gene=EIF2B3
ırddisp.pl?gene=EIF2B1
ırddisp.pl?gene=DICER1
ırddisp.pl?gene=CCL2
ırddisp.pl?gene=FRAXA
ırddisp.pl?gene=DSTYK
ırddisp.pl?gene=PRKCD
ırddisp.pl?gene=JUN
ırddisp.pl?gene=COL1A1
ırddisp.pl?gene=DACH2
ırddisp.pl?gene=APOB
ırddisp.pl?gene=PLAU
ırddisp.pl?gene=BMP6
ırddisp.pl?gene=TNFSF11
ırddisp.pl?gene=RCBTB1
ırddisp.pl?gene=LIG4
ırddisp.pl?gene=MTHFR
ırddisp.pl?gene=ZP3
ırddisp.pl?gene=CFAP47
ırddisp.pl?gene=EPO
ırddisp.pl?gene=VDR
ırddisp.pl?gene=HP
ırddisp.pl?gene=ANGPT2
ırddisp.pl?gene=ACTN4
ırddisp.pl?gene=INVS
ırddisp.pl?gene=HLA-B
ırddisp.pl?gene=MSH4
ırddisp.pl?gene=POLR3A
ırddisp.pl?gene=RELA
ırddisp.pl?gene=ALPP
ırddisp.pl?gene=NANOS3
ırddisp.pl?gene=CDH23
ırddisp.pl?gene=SOHLH2
ırddisp.pl?gene=BDNF
ırddisp.pl?gene=BARD1
ırddisp.pl?gene=MUC16
ırddisp.pl?gene=FLNA
ırddisp.pl?gene=IGF2R
ırddisp.pl?gene=MME

ırddisp.pl?gene=TWIST2
ırddisp.pl?gene=SERPINA3
ırddisp.pl?gene=DAZL
ırddisp.pl?gene=BGLAP
ırddisp.pl?gene=CYP11B2
ırddisp.pl?gene=SMC1A
ırddisp.pl?gene=KDM6A
ırddisp.pl?gene=IGFBP2
ırddisp.pl?gene=ADIPOQ
ırddisp.pl?gene=MAP3K1
ırddisp.pl?gene=COL1A2
ırddisp.pl?gene=ANXA5
ırddisp.pl?gene=ZIC3
ırddisp.pl?gene=MIR145
ırddisp.pl?gene=LHX8
ırddisp.pl?gene=TRAF3IP1
ırddisp.pl?gene=PRKD1
ırddisp.pl?gene=F2
ırddisp.pl?gene=USP8
ırddisp.pl?gene=HELLS
ırddisp.pl?gene=TNNT2
ırddisp.pl?gene=VIM
ırddisp.pl?gene=TNFRSF1A
ırddisp.pl?gene=ADAMTS19
ırddisp.pl?gene=FGF1
ırddisp.pl?gene=GALK1
ırddisp.pl?gene=ERBB3
ırddisp.pl?gene=ENO1
ırddisp.pl?gene=WWOX
ırddisp.pl?gene=SUFU
ırddisp.pl?gene=FGF9
ırddisp.pl?gene=TOP1
ırddisp.pl?gene=ATRX
ırddisp.pl?gene=SPO11
ırddisp.pl?gene=JAG1
ırddisp.pl?gene=ABCB4
ırddisp.pl?gene=TEK
ırddisp.pl?gene=EPRS1
ırddisp.pl?gene=GAPDH
ırddisp.pl?gene=HGF
ırddisp.pl?gene=GPR3
ırddisp.pl?gene=ALX4
ırddisp.pl?gene=EFNB1
ırddisp.pl?gene=NLRP5
ırddisp.pl?gene=CD19
ırddisp.pl?gene=FANCA
ırddisp.pl?gene=CD44
ırddisp.pl?gene=SOX10
ırddisp.pl?gene=DDX4
ırddisp.pl?gene=CYCS
ırddisp.pl?gene=LDB3
ırddisp.pl?gene=NUP133
ırddisp.pl?gene=EIF4ENIF1
ırddisp.pl?gene=TMEM67

rddisp.pl?gene=MT-CYB
rddisp.pl?gene=CD40LG
rddisp.pl?gene=DMRT1
rddisp.pl?gene=TMEM216
rddisp.pl?gene=IQCB1
rddisp.pl?gene=NFKB2
rddisp.pl?gene=FGF7
rddisp.pl?gene=MAPK14
rddisp.pl?gene=FOXE3
rddisp.pl?gene=ACTC1
rddisp.pl?gene=ZP2
rddisp.pl?gene=GSTP1
rddisp.pl?gene=XPNPEP2
rddisp.pl?gene=KIF7
rddisp.pl?gene=PRKAR1A
rddisp.pl?gene=TCF12
rddisp.pl?gene=BCL2L1
rddisp.pl?gene=MMP7
rddisp.pl?gene=CLU
rddisp.pl?gene=CEP164
rddisp.pl?gene=LIMK1
rddisp.pl?gene=RECQL
rddisp.pl?gene=USP9X
rddisp.pl?gene=SDCCAG8
rddisp.pl?gene=CBL
rddisp.pl?gene=POLR3H
rddisp.pl?gene=IFT172
rddisp.pl?gene=ANGPT1
rddisp.pl?gene=MAPK8
rddisp.pl?gene=CCL5
rddisp.pl?gene=VEGFC
rddisp.pl?gene=MAPK3
rddisp.pl?gene=MIR125A
rddisp.pl?gene=COL3A1
rddisp.pl?gene=PRKN
rddisp.pl?gene=SMC1B
rddisp.pl?gene=APEX1
rddisp.pl?gene=CXCL12
rddisp.pl?gene=CPEB1
rddisp.pl?gene=SYNE2
rddisp.pl?gene=SYCP3
rddisp.pl?gene=THBS1
rddisp.pl?gene=IL1RAPL1
rddisp.pl?gene=RAB23
rddisp.pl?gene=CASP10
rddisp.pl?gene=H2AX
rddisp.pl?gene=HPRT1
rddisp.pl?gene=TNFSF10
rddisp.pl?gene=IL6R
rddisp.pl?gene=LCN2
rddisp.pl?gene=SPIDR
rddisp.pl?gene=FANCD2
rddisp.pl?gene=MMP8
rddisp.pl?gene=CSF1

ırddisp.pl?gene=IFNA1
ırddisp.pl?gene=MIR200A
ırddisp.pl?gene=RAD21L1
ırddisp.pl?gene=IL3
ırddisp.pl?gene=ICOSLG
ırddisp.pl?gene=RPGRIP1L
ırddisp.pl?gene=DNMT1
ırddisp.pl?gene=NOTCH3
ırddisp.pl?gene=DAB2
ırddisp.pl?gene=RPS6KB1
ırddisp.pl?gene=NPHS1
ırddisp.pl?gene=COL5A1
ırddisp.pl?gene=SARS2
ırddisp.pl?gene=ELANE
ırddisp.pl?gene=DCN
ırddisp.pl?gene=GJC2
ırddisp.pl?gene=FOXP3
ırddisp.pl?gene=PECAM1
ırddisp.pl?gene=CRH
ırddisp.pl?gene=STRA8
ırddisp.pl?gene=CYP1A1
ırddisp.pl?gene=CXCL10
ırddisp.pl?gene=COG7
ırddisp.pl?gene=DNMT3A
ırddisp.pl?gene=BIRC5
ırddisp.pl?gene=TYMP
ırddisp.pl?gene=SYCP1
ırddisp.pl?gene=FLNB
ırddisp.pl?gene=PLAUR
ırddisp.pl?gene=HSPA4
ırddisp.pl?gene=FAM122C
ırddisp.pl?gene=EFEMP2
ırddisp.pl?gene=MT-CO1
ırddisp.pl?gene=CYP3A4
ırddisp.pl?gene=ATP7B
ırddisp.pl?gene=CXCR4
ırddisp.pl?gene=XPA
ırddisp.pl?gene=THBD
ırddisp.pl?gene=ACAN
ırddisp.pl?gene=IFT80
ırddisp.pl?gene=TBX5
ırddisp.pl?gene=DMPK
ırddisp.pl?gene=GRP
ırddisp.pl?gene=SLPI
ırddisp.pl?gene=DNMT3B
ırddisp.pl?gene=EZH2
ırddisp.pl?gene=SFTPB
ırddisp.pl?gene=XIAP
ırddisp.pl?gene=PTGS1
ırddisp.pl?gene=NRXN1
ırddisp.pl?gene=HDAC9
ırddisp.pl?gene=PPP3CA
ırddisp.pl?gene=ERCC3
ırddisp.pl?gene=PCSK1

ırddisp.pl?gene=RASGRP1
ırddisp.pl?gene=HSPA5
ırddisp.pl?gene=HSPD1
ırddisp.pl?gene=HADHA
ırddisp.pl?gene=NQO1
ırddisp.pl?gene=HNF4A
ırddisp.pl?gene=INF2
ırddisp.pl?gene=CDK1
ırddisp.pl?gene=MUC5AC
ırddisp.pl?gene=MIR182
ırddisp.pl?gene=AIP
ırddisp.pl?gene=ERBB4
ırddisp.pl?gene=BMPR1A
ırddisp.pl?gene=MIR143
ırddisp.pl?gene=MIR9-1
ırddisp.pl?gene=SP1
ırddisp.pl?gene=DHFR
ırddisp.pl?gene=CD34
ırddisp.pl?gene=ARID1A
ırddisp.pl?gene=MIR195
ırddisp.pl?gene=XRCC2
ırddisp.pl?gene=GPC3
ırddisp.pl?gene=ZIC1
ırddisp.pl?gene=GZMB
ırddisp.pl?gene=MIR200C
ırddisp.pl?gene=B4GALNT1
ırddisp.pl?gene=CDC25A
ırddisp.pl?gene=CFLAR
ırddisp.pl?gene=FREM1
ırddisp.pl?gene=LDLR
ırddisp.pl?gene=HFE
ırddisp.pl?gene=BSG
ırddisp.pl?gene=CDH5
ırddisp.pl?gene=RAN
ırddisp.pl?gene=SMAD2
ırddisp.pl?gene=SLC27A4
ırddisp.pl?gene=MIR141
ırddisp.pl?gene=TNNC1
ırddisp.pl?gene=SMARCB1
ırddisp.pl?gene=TMEM231
ırddisp.pl?gene=BCL2
ırddisp.pl?gene=EDNRA
ırddisp.pl?gene=SLC34A1
ırddisp.pl?gene=ABCC2
ırddisp.pl?gene=OXT
ırddisp.pl?gene=CD40
ırddisp.pl?gene=C3
ırddisp.pl?gene=NRAS
ırddisp.pl?gene=SOX2
ırddisp.pl?gene=CHD7
ırddisp.pl?gene=PLP1
ırddisp.pl?gene=CDH2
ırddisp.pl?gene=NFKBIA
ırddisp.pl?gene=DCAF17

ırddisp.pl?gene=KCNQ1OT1
ırddisp.pl?gene=PCNA
ırddisp.pl?gene=THOC6
ırddisp.pl?gene=TP53BP1
ırddisp.pl?gene=RAC1
ırddisp.pl?gene=PGM1
ırddisp.pl?gene=COG4
ırddisp.pl?gene=TXN
ırddisp.pl?gene=AGPAT2
ırddisp.pl?gene=KRT18
ırddisp.pl?gene=F5
ırddisp.pl?gene=JAK1
ırddisp.pl?gene=PTPA
ırddisp.pl?gene=MIR181A1
ırddisp.pl?gene=AKT3
ırddisp.pl?gene=HSP90AA1
ırddisp.pl?gene=NRP1
ırddisp.pl?gene=MIR10B
ırddisp.pl?gene=RARB
ırddisp.pl?gene=CDK6
ırddisp.pl?gene=ABL1
ırddisp.pl?gene=BECN1
ırddisp.pl?gene=PIK3CG
ırddisp.pl?gene=CLDN3
ırddisp.pl?gene=TFRC
ırddisp.pl?gene=GSN
ırddisp.pl?gene=MAP2K2
ırddisp.pl?gene=MIR205
ırddisp.pl?gene=MYLK
ırddisp.pl?gene=PLCG1
ırddisp.pl?gene=TNFRSF10B
ırddisp.pl?gene=FOXM1
ırddisp.pl?gene=FOXO1
ırddisp.pl?gene=SCN5A
ırddisp.pl?gene=RAD52
ırddisp.pl?gene=MIR30A
ırddisp.pl?gene=MALAT1
ırddisp.pl?gene=CC2D2A
ırddisp.pl?gene=GDF15
ırddisp.pl?gene=BCL2L11
ırddisp.pl?gene=CDKN1B
ırddisp.pl?gene=PLG
ırddisp.pl?gene=EHMT1
ırddisp.pl?gene=F3
ırddisp.pl?gene=MECP2
ırddisp.pl?gene=TPM3
ırddisp.pl?gene=SERPINC1
ırddisp.pl?gene=MECOM
ırddisp.pl?gene=MCM3AP
ırddisp.pl?gene=SPG7
ırddisp.pl?gene=NBR1
ırddisp.pl?gene=PAX7
ırddisp.pl?gene=PROM1
ırddisp.pl?gene=MIR142

ırddisp.pl?gene=PAPPA
ırddisp.pl?gene=RAD54L
ırddisp.pl?gene=DLL4
ırddisp.pl?gene=HBEGF
ırddisp.pl?gene=ENPP1
ırddisp.pl?gene=CSF1R
ırddisp.pl?gene=CLDN4
ırddisp.pl?gene=TPM1
ırddisp.pl?gene=KRT14
ırddisp.pl?gene=IL7
ırddisp.pl?gene=COL5A2
ırddisp.pl?gene=SERPINB2
ırddisp.pl?gene=RHO
ırddisp.pl?gene=EIF4EBP1
ırddisp.pl?gene=PTTG1
ırddisp.pl?gene=SLC17A5
ırddisp.pl?gene=EFEMP1
ırddisp.pl?gene=GCLC
ırddisp.pl?gene=ALG2
ırddisp.pl?gene=ITGB1
ırddisp.pl?gene=IL2RA
ırddisp.pl?gene=POLE
ırddisp.pl?gene=TBX19
ırddisp.pl?gene=TFAP2A
ırddisp.pl?gene=ABCG5
ırddisp.pl?gene=TUSC3
ırddisp.pl?gene=SST
ırddisp.pl?gene=MIR150
ırddisp.pl?gene=BAX
ırddisp.pl?gene=ABCA1
ırddisp.pl?gene=GLI2
ırddisp.pl?gene=MIR148A
ırddisp.pl?gene=AGTR1
ırddisp.pl?gene=PLK1
ırddisp.pl?gene=ZFPM2
ırddisp.pl?gene=IFT52
ırddisp.pl?gene=CTSB
ırddisp.pl?gene=DSP
ırddisp.pl?gene=GAS1
ırddisp.pl?gene=CASP7
ırddisp.pl?gene=HMGB1
ırddisp.pl?gene=KCNJ2
ırddisp.pl?gene=CGB5
ırddisp.pl?gene=ATAD1
ırddisp.pl?gene=SLC25A4
ırddisp.pl?gene=GADD45A
ırddisp.pl?gene=WRAP53
ırddisp.pl?gene=PTH1R
ırddisp.pl?gene=RRM2B
ırddisp.pl?gene=TNFRSF10A
ırddisp.pl?gene=LPL
ırddisp.pl?gene=TNNI3
ırddisp.pl?gene=NDUFB11
ırddisp.pl?gene=PROS1

ırddisp.pl?gene=ANPEP
ırddisp.pl?gene=GAD1
ırddisp.pl?gene=FASN
ırddisp.pl?gene=TCTN3
ırddisp.pl?gene=CD4
ırddisp.pl?gene=FBXW7
ırddisp.pl?gene=GRIN2B
ırddisp.pl?gene=PDGFRL
ırddisp.pl?gene=COL11A2
ırddisp.pl?gene=NRG1
ırddisp.pl?gene=ACTA1
ırddisp.pl?gene=GUSB
ırddisp.pl?gene=MIR376C
ırddisp.pl?gene=EIF4E
ırddisp.pl?gene=CST3
ırddisp.pl?gene=CDKN2B
ırddisp.pl?gene=MIRLET7I
ırddisp.pl?gene=HOTAIR
ırddisp.pl?gene=GRN
ırddisp.pl?gene=EZR
ırddisp.pl?gene=ERN1
ırddisp.pl?gene=PALLD
ırddisp.pl?gene=FANCB
ırddisp.pl?gene=NKX2-1
ırddisp.pl?gene=CD247
ırddisp.pl?gene=MED12
ırddisp.pl?gene=IRS2
ırddisp.pl?gene=WNT5A
ırddisp.pl?gene=TTI2
ırddisp.pl?gene=CALCA
ırddisp.pl?gene=TNFRSF11B
ırddisp.pl?gene=GGT1
ırddisp.pl?gene=MBL2
ırddisp.pl?gene=NANOG
ırddisp.pl?gene=ERCC4
ırddisp.pl?gene=SAMD9
ırddisp.pl?gene=PLAGL1
ırddisp.pl?gene=DRD2
ırddisp.pl?gene=VTN
ırddisp.pl?gene=FH
ırddisp.pl?gene=ACP5
ırddisp.pl?gene=FBLN1
ırddisp.pl?gene=LCAT
ırddisp.pl?gene=MIR495
ırddisp.pl?gene=NEK8
ırddisp.pl?gene=ATP6V0A2
ırddisp.pl?gene=COMT
ırddisp.pl?gene=BBS10
ırddisp.pl?gene=COL11A1
ırddisp.pl?gene=TKT
ırddisp.pl?gene=AGT
ırddisp.pl?gene=MSX1
ırddisp.pl?gene=GDNF
ırddisp.pl?gene=PGF

ırddisp.pl?gene=NEAT1
ırddisp.pl?gene=TSHR
ırddisp.pl?gene=CXADR
ırddisp.pl?gene=KCNQ1
ırddisp.pl?gene=RAD21
ırddisp.pl?gene=LYVE1
ırddisp.pl?gene=TUG1
ırddisp.pl?gene=GHRL
ırddisp.pl?gene=NR3C1
ırddisp.pl?gene=CPT2
ırddisp.pl?gene=ALMS1
ırddisp.pl?gene=TLR2
ırddisp.pl?gene=KDM4C
ırddisp.pl?gene=MLX
ırddisp.pl?gene=NACC1
ırddisp.pl?gene=HDAC8
ırddisp.pl?gene=CCNG1
ırddisp.pl?gene=CDKN2B-AS1
ırddisp.pl?gene=CTSL
ırddisp.pl?gene=CDC42
ırddisp.pl?gene=CYP27B1
ırddisp.pl?gene=CD24
ırddisp.pl?gene=FSCN1
ırddisp.pl?gene=XIST
ırddisp.pl?gene=PCSK9
ırddisp.pl?gene=SLC22A18
ırddisp.pl?gene=PGBD3
ırddisp.pl?gene=SLX4
ırddisp.pl?gene=F7
ırddisp.pl?gene=EPHB4
ırddisp.pl?gene=MLH3
ırddisp.pl?gene=NDUFAF2
ırddisp.pl?gene=RPS6KA2
ırddisp.pl?gene=MIR29B1
ırddisp.pl?gene=ZNF711
ırddisp.pl?gene=SERPINA7
ırddisp.pl?gene=CD8A
ırddisp.pl?gene=CLCNKB
ırddisp.pl?gene=RSPO1
ırddisp.pl?gene=FLI1
ırddisp.pl?gene=SMPD1
ırddisp.pl?gene=RPS19
ırddisp.pl?gene=KRT5
ırddisp.pl?gene=GTF2I
ırddisp.pl?gene=ACTB
ırddisp.pl?gene=TGFA
ırddisp.pl?gene=CCK
ırddisp.pl?gene=LIPC
ırddisp.pl?gene=MAGEA1
ırddisp.pl?gene=BAG3
ırddisp.pl?gene=BAZ1B
ırddisp.pl?gene=SERPINA1
ırddisp.pl?gene=CR2
ırddisp.pl?gene=MIR99A

ırddisp.pl?gene=GATA3
ırddisp.pl?gene=PAX8
ırddisp.pl?gene=SUCLG1
ırddisp.pl?gene=SNCA
ırddisp.pl?gene=HMGA1
ırddisp.pl?gene=JUP
ırddisp.pl?gene=STN1
ırddisp.pl?gene=GFAP
ırddisp.pl?gene=TH2LCRR
ırddisp.pl?gene=CRYAA
ırddisp.pl?gene=CFH
ırddisp.pl?gene=XRCC5
ırddisp.pl?gene=NOS3
ırddisp.pl?gene=TMEM138
ırddisp.pl?gene=EMD
ırddisp.pl?gene=XRCC6
ırddisp.pl?gene=SPRY4-IT1
ırddisp.pl?gene=PTHLH
ırddisp.pl?gene=SOD1
ırddisp.pl?gene=CFI
ırddisp.pl?gene=GTF2IRD1
ırddisp.pl?gene=FGF23
ırddisp.pl?gene=KRT20
ırddisp.pl?gene=FXYD2
ırddisp.pl?gene=SELE
ırddisp.pl?gene=HERC2
ırddisp.pl?gene=GHRH
ırddisp.pl?gene=SNRPN
ırddisp.pl?gene=FGFR4
ırddisp.pl?gene=SPG11
ırddisp.pl?gene=SYNE1
ırddisp.pl?gene=TF
ırddisp.pl?gene=CDK2
ırddisp.pl?gene=STIM1
ırddisp.pl?gene=PEX1
ırddisp.pl?gene=RPS6KA3
ırddisp.pl?gene=PURA
ırddisp.pl?gene=SRA1
ırddisp.pl?gene=CGB3
ırddisp.pl?gene=HAND2
ırddisp.pl?gene=POLG2
ırddisp.pl?gene=HMGCR
ırddisp.pl?gene=PEX13
ırddisp.pl?gene=PLEC
ırddisp.pl?gene=NPPB
ırddisp.pl?gene=NDRG1
ırddisp.pl?gene=CD274
ırddisp.pl?gene=MMP3
ırddisp.pl?gene=EDNRB
ırddisp.pl?gene=CRNDE
ırddisp.pl?gene=GPR101
ırddisp.pl?gene=PRKDC
ırddisp.pl?gene=NEB
ırddisp.pl?gene=WNT10A

ırddisp.pl?gene=TMEM43
ırddisp.pl?gene=MFN2
ırddisp.pl?gene=TERF1
ırddisp.pl?gene=THPO
ırddisp.pl?gene=TBL2
ırddisp.pl?gene=NOS2
ırddisp.pl?gene=HDAC1
ırddisp.pl?gene=PEX2
ırddisp.pl?gene=CLIP2
ırddisp.pl?gene=BMPR2
ırddisp.pl?gene=SLC31A1
ırddisp.pl?gene=PTK2
ırddisp.pl?gene=HNF1A
ırddisp.pl?gene=HOXA11
ırddisp.pl?gene=HSPB1
ırddisp.pl?gene=NSD1
ırddisp.pl?gene=ABCC1
ırddisp.pl?gene=IL6ST
ırddisp.pl?gene=PROP1
ırddisp.pl?gene=FOXE1
ırddisp.pl?gene=E2F1
ırddisp.pl?gene=TNXB
ırddisp.pl?gene=GLIS2
ırddisp.pl?gene=CCNE1
ırddisp.pl?gene=PRODH
ırddisp.pl?gene=SUCLA2
ırddisp.pl?gene=MMACHC
ırddisp.pl?gene=SOCS3
ırddisp.pl?gene=TERF2
ırddisp.pl?gene=SPARC
ırddisp.pl?gene=UMOD
ırddisp.pl?gene=ITPR3
ırddisp.pl?gene=GSTM1
ırddisp.pl?gene=CEACAM5
ırddisp.pl?gene=MYH11
ırddisp.pl?gene=PRTN3
ırddisp.pl?gene=EDN3
ırddisp.pl?gene=SETD2
ırddisp.pl?gene=PRSS1
ırddisp.pl?gene=IL18
ırddisp.pl?gene=SOS1
ırddisp.pl?gene=HSD3BP4
ırddisp.pl?gene=GCK
ırddisp.pl?gene=CROCC
ırddisp.pl?gene=GREM1
ırddisp.pl?gene=MYH6
ırddisp.pl?gene=MEGF8
ırddisp.pl?gene=TCF7L2
ırddisp.pl?gene=TMCO1
ırddisp.pl?gene=TYMS
ırddisp.pl?gene=S100B
ırddisp.pl?gene=FOLR1
ırddisp.pl?gene=NPY
ırddisp.pl?gene=LIG1

ırddisp.pl?gene=NOD2
ırddisp.pl?gene=AURKA
ırddisp.pl?gene=GBA
ırddisp.pl?gene=AQP2
ırddisp.pl?gene=FEN1
ırddisp.pl?gene=MS4A1
ırddisp.pl?gene=L1CAM
ırddisp.pl?gene=CASP9
ırddisp.pl?gene=RPA1
ırddisp.pl?gene=BSND
ırddisp.pl?gene=HSD11B2
ırddisp.pl?gene=DNAH8
ırddisp.pl?gene=U2AF1
ırddisp.pl?gene=NOG
ırddisp.pl?gene=NR1H2
ırddisp.pl?gene=CCNA2
ırddisp.pl?gene=LMNB2
ırddisp.pl?gene=EDAR
ırddisp.pl?gene=PON1
ırddisp.pl?gene=WFDC2
ırddisp.pl?gene=TIMP3
ırddisp.pl?gene=MT-ND1
ırddisp.pl?gene=OSR2
ırddisp.pl?gene=AMBP
ırddisp.pl?gene=ABCB11
ırddisp.pl?gene=UGT1A1
ırddisp.pl?gene=LIFR
ırddisp.pl?gene=MCL1
ırddisp.pl?gene=EWSR1
ırddisp.pl?gene=RBBP8
ırddisp.pl?gene=PRKCA
ırddisp.pl?gene=PLOD1
ırddisp.pl?gene=LGALS3
ırddisp.pl?gene=MMUT
ırddisp.pl?gene=FGF3
ırddisp.pl?gene=BCR
ırddisp.pl?gene=EXO1
ırddisp.pl?gene=ABCG2
ırddisp.pl?gene=MLXIPL
ırddisp.pl?gene=LAMA2
ırddisp.pl?gene=HSPA1A
ırddisp.pl?gene=LEMD3
ırddisp.pl?gene=FIG4
ırddisp.pl?gene=MAGT1
ırddisp.pl?gene=AAAS
ırddisp.pl?gene=FGF4
ırddisp.pl?gene=TOP2A
ırddisp.pl?gene=ADA
ırddisp.pl?gene=SNAI1
ırddisp.pl?gene=PRDM10
ırddisp.pl?gene=HSD11B1
ırddisp.pl?gene=PLAT
ırddisp.pl?gene=MIF
ırddisp.pl?gene=DIABLO

ırddisp.pl?gene=CCNB1
ırddisp.pl?gene=LTBP4
ırddisp.pl?gene=MIR23A
ırddisp.pl?gene=LBR
ırddisp.pl?gene=MUC4
ırddisp.pl?gene=NME1
ırddisp.pl?gene=CP
ırddisp.pl?gene=APPL1
ırddisp.pl?gene=CREBBP
ırddisp.pl?gene=YAP1
ırddisp.pl?gene=TUBGCP6
ırddisp.pl?gene=ITGB4
ırddisp.pl?gene=HADHB
ırddisp.pl?gene=GATA6
ırddisp.pl?gene=HLA-DQB1
ırddisp.pl?gene=STS
ırddisp.pl?gene=NPPA
ırddisp.pl?gene=ABCC6
ırddisp.pl?gene=DUPXQ27.3Q28
ırddisp.pl?gene=CPS1
ırddisp.pl?gene=GCM2
ırddisp.pl?gene=C1S
ırddisp.pl?gene=TH
ırddisp.pl?gene=SELP
ırddisp.pl?gene=ZEB1
ırddisp.pl?gene=CDON
ırddisp.pl?gene=DDC
ırddisp.pl?gene=RLIM
ırddisp.pl?gene=BMPR1B
ırddisp.pl?gene=VCL
ırddisp.pl?gene=ROCK1
ırddisp.pl?gene=CLDN14
ırddisp.pl?gene=SMAD7
ırddisp.pl?gene=IL1RN
ırddisp.pl?gene=PLIN1
ırddisp.pl?gene=TPH1
ırddisp.pl?gene=BUB1
ırddisp.pl?gene=TGM1
ırddisp.pl?gene=NAGS
ırddisp.pl?gene=ADAMTS13
ırddisp.pl?gene=TXNRD2
ırddisp.pl?gene=PTPRC
ırddisp.pl?gene=CLDN16
ırddisp.pl?gene=RASSF1
ırddisp.pl?gene=GAA
ırddisp.pl?gene=SKP2
ırddisp.pl?gene=IFI27
ırddisp.pl?gene=ZFHX4
ırddisp.pl?gene=ABCC8
ırddisp.pl?gene=VIP
ırddisp.pl?gene=HSPG2
ırddisp.pl?gene=MITF
ırddisp.pl?gene=CRYAB
ırddisp.pl?gene=NES

ırddisp.pl?gene=CD28
ırddisp.pl?gene=SERPINH1
ırddisp.pl?gene=TFF1
ırddisp.pl?gene=ABCA12
ırddisp.pl?gene=MPO
ırddisp.pl?gene=CXCL1
ırddisp.pl?gene=RBM28
ırddisp.pl?gene=ERCC5
ırddisp.pl?gene=RAP1A
ırddisp.pl?gene=SULF1
ırddisp.pl?gene=CDC25C
ırddisp.pl?gene=ESRRB
ırddisp.pl?gene=APTX
ırddisp.pl?gene=SMARCA2
ırddisp.pl?gene=FZD4
ırddisp.pl?gene=TLR9
ırddisp.pl?gene=SMC3
ırddisp.pl?gene=YARS2
ırddisp.pl?gene=ATP8B1
ırddisp.pl?gene=VCAM1
ırddisp.pl?gene=ALOX5
ırddisp.pl?gene=CTNNA1
ırddisp.pl?gene=DLK1
ırddisp.pl?gene=NCOA3
ırddisp.pl?gene=USP7
ırddisp.pl?gene=TGM2
ırddisp.pl?gene=PPARA
ırddisp.pl?gene=ZEB2
ırddisp.pl?gene=KLK7
ırddisp.pl?gene=UQCRFS1
ırddisp.pl?gene=TP73
ırddisp.pl?gene=MIR135A1
ırddisp.pl?gene=MKS1
ırddisp.pl?gene=PKHD1
ırddisp.pl?gene=B2M
ırddisp.pl?gene=ZIC4
ırddisp.pl?gene=ANXA2
ırddisp.pl?gene=KLRK1
ırddisp.pl?gene=ECHS1
ırddisp.pl?gene=LEPQTL1
ırddisp.pl?gene=RB1CC1
ırddisp.pl?gene=LHB
ırddisp.pl?gene=IL24
ırddisp.pl?gene=CKB
ırddisp.pl?gene=PSAP
ırddisp.pl?gene=CHAT
ırddisp.pl?gene=TRH
ırddisp.pl?gene=PDCD1
ırddisp.pl?gene=IKBKB
ırddisp.pl?gene=MBP
ırddisp.pl?gene=NTRK3
ırddisp.pl?gene=LEPR
ırddisp.pl?gene=SHC1
ırddisp.pl?gene=CTCF

ırddisp.pl?gene=KCNH2
ırddisp.pl?gene=BANF1
ırddisp.pl?gene=F2R
ırddisp.pl?gene=CD36
ırddisp.pl?gene=PCCB
ırddisp.pl?gene=BCKDHA
ırddisp.pl?gene=NFE2L2
ırddisp.pl?gene=G6PD
ırddisp.pl?gene=DDR2
ırddisp.pl?gene=IGFBP5
ırddisp.pl?gene=SGO1
ırddisp.pl?gene=FANCL
ırddisp.pl?gene=MIR24-1
ırddisp.pl?gene=USP53
ırddisp.pl?gene=FANCI
ırddisp.pl?gene=ESRRG
ırddisp.pl?gene=UBE3A
ırddisp.pl?gene=TNFRSF10C
ırddisp.pl?gene=RARA
ırddisp.pl?gene=LTA
ırddisp.pl?gene=PISRT1
ırddisp.pl?gene=NR3C2
ırddisp.pl?gene=CCND3
ırddisp.pl?gene=KLK3
ırddisp.pl?gene=SOX14
ırddisp.pl?gene=BPESC1
ırddisp.pl?gene=SPATA8-AS1
ırddisp.pl?gene=PDX1
ırddisp.pl?gene=MTRR
ırddisp.pl?gene=SELL
ırddisp.pl?gene=CREB1
ırddisp.pl?gene=RAB27A
ırddisp.pl?gene=NAMPT
ırddisp.pl?gene=NPHS2
ırddisp.pl?gene=DLL1
ırddisp.pl?gene=IFNA2
ırddisp.pl?gene=NR1H4
ırddisp.pl?gene=KLK5
ırddisp.pl?gene=PTPN3
ırddisp.pl?gene=CALB2
ırddisp.pl?gene=RPS4X
ırddisp.pl?gene=TAGLN
ırddisp.pl?gene=KLK4
ırddisp.pl?gene=AREG
ırddisp.pl?gene=SELENBP1
ırddisp.pl?gene=AKR1B1
ırddisp.pl?gene=GAS5
ırddisp.pl?gene=BCKDHB
ırddisp.pl?gene=ALPG
ırddisp.pl?gene=GNA11
ırddisp.pl?gene=FANCE
ırddisp.pl?gene=GNAI2
ırddisp.pl?gene=RECQL5
ırddisp.pl?gene=PRF1

ırddisp.pl?gene=PRKG1
ırddisp.pl?gene=ELAVL1
ırddisp.pl?gene=FLT4
ırddisp.pl?gene=RAD51B
ırddisp.pl?gene=CD55
ırddisp.pl?gene=PHB
ırddisp.pl?gene=MT-ATP6
ırddisp.pl?gene=POSTN
ırddisp.pl?gene=KMT2D
ırddisp.pl?gene=MGP
ırddisp.pl?gene=CTAG1B
ırddisp.pl?gene=MYD88
ırddisp.pl?gene=EPHX1
ırddisp.pl?gene=HPSE2
ırddisp.pl?gene=MIRLET7B
ırddisp.pl?gene=MMP12
ırddisp.pl?gene=SDHA
ırddisp.pl?gene=TET2
ırddisp.pl?gene=PAX6
ırddisp.pl?gene=SCNN1A
ırddisp.pl?gene=PCCA
ırddisp.pl?gene=RBPJ
ırddisp.pl?gene=MIR130A
ırddisp.pl?gene=HBG2
ırddisp.pl?gene=MUC5B
ırddisp.pl?gene=AURKB
ırddisp.pl?gene=LAMP2
ırddisp.pl?gene=HOXD13
ırddisp.pl?gene=IFNB1
ırddisp.pl?gene=F9
ırddisp.pl?gene=NUP160
ırddisp.pl?gene=TDGF1
ırddisp.pl?gene=HIBCH
ırddisp.pl?gene=E2F2
ırddisp.pl?gene=RPE65
ırddisp.pl?gene=CD69
ırddisp.pl?gene=CNC2
ırddisp.pl?gene=F10
ırddisp.pl?gene=HARS2
ırddisp.pl?gene=F8
ırddisp.pl?gene=TLR5
ırddisp.pl?gene=KMT2A
ırddisp.pl?gene=FANCG
ırddisp.pl?gene=SHOX2
ırddisp.pl?gene=FKBP10
ırddisp.pl?gene=PSEN1
ırddisp.pl?gene=IL7R
ırddisp.pl?gene=DCK
ırddisp.pl?gene=IL5
ırddisp.pl?gene=NGF
ırddisp.pl?gene=GLA
ırddisp.pl?gene=NFATC1
ırddisp.pl?gene=FGF13
ırddisp.pl?gene=BGN

ırddisp.pl?gene=FGA
ırddisp.pl?gene=DHCR7
ırddisp.pl?gene=ARL13B
ırddisp.pl?gene=HBA2
ırddisp.pl?gene=ABCG8
ırddisp.pl?gene=COL7A1
ırddisp.pl?gene=NIPBL
ırddisp.pl?gene=IFT88
ırddisp.pl?gene=PRLR
ırddisp.pl?gene=BUD23
ırddisp.pl?gene=ITGAM
ırddisp.pl?gene=MIR184
ırddisp.pl?gene=BLOC1S1
ırddisp.pl?gene=TNFRSF1B
ırddisp.pl?gene=SIM1
ırddisp.pl?gene=SLC6A4
ırddisp.pl?gene=HMOX1
ırddisp.pl?gene=TCTN2
ırddisp.pl?gene=CD27
ırddisp.pl?gene=CRHBP
ırddisp.pl?gene=GJB2
ırddisp.pl?gene=COL9A1
ırddisp.pl?gene=MIR486-1
ırddisp.pl?gene=RAG1
ırddisp.pl?gene=CLCN7
ırddisp.pl?gene=CYP27A1
ırddisp.pl?gene=CYB5A
ırddisp.pl?gene=HSPB2
ırddisp.pl?gene=TSPY1
ırddisp.pl?gene=CYP1A2
ırddisp.pl?gene=TNFRSF8
ırddisp.pl?gene=LMX1B
ırddisp.pl?gene=DCLRE1C
ırddisp.pl?gene=LFNG
ırddisp.pl?gene=LPA
ırddisp.pl?gene=SH2D1A
ırddisp.pl?gene=CFL1
ırddisp.pl?gene=PXN
ırddisp.pl?gene=MROH7
ırddisp.pl?gene=SAA1
ırddisp.pl?gene=TPM2
ırddisp.pl?gene=NPC1
ırddisp.pl?gene=NTRK2
ırddisp.pl?gene=ABCB7
ırddisp.pl?gene=CTNND1
ırddisp.pl?gene=NTS
ırddisp.pl?gene=GATA1
ırddisp.pl?gene=GRB2
ırddisp.pl?gene=MUS81
ırddisp.pl?gene=ASPM
ırddisp.pl?gene=IL15
ırddisp.pl?gene=RHEB
ırddisp.pl?gene=SREBF2
ırddisp.pl?gene=ARF1

ırddisp.pl?gene=ASS1
ırddisp.pl?gene=UGT1A6
ırddisp.pl?gene=IDH2
ırddisp.pl?gene=ARSH
ırddisp.pl?gene=MIR186
ırddisp.pl?gene=GFRA1
ırddisp.pl?gene=SALL1
ırddisp.pl?gene=LRP2
ırddisp.pl?gene=BBS1
ırddisp.pl?gene=GAST
ırddisp.pl?gene=HTR2C
ırddisp.pl?gene=CACNA1C
ırddisp.pl?gene=RSF1
ırddisp.pl?gene=NDN
ırddisp.pl?gene=CHRND
ırddisp.pl?gene=SYP
ırddisp.pl?gene=CAMSAP2
ırddisp.pl?gene=SMN1
ırddisp.pl?gene=SEMA3A
ırddisp.pl?gene=RECK
ırddisp.pl?gene=TNFRSF11A
ırddisp.pl?gene=DNAH1
ırddisp.pl?gene=AICDA
ırddisp.pl?gene=RBP4
ırddisp.pl?gene=ATF4
ırddisp.pl?gene=ARAF
ırddisp.pl?gene=GJA5
ırddisp.pl?gene=PML
ırddisp.pl?gene=TARDBP
ırddisp.pl?gene=CCL3
ırddisp.pl?gene=HERC1
ırddisp.pl?gene=KLLN
ırddisp.pl?gene=BBS5
ırddisp.pl?gene=GTF2H3
ırddisp.pl?gene=ACTG2
ırddisp.pl?gene=XDH
ırddisp.pl?gene=DHPS
ırddisp.pl?gene=EFHC2
ırddisp.pl?gene=GSK3B
ırddisp.pl?gene=TNFAIP3
ırddisp.pl?gene=IL13
ırddisp.pl?gene=ADRB2
ırddisp.pl?gene=MIR18A
ırddisp.pl?gene=SLC2A4
ırddisp.pl?gene=GAD2
ırddisp.pl?gene=IL1R1
ırddisp.pl?gene=GARS1
ırddisp.pl?gene=TGIF1
ırddisp.pl?gene=PKM
ırddisp.pl?gene=PXDN
ırddisp.pl?gene=MIR494
ırddisp.pl?gene=GK2
ırddisp.pl?gene=RMI2
ırddisp.pl?gene=RPS17

ırddisp.pl?gene=UGT1A8
ırddisp.pl?gene=BRD4
ırddisp.pl?gene=RNASET2
ırddisp.pl?gene=HES7
ırddisp.pl?gene=PPIA
ırddisp.pl?gene=LNPEP
ırddisp.pl?gene=CNTNAP1
ırddisp.pl?gene=AGER
ırddisp.pl?gene=TCF4
ırddisp.pl?gene=KRT13
ırddisp.pl?gene=RAD17
ırddisp.pl?gene=CEP57
ırddisp.pl?gene=DGUOK
ırddisp.pl?gene=IBSP
ırddisp.pl?gene=WFS1
ırddisp.pl?gene=ZIC2
ırddisp.pl?gene=NHEJ1
ırddisp.pl?gene=FOXK2
ırddisp.pl?gene=COQ8B
ırddisp.pl?gene=DHH
ırddisp.pl?gene=KPNA2
ırddisp.pl?gene=EIF2S1
ırddisp.pl?gene=GDAP1
ırddisp.pl?gene=LARS2-AS1
ırddisp.pl?gene=NODAL
ırddisp.pl?gene=PNPO
ırddisp.pl?gene=CFHR2
ırddisp.pl?gene=RMI1
ırddisp.pl?gene=MIR499A
ırddisp.pl?gene=B9D1
ırddisp.pl?gene=SSBP1
ırddisp.pl?gene=CDH11
ırddisp.pl?gene=KCND2
ırddisp.pl?gene=B9D2
ırddisp.pl?gene=WNT7A
ırddisp.pl?gene=CABIN1
ırddisp.pl?gene=C4A
ırddisp.pl?gene=SATB2
ırddisp.pl?gene=MDC1
ırddisp.pl?gene=IRS4
ırddisp.pl?gene=PHLDA2
ırddisp.pl?gene=RBX1
ırddisp.pl?gene=PBX1
ırddisp.pl?gene=RPS4Y1
ırddisp.pl?gene=BBS2
ırddisp.pl?gene=GPX3
ırddisp.pl?gene=OGG1
ırddisp.pl?gene=MIR15A
ırddisp.pl?gene=MMP13
ırddisp.pl?gene=MIR663AHG
ırddisp.pl?gene=CALM1
ırddisp.pl?gene=COX5A
ırddisp.pl?gene=KEAP1
ırddisp.pl?gene=AHI1

ırddisp.pl?gene=FAT4
ırddisp.pl?gene=TRAF6
ırddisp.pl?gene=SSTR2
ırddisp.pl?gene=DGKE
ırddisp.pl?gene=COL6A3
ırddisp.pl?gene=ACVR2A
ırddisp.pl?gene=SLC52A2
ırddisp.pl?gene=GLB1
ırddisp.pl?gene=RPS29
ırddisp.pl?gene=ADM
ırddisp.pl?gene=NEU1
ırddisp.pl?gene=TNFRSF6B
ırddisp.pl?gene=NFIX
ırddisp.pl?gene=PPP1R15A
ırddisp.pl?gene=FADD
ırddisp.pl?gene=AHR
ırddisp.pl?gene=CHRNA1
ırddisp.pl?gene=ACADS
ırddisp.pl?gene=PSMB8
ırddisp.pl?gene=SLC10A2
ırddisp.pl?gene=SIGLEC5
ırddisp.pl?gene=IL12RB1
ırddisp.pl?gene=LRBA
ırddisp.pl?gene=FDXR
ırddisp.pl?gene=OSGEP
ırddisp.pl?gene=CDC6
ırddisp.pl?gene=MPV17
ırddisp.pl?gene=GTF2H5
ırddisp.pl?gene=TLR3
ırddisp.pl?gene=GPT
ırddisp.pl?gene=COL4A4
ırddisp.pl?gene=EIF2S3
ırddisp.pl?gene=ALDH3A2
ırddisp.pl?gene=CACNA1H
ırddisp.pl?gene=KDM5C
ırddisp.pl?gene=DDB2
ırddisp.pl?gene=SDHB
ırddisp.pl?gene=AIFM1
ırddisp.pl?gene=AMACR
ırddisp.pl?gene=MIR20A
ırddisp.pl?gene=ALPI
ırddisp.pl?gene=FRAS1
ırddisp.pl?gene=SPINK5
ırddisp.pl?gene=LUC7L2
ırddisp.pl?gene=PDGFD
ırddisp.pl?gene=MLANA
ırddisp.pl?gene=CETP
ırddisp.pl?gene=JAK3
ırddisp.pl?gene=INPPL1
ırddisp.pl?gene=BBS4
ırddisp.pl?gene=NUP155
ırddisp.pl?gene=DSPP
ırddisp.pl?gene=RIPK1
ırddisp.pl?gene=AP3B1

ırddisp.pl?gene=TRIM5
ırddisp.pl?gene=MCPH1
ırddisp.pl?gene=IGFBP4
ırddisp.pl?gene=STAG1
ırddisp.pl?gene=PPP1R15B
ırddisp.pl?gene=APRT
ırddisp.pl?gene=CHRNB1
ırddisp.pl?gene=BBS9
ırddisp.pl?gene=SALL4
ırddisp.pl?gene=IGHE
ırddisp.pl?gene=ENO2
ırddisp.pl?gene=CAPN10
ırddisp.pl?gene=PRKAG2
ırddisp.pl?gene=PDLIM1
ırddisp.pl?gene=ACVR1
ırddisp.pl?gene=SLSN3
ırddisp.pl?gene=HEPACAM
ırddisp.pl?gene=APP
ırddisp.pl?gene=GCSH
ırddisp.pl?gene=EIF2AK4
ırddisp.pl?gene=LCT
ırddisp.pl?gene=MTHFD1
ırddisp.pl?gene=ARHGAP10
ırddisp.pl?gene=CRKL
ırddisp.pl?gene=PIK3CD
ırddisp.pl?gene=ARX
ırddisp.pl?gene=APOC3
ırddisp.pl?gene=NR4A1
ırddisp.pl?gene=STAT5B
ırddisp.pl?gene=LIG3
ırddisp.pl?gene=LAMA4
ırddisp.pl?gene=TARS1
ırddisp.pl?gene=CYP1B1
ırddisp.pl?gene=ADAR
ırddisp.pl?gene=ACADVL
ırddisp.pl?gene=CSH1
ırddisp.pl?gene=OTC
ırddisp.pl?gene=GLUL
ırddisp.pl?gene=PSEN2
ırddisp.pl?gene=EPOR
ırddisp.pl?gene=ATRIP
ırddisp.pl?gene=BMI1
ırddisp.pl?gene=NAA10
ırddisp.pl?gene=SCT
ırddisp.pl?gene=MT-CO3
ırddisp.pl?gene=CPOX
ırddisp.pl?gene=LAMB2
ırddisp.pl?gene=MYB
ırddisp.pl?gene=LIF
ırddisp.pl?gene=TIMM8A
ırddisp.pl?gene=APOH
ırddisp.pl?gene=IL2RB
ırddisp.pl?gene=POLR3B
ırddisp.pl?gene=CLPS

ırddisp.pl?gene=GBGT1
ırddisp.pl?gene=PTX3
ırddisp.pl?gene=FDX1
ırddisp.pl?gene=IMMT
ırddisp.pl?gene=GC
ırddisp.pl?gene=ERCC6L2
ırddisp.pl?gene=ACOT11
ırddisp.pl?gene=TCTN1
ırddisp.pl?gene=CEL
ırddisp.pl?gene=LTF
ırddisp.pl?gene=ITGAV
ırddisp.pl?gene=GCG
ırddisp.pl?gene=DGCR2
ırddisp.pl?gene=GATA2
ırddisp.pl?gene=TSPAN12
ırddisp.pl?gene=XBP1
ırddisp.pl?gene=COL6A1
ırddisp.pl?gene=SLC5A3
ırddisp.pl?gene=H6PD
ırddisp.pl?gene=PROKR2
ırddisp.pl?gene=EIF2S2
ırddisp.pl?gene=SLC26A4
ırddisp.pl?gene=TOR1A
ırddisp.pl?gene=MIR181A2
ırddisp.pl?gene=LGALS7
ırddisp.pl?gene=LEF1
ırddisp.pl?gene=NDUFS2
ırddisp.pl?gene=GNB4
ırddisp.pl?gene=STAC2
ırddisp.pl?gene=SCARB1
ırddisp.pl?gene=TUBB
ırddisp.pl?gene=COG2
ırddisp.pl?gene=SYNPO2
ırddisp.pl?gene=TNFSF13B
ırddisp.pl?gene=FGF17
ırddisp.pl?gene=NDUFA12
ırddisp.pl?gene=SERPINF1
ırddisp.pl?gene=NEK9
ırddisp.pl?gene=KIF3A
ırddisp.pl?gene=DPP4
ırddisp.pl?gene=LAGE3
ırddisp.pl?gene=TH2-LCR
ırddisp.pl?gene=SSTR1
ırddisp.pl?gene=COL4A5
ırddisp.pl?gene=KRT19
ırddisp.pl?gene=NDUFA9
ırddisp.pl?gene=CASP1
ırddisp.pl?gene=MKI67
ırddisp.pl?gene=FAM124B
ırddisp.pl?gene=NUP85
ırddisp.pl?gene=SSTR3
ırddisp.pl?gene=LCK
ırddisp.pl?gene=CST6
ırddisp.pl?gene=KRT17

ırddisp.pl?gene=ATCAY
ırddisp.pl?gene=MAD2L2
ırddisp.pl?gene=CD79A
ırddisp.pl?gene=HSD3B1
ırddisp.pl?gene=CCL11
ırddisp.pl?gene=STRADB
ırddisp.pl?gene=MIR203A
ırddisp.pl?gene=LIPE
ırddisp.pl?gene=SERPINA6
ırddisp.pl?gene=CNTLN
ırddisp.pl?gene=GLI1
ırddisp.pl?gene=SRD5A1
ırddisp.pl?gene=DBH
ırddisp.pl?gene=MAPT
ırddisp.pl?gene=GALE
ırddisp.pl?gene=AKAP6
ırddisp.pl?gene=SSTR5
ırddisp.pl?gene=ABCA4
ırddisp.pl?gene=ASPA
ırddisp.pl?gene=KL
ırddisp.pl?gene=CSH2
ırddisp.pl?gene=UNC13D
ırddisp.pl?gene=ACHE
ırddisp.pl?gene=HTR2A
ırddisp.pl?gene=EIF4G1
ırddisp.pl?gene=CCNH
ırddisp.pl?gene=RPS20
ırddisp.pl?gene=CAVIN1
ırddisp.pl?gene=BAG5
ırddisp.pl?gene=GAL
ırddisp.pl?gene=CLCN1
ırddisp.pl?gene=ABCC3
ırddisp.pl?gene=COL6A2
ırddisp.pl?gene=LGALS7B
ırddisp.pl?gene=UGDH
ırddisp.pl?gene=MRPL44
ırddisp.pl?gene=GALM
ırddisp.pl?gene=UGP2
ırddisp.pl?gene=UAP1
ırddisp.pl?gene=GALK2
ırddisp.pl?gene=CLC
ırddisp.pl?gene=TCF15
ırddisp.pl?gene=MST1R
ırddisp.pl?gene=ITGAL
ırddisp.pl?gene=IFT20
ırddisp.pl?gene=ALOXE3
ırddisp.pl?gene=MATK
ırddisp.pl?gene=XPC
ırddisp.pl?gene=MTR
ırddisp.pl?gene=ALOX12B
ırddisp.pl?gene=PPP1CB
ırddisp.pl?gene=CDC7
ırddisp.pl?gene=SMCR8
ırddisp.pl?gene=EIF5AL1

ırddisp.pl?gene=SCGB1A1
ırddisp.pl?gene=HSD17B1
ırddisp.pl?gene=VRK2
ırddisp.pl?gene=FGR
ırddisp.pl?gene=PSMB5
ırddisp.pl?gene=CHGA
ırddisp.pl?gene=IL12B
ırddisp.pl?gene=INSL3
ırddisp.pl?gene=PDGFB
ırddisp.pl?gene=CTSA
ırddisp.pl?gene=C2CD3
ırddisp.pl?gene=P2RY12
ırddisp.pl?gene=SKI
ırddisp.pl?gene=LDLRAP1
ırddisp.pl?gene=GLDC
ırddisp.pl?gene=HSPA8
ırddisp.pl?gene=SGCE
ırddisp.pl?gene=POLD1
ırddisp.pl?gene=TRPV6
ırddisp.pl?gene=GSR
ırddisp.pl?gene=COL17A1
ırddisp.pl?gene=CBS
ırddisp.pl?gene=EGR2
ırddisp.pl?gene=BMP1
ırddisp.pl?gene=SSH1
ırddisp.pl?gene=DLG4
ırddisp.pl?gene=GDF5
ırddisp.pl?gene=CCR5
ırddisp.pl?gene=FKBP1A
ırddisp.pl?gene=MYOCD
ırddisp.pl?gene=CDH3
ırddisp.pl?gene=HNRNPA2B1
ırddisp.pl?gene=FXN
ırddisp.pl?gene=APLN
ırddisp.pl?gene=PLOD2
ırddisp.pl?gene=GSC
ırddisp.pl?gene=AGTR2
ırddisp.pl?gene=CLDN11
ırddisp.pl?gene=ANKRD49
ırddisp.pl?gene=UBE2G1
ırddisp.pl?gene=HSPA12A
ırddisp.pl?gene=TYR
ırddisp.pl?gene=UPF1
ırddisp.pl?gene=MC4R
ırddisp.pl?gene=OSGIN2
ırddisp.pl?gene=MAS1L
ırddisp.pl?gene=COQ2
ırddisp.pl?gene=CDH13
ırddisp.pl?gene=TNFSF12
ırddisp.pl?gene=NUP153
ırddisp.pl?gene=SMARCD2
ırddisp.pl?gene=NCAM1
ırddisp.pl?gene=GSTT1
ırddisp.pl?gene=POLB

ırddisp.pl?gene=CENPI
ırddisp.pl?gene=STAT4
ırddisp.pl?gene=BIRC2
ırddisp.pl?gene=LOC110806262
ırddisp.pl?gene=TTC30B
ırddisp.pl?gene=AKAP13
ırddisp.pl?gene=SACS
ırddisp.pl?gene=EIF5
ırddisp.pl?gene=POLR3K
ırddisp.pl?gene=BCL7B
ırddisp.pl?gene=HLA-DQA1
ırddisp.pl?gene=BBS12
ırddisp.pl?gene=SMARCAL1
ırddisp.pl?gene=CAMP
ırddisp.pl?gene=MT-TF
ırddisp.pl?gene=MIR196A2
ırddisp.pl?gene=ACOT7
ırddisp.pl?gene=EIF1
ırddisp.pl?gene=UFD1
ırddisp.pl?gene=TRIP11
ırddisp.pl?gene=SDC1
ırddisp.pl?gene=TSEN34
ırddisp.pl?gene=SCYL1
ırddisp.pl?gene=PDIA4
ırddisp.pl?gene=KCNN4
ırddisp.pl?gene=WNT3A
ırddisp.pl?gene=PGK1
ırddisp.pl?gene=PCTP
ırddisp.pl?gene=ACOT12
ırddisp.pl?gene=SYNGR2
ırddisp.pl?gene=LRIT1
ırddisp.pl?gene=SPTSSB
ırddisp.pl?gene=KRTAP9-9
ırddisp.pl?gene=CCNT2
ırddisp.pl?gene=MRPS34
ırddisp.pl?gene=RCC1L
ırddisp.pl?gene=DDI1
ırddisp.pl?gene=SLC2A2
ırddisp.pl?gene=SLC12A6
ırddisp.pl?gene=USF1
ırddisp.pl?gene=VANGL1
ırddisp.pl?gene=APAF1
ırddisp.pl?gene=ARHGAP31
ırddisp.pl?gene=IL33
ırddisp.pl?gene=CYP24A1
ırddisp.pl?gene=LOC108964933
ırddisp.pl?gene=CA2
ırddisp.pl?gene=TRAPPC10
ırddisp.pl?gene=MIR675
ırddisp.pl?gene=MICA
ırddisp.pl?gene=SDHD
ırddisp.pl?gene=AKAP1
ırddisp.pl?gene=CD81
ırddisp.pl?gene=UBR4

rddisp.pl?gene=TOP3A
rddisp.pl?gene=HTR1A
rddisp.pl?gene=MIR10A
rddisp.pl?gene=NLRP7
rddisp.pl?gene=GLUD1
rddisp.pl?gene=MAGI2
rddisp.pl?gene=VEGFD
rddisp.pl?gene=PRDM16
rddisp.pl?gene=GHSR
rddisp.pl?gene=ADAMTS4
rddisp.pl?gene=AMER1
rddisp.pl?gene=DACT1
rddisp.pl?gene=DDB1
rddisp.pl?gene=RASA1
rddisp.pl?gene=NR1I2
rddisp.pl?gene=FAM111A
rddisp.pl?gene=NFATC2
rddisp.pl?gene=STAG2
rddisp.pl?gene=SDHC
rddisp.pl?gene=CEP104
rddisp.pl?gene=MIR423
rddisp.pl?gene=TCN1
rddisp.pl?gene=MB
rddisp.pl?gene=SPAST
rddisp.pl?gene=OXTR
rddisp.pl?gene=LINC01554
rddisp.pl?gene=MT-TH
rddisp.pl?gene=UBB
rddisp.pl?gene=C9orf72
rddisp.pl?gene=CD80
rddisp.pl?gene=XRCC4
rddisp.pl?gene=SSTR4
rddisp.pl?gene=PPP1R1B
rddisp.pl?gene=GRIA1
rddisp.pl?gene=CAMK4
rddisp.pl?gene=SOX8
rddisp.pl?gene=CLTCL1
rddisp.pl?gene=AKT1S1
rddisp.pl?gene=AQP4
rddisp.pl?gene=RCAN1
rddisp.pl?gene=EIF6
rddisp.pl?gene=DDIT3
rddisp.pl?gene=MLKL
rddisp.pl?gene=H19-ICR
rddisp.pl?gene=DYNC2H1
rddisp.pl?gene=DPYD
rddisp.pl?gene=CYP2D6
rddisp.pl?gene=GBA2
rddisp.pl?gene=PIK3C2A
rddisp.pl?gene=RPS6KB2
rddisp.pl?gene=RUNX1
rddisp.pl?gene=UNG
rddisp.pl?gene=CCP110
rddisp.pl?gene=CEP97

ırddisp.pl?gene=GGT2
ırddisp.pl?gene=GGTLC3
ırddisp.pl?gene=TTC16
ırddisp.pl?gene=YBX1
ırddisp.pl?gene=LAMA3
ırddisp.pl?gene=FBN2
ırddisp.pl?gene=LOXL1
ırddisp.pl?gene=APOA4
ırddisp.pl?gene=SHANK3
ırddisp.pl?gene=ABHD12
ırddisp.pl?gene=KNG1
ırddisp.pl?gene=SLC27A2
ırddisp.pl?gene=KRT8
ırddisp.pl?gene=UGCG
ırddisp.pl?gene=PHF8
ırddisp.pl?gene=ADCY1
ırddisp.pl?gene=GET1
ırddisp.pl?gene=VILL
ırddisp.pl?gene=PAM16
ırddisp.pl?gene=ITGAX
ırddisp.pl?gene=LGALS1
ırddisp.pl?gene=RAB5A
ırddisp.pl?gene=DKK1
ırddisp.pl?gene=IRF4
ırddisp.pl?gene=CDK7
ırddisp.pl?gene=HLCS
ırddisp.pl?gene=RHBDF2
ırddisp.pl?gene=RAB3GAP1
ırddisp.pl?gene=ITGA4
ırddisp.pl?gene=BCOR
ırddisp.pl?gene=BLZF1
ırddisp.pl?gene=LHX1
ırddisp.pl?gene=PMM1
ırddisp.pl?gene=IL1RAPL2
ırddisp.pl?gene=HHIP
ırddisp.pl?gene=FERD3L
ırddisp.pl?gene=ITGB2
ırddisp.pl?gene=GNAQ
ırddisp.pl?gene=IL16
ırddisp.pl?gene=ITPR1
ırddisp.pl?gene=NOTCH4
ırddisp.pl?gene=COL10A1
ırddisp.pl?gene=PHOX2B
ırddisp.pl?gene=MAPK10
ırddisp.pl?gene=RIPK3
ırddisp.pl?gene=FAF1
ırddisp.pl?gene=DHCR24
ırddisp.pl?gene=PF4
ırddisp.pl?gene=DYRK1A
ırddisp.pl?gene=FUS
ırddisp.pl?gene=STRADA
ırddisp.pl?gene=MIR33A
ırddisp.pl?gene=S100A1
ırddisp.pl?gene=CLDN10

ırddisp.pl?gene=MIR330
ırddisp.pl?gene=SUN2
ırddisp.pl?gene=NT5C
ırddisp.pl?gene=PC
ırddisp.pl?gene=CEBPB
ırddisp.pl?gene=CD86
ırddisp.pl?gene=MMD
ırddisp.pl?gene=IFNGR1
ırddisp.pl?gene=AGA
ırddisp.pl?gene=MUC2
ırddisp.pl?gene=MIR122
ırddisp.pl?gene=RPGR
ırddisp.pl?gene=GJB1
ırddisp.pl?gene=FCN2
ırddisp.pl?gene=GFI1
ırddisp.pl?gene=ASL
ırddisp.pl?gene=HS6ST1
ırddisp.pl?gene=CEACAM16
ırddisp.pl?gene=HJV
ırddisp.pl?gene=PPP3R1
ırddisp.pl?gene=PPP3CB
ırddisp.pl?gene=IFT57
ırddisp.pl?gene=TRPV5
ırddisp.pl?gene=PPIB
ırddisp.pl?gene=POLR2L
ırddisp.pl?gene=CTTN
ırddisp.pl?gene=NFATC4
ırddisp.pl?gene=HPS3
ırddisp.pl?gene=ESD
ırddisp.pl?gene=RPL10
ırddisp.pl?gene=PPP6C
ırddisp.pl?gene=ALDH2
ırddisp.pl?gene=SLC6A3
ırddisp.pl?gene=DCX
ırddisp.pl?gene=APC2
ırddisp.pl?gene=HLA-G
ırddisp.pl?gene=PPP3CC
ırddisp.pl?gene=NFATC3
ırddisp.pl?gene=AKAP5
ırddisp.pl?gene=AKAP7
ırddisp.pl?gene=C1orf122
ırddisp.pl?gene=CXCR2
ırddisp.pl?gene=EBAG9
ırddisp.pl?gene=CALR
ırddisp.pl?gene=AQP1
ırddisp.pl?gene=PI3
ırddisp.pl?gene=SMARCA5
ırddisp.pl?gene=ALG9
ırddisp.pl?gene=AQP3
ırddisp.pl?gene=ACTG1
ırddisp.pl?gene=MEST
ırddisp.pl?gene=LAMB3
ırddisp.pl?gene=KRT10
ırddisp.pl?gene=ACVR1B

ırddisp.pl?gene=CHI3L1
ırddisp.pl?gene=CCT3
ırddisp.pl?gene=SLC30A5
ırddisp.pl?gene=ARFGAP3
ırddisp.pl?gene=SLC30A7
ırddisp.pl?gene=ATP5MD
ırddisp.pl?gene=CARD11
ırddisp.pl?gene=VCAN
ırddisp.pl?gene=ARFGAP1
ırddisp.pl?gene=RAB3IL1
ırddisp.pl?gene=ARFGAP2
ırddisp.pl?gene=AHCTF1
ırddisp.pl?gene=NUP43
ırddisp.pl?gene=TMEM120A
ırddisp.pl?gene=DHX9
ırddisp.pl?gene=RPGRIP1
ırddisp.pl?gene=VEGFB
ırddisp.pl?gene=GPR108
ırddisp.pl?gene=LRRC56
ırddisp.pl?gene=PYY
ırddisp.pl?gene=SAT1
ırddisp.pl?gene=SLC25A13
ırddisp.pl?gene=SPG21
ırddisp.pl?gene=ZFP57
ırddisp.pl?gene=CHRNA3
ırddisp.pl?gene=SPATA7
ırddisp.pl?gene=ABCG1
ırddisp.pl?gene=BTK
ırddisp.pl?gene=SGPL1
ırddisp.pl?gene=OPRM1
ırddisp.pl?gene=CDKN2C
ırddisp.pl?gene=SLC11A1
ırddisp.pl?gene=ZFYVE26
ırddisp.pl?gene=SNCG
ırddisp.pl?gene=PLEK
ırddisp.pl?gene=SNURF
ırddisp.pl?gene=HPR
ırddisp.pl?gene=CCL4
ırddisp.pl?gene=CHUK
ırddisp.pl?gene=ICMT
ırddisp.pl?gene=PRDM1
ırddisp.pl?gene=CTSG
ırddisp.pl?gene=ADCY10
ırddisp.pl?gene=ALK
ırddisp.pl?gene=ANAPC1
ırddisp.pl?gene=HNRNPH1
ırddisp.pl?gene=SMARCA1
ırddisp.pl?gene=COL27A1
ırddisp.pl?gene=TGFBR3
ırddisp.pl?gene=SETX
ırddisp.pl?gene=HOXD9
ırddisp.pl?gene=FCGR2A
ırddisp.pl?gene=MYH9
ırddisp.pl?gene=IL11RA

ırddisp.pl?gene=DDX41
ırddisp.pl?gene=IDO1
ırddisp.pl?gene=RHOD
ırddisp.pl?gene=MYCN
ırddisp.pl?gene=POLR2A
ırddisp.pl?gene=CITED2
ırddisp.pl?gene=BAD
ırddisp.pl?gene=ODC1
ırddisp.pl?gene=MMAA
ırddisp.pl?gene=CBX5
ırddisp.pl?gene=CCR2
ırddisp.pl?gene=DIO2
ırddisp.pl?gene=HUWE1
ırddisp.pl?gene=PSTPIP1
ırddisp.pl?gene=CCL27
ırddisp.pl?gene=B3GALT4
ırddisp.pl?gene=SUN1
ırddisp.pl?gene=TLR6
ırddisp.pl?gene=CXCL13
ırddisp.pl?gene=PIK3R4
ırddisp.pl?gene=UCP1
ırddisp.pl?gene=NAGLU
ırddisp.pl?gene=BCL6
ırddisp.pl?gene=CTCFL
ırddisp.pl?gene=NLRP2
ırddisp.pl?gene=PEG3
ırddisp.pl?gene=PDE11A
ırddisp.pl?gene=SGK1
ırddisp.pl?gene=H1-1
ırddisp.pl?gene=MATN1
ırddisp.pl?gene=CCN1
ırddisp.pl?gene=ACCS
ırddisp.pl?gene=ZNRF3
ırddisp.pl?gene=PRICKLE4
ırddisp.pl?gene=RTP1
ırddisp.pl?gene=XAGE2
ırddisp.pl?gene=SLC29A1
ırddisp.pl?gene=NCF1
ırddisp.pl?gene=MIR379
ırddisp.pl?gene=HOXA13
ırddisp.pl?gene=ABHD5
ırddisp.pl?gene=PON2
ırddisp.pl?gene=SLC33A1
ırddisp.pl?gene=CUL1
ırddisp.pl?gene=CSN1S1
ırddisp.pl?gene=FBL
ırddisp.pl?gene=OTX2
ırddisp.pl?gene=PAX5
ırddisp.pl?gene=WEE1
ırddisp.pl?gene=CYP2C19
ırddisp.pl?gene=PGAP1
ırddisp.pl?gene=XPNPEP3
ırddisp.pl?gene=TSACC
ırddisp.pl?gene=NFIB

ırddisp.pl?gene=KDM1A
ırddisp.pl?gene=IVD
ırddisp.pl?gene=NF2
ırddisp.pl?gene=HOXB2
ırddisp.pl?gene=EIF2AK3
ırddisp.pl?gene=HNRNPH2
ırddisp.pl?gene=UPF3B
ırddisp.pl?gene=STAT5A
ırddisp.pl?gene=NGFR
ırddisp.pl?gene=TLR7
ırddisp.pl?gene=TBX4
ırddisp.pl?gene=CD59
ırddisp.pl?gene=LAMP1
ırddisp.pl?gene=G6PC
ırddisp.pl?gene=FGF6
ırddisp.pl?gene=RPS6
ırddisp.pl?gene=BTD
ırddisp.pl?gene=ADAMTSL1
ırddisp.pl?gene=LMOD1
ırddisp.pl?gene=PHF1
ırddisp.pl?gene=FMR1-AS1
ırddisp.pl?gene=DEFB1
ırddisp.pl?gene=IKZF1
ırddisp.pl?gene=ITGA2
ırddisp.pl?gene=MAD2L1
ırddisp.pl?gene=CIITA
ırddisp.pl?gene=FNDC5
ırddisp.pl?gene=REEP1
ırddisp.pl?gene=SYNGAP1
ırddisp.pl?gene=CSF3R
ırddisp.pl?gene=PDPK1
ırddisp.pl?gene=TUBB3
ırddisp.pl?gene=BAK1
ırddisp.pl?gene=SLC4A1
ırddisp.pl?gene=KHDC3L
ırddisp.pl?gene=VAV1
ırddisp.pl?gene=FOXC2
ırddisp.pl?gene=NSD2
ırddisp.pl?gene=DMWD
ırddisp.pl?gene=CYBA
ırddisp.pl?gene=SIRT2
ırddisp.pl?gene=HCRT
ırddisp.pl?gene=RLN1
ırddisp.pl?gene=UFSP2
ırddisp.pl?gene=NPR2
ırddisp.pl?gene=TNFRSF17
ırddisp.pl?gene=HLA-C
ırddisp.pl?gene=KCNJ5
ırddisp.pl?gene=ADD1
ırddisp.pl?gene=RPS6KA1
ırddisp.pl?gene=NSDHL
ırddisp.pl?gene=EMG1
ırddisp.pl?gene=TAC3
ırddisp.pl?gene=PPARGC1A

ırddisp.pl?gene=CR1
ırddisp.pl?gene=MMADHC
ırddisp.pl?gene=CCT7
ırddisp.pl?gene=CD47
ırddisp.pl?gene=DYNC1H1
ırddisp.pl?gene=KHDRBS3
ırddisp.pl?gene=CUL4A
ırddisp.pl?gene=UTRN
ırddisp.pl?gene=KIF1C
ırddisp.pl?gene=ARMC5
ırddisp.pl?gene=NT5C1B
ırddisp.pl?gene=CD163
ırddisp.pl?gene=ARG1
ırddisp.pl?gene=GANAB
ırddisp.pl?gene=PAM
ırddisp.pl?gene=TMLHE
ırddisp.pl?gene=TULP1
ırddisp.pl?gene=CSTB
ırddisp.pl?gene=IL18R1
ırddisp.pl?gene=EHMT2
ırddisp.pl?gene=PRKAA2
ırddisp.pl?gene=RARS1
ırddisp.pl?gene=ARSL
ırddisp.pl?gene=STAT6
ırddisp.pl?gene=FCGR3A
ırddisp.pl?gene=DSG2
ırddisp.pl?gene=PYGM
ırddisp.pl?gene=CD14
ırddisp.pl?gene=PDSS1
ırddisp.pl?gene=BCAP31
ırddisp.pl?gene=EPHX2
ırddisp.pl?gene=TLR1
ırddisp.pl?gene=RBFOX2
ırddisp.pl?gene=HCFC1
ırddisp.pl?gene=ATP6AP1
ırddisp.pl?gene=TFEB
ırddisp.pl?gene=PLA2G2A
ırddisp.pl?gene=DMC1
ırddisp.pl?gene=RPS14
ırddisp.pl?gene=RUNX3
ırddisp.pl?gene=TMPO
ırddisp.pl?gene=PTPN14
ırddisp.pl?gene=PLA2G7
ırddisp.pl?gene=MIR16-1
ırddisp.pl?gene=NR1I3
ırddisp.pl?gene=CHRNE
ırddisp.pl?gene=GLS
ırddisp.pl?gene=SFTPD
ırddisp.pl?gene=KCNE1
ırddisp.pl?gene=IL23R
ırddisp.pl?gene=FABP3
ırddisp.pl?gene=TLR8
ırddisp.pl?gene=SYNE3
ırddisp.pl?gene=GJA4

ırddisp.pl?gene=COL18A1
ırddisp.pl?gene=CD63
ırddisp.pl?gene=CEBPD
ırddisp.pl?gene=ADCYAP1
ırddisp.pl?gene=GALNS
ırddisp.pl?gene=MNX1
ırddisp.pl?gene=PTRH2
ırddisp.pl?gene=FGFRL1
ırddisp.pl?gene=F2RL1
ırddisp.pl?gene=MVK
ırddisp.pl?gene=MASP2
ırddisp.pl?gene=BID
ırddisp.pl?gene=SAG
ırddisp.pl?gene=TFAM
ırddisp.pl?gene=NSUN2
ırddisp.pl?gene=SMAD1
ırddisp.pl?gene=PLK4
ırddisp.pl?gene=MUTYH
ırddisp.pl?gene=GHRHR
ırddisp.pl?gene=VHL
ırddisp.pl?gene=EPHA4
ırddisp.pl?gene=MIR30C1
ırddisp.pl?gene=SREBF1
ırddisp.pl?gene=COL4A3
ırddisp.pl?gene=TRPS1
ırddisp.pl?gene=VMA21
ırddisp.pl?gene=TJP1
ırddisp.pl?gene=CCR7
ırddisp.pl?gene=SULT2A1
ırddisp.pl?gene=UTP4
ırddisp.pl?gene=SPART
ırddisp.pl?gene=ETV6
ırddisp.pl?gene=CCR3
ırddisp.pl?gene=LYST
ırddisp.pl?gene=SIRT3
ırddisp.pl?gene=RAB7A
ırddisp.pl?gene=ASCL1
ırddisp.pl?gene=NCOR1
ırddisp.pl?gene=ANOS1
ırddisp.pl?gene=REEP4
ırddisp.pl?gene=FTO
ırddisp.pl?gene=FGF18
ırddisp.pl?gene=ADAMTSL4
ırddisp.pl?gene=MTNR1B
ırddisp.pl?gene=ELOVL1
ırddisp.pl?gene=EDA2R
ırddisp.pl?gene=NLRP6
ırddisp.pl?gene=DNASE1
ırddisp.pl?gene=MC1R
ırddisp.pl?gene=FUCA1
ırddisp.pl?gene=SEC23B
ırddisp.pl?gene=CORIN
ırddisp.pl?gene=SMO
ırddisp.pl?gene=ITGA3

ırddisp.pl?gene=PAFAH1B1
ırddisp.pl?gene=FA2H
ırddisp.pl?gene=CYP2C9
ırddisp.pl?gene=XPO1
ırddisp.pl?gene=CISD2
ırddisp.pl?gene=NOS1
ırddisp.pl?gene=IL31RA
ırddisp.pl?gene=KLF4
ırddisp.pl?gene=HMGCL
ırddisp.pl?gene=BCKDK
ırddisp.pl?gene=GNPTG
ırddisp.pl?gene=IMPDH1
ırddisp.pl?gene=COL4A1
ırddisp.pl?gene=MIR374B
ırddisp.pl?gene=DNMT3L
ırddisp.pl?gene=LAMA1
ırddisp.pl?gene=ESPL1
ırddisp.pl?gene=TNNI2
ırddisp.pl?gene=MIR500A
ırddisp.pl?gene=M6PR
ırddisp.pl?gene=MTHFS
ırddisp.pl?gene=MAP3K7
ırddisp.pl?gene=NAT10
ırddisp.pl?gene=VARS1
ırddisp.pl?gene=THADA
ırddisp.pl?gene=CHKA
ırddisp.pl?gene=LRP6
ırddisp.pl?gene=LTBP1
ırddisp.pl?gene=KARS1
ırddisp.pl?gene=STOX1
ırddisp.pl?gene=IVL
ırddisp.pl?gene=SLC5A5
ırddisp.pl?gene=TAP2
ırddisp.pl?gene=ARNTL
ırddisp.pl?gene=CDK8
ırddisp.pl?gene=DNM1L
ırddisp.pl?gene=HSPB8
ırddisp.pl?gene=SPRY2
ırddisp.pl?gene=RAC2
ırddisp.pl?gene=AKAP12
ırddisp.pl?gene=PIK3R2
ırddisp.pl?gene=S100A8
ırddisp.pl?gene=NTF3
ırddisp.pl?gene=UBE2T
ırddisp.pl?gene=BMP7
ırddisp.pl?gene=UBE2D1
ırddisp.pl?gene=GGCT
ırddisp.pl?gene=PLCE1
ırddisp.pl?gene=CEACAM6
ırddisp.pl?gene=FCN3
ırddisp.pl?gene=MIR378A
ırddisp.pl?gene=LONP1
ırddisp.pl?gene=WNT11
ırddisp.pl?gene=JAZF1

ırddisp.pl?gene=IRAK1
ırddisp.pl?gene=PDGFA
ırddisp.pl?gene=PROCR
ırddisp.pl?gene=AXIN2
ırddisp.pl?gene=GGCX
ırddisp.pl?gene=RBL2
ırddisp.pl?gene=TNFRSF25
ırddisp.pl?gene=CRHR2
ırddisp.pl?gene=TACR3
ırddisp.pl?gene=GMNN
ırddisp.pl?gene=ARHGAP32
ırddisp.pl?gene=CSTA
ırddisp.pl?gene=CRB1
ırddisp.pl?gene=PRPH2
ırddisp.pl?gene=ELAVL4
ırddisp.pl?gene=ELAVL3
ırddisp.pl?gene=REEP2
ırddisp.pl?gene=RPS3
ırddisp.pl?gene=ENPP2
ırddisp.pl?gene=CRX
ırddisp.pl?gene=CHRNG
ırddisp.pl?gene=GRPR
ırddisp.pl?gene=HYMAI
ırddisp.pl?gene=GMPS
ırddisp.pl?gene=RDH12
ırddisp.pl?gene=TOPBP1
ırddisp.pl?gene=RPTOR
ırddisp.pl?gene=IGHM
ırddisp.pl?gene=SIRT6
ırddisp.pl?gene=EPAS1
ırddisp.pl?gene=CYP2E1
ırddisp.pl?gene=IL21
ırddisp.pl?gene=MYO5A
ırddisp.pl?gene=SMC4
ırddisp.pl?gene=CD52
ırddisp.pl?gene=HK2
ırddisp.pl?gene=SHMT1
ırddisp.pl?gene=FOXC1
ırddisp.pl?gene=LRP1
ırddisp.pl?gene=ARSA
ırddisp.pl?gene=CFB
ırddisp.pl?gene=MEF2A
ırddisp.pl?gene=S100A9
ırddisp.pl?gene=CD9
ırddisp.pl?gene=PDZK1IP1
ırddisp.pl?gene=CD68
ırddisp.pl?gene=NAT2
ırddisp.pl?gene=PROC
ırddisp.pl?gene=JAM3
ırddisp.pl?gene=FAM161A
ırddisp.pl?gene=DCTN1
ırddisp.pl?gene=DNA2
ırddisp.pl?gene=MMAB
ırddisp.pl?gene=HSD17B2

ırddisp.pl?gene=KMT2B
ırddisp.pl?gene=GATA5
ırddisp.pl?gene=TRIM27
ırddisp.pl?gene=YWHAQ
ırddisp.pl?gene=RBBP4
ırddisp.pl?gene=PRKAA1
ırddisp.pl?gene=ERVW-1
ırddisp.pl?gene=NUP88
ırddisp.pl?gene=PRSS2
ırddisp.pl?gene=MYOG
ırddisp.pl?gene=SUV39H1
ırddisp.pl?gene=CNN1
ırddisp.pl?gene=HTRA2
ırddisp.pl?gene=ITGA6
ırddisp.pl?gene=IRF1
ırddisp.pl?gene=SLC9A3R1
ırddisp.pl?gene=ACP1
ırddisp.pl?gene=TACR1
ırddisp.pl?gene=SPRY1
ırddisp.pl?gene=BIRC3
ırddisp.pl?gene=YY1
ırddisp.pl?gene=ATP2A2
ırddisp.pl?gene=TFAP2B
ırddisp.pl?gene=FKBP14
ırddisp.pl?gene=TCF3
ırddisp.pl?gene=HBG1
ırddisp.pl?gene=INPP5J
ırddisp.pl?gene=GUCY2D
ırddisp.pl?gene=TCEA1
ırddisp.pl?gene=USH2A
ırddisp.pl?gene=COL9A2
ırddisp.pl?gene=ABO
ırddisp.pl?gene=PDE4D
ırddisp.pl?gene=ACAT1
ırddisp.pl?gene=CNR1
ırddisp.pl?gene=IL11
ırddisp.pl?gene=EDN2
ırddisp.pl?gene=CPT1A
ırddisp.pl?gene=PLA2G10
ırddisp.pl?gene=RUNX1T1
ırddisp.pl?gene=C4B
ırddisp.pl?gene=LCA5
ırddisp.pl?gene=TSPAN32
ırddisp.pl?gene=ADAM17
ırddisp.pl?gene=KRT1
ırddisp.pl?gene=MAOA
ırddisp.pl?gene=PCMT1
ırddisp.pl?gene=HNRNPA1
ırddisp.pl?gene=HSPB6
ırddisp.pl?gene=S100A4
ırddisp.pl?gene=PTER
ırddisp.pl?gene=SRSF6
ırddisp.pl?gene=FCGR2B
ırddisp.pl?gene=RICTOR

ırddisp.pl?gene=ACADSB
ırddisp.pl?gene=MCEE
ırddisp.pl?gene=AIPL1
ırddisp.pl?gene=MYCBP2
ırddisp.pl?gene=LMBRD1
ırddisp.pl?gene=EYA3
ırddisp.pl?gene=POC1B
ırddisp.pl?gene=POLD4
ırddisp.pl?gene=GGACT
ırddisp.pl?gene=CCDC92
ırddisp.pl?gene=MAK16
ırddisp.pl?gene=RHNO1
ırddisp.pl?gene=OR51S1
ırddisp.pl?gene=FAAP24
ırddisp.pl?gene=CD151
ırddisp.pl?gene=DSC2
ırddisp.pl?gene=SERPINF2
ırddisp.pl?gene=ACACA
ırddisp.pl?gene=RYR3
ırddisp.pl?gene=ATXN2
ırddisp.pl?gene=HSD17B3
ırddisp.pl?gene=PRKCB
ırddisp.pl?gene=ALG8
ırddisp.pl?gene=FURIN
ırddisp.pl?gene=PIKFYVE
ırddisp.pl?gene=LOC110806263
ırddisp.pl?gene=NPPC
ırddisp.pl?gene=CD58
ırddisp.pl?gene=FABP4
ırddisp.pl?gene=FABP5
ırddisp.pl?gene=MIR19B1
ırddisp.pl?gene=HDAC6
ırddisp.pl?gene=TFF3
ırddisp.pl?gene=GCN1
ırddisp.pl?gene=TRPC3
ırddisp.pl?gene=MMP10
ırddisp.pl?gene=MAP2K7
ırddisp.pl?gene=RPS27A
ırddisp.pl?gene=RRM1
ırddisp.pl?gene=GAS6
ırddisp.pl?gene=LGALS4
ırddisp.pl?gene=THRB
ırddisp.pl?gene=PSMB9
ırddisp.pl?gene=SLAMF1
ırddisp.pl?gene=DDR1
ırddisp.pl?gene=MIR154
ırddisp.pl?gene=MICU1
ırddisp.pl?gene=PPIG
ırddisp.pl?gene=FRS2
ırddisp.pl?gene=SOCS1
ırddisp.pl?gene=MIR1-2
ırddisp.pl?gene=TARS2
ırddisp.pl?gene=PMEL
ırddisp.pl?gene=TRIP13

ırddisp.pl?gene=ADIPOR1
ırddisp.pl?gene=STMN1
ırddisp.pl?gene=PITX2
ırddisp.pl?gene=RAB6A
ırddisp.pl?gene=CPLANE1
ırddisp.pl?gene=ADNP
ırddisp.pl?gene=HAX1
ırddisp.pl?gene=ADH1B
ırddisp.pl?gene=FOLR3
ırddisp.pl?gene=VAMP2
ırddisp.pl?gene=FLT3
ırddisp.pl?gene=MDK
ırddisp.pl?gene=CASP14
ırddisp.pl?gene=SIRT7
ırddisp.pl?gene=HEY2
ırddisp.pl?gene=EFTUD2
ırddisp.pl?gene=EPHB1
ırddisp.pl?gene=MIR382
ırddisp.pl?gene=CDT1
ırddisp.pl?gene=HNMT
ırddisp.pl?gene=NPRL3
ırddisp.pl?gene=NUPR1
ırddisp.pl?gene=SORT1
ırddisp.pl?gene=TRPM7
ırddisp.pl?gene=SPTLC2
ırddisp.pl?gene=TCEA3
ırddisp.pl?gene=REG3A
ırddisp.pl?gene=SCD
ırddisp.pl?gene=AFG3L2
ırddisp.pl?gene=TYRP1
ırddisp.pl?gene=SIRT5
ırddisp.pl?gene=NFKBIB
ırddisp.pl?gene=NME2
ırddisp.pl?gene=MIR346
ırddisp.pl?gene=PRMT1
ırddisp.pl?gene=MAX
ırddisp.pl?gene=NSMF
ırddisp.pl?gene=COLQ
ırddisp.pl?gene=AEBP1
ırddisp.pl?gene=IAPP
ırddisp.pl?gene=RRM2
ırddisp.pl?gene=CLDN5
ırddisp.pl?gene=BAIAP2L1
ırddisp.pl?gene=ECE1
ırddisp.pl?gene=ULK1
ırddisp.pl?gene=IGF2BP2
ırddisp.pl?gene=ACADL
ırddisp.pl?gene=ANLN
ırddisp.pl?gene=PENK
ırddisp.pl?gene=YWHAB
ırddisp.pl?gene=H2BC21
ırddisp.pl?gene=GOLPH3
ırddisp.pl?gene=YWHAZ
ırddisp.pl?gene=FGF16

ırddisp.pl?gene=ISG15
ırddisp.pl?gene=GUCY2C
ırddisp.pl?gene=DUSP19
ırddisp.pl?gene=AHSG
ırddisp.pl?gene=PABPN1
ırddisp.pl?gene=ZNF23
ırddisp.pl?gene=HOTTIP
ırddisp.pl?gene=ABHD11
ırddisp.pl?gene=PODXL
ırddisp.pl?gene=SULT1A1
ırddisp.pl?gene=LEFTY2
ırddisp.pl?gene=MRPL12
ırddisp.pl?gene=SOCS2
ırddisp.pl?gene=SERPING1
ırddisp.pl?gene=PNKD
ırddisp.pl?gene=GTF2H4
ırddisp.pl?gene=DDIT4
ırddisp.pl?gene=SRSF1
ırddisp.pl?gene=TRPV1
ırddisp.pl?gene=PRKACA
ırddisp.pl?gene=CHRD
ırddisp.pl?gene=PROK1
ırddisp.pl?gene=JAG2
ırddisp.pl?gene=NCOA1
ırddisp.pl?gene=HBE1
ırddisp.pl?gene=HTR3A
ırddisp.pl?gene=TMEM150B
ırddisp.pl?gene=GAR1
ırddisp.pl?gene=TIMP4
ırddisp.pl?gene=SACM1L
ırddisp.pl?gene=ANG
ırddisp.pl?gene=MIR212
ırddisp.pl?gene=CYP3A5
ırddisp.pl?gene=CLDN1
ırddisp.pl?gene=ADIPOR2
ırddisp.pl?gene=APOD
ırddisp.pl?gene=PIP
ırddisp.pl?gene=LAMB1
ırddisp.pl?gene=CYP51A1
ırddisp.pl?gene=PCBD1
ırddisp.pl?gene=NR5A2
ırddisp.pl?gene=DMP1
ırddisp.pl?gene=MAGEA3
ırddisp.pl?gene=IL4R
ırddisp.pl?gene=NOP2
ırddisp.pl?gene=S100A10
ırddisp.pl?gene=UBC
ırddisp.pl?gene=PES1
ırddisp.pl?gene=SLC25A1
ırddisp.pl?gene=CXCR3
ırddisp.pl?gene=ASIC1
ırddisp.pl?gene=RXRA
ırddisp.pl?gene=ACSL3
ırddisp.pl?gene=FZD1

ırddisp.pl?gene=EPHA1
ırddisp.pl?gene=SOD3
ırddisp.pl?gene=MXD1
ırddisp.pl?gene=BNIP3
ırddisp.pl?gene=GRM1
ırddisp.pl?gene=SNAP25
ırddisp.pl?gene=CYSLTR1
ırddisp.pl?gene=CUL3
ırddisp.pl?gene=NEIL2
ırddisp.pl?gene=MYF6
ırddisp.pl?gene=TAC1
ırddisp.pl?gene=PPARD
ırddisp.pl?gene=LBP
ırddisp.pl?gene=PRDX1
ırddisp.pl?gene=TTPA
ırddisp.pl?gene=PDE4A
ırddisp.pl?gene=EGR1
ırddisp.pl?gene=NDUFS3
ırddisp.pl?gene=LY96
ırddisp.pl?gene=PIGR
ırddisp.pl?gene=NR0B2
ırddisp.pl?gene=SLC1A2
ırddisp.pl?gene=RPL7A
ırddisp.pl?gene=DNM1
ırddisp.pl?gene=CLN3
ırddisp.pl?gene=H3C14
ırddisp.pl?gene=SFN
ırddisp.pl?gene=ACKR1
ırddisp.pl?gene=F13A1
ırddisp.pl?gene=HLA-E
ırddisp.pl?gene=NPC1L1
ırddisp.pl?gene=SOX18
ırddisp.pl?gene=TBCE
ırddisp.pl?gene=PTPRN
ırddisp.pl?gene=PATZ1
ırddisp.pl?gene=EPHB2
ırddisp.pl?gene=GNB3
ırddisp.pl?gene=MRPS16
ırddisp.pl?gene=NARS2
ırddisp.pl?gene=NMB
ırddisp.pl?gene=GLO1
ırddisp.pl?gene=TRO
ırddisp.pl?gene=DEFB4A
ırddisp.pl?gene=RPA2
ırddisp.pl?gene=FCGRT
ırddisp.pl?gene=CDC20
ırddisp.pl?gene=FBLN2
ırddisp.pl?gene=PLA2G6
ırddisp.pl?gene=CPB2
ırddisp.pl?gene=PRDX5
ırddisp.pl?gene=MYF5
ırddisp.pl?gene=PIBF1
ırddisp.pl?gene=LOXL2
ırddisp.pl?gene=MIR125B2

ırddisp.pl?gene=SETDB1
ırddisp.pl?gene=BCHE
ırddisp.pl?gene=TPH2
ırddisp.pl?gene=TNS1
ırddisp.pl?gene=PLA2G4A
ırddisp.pl?gene=IGFBP7
ırddisp.pl?gene=CDK12
ırddisp.pl?gene=PRPF8
ırddisp.pl?gene=TCL1A
ırddisp.pl?gene=RHAG
ırddisp.pl?gene=AKR1C3
ırddisp.pl?gene=RAB3GAP2
ırddisp.pl?gene=GET3
ırddisp.pl?gene=HSD17B7
ırddisp.pl?gene=HLX
ırddisp.pl?gene=DEPTOR
ırddisp.pl?gene=F12
ırddisp.pl?gene=ID2
ırddisp.pl?gene=GALNT17
ırddisp.pl?gene=CDC73
ırddisp.pl?gene=ATF2
ırddisp.pl?gene=PARS2
ırddisp.pl?gene=CD70
ırddisp.pl?gene=TSPO
ırddisp.pl?gene=GJA8
ırddisp.pl?gene=ATP6AP2
ırddisp.pl?gene=SYK
ırddisp.pl?gene=GTF2H1
ırddisp.pl?gene=WNT8B
ırddisp.pl?gene=SLC4A4
ırddisp.pl?gene=TLE1
ırddisp.pl?gene=UBA1
ırddisp.pl?gene=WDR45B
ırddisp.pl?gene=TRIM50
ırddisp.pl?gene=MT2A
ırddisp.pl?gene=PSMB4
ırddisp.pl?gene=AP2B1
ırddisp.pl?gene=SULT1E1
ırddisp.pl?gene=ANTXR2
ırddisp.pl?gene=LAMC2
ırddisp.pl?gene=TUBA1B
ırddisp.pl?gene=OSM
ırddisp.pl?gene=CARTPT
ırddisp.pl?gene=CLSPN
ırddisp.pl?gene=YME1L1
ırddisp.pl?gene=PROK2
ırddisp.pl?gene=KAT6A
ırddisp.pl?gene=TSHB
ırddisp.pl?gene=CHRNA2
ırddisp.pl?gene=CHRNB2
ırddisp.pl?gene=PYCARD
ırddisp.pl?gene=CISH
ırddisp.pl?gene=KPNB1
ırddisp.pl?gene=GABRG3

ırddisp.pl?gene=HDAC4
ırddisp.pl?gene=NRIP1
ırddisp.pl?gene=ACVR2B
ırddisp.pl?gene=WASL
ırddisp.pl?gene=PEG10
ırddisp.pl?gene=WNT1
ırddisp.pl?gene=SLC7A7
ırddisp.pl?gene=PRKCE
ırddisp.pl?gene=BCL11A
ırddisp.pl?gene=MAGED1
ırddisp.pl?gene=THBS2
ırddisp.pl?gene=KIF1B
ırddisp.pl?gene=RLN2
ırddisp.pl?gene=TFPI
ırddisp.pl?gene=UBE2D2
ırddisp.pl?gene=PRKCSH
ırddisp.pl?gene=SSR1
ırddisp.pl?gene=AOC1
ırddisp.pl?gene=CRABP1
ırddisp.pl?gene=MAP4
ırddisp.pl?gene=EXOSC9
ırddisp.pl?gene=ASXL3
ırddisp.pl?gene=CSN2
ırddisp.pl?gene=RPS27
ırddisp.pl?gene=FTH1
ırddisp.pl?gene=MAVS
ırddisp.pl?gene=DSE
ırddisp.pl?gene=DNTT
ırddisp.pl?gene=LIMS2
ırddisp.pl?gene=PTS
ırddisp.pl?gene=GPBAR1
ırddisp.pl?gene=ADAM10
ırddisp.pl?gene=CCL19
ırddisp.pl?gene=SLC19A1
ırddisp.pl?gene=CD83
ırddisp.pl?gene=ACLY
ırddisp.pl?gene=SNX19
ırddisp.pl?gene=ASH2L
ırddisp.pl?gene=DPY30
ırddisp.pl?gene=HEYL
ırddisp.pl?gene=OBSCN
ırddisp.pl?gene=CTNNAL1
ırddisp.pl?gene=NISCH
ırddisp.pl?gene=CARS1
ırddisp.pl?gene=ODR4
ırddisp.pl?gene=CD200
ırddisp.pl?gene=IGFBP6
ırddisp.pl?gene=RPS6KA6
ırddisp.pl?gene=MIR27B
ırddisp.pl?gene=CNBP
ırddisp.pl?gene=MT-ND4
ırddisp.pl?gene=REV3L
ırddisp.pl?gene=NEUROD1
ırddisp.pl?gene=GPX1

ırddisp.pl?gene=CYP2B6
ırddisp.pl?gene=ACE2
ırddisp.pl?gene=UCP2
ırddisp.pl?gene=GAS2
ırddisp.pl?gene=SEC63
ırddisp.pl?gene=APELA
ırddisp.pl?gene=ID3
ırddisp.pl?gene=PCDHGA8
ırddisp.pl?gene=SAA4
ırddisp.pl?gene=UTS2
ırddisp.pl?gene=MIR1271
ırddisp.pl?gene=WNK4
ırddisp.pl?gene=PDS5B
ırddisp.pl?gene=OGT
ırddisp.pl?gene=AQP5
ırddisp.pl?gene=PSMA1
ırddisp.pl?gene=VARS2
ırddisp.pl?gene=AGPS
ırddisp.pl?gene=CDCA7
ırddisp.pl?gene=SLC5A1
ırddisp.pl?gene=FOXA2
ırddisp.pl?gene=FARS2
ırddisp.pl?gene=CLPX
ırddisp.pl?gene=CLRN1
ırddisp.pl?gene=SRD5A2
ırddisp.pl?gene=HMBS
ırddisp.pl?gene=MSTN
ırddisp.pl?gene=SEM1
ırddisp.pl?gene=FES
ırddisp.pl?gene=CDKAL1
ırddisp.pl?gene=MFAP2
ırddisp.pl?gene=DDRGK1
ırddisp.pl?gene=AHCY
ırddisp.pl?gene=FUCA2
ırddisp.pl?gene=TXNL4A
ırddisp.pl?gene=PAX4
ırddisp.pl?gene=L2HGDH
ırddisp.pl?gene=NORAD
ırddisp.pl?gene=UIMC1
ırddisp.pl?gene=CEP89
ırddisp.pl?gene=CCL18
ırddisp.pl?gene=OPRD1
ırddisp.pl?gene=MT-CO2
ırddisp.pl?gene=FGB
ırddisp.pl?gene=SLCO1B3
ırddisp.pl?gene=CKS1B
ırddisp.pl?gene=FCER2
ırddisp.pl?gene=MMP11
ırddisp.pl?gene=MSR1
ırddisp.pl?gene=HTRA1
ırddisp.pl?gene=ZAP70
ırddisp.pl?gene=PROX1
ırddisp.pl?gene=SOST
ırddisp.pl?gene=ONECUT2

ırddisp.pl?gene=RIT1
ırddisp.pl?gene=FBN3
ırddisp.pl?gene=CGB7
ırddisp.pl?gene=AMD1
ırddisp.pl?gene=USP14
ırddisp.pl?gene=DCT
ırddisp.pl?gene=SNX10
ırddisp.pl?gene=CALD1
ırddisp.pl?gene=GPAM
ırddisp.pl?gene=TFDP3
ırddisp.pl?gene=ELK1
ırddisp.pl?gene=UFL1
ırddisp.pl?gene=MDH2
ırddisp.pl?gene=NTF4
ırddisp.pl?gene=HELQ
ırddisp.pl?gene=ATF3
ırddisp.pl?gene=TCF20
ırddisp.pl?gene=LATS1
ırddisp.pl?gene=CRHR1
ırddisp.pl?gene=GABRB3
ırddisp.pl?gene=MEPE
ırddisp.pl?gene=RBL1
ırddisp.pl?gene=ENPP3
ırddisp.pl?gene=CD99
ırddisp.pl?gene=PNPLA4
ırddisp.pl?gene=RAD9A
ırddisp.pl?gene=WDR62
ırddisp.pl?gene=PABPC1
ırddisp.pl?gene=RNF135
ırddisp.pl?gene=CD2
ırddisp.pl?gene=RPS20P23
ırddisp.pl?gene=ANXA4
ırddisp.pl?gene=MAGEA4
ırddisp.pl?gene=MIR136
ırddisp.pl?gene=SERPINB3
ırddisp.pl?gene=TBX21
ırddisp.pl?gene=APOC1
ırddisp.pl?gene=NR1H3
ırddisp.pl?gene=PLA2G1B
ırddisp.pl?gene=TNKS
ırddisp.pl?gene=UBR5
ırddisp.pl?gene=IGF2-AS
ırddisp.pl?gene=WNT10B
ırddisp.pl?gene=PTGDS
ırddisp.pl?gene=SNAP29
ırddisp.pl?gene=ARF6
ırddisp.pl?gene=ZNHIT3
ırddisp.pl?gene=PNPLA2
ırddisp.pl?gene=SLC4A2
ırddisp.pl?gene=LYN
ırddisp.pl?gene=IKBKG
ırddisp.pl?gene=MAPKAPK2
ırddisp.pl?gene=HEY1
ırddisp.pl?gene=ITGA5

ırddisp.pl?gene=HOXD10
ırddisp.pl?gene=PDE3B
ırddisp.pl?gene=CXCL9
ırddisp.pl?gene=CALB1
ırddisp.pl?gene=POU1F1
ırddisp.pl?gene=CXCR1
ırddisp.pl?gene=LAMA5
ırddisp.pl?gene=CX3CL1
ırddisp.pl?gene=MIR101-1
ırddisp.pl?gene=CHD4
ırddisp.pl?gene=RNASEL
ırddisp.pl?gene=POLH
ırddisp.pl?gene=NUP98
ırddisp.pl?gene=FUT2
ırddisp.pl?gene=BLVRB
ırddisp.pl?gene=NEFL
ırddisp.pl?gene=LPP
ırddisp.pl?gene=GLMN
ırddisp.pl?gene=CDH17
ırddisp.pl?gene=GIP
ırddisp.pl?gene=CLTC
ırddisp.pl?gene=PAK1
ırddisp.pl?gene=ATP2A1
ırddisp.pl?gene=STIL
ırddisp.pl?gene=TRPC4
ırddisp.pl?gene=CTPS1
ırddisp.pl?gene=IDE
ırddisp.pl?gene=SOX6
ırddisp.pl?gene=SOX5
ırddisp.pl?gene=POU2AF1
ırddisp.pl?gene=SLC22A4
ırddisp.pl?gene=CXCL2
ırddisp.pl?gene=ACAA1
ırddisp.pl?gene=MYOZ1
ırddisp.pl?gene=NOD1
ırddisp.pl?gene=NEK2
ırddisp.pl?gene=PCSK2
ırddisp.pl?gene=ARL6
ırddisp.pl?gene=MCF2
ırddisp.pl?gene=CASP5
ırddisp.pl?gene=DBI
ırddisp.pl?gene=PSMC4
ırddisp.pl?gene=GPX4
ırddisp.pl?gene=STUB1
ırddisp.pl?gene=P4HB
ırddisp.pl?gene=TRPC1
ırddisp.pl?gene=H4-16
ırddisp.pl?gene=UCN
ırddisp.pl?gene=HDAC3
ırddisp.pl?gene=TRAF2
ırddisp.pl?gene=LIN9
ırddisp.pl?gene=MAGEF1
ırddisp.pl?gene=MIR133A1
ırddisp.pl?gene=PSAT1

ırddisp.pl?gene=GJC1
ırddisp.pl?gene=SLC13A5
ırddisp.pl?gene=AIM2
ırddisp.pl?gene=NAP1L4
ırddisp.pl?gene=KRT4
ırddisp.pl?gene=NR2F2
ırddisp.pl?gene=BBC3
ırddisp.pl?gene=CES1
ırddisp.pl?gene=FAM3C
ırddisp.pl?gene=TSLP
ırddisp.pl?gene=ROR2
ırddisp.pl?gene=PLCB4
ırddisp.pl?gene=ATF6
ırddisp.pl?gene=AFF4
ırddisp.pl?gene=PRB1
ırddisp.pl?gene=TPX2
ırddisp.pl?gene=OLR1
ırddisp.pl?gene=GPNMB
ırddisp.pl?gene=CUX1
ırddisp.pl?gene=DLX4
ırddisp.pl?gene=SLC22A2
ırddisp.pl?gene=SMAD5
ırddisp.pl?gene=FOXP1
ırddisp.pl?gene=PGRMC2
ırddisp.pl?gene=DGAT1
ırddisp.pl?gene=SOAT1
ırddisp.pl?gene=FADS2
ırddisp.pl?gene=ATAD2
ırddisp.pl?gene=ITIH2
ırddisp.pl?gene=CUL7
ırddisp.pl?gene=ECI2
ırddisp.pl?gene=MIR133B
ırddisp.pl?gene=RAD18
ırddisp.pl?gene=TRIM21
ırddisp.pl?gene=SCG5
ırddisp.pl?gene=TGFBI
ırddisp.pl?gene=ALDH3A1
ırddisp.pl?gene=KIF5A
ırddisp.pl?gene=DDX58
ırddisp.pl?gene=OPHN1
ırddisp.pl?gene=GJA3
ırddisp.pl?gene=GOLGA5
ırddisp.pl?gene=TFR2
ırddisp.pl?gene=HOXA4
ırddisp.pl?gene=RPL3
ırddisp.pl?gene=HDAC2
ırddisp.pl?gene=PTGER3
ırddisp.pl?gene=PTBP1
ırddisp.pl?gene=PRSS23
ırddisp.pl?gene=CYP2C8
ırddisp.pl?gene=CRELD1
ırddisp.pl?gene=WDR5
ırddisp.pl?gene=CKMT2
ırddisp.pl?gene=AGK

ırddisp.pl?gene=VAPB
ırddisp.pl?gene=PTGIS
ırddisp.pl?gene=VASH1
ırddisp.pl?gene=TYK2
ırddisp.pl?gene=PDP1
ırddisp.pl?gene=GDF2
ırddisp.pl?gene=WASHC5
ırddisp.pl?gene=ROS1
ırddisp.pl?gene=ADCY7
ırddisp.pl?gene=BAP1
ırddisp.pl?gene=RREB1
ırddisp.pl?gene=MIR449B
ırddisp.pl?gene=TNFRSF4
ırddisp.pl?gene=HMMR
ırddisp.pl?gene=PRKAR1B
ırddisp.pl?gene=LDHA
ırddisp.pl?gene=C19orf33
ırddisp.pl?gene=SGCG
ırddisp.pl?gene=NCS1
ırddisp.pl?gene=SLC25A6
ırddisp.pl?gene=CELF2
ırddisp.pl?gene=FPGS
ırddisp.pl?gene=HSF1
ırddisp.pl?gene=NRGN
ırddisp.pl?gene=ASH1L
ırddisp.pl?gene=GCLM
ırddisp.pl?gene=ADCY2
ırddisp.pl?gene=SMURF1
ırddisp.pl?gene=DNAJB1
ırddisp.pl?gene=CRABP2
ırddisp.pl?gene=CADM1
ırddisp.pl?gene=RBFOX1
ırddisp.pl?gene=F11
ırddisp.pl?gene=OPTN
ırddisp.pl?gene=STIP1
ırddisp.pl?gene=NOX4
ırddisp.pl?gene=TAB2
ırddisp.pl?gene=AHSP
ırddisp.pl?gene=MIR19A
ırddisp.pl?gene=ITIH4
ırddisp.pl?gene=FBXO32
ırddisp.pl?gene=PIK3C3
ırddisp.pl?gene=ATG14
ırddisp.pl?gene=ITGA2B
ırddisp.pl?gene=MRPL23
ırddisp.pl?gene=CHST11
ırddisp.pl?gene=CENPA
ırddisp.pl?gene=IDH3G
ırddisp.pl?gene=WAPL
ırddisp.pl?gene=BMP10
ırddisp.pl?gene=CELF1
ırddisp.pl?gene=TXNIP
ırddisp.pl?gene=HAVCR1
ırddisp.pl?gene=TFE3

ırddisp.pl?gene=ENPEP
ırddisp.pl?gene=STARD3
ırddisp.pl?gene=SKIL
ırddisp.pl?gene=PTPN6
ırddisp.pl?gene=CYB561
ırddisp.pl?gene=EIPR1
ırddisp.pl?gene=RFWD3
ırddisp.pl?gene=LAMC1
ırddisp.pl?gene=PRKCZ
ırddisp.pl?gene=NDUFS4
ırddisp.pl?gene=ADORA2A
ırddisp.pl?gene=HES1
ırddisp.pl?gene=ADH4
ırddisp.pl?gene=HACD1
ırddisp.pl?gene=IL27
ırddisp.pl?gene=ISL1
ırddisp.pl?gene=SCAP
ırddisp.pl?gene=TSSC4
ırddisp.pl?gene=DUS3L
ırddisp.pl?gene=MDM4
ırddisp.pl?gene=PVALB
ırddisp.pl?gene=HSD17B12
ırddisp.pl?gene=IRF7
ırddisp.pl?gene=RARRES2
ırddisp.pl?gene=PSMB2
ırddisp.pl?gene=HUS1
ırddisp.pl?gene=LRRK2
ırddisp.pl?gene=TACSTD2
ırddisp.pl?gene=ALKBH5
ırddisp.pl?gene=SLC1A5
ırddisp.pl?gene=HPGDS
ırddisp.pl?gene=RPSA
ırddisp.pl?gene=ST3GAL4
ırddisp.pl?gene=UBE2L3
ırddisp.pl?gene=COASY
ırddisp.pl?gene=GRHL2
ırddisp.pl?gene=PCM1
ırddisp.pl?gene=LCN1
ırddisp.pl?gene=USP2
ırddisp.pl?gene=ZNF496
ırddisp.pl?gene=UCP3
ırddisp.pl?gene=SLC35A1
ırddisp.pl?gene=NEDD4L
ırddisp.pl?gene=TAF4B
ırddisp.pl?gene=ETS2
ırddisp.pl?gene=REL
ırddisp.pl?gene=TXN2
ırddisp.pl?gene=CDK9
ırddisp.pl?gene=BHLHE40
ırddisp.pl?gene=SMC2
ırddisp.pl?gene=OPA1
ırddisp.pl?gene=HSP90AB1
ırddisp.pl?gene=YBX2
ırddisp.pl?gene=FXYD1

ırddisp.pl?gene=AQR
ırddisp.pl?gene=LMLN
ırddisp.pl?gene=FZR1
ırddisp.pl?gene=KLHDC2
ırddisp.pl?gene=GJB3
ırddisp.pl?gene=SH3BP4
ırddisp.pl?gene=GJB4
ırddisp.pl?gene=GSS
ırddisp.pl?gene=HOXD12
ırddisp.pl?gene=PLCZ1
ırddisp.pl?gene=ESM1
ırddisp.pl?gene=MSN
ırddisp.pl?gene=SPRY4
ırddisp.pl?gene=LHX3
ırddisp.pl?gene=MKKS
ırddisp.pl?gene=TRIM37
ırddisp.pl?gene=GSTA3
ırddisp.pl?gene=PRPF31
ırddisp.pl?gene=PAK3
ırddisp.pl?gene=CXCL16
ırddisp.pl?gene=ITLN1
ırddisp.pl?gene=LDHB
ırddisp.pl?gene=XPO5
ırddisp.pl?gene=HJURP
ırddisp.pl?gene=ZMYND10
ırddisp.pl?gene=EGLN1
ırddisp.pl?gene=SIL1
ırddisp.pl?gene=AMELX
ırddisp.pl?gene=FRZB
ırddisp.pl?gene=RAB1A
ırddisp.pl?gene=SEC24B
ırddisp.pl?gene=ACBD5
ırddisp.pl?gene=HIKESHI
ırddisp.pl?gene=FJX1
ırddisp.pl?gene=MACO1
ırddisp.pl?gene=GALNT3
ırddisp.pl?gene=BUB3
ırddisp.pl?gene=ACAD9
ırddisp.pl?gene=ARHGDIA
ırddisp.pl?gene=DEFB103B
ırddisp.pl?gene=MBD2
ırddisp.pl?gene=CUL4B
ırddisp.pl?gene=NUP210
ırddisp.pl?gene=MARCKS
ırddisp.pl?gene=MAP2K6
ırddisp.pl?gene=CAPN2
ırddisp.pl?gene=TAP1
ırddisp.pl?gene=SETD1B
ırddisp.pl?gene=WDR70
ırddisp.pl?gene=DAPK1
ırddisp.pl?gene=EIF4A3
ırddisp.pl?gene=SLC1A1
ırddisp.pl?gene=KRIT1
ırddisp.pl?gene=TUBA1A

ırddisp.pl?gene=TMEM87A
ırddisp.pl?gene=OPRL1
ırddisp.pl?gene=TP53COR1
ırddisp.pl?gene=NEK3
ırddisp.pl?gene=FBXO5
ırddisp.pl?gene=DVL3
ırddisp.pl?gene=AP1M1
ırddisp.pl?gene=VSIR
ırddisp.pl?gene=ATG7
ırddisp.pl?gene=WNT6
ırddisp.pl?gene=NEUROG1
ırddisp.pl?gene=OAS1
ırddisp.pl?gene=IQGAP1
ırddisp.pl?gene=SLC30A8
ırddisp.pl?gene=RPS18
ırddisp.pl?gene=IFIH1
ırddisp.pl?gene=PDIA3
ırddisp.pl?gene=TNFSF13
ırddisp.pl?gene=PARD3B
ırddisp.pl?gene=SELENOS
ırddisp.pl?gene=PTGFR
ırddisp.pl?gene=OLIG2
ırddisp.pl?gene=COL4A2
ırddisp.pl?gene=DAXX
ırddisp.pl?gene=PAX9
ırddisp.pl?gene=ZNF214
ırddisp.pl?gene=PDHX
ırddisp.pl?gene=NPY2R
ırddisp.pl?gene=BBS7
ırddisp.pl?gene=SLIT2
ırddisp.pl?gene=UBE2I
ırddisp.pl?gene=TACC1
ırddisp.pl?gene=MT-ND5
ırddisp.pl?gene=HTT
ırddisp.pl?gene=MIR411
ırddisp.pl?gene=DUSP6
ırddisp.pl?gene=HOXB1
ırddisp.pl?gene=CANX
ırddisp.pl?gene=HRH2
ırddisp.pl?gene=SH2B3
ırddisp.pl?gene=NDUFS8
ırddisp.pl?gene=VLDLR
ırddisp.pl?gene=PTOV1
ırddisp.pl?gene=SLC12A2
ırddisp.pl?gene=ACVRL1
ırddisp.pl?gene=CTSK
ırddisp.pl?gene=NBAS
ırddisp.pl?gene=IL13RA2
ırddisp.pl?gene=MAOB
ırddisp.pl?gene=SULF2
ırddisp.pl?gene=PUM1
ırddisp.pl?gene=CLIP1
ırddisp.pl?gene=CANT1
ırddisp.pl?gene=NT5E

ırddisp.pl?gene=SLC12A4
ırddisp.pl?gene=CCL20
ırddisp.pl?gene=PDXDC1
ırddisp.pl?gene=HMCN1
ırddisp.pl?gene=FOSL2
ırddisp.pl?gene=ABCC4
ırddisp.pl?gene=ZNF695
ırddisp.pl?gene=CERK
ırddisp.pl?gene=CASK
ırddisp.pl?gene=AKR1C2
ırddisp.pl?gene=MAP3K14
ırddisp.pl?gene=PSMC6
ırddisp.pl?gene=DGKB
ırddisp.pl?gene=MAN1B1
ırddisp.pl?gene=SRF
ırddisp.pl?gene=FYN
ırddisp.pl?gene=DRD1
ırddisp.pl?gene=ADAMTS16
ırddisp.pl?gene=KCNK9
ırddisp.pl?gene=UBE2D3
ırddisp.pl?gene=CCDC120
ırddisp.pl?gene=EPHB3
ırddisp.pl?gene=GNAI1
ırddisp.pl?gene=SLCO1B1
ırddisp.pl?gene=LAT
ırddisp.pl?gene=KCNN3
ırddisp.pl?gene=ACSL6
ırddisp.pl?gene=JUNB
ırddisp.pl?gene=CCR9
ırddisp.pl?gene=GATM
ırddisp.pl?gene=ROCK2
ırddisp.pl?gene=MID2
ırddisp.pl?gene=DGCR8
ırddisp.pl?gene=SLIT3
ırddisp.pl?gene=IL18BP
ırddisp.pl?gene=DRD3
ırddisp.pl?gene=EPHA3
ırddisp.pl?gene=DEPDC5
ırddisp.pl?gene=TERF2IP
ırddisp.pl?gene=PLCB1
ırddisp.pl?gene=TXNRD1
ırddisp.pl?gene=ATG9A
ırddisp.pl?gene=VPS35
ırddisp.pl?gene=SIM2
ırddisp.pl?gene=MUC3A
ırddisp.pl?gene=TACC3
ırddisp.pl?gene=SAR1B
ırddisp.pl?gene=FALEC
ırddisp.pl?gene=TRIM28
ırddisp.pl?gene=TMOD1
ırddisp.pl?gene=MIR1908
ırddisp.pl?gene=TTC8
ırddisp.pl?gene=EPS15
ırddisp.pl?gene=TOP3B

ırddisp.pl?gene=COL16A1
ırddisp.pl?gene=NAP1L5
ırddisp.pl?gene=TMEM258
ırddisp.pl?gene=PER2
ırddisp.pl?gene=NCOA6
ırddisp.pl?gene=TNC
ırddisp.pl?gene=KLK15
ırddisp.pl?gene=WIF1
ırddisp.pl?gene=PPP1R3A
ırddisp.pl?gene=BACH1
ırddisp.pl?gene=SLC25A3
ırddisp.pl?gene=HCK
ırddisp.pl?gene=MIR216A
ırddisp.pl?gene=SLC3A2
ırddisp.pl?gene=CX3CR1
ırddisp.pl?gene=PPP2R3C
ırddisp.pl?gene=ITFG1
ırddisp.pl?gene=PARK7
ırddisp.pl?gene=CEP70
ırddisp.pl?gene=MGAM
ırddisp.pl?gene=EMP1
ırddisp.pl?gene=FMO3
ırddisp.pl?gene=CEND1
ırddisp.pl?gene=ROBO1
ırddisp.pl?gene=TMEM199
ırddisp.pl?gene=MC3R
ırddisp.pl?gene=HAVCR2
ırddisp.pl?gene=AGPAT1
ırddisp.pl?gene=GSTZ1
ırddisp.pl?gene=SCD5
ırddisp.pl?gene=VKORC1L1
ırddisp.pl?gene=CERS4
ırddisp.pl?gene=GPM6B
ırddisp.pl?gene=LPCAT3
ırddisp.pl?gene=BBX
ırddisp.pl?gene=PEBP1
ırddisp.pl?gene=SRSF2
ırddisp.pl?gene=FOXO4
ırddisp.pl?gene=ZNF142
ırddisp.pl?gene=GPI
ırddisp.pl?gene=GNB1
ırddisp.pl?gene=HRH1
ırddisp.pl?gene=IFI16
ırddisp.pl?gene=FABP12
ırddisp.pl?gene=HAS1
ırddisp.pl?gene=NUP93
ırddisp.pl?gene=PRKCI
ırddisp.pl?gene=PRDX3
ırddisp.pl?gene=BAMBI
ırddisp.pl?gene=PHGDH
ırddisp.pl?gene=ELP1
ırddisp.pl?gene=SLC6A9
ırddisp.pl?gene=YWHAE
ırddisp.pl?gene=DKK3

ırddisp.pl?gene=KRT6A
ırddisp.pl?gene=NCL
ırddisp.pl?gene=SUPT5H
ırddisp.pl?gene=SMOX
ırddisp.pl?gene=KAT2B
ırddisp.pl?gene=THY1
ırddisp.pl?gene=MAP4K4
ırddisp.pl?gene=IL10RA
ırddisp.pl?gene=VAMP4
ırddisp.pl?gene=ICAM5
ırddisp.pl?gene=IFNGR2
ırddisp.pl?gene=ID1
ırddisp.pl?gene=TRPM4
ırddisp.pl?gene=NDUFA13
ırddisp.pl?gene=MCM5
ırddisp.pl?gene=AOPEP
ırddisp.pl?gene=EHF
ırddisp.pl?gene=CAP1
ırddisp.pl?gene=MYO6
ırddisp.pl?gene=CSNK2A1
ırddisp.pl?gene=GNLY
ırddisp.pl?gene=COL14A1
ırddisp.pl?gene=COIL
ırddisp.pl?gene=CEBPA
ırddisp.pl?gene=FUT1
ırddisp.pl?gene=SGCB
ırddisp.pl?gene=MSMB
ırddisp.pl?gene=NRF1
ırddisp.pl?gene=PKN1
ırddisp.pl?gene=TRA
ırddisp.pl?gene=DDOST
ırddisp.pl?gene=NLRP11
ırddisp.pl?gene=LPO
ırddisp.pl?gene=HPX
ırddisp.pl?gene=OPRK1
ırddisp.pl?gene=VAPA
ırddisp.pl?gene=PPA2
ırddisp.pl?gene=EEF1B2
ırddisp.pl?gene=PDLIM5
ırddisp.pl?gene=TYRO3
ırddisp.pl?gene=C1QBP
ırddisp.pl?gene=THRA
ırddisp.pl?gene=KLKB1
ırddisp.pl?gene=PSMD7
ırddisp.pl?gene=CDC27
ırddisp.pl?gene=H4C1
ırddisp.pl?gene=TNFAIP6
ırddisp.pl?gene=INTS11
ırddisp.pl?gene=CSK
ırddisp.pl?gene=CCL25
ırddisp.pl?gene=PSMD1
ırddisp.pl?gene=PTPN2
ırddisp.pl?gene=NPR3
ırddisp.pl?gene=RXRB

ırddisp.pl?gene=BCL2L2
ırddisp.pl?gene=ADCY5
ırddisp.pl?gene=MMP26
ırddisp.pl?gene=BCAT1
ırddisp.pl?gene=DTL
ırddisp.pl?gene=CYP2J2
ırddisp.pl?gene=RNF8
ırddisp.pl?gene=SIK2
ırddisp.pl?gene=USP44
ırddisp.pl?gene=CCL22
ırddisp.pl?gene=TRAP1
ırddisp.pl?gene=HSP90B1
ırddisp.pl?gene=FTL
ırddisp.pl?gene=PRRT2
ırddisp.pl?gene=FPR1
ırddisp.pl?gene=MAPK7
ırddisp.pl?gene=TRIM32
ırddisp.pl?gene=SUMO1
ırddisp.pl?gene=MIR217
ırddisp.pl?gene=CBX3
ırddisp.pl?gene=SERPINB7
ırddisp.pl?gene=MALT1
ırddisp.pl?gene=NCR3
ırddisp.pl?gene=CCDC114
ırddisp.pl?gene=UBE2A
ırddisp.pl?gene=KLF5
ırddisp.pl?gene=DVL1
ırddisp.pl?gene=PIN1
ırddisp.pl?gene=GPX5
ırddisp.pl?gene=KCNK2
ırddisp.pl?gene=TM7SF3
ırddisp.pl?gene=MICB
ırddisp.pl?gene=EME1
ırddisp.pl?gene=TFB1M
ırddisp.pl?gene=ACVR1C
ırddisp.pl?gene=NXF2
ırddisp.pl?gene=CTRL
ırddisp.pl?gene=IMPA1
ırddisp.pl?gene=IL17F
ırddisp.pl?gene=MAP2K3
ırddisp.pl?gene=ERLIN2
ırddisp.pl?gene=PRKCQ
ırddisp.pl?gene=PSIP1
ırddisp.pl?gene=PTPRU
ırddisp.pl?gene=ETV5
ırddisp.pl?gene=TBK1
ırddisp.pl?gene=CEACAM1
ırddisp.pl?gene=BMP3
ırddisp.pl?gene=MYH10
ırddisp.pl?gene=SLC25A19
ırddisp.pl?gene=FBP1
ırddisp.pl?gene=KIF14
ırddisp.pl?gene=PNPLA3
ırddisp.pl?gene=CXCL5

ırddisp.pl?gene=TRAF1
ırddisp.pl?gene=INHBC
ırddisp.pl?gene=EIF2AK2
ırddisp.pl?gene=BTRC
ırddisp.pl?gene=BCL2A1
ırddisp.pl?gene=OVGP1
ırddisp.pl?gene=CDKL5
ırddisp.pl?gene=MSBP1
ırddisp.pl?gene=MAP1LC3A
ırddisp.pl?gene=TUBB1
ırddisp.pl?gene=ARCN1
ırddisp.pl?gene=PSMD3
ırddisp.pl?gene=PSMD11
ırddisp.pl?gene=PSMC2
ırddisp.pl?gene=PLS3
ırddisp.pl?gene=ADIPQTL4
ırddisp.pl?gene=TREX1
ırddisp.pl?gene=PSMD10
ırddisp.pl?gene=CRAT
ırddisp.pl?gene=BRD7
ırddisp.pl?gene=DROSHA
ırddisp.pl?gene=FIBP
ırddisp.pl?gene=DMBT1
ırddisp.pl?gene=APOBEC3G
ırddisp.pl?gene=CBX1
ırddisp.pl?gene=TBL1XR1
ırddisp.pl?gene=EPM2A
ırddisp.pl?gene=OGDH
ırddisp.pl?gene=CYP2A6
ırddisp.pl?gene=TMEM107
ırddisp.pl?gene=TNXA
ırddisp.pl?gene=B3GLCT
ırddisp.pl?gene=XAF1
ırddisp.pl?gene=ECT2
ırddisp.pl?gene=ADAMTS9
ırddisp.pl?gene=PPIF
ırddisp.pl?gene=EIF5A
ırddisp.pl?gene=F11R
ırddisp.pl?gene=SKP1
ırddisp.pl?gene=SP3
ırddisp.pl?gene=C5AR1
ırddisp.pl?gene=UBTF
ırddisp.pl?gene=ARRB2
ırddisp.pl?gene=CHFR
ırddisp.pl?gene=ZFX
ırddisp.pl?gene=UGT2B7
ırddisp.pl?gene=AQP8
ırddisp.pl?gene=GART
ırddisp.pl?gene=PANX1
ırddisp.pl?gene=GZMA
ırddisp.pl?gene=MAP2
ırddisp.pl?gene=HK1
ırddisp.pl?gene=TMEM86A
ırddisp.pl?gene=TAPBP

ırddisp.pl?gene=DRD4
ırddisp.pl?gene=FDFT1
ırddisp.pl?gene=LRPAP1
ırddisp.pl?gene=PSMA3
ırddisp.pl?gene=RAPGEF3
ırddisp.pl?gene=XPO7
ırddisp.pl?gene=TTF2
ırddisp.pl?gene=SLC46A1
ırddisp.pl?gene=FGF14
ırddisp.pl?gene=KAT5
ırddisp.pl?gene=ANGPTL2
ırddisp.pl?gene=MIR328
ırddisp.pl?gene=RARG
ırddisp.pl?gene=WNT2
ırddisp.pl?gene=BST2
ırddisp.pl?gene=IGKC
ırddisp.pl?gene=C4BPA
ırddisp.pl?gene=MAGEA11
ırddisp.pl?gene=SLC34A2
ırddisp.pl?gene=CTH
ırddisp.pl?gene=MYOZ2
ırddisp.pl?gene=RAB18
ırddisp.pl?gene=HSPA6
ırddisp.pl?gene=MYOC
ırddisp.pl?gene=DOCK1
ırddisp.pl?gene=PDYN
ırddisp.pl?gene=KDM5B
ırddisp.pl?gene=USP15
ırddisp.pl?gene=VANGL2
ırddisp.pl?gene=VGF
ırddisp.pl?gene=EXT1
ırddisp.pl?gene=KIR2DL1
ırddisp.pl?gene=NAT1
ırddisp.pl?gene=DYNLL1
ırddisp.pl?gene=KLK2
ırddisp.pl?gene=COPB2
ırddisp.pl?gene=PSMA7
ırddisp.pl?gene=HSPE1
ırddisp.pl?gene=TUBB4A
ırddisp.pl?gene=CKAP5
ırddisp.pl?gene=TUBG1
ırddisp.pl?gene=CREM
ırddisp.pl?gene=GDF1
ırddisp.pl?gene=BHMT
ırddisp.pl?gene=CCM2
ırddisp.pl?gene=LOC108783649
ırddisp.pl?gene=TBX3
ırddisp.pl?gene=DEK
ırddisp.pl?gene=IFNAR2
ırddisp.pl?gene=PSMA2
ırddisp.pl?gene=PRRC2A
ırddisp.pl?gene=DCTN2
ırddisp.pl?gene=COL4A6
ırddisp.pl?gene=SERPINA12

ırddisp.pl?gene=ITGA1
ırddisp.pl?gene=CSPG4
ırddisp.pl?gene=NUAK2
ırddisp.pl?gene=GP2
ırddisp.pl?gene=PEPD
ırddisp.pl?gene=ITGB8
ırddisp.pl?gene=MCCC2
ırddisp.pl?gene=TRA2B
ırddisp.pl?gene=NCOR2
ırddisp.pl?gene=CACNA1G
ırddisp.pl?gene=SALL2
ırddisp.pl?gene=GNL3
ırddisp.pl?gene=RNASE2
ırddisp.pl?gene=IGFALS
ırddisp.pl?gene=CA4
ırddisp.pl?gene=GABBR2
ırddisp.pl?gene=SLC38A2
ırddisp.pl?gene=DIO1
ırddisp.pl?gene=HOXB4
ırddisp.pl?gene=CAPN1
ırddisp.pl?gene=STK4
ırddisp.pl?gene=SCAMP1
ırddisp.pl?gene=SLC1A3
ırddisp.pl?gene=B4GALT1
ırddisp.pl?gene=SMARCE1
ırddisp.pl?gene=INTS4
ırddisp.pl?gene=DOT1L
ırddisp.pl?gene=PSMD9
ırddisp.pl?gene=SET
ırddisp.pl?gene=MRPL45
ırddisp.pl?gene=CDK5RAP2
ırddisp.pl?gene=NUDT6
ırddisp.pl?gene=PARP2
ırddisp.pl?gene=IL3RA
ırddisp.pl?gene=INSRR
ırddisp.pl?gene=UBE2N
ırddisp.pl?gene=PSMB3
ırddisp.pl?gene=CAST
ırddisp.pl?gene=PSMB1
ırddisp.pl?gene=RTN1
ırddisp.pl?gene=DLX1
ırddisp.pl?gene=DERL1
ırddisp.pl?gene=SH3KBP1
ırddisp.pl?gene=TBX2
ırddisp.pl?gene=CD177
ırddisp.pl?gene=EEF1A1
ırddisp.pl?gene=FOSB
ırddisp.pl?gene=PRDX2
ırddisp.pl?gene=ITGB6
ırddisp.pl?gene=SLC12A7
ırddisp.pl?gene=CBR3
ırddisp.pl?gene=NCR2
ırddisp.pl?gene=HYAL1
ırddisp.pl?gene=DDAH2

ırddisp.pl?gene=NCR1
ırddisp.pl?gene=ZMYND11
ırddisp.pl?gene=HOXB5
ırddisp.pl?gene=NHS
ırddisp.pl?gene=CYLD
ırddisp.pl?gene=PCBP1
ırddisp.pl?gene=CUL5
ırddisp.pl?gene=KTN1
ırddisp.pl?gene=UQCRC2
ırddisp.pl?gene=SLC2A6
ırddisp.pl?gene=DRD5
ırddisp.pl?gene=RNF5
ırddisp.pl?gene=PAF1
ırddisp.pl?gene=ALOX15B
ırddisp.pl?gene=PRG2
ırddisp.pl?gene=OGN
ırddisp.pl?gene=COL24A1
ırddisp.pl?gene=GSTM2
ırddisp.pl?gene=KNL1
ırddisp.pl?gene=GOPC
ırddisp.pl?gene=FKBP5
ırddisp.pl?gene=SLC6A2
ırddisp.pl?gene=PINK1
ırddisp.pl?gene=ACTN3
ırddisp.pl?gene=AQP9
ırddisp.pl?gene=GDF6
ırddisp.pl?gene=PLEKHA1
ırddisp.pl?gene=CHRNA4
ırddisp.pl?gene=IL37
ırddisp.pl?gene=NOL11
ırddisp.pl?gene=HEXB
ırddisp.pl?gene=MAPK9
ırddisp.pl?gene=RPS15
ırddisp.pl?gene=CALCR
ırddisp.pl?gene=ALDOA
ırddisp.pl?gene=UGT2B4
ırddisp.pl?gene=RBM8A
ırddisp.pl?gene=RALGDS
ırddisp.pl?gene=NADSYN1
ırddisp.pl?gene=LMAN1
ırddisp.pl?gene=ALOX15
ırddisp.pl?gene=TOMM40
ırddisp.pl?gene=TOR1AIP1
ırddisp.pl?gene=CHRNA7
ırddisp.pl?gene=SLC12A5
ırddisp.pl?gene=APLNR
ırddisp.pl?gene=PFKFB3
ırddisp.pl?gene=SEC22B
ırddisp.pl?gene=TRIP10
ırddisp.pl?gene=CASP4
ırddisp.pl?gene=CLINT1
ırddisp.pl?gene=SSR4
ırddisp.pl?gene=ADRB1
ırddisp.pl?gene=CITED4

ırddisp.pl?gene=CCNT1
ırddisp.pl?gene=GDF11
ırddisp.pl?gene=MCHR1
ırddisp.pl?gene=ANKK1
ırddisp.pl?gene=HNRNPC
ırddisp.pl?gene=IRAK4
ırddisp.pl?gene=RPLP0
ırddisp.pl?gene=CYC1
ırddisp.pl?gene=HSPA1B
ırddisp.pl?gene=CRISP3
ırddisp.pl?gene=IL15RA
ırddisp.pl?gene=CWF19L2
ırddisp.pl?gene=MIR760
ırddisp.pl?gene=HIC1
ırddisp.pl?gene=PNLIPRP1
ırddisp.pl?gene=SP100
ırddisp.pl?gene=RPS13
ırddisp.pl?gene=LOC108863620
ırddisp.pl?gene=CLDN6
ırddisp.pl?gene=PARD3
ırddisp.pl?gene=TIMM50
ırddisp.pl?gene=USP1
ırddisp.pl?gene=RAB11A
ırddisp.pl?gene=TFDP1
ırddisp.pl?gene=OAS3
ırddisp.pl?gene=IRF9
ırddisp.pl?gene=CYP26A1
ırddisp.pl?gene=SCARA3
ırddisp.pl?gene=MIR208A
ırddisp.pl?gene=SNAP23
ırddisp.pl?gene=A2M
ırddisp.pl?gene=RSPO3
ırddisp.pl?gene=TPPP3
ırddisp.pl?gene=DHRS4
ırddisp.pl?gene=RRAD
ırddisp.pl?gene=TRB
ırddisp.pl?gene=TGM3
ırddisp.pl?gene=ICAM3
ırddisp.pl?gene=CNR2
ırddisp.pl?gene=INHBE
ırddisp.pl?gene=ALDH5A1
ırddisp.pl?gene=C7
ırddisp.pl?gene=PSMC1
ırddisp.pl?gene=MEOX1
ırddisp.pl?gene=TNIP1
ırddisp.pl?gene=CSNK2A2
ırddisp.pl?gene=FMO1
ırddisp.pl?gene=RXFP1
ırddisp.pl?gene=ENHO
ırddisp.pl?gene=STX6
ırddisp.pl?gene=UPK1B
ırddisp.pl?gene=EGFL7
ırddisp.pl?gene=LSP1
ırddisp.pl?gene=KMT5C

ırddisp.pl?gene=AOX1
ırddisp.pl?gene=NCK1
ırddisp.pl?gene=UBA52
ırddisp.pl?gene=COX6A1
ırddisp.pl?gene=CAMK2G
ırddisp.pl?gene=SLC20A1
ırddisp.pl?gene=CCL8
ırddisp.pl?gene=POLR2H
ırddisp.pl?gene=VTI1B
ırddisp.pl?gene=CDK5
ırddisp.pl?gene=LSM2
ırddisp.pl?gene=HMGCS2
ırddisp.pl?gene=UGT1A9
ırddisp.pl?gene=FAT1
ırddisp.pl?gene=GOSR2
ırddisp.pl?gene=PRKAR2A
ırddisp.pl?gene=CENPB
ırddisp.pl?gene=ATP2C1
ırddisp.pl?gene=IPO5
ırddisp.pl?gene=LRPPRC
ırddisp.pl?gene=PNPT1
ırddisp.pl?gene=MCM3
ırddisp.pl?gene=IFN1%40
ırddisp.pl?gene=ARID2
ırddisp.pl?gene=EIF4A2
ırddisp.pl?gene=LMO2
ırddisp.pl?gene=PTK7
ırddisp.pl?gene=ACR
ırddisp.pl?gene=TBC1D20
ırddisp.pl?gene=KIF2A
ırddisp.pl?gene=TLN1
ırddisp.pl?gene=AVPR1A
ırddisp.pl?gene=RPL32
ırddisp.pl?gene=LTA4H
ırddisp.pl?gene=SGIP1
ırddisp.pl?gene=NYAP2
ırddisp.pl?gene=CNTF
ırddisp.pl?gene=SLC5A6
ırddisp.pl?gene=GABRB2
ırddisp.pl?gene=EML1
ırddisp.pl?gene=HRG
ırddisp.pl?gene=INPP5D
ırddisp.pl?gene=IL22
ırddisp.pl?gene=OASL
ırddisp.pl?gene=NOLC1
ırddisp.pl?gene=MGAT1
ırddisp.pl?gene=GRM5
ırddisp.pl?gene=OSBP
ırddisp.pl?gene=LOC109029530
ırddisp.pl?gene=TRADD
ırddisp.pl?gene=BRD9
ırddisp.pl?gene=LOXL3
ırddisp.pl?gene=CHRNA5
ırddisp.pl?gene=RTN4

ırddisp.pl?gene=ACTN1
ırddisp.pl?gene=ARHGAP18
ırddisp.pl?gene=CYSLTR2
ırddisp.pl?gene=CXorf56
ırddisp.pl?gene=PTPRO
ırddisp.pl?gene=ALYREF
ırddisp.pl?gene=CCL28
ırddisp.pl?gene=HMOX2
ırddisp.pl?gene=ABCC11
ırddisp.pl?gene=TFCP2
ırddisp.pl?gene=MAN2A1
ırddisp.pl?gene=ESYT1
ırddisp.pl?gene=FOXD1
ırddisp.pl?gene=CHRNB4
ırddisp.pl?gene=ITK
ırddisp.pl?gene=SMURF2
ırddisp.pl?gene=MED15
ırddisp.pl?gene=NAT8
ırddisp.pl?gene=MYO7A
ırddisp.pl?gene=MFHAS1
ırddisp.pl?gene=PIGN
ırddisp.pl?gene=DLX2
ırddisp.pl?gene=SLC6A8
ırddisp.pl?gene=HTR5A
ırddisp.pl?gene=MIR582
ırddisp.pl?gene=LILRB1
ırddisp.pl?gene=KAT2A
ırddisp.pl?gene=HDAC7
ırddisp.pl?gene=RBCK1
ırddisp.pl?gene=DNAJA3
ırddisp.pl?gene=LEFTY1
ırddisp.pl?gene=HECTD4
ırddisp.pl?gene=FMN2
ırddisp.pl?gene=LEPROTL1
ırddisp.pl?gene=MERTK
ırddisp.pl?gene=PSME1
ırddisp.pl?gene=FOLH1
ırddisp.pl?gene=RTRAF
ırddisp.pl?gene=ENDOG
ırddisp.pl?gene=NEK4
ırddisp.pl?gene=TAX1BP1
ırddisp.pl?gene=EBF2
ırddisp.pl?gene=GDF3
ırddisp.pl?gene=GDF10
ırddisp.pl?gene=ADRA2B
ırddisp.pl?gene=ERRFI1
ırddisp.pl?gene=SFPQ
ırddisp.pl?gene=ABCA7
ırddisp.pl?gene=CRY2
ırddisp.pl?gene=DUX4
ırddisp.pl?gene=NDUFA4
ırddisp.pl?gene=PTPN9
ırddisp.pl?gene=ALDH1B1
ırddisp.pl?gene=CHRNA6

ırddisp.pl?gene=CHRNA9
ırddisp.pl?gene=BMP5
ırddisp.pl?gene=CHRNB3
ırddisp.pl?gene=B3GNT5
ırddisp.pl?gene=SLC2A12
ırddisp.pl?gene=BMP8B
ırddisp.pl?gene=GDF7
ırddisp.pl?gene=DOK5
ırddisp.pl?gene=CHRNA10
ırddisp.pl?gene=GRK3
ırddisp.pl?gene=HIVEP1
ırddisp.pl?gene=FGFR1OP2
ırddisp.pl?gene=ZNF71
ırddisp.pl?gene=TENT5A
ırddisp.pl?gene=CFAP94
ırddisp.pl?gene=HSD17B10
ırddisp.pl?gene=ORC2
ırddisp.pl?gene=SNRPD1
ırddisp.pl?gene=CYB5B
ırddisp.pl?gene=STX5
ırddisp.pl?gene=STX8
ırddisp.pl?gene=TMEM230
ırddisp.pl?gene=TMEM201
ırddisp.pl?gene=RUSF1
ırddisp.pl?gene=RPS6KA5
ırddisp.pl?gene=SMARCC2
ırddisp.pl?gene=SEL1L
ırddisp.pl?gene=TMEM147
ırddisp.pl?gene=PPP1CA
ırddisp.pl?gene=EIF4B
ırddisp.pl?gene=UGT1A
ırddisp.pl?gene=GTF3A
ırddisp.pl?gene=FZD2
ırddisp.pl?gene=USP4
ırddisp.pl?gene=SYVN1
ırddisp.pl?gene=ANO6
ırddisp.pl?gene=KPNA6
ırddisp.pl?gene=NCBP1
ırddisp.pl?gene=DAB2IP
ırddisp.pl?gene=PPP1R14A
ırddisp.pl?gene=NONO
ırddisp.pl?gene=BRAT1
ırddisp.pl?gene=MMP25
ırddisp.pl?gene=ADRA2C
ırddisp.pl?gene=CDC34
ırddisp.pl?gene=COPS2
ırddisp.pl?gene=FGF20
ırddisp.pl?gene=UGGT2
ırddisp.pl?gene=SPRR1A
ırddisp.pl?gene=ANAPC10
ırddisp.pl?gene=KPNA1
ırddisp.pl?gene=PICALM
ırddisp.pl?gene=TNFAIP8L2
ırddisp.pl?gene=SLC25A11

[illegible]rddisp.pl?gene=PAFAH1B2
[illegible]rddisp.pl?gene=RANBP9
[illegible]rddisp.pl?gene=MAPK11
[illegible]rddisp.pl?gene=TRIM22
[illegible]rddisp.pl?gene=SYNGR1
[illegible]rddisp.pl?gene=MLC1
[illegible]rddisp.pl?gene=USO1
[illegible]rddisp.pl?gene=RRBP1
[illegible]rddisp.pl?gene=EMC1
[illegible]rddisp.pl?gene=KPNA3
[illegible]rddisp.pl?gene=ZC3HAV1
[illegible]rddisp.pl?gene=GTF2B
[illegible]rddisp.pl?gene=CUL2
[illegible]rddisp.pl?gene=OS9
[illegible]rddisp.pl?gene=EDEM1
[illegible]rddisp.pl?gene=LCP2
[illegible]rddisp.pl?gene=TSPAN7
[illegible]rddisp.pl?gene=LOC109504728
[illegible]rddisp.pl?gene=MBD1
[illegible]rddisp.pl?gene=DDX20
[illegible]rddisp.pl?gene=TRIM13
[illegible]rddisp.pl?gene=PDZD8
[illegible]rddisp.pl?gene=AAVS1
