# Supplementary material for: Hyperin Alleviates Triptolide-Induced Ovarian Granulosa Cell Injury by Regulating AKT/TSC1/mTORC1 Signaling
Source: Evid Based Complement Alternat Med. 2021 Oct 18;2021:9399261. doi: 10.1155/2021/9399261 (PMC8545507; doi:10.1155/2021/9399261)
Supplement: Supplementary Materials — Supplementary 1: the predicted target proteins of HR. Supplementary 2: the predicted targets of POI. Supplementary 3: the interaction targets of HR and POI. Supplementary 4: the degree value, betweenness centrality, and closeness centrality of the interaction targets of HR and POI analyzed using PPI. Supplementary 5: the details of predicted KEGG pathways of interaction targets of HR and POI. [file 9399261.f1.zip › 9399261.f1/Supplementary 3-Common targets of hyperin and POI.pdf]

AKT1
CYP19A1
TERT
MMP2
EGFR
KISS1R
TNF
VEGFA
CHEK2
SRC
INSR
ITGB3
MET
PIK3R1
CHEK1
MMP9
IL2
IGF1R
KDR
CCND1
PTGS2
PRKCD
DNMT1
FLNB
PTGS1
CDK1
HFE
RAC1
RARB
PIK3CG
MYLK
PLG
ITGB1
PLK1
ERCC4
RPS6KA3
MMP3
NOS2
PTK2
CCNE1
PRSS1
MCL1
PRKCA
ABCG2
TOP2A
MPO
ALOX5
PPARA
ZEB2
B2M
BCKDHA
AKR1B1
AURKB
HIBCH

F10
XDH
GSK3B
GAD2
MMP13
APP
CYP1B1
ITGAV
DPP4
MAPT
ACHE
UAP1
CA2
ALDH2
ALK
SLC29A1
HSD17B2
MAOA
PRKCB
FLT3
SRSF1
GLO1
SYK
PRKCE
PLA2G1B
ITGA5
CXCR1
IDE
NEK2
TRIM21
NOX4
ITGA2B
ADORA2A
HSP90AB1
DAPK1
UBE2I
HCK
CSNK2A1
PKN1
RNF8
DRD4
CA4
ITGB6
ALOX15
NCBP1
ADRA2C
