# Supplementary material for: Hyperin Alleviates Triptolide-Induced Ovarian Granulosa Cell Injury by Regulating AKT/TSC1/mTORC1 Signaling
Source: Evid Based Complement Alternat Med. 2021 Oct 18;2021:9399261. doi: 10.1155/2021/9399261 (PMC8545507; doi:10.1155/2021/9399261)
Supplement: Supplementary Materials — Supplementary 1: the predicted target proteins of HR. Supplementary 2: the predicted targets of POI. Supplementary 3: the interaction targets of HR and POI. Supplementary 4: the degree value, betweenness centrality, and closeness centrality of the interaction targets of HR and POI analyzed using PPI. Supplementary 5: the details of predicted KEGG pathways of interaction targets of HR and POI. [file 9399261.f1.zip › 9399261.f1/Supplementary 4-PPI.pdf]

SUID	AverageSh	BetweennessC	ClosenessCe	ClusteringC	Degree	Eccentr	IsSingle	name
95	1.443299	0.16125554	0.69285714	0.24444444	55	3	FALSE	AKT1
108	1.5876289	0.09795359	0.62987013	0.28080808	45	3	FALSE	TNF
91	1.5051546	0.08876098	0.66438356	0.28705882	51	3	FALSE	VEGFA
93	1.5463918	0.06728224	0.64666667	0.32657005	46	3	FALSE	EGFR
77	1.5876289	0.05773451	0.62987013	0.33913043	46	3	FALSE	SRC
216	1.7113402	0.0466183	0.58433735	0.36666667	36	3	FALSE	MMP9
73	1.7216495	0.03905775	0.58083832	0.40998217	34	3	FALSE	CCND1
138	2.0618557	0.03883029	0.485	0.38888889	9	3	FALSE	AKR1B1
112	1.7010309	0.03832372	0.58787879	0.38324421	34	3	FALSE	PTGS2
228	1.9175258	0.03145988	0.52150538	0.40692641	22	3	FALSE	CHEK1
207	1.9690722	0.03095449	0.5078534	0.36764706	17	3	FALSE	DNMT1
221	1.8865979	0.0251488	0.53005464	0.39920949	23	3	FALSE	CDK1
131	2.0824742	0.02477055	0.48019802	0.38095238	15	4	FALSE	PRKCE
162	2.3092784	0.0218048	0.43303571	0.2	5	4	FALSE	SRSF1
224	2.0412371	0.02121836	0.48989899	0.56363636	11	3	FALSE	CYP19A1
115	1.9690722	0.02032771	0.5078534	0.31617647	17	3	FALSE	MAPT
105	1.8969072	0.01868358	0.52717391	0.41904762	21	3	FALSE	APP
72	2.0515464	0.01816556	0.48743719	0.43939394	12	3	FALSE	ABCG2
97	2.0412371	0.01714928	0.48989899	0.37878788	12	3	FALSE	ACHE
288	2	0.01650746	0.5	0.3956044	14	3	FALSE	B2M
231	1.814433	0.01504717	0.55113636	0.43103448	29	3	FALSE	PIK3R1
87	1.7938144	0.01500876	0.55747126	0.5	29	3	FALSE	ITGB1
89	1.7628866	0.01373056	0.56725146	0.47126437	30	3	FALSE	KDR
184	1.9690722	0.01317929	0.5078534	0.63970588	17	3	FALSE	PLG
100	1.9072165	0.01285202	0.52432432	0.39393939	22	3	FALSE	MPO
166	2.0309278	0.01264208	0.49238579	0.48529412	17	3	FALSE	ITGA2B
129	1.9381443	0.01170852	0.51595745	0.42690058	19	3	FALSE	PRKCB
154	1.8453608	0.01132331	0.54189944	0.54769231	26	3	FALSE	MMP2
136	1.9587629	0.01131407	0.51052632	0.39047619	21	4	FALSE	PRKCA
212	1.8865979	0.01051302	0.53005464	0.52813853	22	3	FALSE	MCL1
267	1.9896907	0.00987479	0.50259067	0.35294118	18	4	FALSE	SYK
308	2.0927835	0.00973869	0.47783251	0.44166667	16	3	FALSE	TOP2A
203	1.9175258	0.00920622	0.52150538	0.46320346	22	3	FALSE	RAC1
214	1.9484536	0.00914133	0.51322751	0.36842105	19	3	FALSE	HSP90AB1
201	2.1134021	0.00814749	0.47317073	0.42424242	12	3	FALSE	UBE2I
134	2.0206186	0.00749104	0.49489796	0.35238095	15	4	FALSE	PRKCD
157	1.8659794	0.00718726	0.5359116	0.54545455	23	3	FALSE	IL2
119	2.2061856	0.00712245	0.45327103	0.6	5	4	FALSE	ADORA2A
174	1.8659794	0.00644683	0.5359116	0.53985507	24	3	FALSE	IGF1R
197	1.8865979	0.00631653	0.53005464	0.54	25	3	FALSE	PTK2
110	2.4123711	0.00614935	0.41452991	0.36111111	9	4	FALSE	ADRA2C
235	1.9381443	0.00598293	0.51595745	0.56277056	22	3	FALSE	ITGB3
98	2.185567	0.00554498	0.45754717	0.47619048	7	3	FALSE	DRD4
466	2.0206186	0.0053519	0.49489796	0.58823529	18	4	FALSE	ITGAV
179	1.9484536	0.00445431	0.51322751	0.70833333	16	3	FALSE	MMP3
297	2.3608247	0.00440393	0.42358079	0.55128205	13	4	FALSE	PLK1
186	1.9484536	0.00439299	0.51322751	0.55882353	17	3	FALSE	TERT
293	2.2989691	0.00416594	0.43497758	0.54545455	12	4	FALSE	AURKB
83	1.9381443	0.00367817	0.51595745	0.5952381	21	4	FALSE	MET
160	2.0206186	0.00347668	0.49489796	0.53030303	12	3	FALSE	FLT3
190	1.9484536	0.00335253	0.51322751	0.52205882	17	3	FALSE	GSK3B
117	2.8453608	0.00333288	0.35144928	0.3	5	4	FALSE	MAOA
127	2.072165	0.00330948	0.48258706	0.55555556	10	3	FALSE	CXCR1

300	2.3814433	0.00308505	0.41991342	0.62121212	12	4	FALSE	CHEK2
168	2.072165	0.0029416	0.48258706	0.61538462	14	3	FALSE	CCNE1
81	2.5257732	0.00279974	0.39591837	0.3	5	4	FALSE	CYP1B1
303	2.0927835	0.00242015	0.47783251	0.61111111	9	4	FALSE	MYLK
152	2.1030928	0.00238243	0.4754902	0.60714286	8	3	FALSE	CSNK2A1
170	2.0618557	0.00218319	0.485	0.77777778	10	3	FALSE	NOX4
194	2.2989691	0.00201062	0.43497758	0.66666667	4	4	FALSE	RARB
219	2.1030928	0.0019261	0.4754902	0.60606061	12	4	FALSE	ALOX5
164	2.072165	0.00185982	0.48258706	0.84444444	10	3	FALSE	MMP13
139	2.9690722	0.00157725	0.33680556	0	3	4	FALSE	ALDH2
192	2.072165	0.00155577	0.48258706	0.65454545	11	3	FALSE	NOS2
482	2.072165	0.00155113	0.48258706	0.75641026	13	4	FALSE	ITGA5
226	2.1134021	0.00144824	0.47317073	0.39285714	8	3	FALSE	PPARA
387	2.2680412	0.0014246	0.44090909	0.66666667	6	3	FALSE	RNF8
103	2.1443299	0.00137083	0.46634615	0.47619048	7	3	FALSE	IDE
391	2.257732	0.00126285	0.44292237	0.61111111	9	4	FALSE	FLNB
305	2.6907217	0.0011119	0.37164751	0.66666667	6	4	FALSE	NEK2
271	2.1546392	0.00104078	0.46411483	0.55555556	10	4	FALSE	HCK
205	2.0927835	0.00103689	0.47783251	0.57777778	10	3	FALSE	PIK3CG
181	2.1134021	9.72E-04	0.47317073	0.63636364	12	3	FALSE	INSR
238	3.0103093	8.94E-04	0.33219178	0	2	5	FALSE	GAD2
254	2.2268041	8.35E-04	0.44907407	0.64444444	10	4	FALSE	PTGS1
199	2.1237113	5.56E-04	0.47087379	0.61904762	7	3	FALSE	PKN1
509	2.3298969	5.49E-04	0.42920354	0.77777778	10	4	FALSE	ITGB6
188	2.1546392	5.32E-04	0.46411483	0.83333333	9	4	FALSE	PLA2G1B
79	2.2989691	4.35E-04	0.43497758	0.33333333	4	4	FALSE	CA4
241	2.2061856	3.56E-04	0.45327103	0.71111111	10	4	FALSE	ALK
75	2.3298969	3.32E-04	0.42920354	0.5	5	4	FALSE	KISS1R
446	2.7525773	2.63E-04	0.36329588	0.33333333	3	4	FALSE	DAPK1
144	2.4742268	2.22E-04	0.40416667	0.66666667	4	4	FALSE	XDH
149	3	1.91E-04	0.33333333	0	2	4	FALSE	GLO1
252	2.2680412	1.23E-04	0.44090909	0.9047619	7	4	FALSE	ALOX15
322	2.7938144	1.04E-04	0.35793358	0	2	4	FALSE	HFE
85	2.9587629	1.01E-04	0.33797909	0	2	4	FALSE	SLC29A1
278	2.556701	5.69E-05	0.39112903	0.66666667	4	4	FALSE	PRSS1
329	2.5773196	4.96E-05	0.388	0	2	4	FALSE	CA2
233	2.1237113	3.68E-05	0.47087379	0.95238095	7	3	FALSE	ZEB2
336	2.371134	2.69E-05	0.42173913	0.66666667	3	4	FALSE	RPS6KA3
209	2.1340206	0	0.46859903	1	7	3	FALSE	DPP4
313	2.5360825	0	0.39430894	1	2	4	FALSE	TRIM21
326	2.4845361	0	0.40248963	1	2	4	FALSE	BCKDHA
400	2.8865979	0	0.34642857	1	3	4	FALSE	ERCC4
433	2.9896907	0	0.33448276	1	2	4	FALSE	HSD17B2
504	2.8350516	0	0.35272727	1	2	4	FALSE	F10
774	3.2989691	0	0.303125	0	1	5	FALSE	NCBP1

NeighborhoodConnectivity	NumberOfDirectedEdges	NumberOfUndirectedEdges	PartnerOfMultiEdgedNodePairs	Radiality	selected	SelfLoops	shared name	Stress
18.52727	55	0	0	0.91134	FALSE	0	AKT1	8470
18.71111	45	0	0	0.882474	FALSE	0	TNF	5192
19.56863	51	0	0	0.898969	FALSE	0	VEGFA	6002
21.17391	46	0	0	0.890722	FALSE	0	EGFR	4930
21.15217	46	0	0	0.882474	FALSE	0	SRC	4046
21.44444	36	0	0	0.857732	FALSE	0	MMP9	3108
23.23529	34	0	0	0.85567	FALSE	0	CCND1	3314
28.77778	9	0	0	0.787629	FALSE	0	AKR1B1	1916
21.55882	34	0	0	0.859794	FALSE	0	PTGS2	2782
20.40909	22	0	0	0.816495	FALSE	0	CHEK1	2478
22.23529	17	0	0	0.806186	FALSE	0	DNMT1	2080
20.69565	23	0	0	0.82268	FALSE	0	CDK1	2102
19.8	15	0	0	0.783505	FALSE	0	PRKCE	1454
16.4	5	0	0	0.738144	FALSE	0	SRSF1	778
31.54545	11	0	0	0.791753	FALSE	0	CYP19A1	1266
20	17	0	0	0.806186	FALSE	0	MAPT	1170
24.42857	21	0	0	0.820619	FALSE	0	APP	1462
27.33333	12	0	0	0.789691	FALSE	0	ABCG2	1238
21.33333	12	0	0	0.791753	FALSE	0	ACHE	1062
25.71429	14	0	0	0.8	FALSE	0	B2M	1186
23.55172	29	0	0	0.837113	FALSE	0	PIK3R1	1248
25.13793	29	0	0	0.841237	FALSE	0	ITGB1	1446
25.33333	30	0	0	0.847423	FALSE	0	KDR	1316
28.64706	17	0	0	0.806186	FALSE	0	PLG	962
20.54545	22	0	0	0.818557	FALSE	0	MPO	896
23.82353	17	0	0	0.793814	FALSE	0	ITGA2B	1014
23.05263	19	0	0	0.812371	FALSE	0	PRKCB	966
25.76923	26	0	0	0.830928	FALSE	0	MMP2	1048
20.95238	21	0	0	0.808247	FALSE	0	PRKCA	946
27.81818	22	0	0	0.82268	FALSE	0	MCL1	1354
20.38889	18	0	0	0.802062	FALSE	0	SYK	652
18.1875	16	0	0	0.781443	FALSE	0	TOP2A	814
23.95455	22	0	0	0.816495	FALSE	0	RAC1	774
22.05263	19	0	0	0.810309	FALSE	0	HSP90AB1	734
16.91667	12	0	0	0.77732	FALSE	0	UBE2I	494
21.06667	15	0	0	0.795876	FALSE	0	PRKCD	594
27.56522	23	0	0	0.826804	FALSE	0	IL2	676
35	5	0	0	0.758763	FALSE	0	ADORA2A	418
26.95833	24	0	0	0.826804	FALSE	0	IGF1R	682
25.4	25	0	0	0.82268	FALSE	0	PTK2	740
13.88889	9	0	0	0.717526	FALSE	0	ADRA2C	340
26.18182	22	0	0	0.812371	FALSE	0	ITGB3	748
18.57143	7	0	0	0.762887	FALSE	0	DRD4	382
24.16667	18	0	0	0.795876	FALSE	0	ITGAV	472
31.25	16	0	0	0.810309	FALSE	0	MMP3	484
15.46154	13	0	0	0.727835	FALSE	0	PLK1	422
28.11765	17	0	0	0.810309	FALSE	0	TERT	620
15.83333	12	0	0	0.740206	FALSE	0	AURKB	256
27.7619	21	0	0	0.812371	FALSE	0	MET	390
28.91667	12	0	0	0.795876	FALSE	0	FLT3	374
28.94118	17	0	0	0.810309	FALSE	0	GSK3B	410
6.6	5	0	0	0.630928	FALSE	0	MAOA	100
30.2	10	0	0	0.785567	FALSE	0	CXCR1	372

16.16667	12	0	0	0.723711	FALSE	0	CHEK2	274
24.42857	14	0	0	0.785567	FALSE	0	CCNE1	420
17	5	0	0	0.694845	FALSE	0	CYP1B1	168
29.55556	9	0	0	0.781443	FALSE	0	MYLK	276
25.375	8	0	0	0.779381	FALSE	0	CSNK2A1	176
36.9	10	0	0	0.787629	FALSE	0	NOX4	236
27.25	4	0	0	0.740206	FALSE	0	RARB	162
26.91667	12	0	0	0.779381	FALSE	0	ALOX5	170
33	10	0	0	0.785567	FALSE	0	MMP13	222
5.333333	3	0	0	0.606186	FALSE	0	ALDH2	86
30.63636	11	0	0	0.785567	FALSE	0	NOS2	126
29.61538	13	0	0	0.785567	FALSE	0	ITGA5	258
27.875	8	0	0	0.77732	FALSE	0	PPARA	136
21.16667	6	0	0	0.746392	FALSE	0	RNF8	182
23.85714	7	0	0	0.771134	FALSE	0	IDE	122
21.11111	9	0	0	0.748454	FALSE	0	FLNB	150
15.16667	6	0	0	0.661856	FALSE	0	NEK2	52
27.5	10	0	0	0.769072	FALSE	0	HCK	112
30.1	10	0	0	0.781443	FALSE	0	PIK3CG	112
27.16667	12	0	0	0.77732	FALSE	0	INSR	122
9	2	0	0	0.597938	FALSE	0	GAD2	44
23	10	0	0	0.754639	FALSE	0	PTGS1	66
24.28571	7	0	0	0.775258	FALSE	0	PKN1	64
21.9	10	0	0	0.734021	FALSE	0	ITGB6	78
28.11111	9	0	0	0.769072	FALSE	0	PLA2G1B	58
23	4	0	0	0.740206	FALSE	0	CA4	36
30	10	0	0	0.758763	FALSE	0	ALK	44
23.8	5	0	0	0.734021	FALSE	0	KISS1R	40
12.66667	3	0	0	0.649485	FALSE	0	DAPK1	16
21.5	4	0	0	0.705155	FALSE	0	XDH	12
7	2	0	0	0.6	FALSE	0	GLO1	12
28.14286	7	0	0	0.746392	FALSE	0	ALOX15	14
15.5	2	0	0	0.641237	FALSE	0	HFE	12
8.5	2	0	0	0.608247	FALSE	0	SLC29A1	6
20.75	4	0	0	0.68866	FALSE	0	PRSS1	6
26.5	2	0	0	0.684536	FALSE	0	CA2	6
38.42857	7	0	0	0.775258	FALSE	0	ZEB2	10
32.33333	3	0	0	0.725773	FALSE	0	RPS6KA3	2
37.85714	7	0	0	0.773196	FALSE	0	DPP4	0
29.5	2	0	0	0.692784	FALSE	0	TRIM21	0
31.5	2	0	0	0.703093	FALSE	0	BCKDHA	0
15.66667	3	0	0	0.62268	FALSE	0	ERCC4	0
8	2	0	0	0.602062	FALSE	0	HSD17B2	0
17	2	0	0	0.63299	FALSE	0	F10	0
5	1	0	0	0.540206	FALSE	0	NCBP1	0

TopologicalCoefficient
0.192992
0.205372
0.208177
0.222883
0.232441
0.243371
0.25817
0.356944
0.234335
0.245893
0.273057
0.245859
0.269406
0.262295
0.384701
0.243185
0.284053
0.348291
0.263374
0.315697
0.273857
0.285658
0.281481
0.345145
0.246988
0.309396
0.274436
0.299642
0.258671
0.323467
0.251715
0.252604
0.288609
0.265694
0.230594
0.2625
0.316842
0.483333
0.313469
0.306024
0.267094
0.323232
0.259557
0.313131
0.363372
0.291727
0.330796
0.271552
0.334481
0.348394
0.340484
0.31
0.3775

0.310897
0.321429
0.381818
0.372714
0.321203
0.455556
0.419231
0.354167
0.4125
0.361111
0.387802
0.379684
0.357372
0.328125
0.31619
0.318182
0.454545
0.376712
0.385897
0.367117
0.5
0.343284
0.311355
0.377586
0.37988
0.348485
0.434783
0.390164
0.425287
0.421569
0.545455
0.426407
0.58
0.576923
0.461111
0.579545
0.492674
0.530055
0.491651
0.614583
0.605769
0.712121
0.615385
0.62963
0
